# GaitSmart motion analysis compared to commonly used function outcome measures in the IMI-APPROACH knee osteoarthritis cohort

**DOI:** 10.1371/journal.pone.0265883

**Published:** 2022-03-23

**Authors:** Eefje M. van Helvoort, D. Hodgins, Simon C. Mastbergen, Anne C. A. Marijnissen, M. Kloppenburg, Fransisco J. Blanco, Ida K. Haugen, F. Berenbaum, Floris P. J. G. Lafeber, Paco M. J. Welsing

**Affiliations:** 1 Department of Rheumatology & Clinical Immunology, University Medical Center Utrecht, Utrecht University, Utrecht, The Netherlands; 2 Dynamic Metrics Limited, Codicote, United Kingdom; 3 Department of Rheumatology, Leiden University Medical Center, Leiden, The Netherlands; 4 Department of Clinical Epidemiology, Leiden University Medical Center, Leiden, The Netherlands; 5 Servicio de Reumatología, INIBIC-Hospital Universitario A Coruña, Grupo de Investigación Reumatologia, Agrupación CICA-INIBIC, Departamento de Fisioterapia, Medicina y Ciencias Biomédicas, Universidad de A Coruña, A Coruña, Spain; 6 Department of Rheumatology, Diakonhjemmet Hospital, Oslo, Norway; 7 Sorbonne Université, Institut National de la Santé et de la Recherché Médicale (INSERM), APHP hôpital Saint-Antoine, Paris, France; Mugla Sitki Kocman Universitesi, TURKEY

## Abstract

**Background:**

There are multiple measures for assessment of physical function in knee osteoarthritis (OA), but each has its strengths and limitations. The GaitSmart® system, which uses inertial measurement units (IMUs), might be a user-friendly and objective method to assess function. This study evaluates the validity and responsiveness of GaitSmart® motion analysis as a function measurement in knee OA and compares this to Knee Injury and Osteoarthritis Outcome Score (KOOS), Short Form 36 Health Survey (SF-36), 30s chair stand test, and 40m self-paced walk test.

**Methods:**

The 2-year Innovative Medicines Initiative—Applied Public-Private Research enabling OsteoArthritis Clinical Headway (IMI-APPROACH) knee OA cohort was conducted between January 2018 and April 2021. For this study, available baseline and 6 months follow-up data (n = 262) was used. Principal component analysis was used to investigate whether above mentioned function instruments could represent one or more function domains. Subsequently, linear regression was used to explore the association between GaitSmart® parameters and those function domains. In addition, standardized response means, effect sizes and t-tests were calculated to evaluate the ability of GaitSmart® to differentiate between good and poor general health (based on SF-36). Lastly, the responsiveness of GaitSmart® to detect changes in function was determined.

**Results:**

KOOS, SF-36, 30s chair test and 40m self-paced walk test were first combined into one function domain (*total function*). Thereafter, two function domains were substracted related to either performance based (*objective function*) or self-reported (*subjective function*) function. Linear regression resulted in the highest R^2^ for the total function domain: 0.314 (R^2^ for objective and subjective function were 0.252 and 0.142, respectively.). Furthermore, GaitSmart® was able to distinguish a difference in general health status, and is responsive to changes in the different aspects of objective function (Standardized response mean (SRMs) up to 0.74).

**Conclusion:**

GaitSmart® analysis can reflect performance based and self-reported function and may be of value in the evaluation of function in knee OA. Future studies are warranted to validate whether GaitSmart® can be used as clinical outcome measure in OA research and clinical practice.

## Introduction

There are multiple measures for assessment of physical function in knee osteoarthritis (OA) [1], and each has its strengths and limitations. Gait analysis in an optical gait lab is often used as gold standard [2, 3] but has disadvantages. It is not always available, very costly, and time consuming. For performance-based tests (PBT) the opposite is true, they are easily performed in an everyday environment and take a few minutes. Limitations of PBT are the poor construct validity and responsiveness to change [4, 5]. Besides, PBT do not give any information on quality of movement, in contrast to gait analysis [6]. PBT and gait analysis are said to be objective measures [7, 8], containing information about the ability to complete a task. Self-reported measures, like the Knee injury and Osteoarthritis Outcome Score (KOOS) and Short Form 36 Health Survey (SF-36) give information concerning the experience associated with doing the task. Patients are not simply reporting their ability to move around, but their response also includes what they are experiencing during a task [9]. Construct validity and responsiveness to change are better for KOOS than PBT, including subscales activities of daily living and sport and recreational function [10]. However, self-reported function is more influenced by pain than performance based function [11, 12]. Pain while performing a task will influence the experience of doing the task, but might not always influence the ability to perform a task. As such, subjective function might be more influenced by pain than objective function.

Self-reported and performance based measures assess different aspects of function (experience vs ability) and are poorly correlated [9, 13]. The first month after total knee arthroplasty, PBT and self-reported measures show inverse trajectories of improvement. The poor concurrent validity between both measures implicates that using solely self-reported measures or PBT is not sufficient to fully characterize function [8, 12]. Variable correlations between self-reported function and gait parameters have been found as well [14–16]. As such, PBT (objective) and self-reported function (subjective) offer complementary information, essential to clinical research and practice [1, 8, 9].

The GaitSmart® system, which uses inertial measurement units (IMUs), might be a user-friendly and more objective method to assess function. Because no pressure plates or cameras are needed, it can be carried out virtually everywhere, taking approximately 10–15 minutes. When comparing the use of IMUs to 3D analysis in an optical gait lab, no differences were found in e.g. determining pelvic tilt and knee range of motion (ROM) [3]. It has been shown previously, that GaitSmart® analysis gives additional information over Patient Reported Outcome Measures (PROMs) and radiographic outcomes for OA [17].

As said, commonly used function measures all have their limitations and gait analysis using the GaitSmart® system might overcome these limitations and provide an easy applicable, objective measurement for physical function with good validity and responsiveness to change. The objective of this study was to investigate construct validity and responsiveness of GaitSmart® as measurement of function in knee OA. For this purpose, multiple questions were answered. i. Is GaitSmart^®^ related with commonly used outcome measures for function? ii. Can GaitSmart® differentiate between groups with different general health status? iii. Is GaitSmart® able to measure change in function over a six month period? We hypothesize that GaitSmart® is a more objective measurement for physical function compared to questionnaires, and is more precise compared to PBT.

## Materials and methods

### Participants

297 people with knee OA were included in the Innovative Medicines Initiative—Applied Public-Private Research enabling OsteoArthritis Clinical Headway (IMI-APPROACH) cohort study from January 2018 until April 2019 (age; 66.5±7.1, female; 230 (77%), BMI; 28.1±5.3) [18]. The IMI-APPROACH is an international multicenter, prospective observational cohort study with the ultimate aim to predict disease progression and define multiple OA phenotypes. The study is being conducted in compliance with the protocol, Good Clinical Practice (GCP), the Declaration of Helsinki, and applicable ethical and legal regulatory requirements (for all countries involved), and is registered under clinicaltrials.gov nr: NCT03883568. All participants have received oral and written information and provided written informed consent.

At screening, for each participant an index knee was determined based on American College of Rheumatology clinical criteria [19] or (if equal between two knees) the most painful knee was chosen as index knee. Screenings’ data was used in machine learning models to determine predicted progression probability for pain (P) and one for structural progression (S) [20]. Participants with the highest predicted progression scores were included in the IMI-APPROACH cohort [18]. For this study, baseline (BL) and six months follow-up (M6) data were used.

### GaitSmart® measurement

The GaitSmart® system uses six IMUs to evaluate gait mechanics. These IMUs comprise three tri-axial accelerometers and three tri-axial gyroscopes, making it possible to measure movements in the sagittal and frontal plane [3]. After attaching the IMUs to the body, participants are asked to walk 15–20 meters at their own self-selected speed and return. Subsequently, data is extracted from the IMUs and analyzed. The resulting report contains ROM of pelvis, hips, thighs, knees in swing and stance phase, and calves in the sagittal plane, stride duration, medial-lateral movement of thighs and calves, and symmetry scores between left and right (extensive description reported previously) [17]. The IMUs are accurate to 0.11°, although the measurement error depends on positioning on the body. A previous study showed a reproducibility of ±2.8° knee ROM in swing [2].

### Performance based tests

Two PBT, recommended by OsteoArthritis Research Society International (OARSI) [21], were used in IMI-APPROACH. For the 30s chair-stand test (chair) participants had to stand up completely from a sitting position in the middle of a seat with feet shoulder width apart, flat on the floor, arms crossed at chest, and then sit completely. The result is the number of repetitions completed in thirty seconds. The 40m self-paced walk test (walk) records time in seconds needed to walk as quickly but as safe as possible (regular walking, no running) to a mark 10m away, return, and repeat for a total distance of 40m. In knee OA patients, intraclass correlation coefficients (ICC) were previously found to be 0.90 (95%CI 0.68–0.96) and 0.93 (95%CI 0.85–0.96) for the chair and walk test, respectively. Construct validity was found to be poor for both tests, as only 5 out of 12 (42%) and 4 out of 25 (27%) of predefined hypotheses were confirmed for the chair and walk test, respectively [4].

### Function questionnaires

Self reported function was evaluated using corresponding subscales of the KOOS questionnaire: activities of daily living (daily function) and sport and recreational function (sports) [22], and the ‘physical functioning’ (SF-36 physical function) and ‘role limitations due to physical health’ scales (SF-36 role physical) of the SF-36 [23]. In OA patients, ICC was found to be 0.89 (95%CI 0.84–0.93) for KOOS daily function and 0.83 (95%CI 0.73–0.89) for KOOS sports. When compared to SF-36 physical function, construct validity of KOOS daily function was 0.65 (95% CI 0.64–0.66) and construct validity of KOOS sports was 0.46 (95%CI 0.44–0.47) [10].

### Statistical analysis

All individual GaitSmart® parameters were used for analyses. Additionally to these individual GaitSmart^®^ parameters, five GaitSmart® domains: GaitSmart® (GS) Knee, GS Hip, GS Difference knee, GS Difference stance, and GS Difference hip, were also used for analyses (S1 Table in S1 File). These domains have been identified previously by use of principal component analysis (PCA) [17].

As there is no ‘gold standard’ instrument to assess function, and several instruments are used, we evaluated whether six commonly used instruments (two PBTs, two KOOS subscales, and two SF-36 subscales) could represent one or multiple function domains (e.g. objective and/or subjective function) using PCA on cross-sectional IMI-APPROACH baseline data. Resulting domain(s) (combining all six common function outcome measures) were used as outcome measures for linear regression analyses.

#### I. Relation between GaitSmart® and common outcome measures for function

Linear regression analysis was used to explore whether individual GaitSmart® parameters were associated with derived function domains. Additionally, these analyses were performed using GaitSmart® domains (instead of individual GaitSmart® parameters) as independent variables (S2 Table in S2 File).

Modelling started with a ‘full model’ including all GaitSmart® parameters. Then GaitSmart® parameters with a p-value >0.2 were removed, starting with the least statistically significant variable. In case the adjusted R^2^ diminished relevantly, the variable was retained. Resulting regression formula(s) were then used to construct GaitSmart® based function scores related to total, objective, and subjective function domains (GS total function, GS objective function, and GS subjective function).

#### II. Differentiation between two groups with different general health status

Participants were divided into two subgroups based on the first question of the SF-36:

“In general, would you say your health is”: 1. Excellent, 2. Very good, 3. Good, 4. Fair, 5. Poor.

The two groups were defined as either ‘poor’ general health (4 and 5) or ‘good’ general health (1 and 2). Cross-sectional data of participants with a succesfull GaitSmart® analysis at baseline were used. T-tests and effect sizes (Hedges’ *g)* were calculated to evaluate whether GaitSmart® is able to differentiate between these groups.

#### III. Six months change in function

Changes from BL to M6 were calculated for each separate function outcome measure and GaitSmart® based function scores. Pearson’s correlation coefficients were calculated to compare changes between commonly used function outcome measures and GaitSmart® based function scores.

Subsequently, patients were divided based on an increase or decrease of at least the minimal detectable change (MDC) in each of the commonly used function outcome measures [10, 24, 25] (those without at least a MDC were left out of analyses). For each of these subgroups standardized response mean (mean change (i.e. M6-BL) in outcome variable divided by the standard deviation of this change) within the subgroup was calculated for the other function outcome measures and GaitSmart® based function scores. The difference in change scores between subgroups was compared using t-tests and effect sizes (Hedges’ *g)* to evaluate responsiveness of GaitSmart® to clinical change, compared to commonly used function outcome measures. An effect size of 0.5–0.8 is considered moderate, an effect size of 0.8 or higher is considered high. Statistical analysis was performed using IBM SPSS statistics version 25.0.0.2. P-values <0.05 were considered statistically significant for all analyses.

## Results

**Fig 1:**
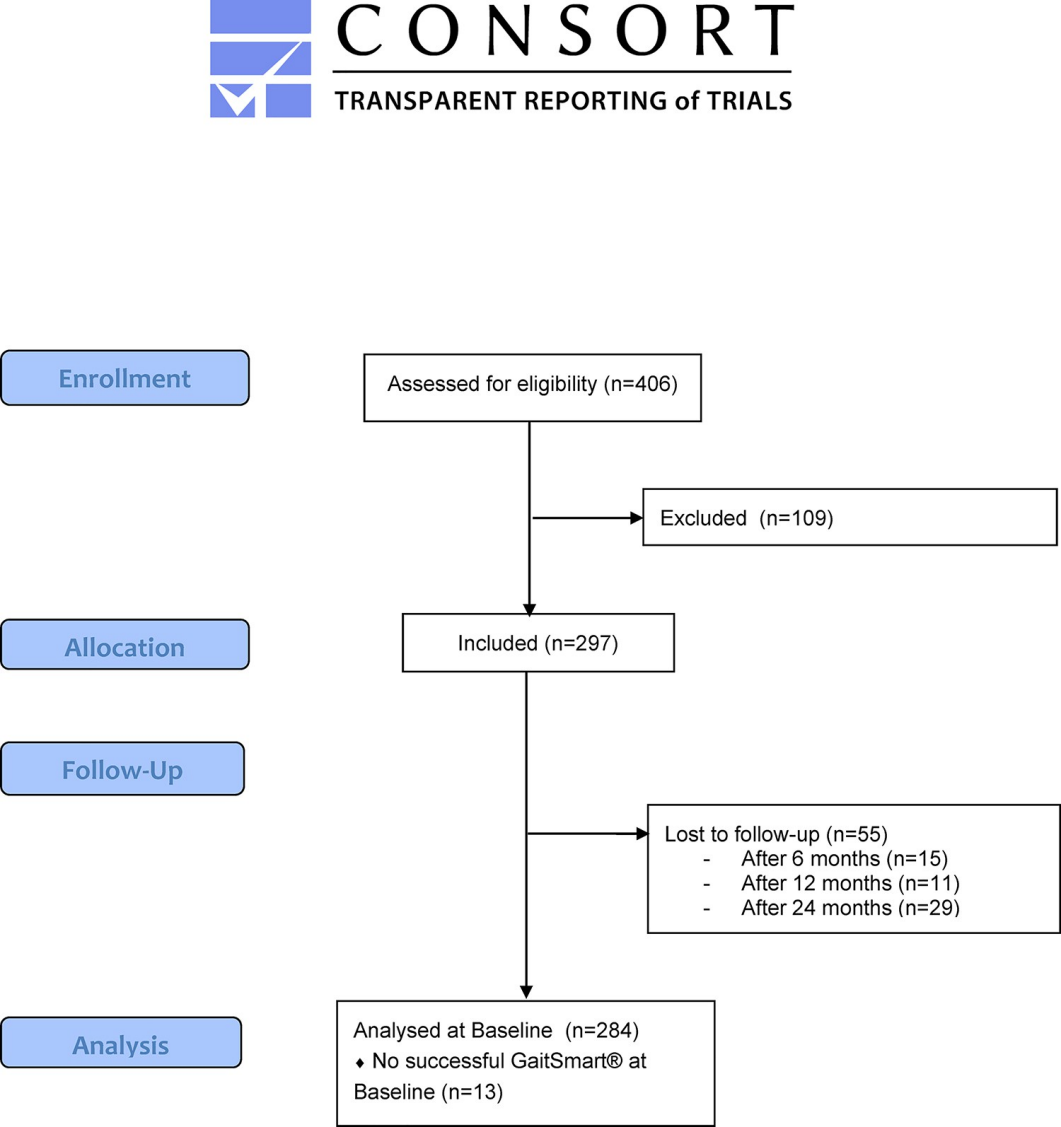
CONSORT flow diagram.

Of the IMI-APPROACH cohort (n = 297), 284 participants had a successful GaitSmart® measurement at baseline, of which 262 also had a successful GaitSmart® measurement at M6. Missing analyses were due to user errors, technical issues, or drop-outs. Two participants could not perform the chair test at M6, while all 262 successfully performed the walk test. Both KOOS subscales were available for all 262 participants, SF-36 physical function was missing for three participants, and SF-36 role physical was missing for one participant. For each of the analyses the maximum available full data set was used (Fig 1).

### Principal component analysis on baseline function outcome measures

Using the default setting of an eigenvalue>1 in the PCA, all six function outcome measures loaded on one domain: *total function*. We also performed a PCA defining extraction of two components, where we found a division into a more *objective function* domain (PBT as main loading factors) and a more *subjective function* domain (KOOS as main loading factors). SF-36 was found to load both components, strongest on subjective function (Table 1).

**Table 1:** Principal component analysis function outcome parameters.

	Total function*	Objective function**	Subjective function**
Chair (times standing up)	0.611	**0.808**	0.110
Walk (s)	0.688	**0.792**	0.225
KOOS daily function	0.847	0.297	**0.863**
KOOS sports	0.730	0.081	**0.895**
SF-36 physical function	0.885	0.517	**0.722**
SF-36 role physical	0.697	0.563	0.435

Loadings are given as absolute values. Bold values indicate strong loadings, indicating a strong correlation between the parameter and that component.

KOOS: Knee injury and Osteoarthritis Outcome Score, SF-36: Short Form 36 Health Survey.

*result of principal component analysis when numbers of extracted domains was not set.

**result of principal component analysis defining extraction of two domains

#### I. Relation between GaitSmart® and derived function domains

In the model for subjective function, only index side variables (ROM and stance flexion index knee and ROM index hip) were statistically significant (Table 2A). The final model had an adjusted R^2^ of 0.141.

**Table 2:** Linear regression analysis for commonly used function domains.

**A. Subjective function domain**				**adjusted R**^**2**^ **0.141**	
*Independent variable*	*B (unstand*.*)*	*95%CI*		*Beta (stand*.*)*	*p-value*
Constant	-2.653	-3.587	-1.720		.000
ROM index knee	.025	.007	.043	.184	.006
Stance flexion index knee	.029	.001	.058	.143	.004
ROM index hip	.022	.006	.039	.167	.009
**B. Objective function domain**				**adjusted R**^**2**^ **0.252**	
*Independent variable*	*B (unstand*.*)*	*95%CI*		*Beta (stand*.*)*	*p-value*
Constant	-3.562	-4.432	-2.691		.000
ROM contralateral knee	.026	.011	.040	.188	.001
Difference range calf	.047	.000	.094	.104	.050
Speed	2.018	1.475	2.560	.413	.000
**C. Total function domain**				**adjusted R**^**2**^ **0.314**	
*Independent variable*	*B (unstand*.*)*	*95%CI*		*Beta (stand*.*)*	*p-value*
Constant	-2.744	-4.138	-1.349		0.000
ROM contralateral knee	.030	0.014	.045	.213	0.000
Stance flexion index knee	.029	0.004	.054	.141	0.023
ROM index hip	.026	0007	.044	.194	0.007
Average duration	-.977	-1.852	-.102	-.119	0.029
Stride length	.760	.025	1.494	.144	0.043

ROM: Range of motion

In the model for objective function, contralateral side (ROM contralateral knee), difference between both sides (difference range calf), and speed were statistically significant (Table 2B). The final model had a higher adjusted R^2^ compared to the model for subjective function; 0.252.

Finally, in the model for total function, parameters for index side (stance flexion index knee and ROM index hip) as well as contralateral side (ROM contralateral knee), and general parameters (average duration and stride length) were statistically significant (Table 2C). The final model had a higher adjusted R^2^: 0.314.

For the final models with GaitSmart® domains instead of individual GaitSmart® parameters as independent variables, see S2-S4 Tables in S2 File. Final adjusted R^2^ for each model were comparable to the ones found with individual GaitSmart^®^ parameters as determinants.

#### II. Differentiation between groups with different general health status

Table 3 shows the results of t-tests and effect sizes in the poor and good general health group. All individual commonly used function outcome measures and all three GaitSmart® based function scores are able to discriminate between participants with poor and good general health. Effect sizes are highest for SF-36 subscales. Nevertheless, all effect sizes were found to be high (>0.8).

**Table 3:** Effect sizes of different functional outcome measures in subgroups based on general health.

	Poor general health	Good general health		
	n = 79	n = 58	Effect size	T-test
	Mean (SD)	Mean (SD)	Hedges’ *g*	p-value
SF-36 physical function	44.43 (18.24)	75.09 (17.31)	1.72	**<0.001**
SF-36 role physical	44.38 (25.35)	81.36 (17.85)	1.64	**<0.001**
KOOS daily function	57.52 (17.48)	80.87 (15.94)	1.39	**<0.001**
Walk (s)	32.99 (8.40)	24.05 (3.96)	1.30	**<0.001**
**GS objective function**	**-0.23 (0.48)**	**0.29 (0.43)**	**1.14**	**<0.001**
**GS total function**	**-0.26 (0.56)**	**0.28 (0.53)**	**0.98**	**<0.001**
KOOS sports	29.75 (22.63)	53.88 (27.47)	0.97	**<0.001**
**GS subjective function**	**-0.19 (0.36)**	**0.13 (0.37)**	**0.88**	**<0.001**
Chair (# standing up)	8.95 (3.08)	11.31 (2.47)	0.83	**<0.001**

GaitSmart® (GS) function outcomes are given in bold. Bold p-values indicate statistically significant p-value (<0.05). KOOS: Knee injury and Osteoarthritis Outcome Score, SF-36: Short Form 36 Health Survey.

Cases were excluded listwise.

### Six months change in function

Table 4 shows Pearson’s correlation coefficients between changes from BL to M6 (M6-BL) for commonly used function outcome measures and GaitSmart® based function scores. Clearly all GaitSmart® based function scores correlated best with PBTs.

**Table 4:** Pearson’s correlation coefficients between changes from baseline to M6 for all used functional outcome measures.

	ΔGS subjective function		ΔGS objective function		ΔGS total function	
	*r*	p-value	*r*	p-value	*r*	p-value
ΔChair (# standing up)	**0.128**	**0.041**	**0.163**	**0.009**	0.092	0.143
ΔWalk (s)	**-0.263**	**<0.001**	**-0.262**	**<0.001**	**-0.311**	**<0.001**
ΔKOOS daily function	0.084	0.176	0.049	0.431	0.095	0.129
ΔKOOS sports	0.045	0.475	0.039	0.533	0.042	0.508
ΔSF-36 physical function	0.096	0.124	0.105	0.093	0.089	0.156
ΔSF-36 role physical	0.084	0.177	0.098	0.116	0.069	0.273

Statistically significant values are given in bold.

GS: GaitSmart®, KOOS: Knee injury and Osteoarthritis Outcome Score, SF-36: Short Form 36 Health Survey.

GaitSmart® appears more related to PBT than to questionnaires as deduced thus far. Therefore, the study population was divided in two groups based on an increase or decrease of at least the MDC on the chair test (Table 5) or the walk test (Table 6). Standardized response mean (SRMs), effect sizes, and results of t-tests between those with an in- or decrease are shown for all function tests.

**Table 5:** Standardized response means and effect sizes of different functional outcome measures in subgroups based on MDC (= 2) of the 30s chair stand up test.

	Worsened chair test		Improved chair test			
	n = 41		n = 79		Effect size	T-test
	M6-BL mean (SD)	SRM	M6-BL mean (SD)	SRM	Hedges’ *g*	p-value
Walk (s)	0.02 (5.69)	0.00	-1.53 (6.66)	-0.23	0.24	0.205
KOOS daily function	-0.61 (16.07)	-0.04	3.11 (13.27)	0.23	0.26	0.179
KOOS sports	-0.37 (21.13)	-0.02	-0.82 (19.49)	-0.04	0.02	0.906
SF-36 physical function	-2.32 (15.05)	-0.15	-0.95 (14.63)	-0.06	0.09	0.631
SF-36 role physical	-5.04 (23.85)	-0.21	-0.63 (19.80)	-0.03	0.21	0.285
GS total function	-0.12 (0.49)	-0.25	0.03 (0.57)	0.05	0.28	0.148
GS objective function	0.00 (0.44)	0.01	0.18 (0.42)	**0.43**	0.41	**0.033**
GS subjective function	-0.09 (0.32)	**-0.26**	0.06 (0.36)	0.16	0.41	**0.035**

SRM: Standardized response mean, M6: 6 months follow-up visit, BL: Baseline, KOOS: Knee injury and Osteoarthritis Outcome Score, SF-36: Short Form 36 Health Survey, GS: GaitSmart®; highest SRM for each in bold; p-values <0.05 in bold.

**Table 6:** Standardized response means and effect sizes of different functional outcome measures in subgroups based on MDC (= 0.19m/s) of the 40m self paced walk test.

	Worsened walk test		Improved walk test			
	n = 43		n = 48		Effect size	T-test
	M6-BL mean (SD)	SRM	M6-BL mean (SD)	SRM	Hedges’ *g*	p-value
Chair (# standing up)	-0.02 (2.47)	-0.00	1.06 (2.32)	0.46	0.45	**0.033**
KOOS daily function	-0.79 (14.98)	-0.05	2.08 (14.62)	0.14	0.19	0.359
KOOS sports	-2.67 (21.39)	-0.13	-6.56 (21.57)	-0.30	0.18	0.391
SF-36 physical function	-3.95 (15.87)	-0.25	-0.83 (16.38)	-0.05	0.19	0.360
SF-36 role physical	-7.27 (21.73)	-0.33	1.69 (22.40)	0.08	0.41	0.057
GS total function	-0.33 (0.52)	**-0.64**	0.17 (0.33)	0.52	1.18	**<0.001**
GS objective function	-0.18 (0.36)	-0.50	0.24 (0.32)	**0.74**	1.23	**<0.001**
GS subjective function	-0.15 (0.35)	-0.44	0.11 (0.24)	0.49	0.92	**<0.001**

SRM: Standardized response mean, M6: 6 months follow-up visit, BL: Baseline, KOOS: Knee injury and Osteoarthritis Outcome Score, SF-36: Short Form 36 Health Survey, GS: GaitSmart®; highest SRM for each in bold; p-values <0.05 in bold.

If there is a decrease in sit-to-stand activity (Table 5), this decrease is most prominently detected by GS subjective (and total) function score. If there is an improvement in sit-to-stand activity, this is also most prominently found in the GS (objective) function score. Effect sizes for worsening compared to improving are highest for all three GaitSmart® based function scores, meaning these are more responsive to detect an actual change in sit-to-stand activity, as compared to commonly used function paramaters, including the walk test.

A decrease in walking activity (Table 6) is most prominently detected by GS total function score, and an improvement in walking activity is most prominently found in GS objective function score. Effect sizes for worsening compared to improving are highest for all three GaitSmart® based function scores, meaning these are more responsive to detect an actual change in walk activity, as compared to commonly used function parameters. Also the chair test shows a statistically significant difference between the worsened and improved walk test group, however with a lower effect size.

For self-reported function outcome measures (KOOS and SF-36 subscales) the same analyses have been performed based on an increase or decrease of at least the MDC (S5-S8 Tables in S3 File). Worsening and improvement in one of the self-reported function outcome measures are most prominently detected by the other self-reported function outcome measures. Effect sizes for worsening compared to improving were low for GaitSmart® based function scores, meaning these GaitSmart® based function scores are minimally responsive to detect an actual change in self-reported function.

Since selection of IMI-APPROACH participants is based on predicted progression scores (P and S, see above) we also evaluated change in commonly used and GaitSmart® based function scores in participants with low predicted progression (P ánd S score below median value) and high predicted progression (P ánd S score above median value). These data have been provided in S9 Table in S4 File and demonstrated that GS total function and GS subjective function were also discriminative between these predicted progression subgroups (S9 Table in S4 File).

## Discussion

GaitSmart® analysis is related to commonly used function outcome measures, specifically more objective outcomes, with good sensitivity to observe short term changes over time. GaitSmart® is considered of additive value because it is easy to use (contrary to analysis in an optic gait lab), gives information on subjective and objective function (contrary to questionnaires), is sensitive for short term change (contrary to PBT), and gives information on quality of gait.

As expected, the adjusted R^2^ is higher for the objective function domain compared to the subjective function domain. Not surprisingly. GaitSmart®, as objective measurement, did not represent a high proportion of the variability in the subjective function domain (R^2^ for subjective function domain is low). This finding is confirmed by longitudinal analyses. Changes in GaitSmart® based function scores are specifically related to changes in PBT and less to changes in function questionnaires. Nevertheless, the adjusted R^2^ is highest in the model for the total function domain. This indicates that both objective function and subjective function contribute to the total function domain. As such, GaitSmart® is of use as function outcome measure, combining evaluation of both constructs of function.

There is a notable difference between the models for objective and subjective function. In the model for subjective function, GaitSmart® parameters that are related to the index leg are statistically significant, this in contrast to objective function where differences between both legs were found to be more dominant. The index leg is the leg which was most painful for participants at screening, indicating pain is more important for subjective function compared to objective function. This is confirmed by Terwee et al., who found better correlations between pain and self-reported function than between pain and performance-based measures [11]. Moreover, change in pain was found to be the principal determinant of change in self-reported function [9].

Prediction models using GaitSmart® domains as independent variables show the same trend. In the model for subjective function, ‘GS Difference stance’ is included. Most likely, stance phase of a stride is more painful than swing phase, because in that phase, loading is applied. Therefore stance phase, and with that ‘GS Difference stance’, might be more important for a person’s view of their function (subjective), compared to their actual function (objective). These findings support the result of the PCA: subjective function is mainly determined by questionnaires, a reflection of people’s opinion about their function.

In the model for objective function, hip related GS domains are included, suggesting a contribution of the hip joint in someone’s objective function, which apparently is less pain related but more related to actual performance.

Not surprisingly, in the analysis where subgroups were based on the first question of the SF-36, highest effect sizes were found for both SF-36 subscales. This question is not part of the SF-36 subscales, but is included in the general health subscale. General health is related to both physical and mental health [26], and it remains uncertain if and how function and general health are influenced by each other. Nevertheless, effect sizes were high (>0.8) for all parameters, including GaitSmart® based function scores, indicating that all function outcome measures are able to differentiate between participants with different general health status.

In case of dichotomisation based on PBT, GaitSmart® based function scores show the highest effect sizes for a six month change. Interestingly, GS subjective function score, also shows higher effect sizes for a six month change in the objective PBT. In case of dichotomization based on function questionnaires effect sizes were significantly smaller. This implies that although GaitSmart^®^ includes both subjective and objective function, it best describes objective function.

With respect to the IMI-APPROACH cohort it appeared that GaitSmart® showed the highest SRM for a six month change in the low progression group. Assuming these participants will indeed slowly progress, this shows that GaitSmart® is able to detect small changes in function. Of course final follow-up data is needed to further evaluate usability of GaitSmart® to detect disease progression in different knee OA subgroups.

No exercise programs (or other interventions) were prescribed during the study, but concomitant OA treatments, like rehabilitation programs, were allowed. These programs might improve physical function. However, this most likely would affect both functional outcome measures as well was GaitSmart® parameters and as such would not bias the concurrent association between measures. Besides, the ability of GaitSmart® to detect short term changes in physical function was evaluated, and the reason for this change in physical function (e.g. natural disease course or improvement after rehabilitation program) is not expected to affect this.

The main limitation of the study is that no difference is made between patients with unilateral or bilateral OA. Although gait is a characteristic of an individual rather than of a specific joint, in future studies GaitSmart® should be evaluated in specific subgroups of OA (e.g. different Kellgren and Lawrence grades, unilateral *vs* bilateral, with *vs* without concomitant OA in other joints). Nevertheless, the results of this subanalysis of the IMI-APPROACH cohort study provide a first indication of the additional value of GaitSmart® motion analysis in the assessment of physical function in OA patients.

In conclusion, this study shows that GaitSmart® is related to commonly used function outcome measures and includes evaluation of subjective and objective function with a dominance on objective function. GaitSmart® is responsive to changes in different aspects of objective function. Future studies using GaitSmart® are warranted to validate whether GaitSmart® can be used as clinical outcome measure in research and clinical practice.

## Patient and public involvement

A Patient Council (PC) was instituted to represent the patient’s perspective in the APPROACH consortium. The PC contributed to design of the clinical study and helped to shape the project with particular consideration for the interests of study participants.

## Supporting information

S1 Checklist TREND statement checklist. (PDF)

S1 File Principal component analysis individual GaitSmart® analysis. (PDF)

S2 File Linear regression analyses using GaitSmart® domains. (PDF)

S3 File Standardized response means, effect sizes and statistical significance by t-tests between those with an in- or decrease by at least the MDC of the KOOS or SF-36 subscales. (PDF)

S4 File Standardized response means, effect sizes and statistical significance by t-tests between those with low or high predicted progression. (PDF)

S5 File Photos of used techniques. (PDF)

S6 File Minimal data set. (PDF)
