# Supplementary material for: GaitSmart motion analysis compared to commonly used function outcome measures in the IMI-APPROACH knee osteoarthritis cohort
Source: PLoS One. 2022 Mar 23;17(3):e0265883. doi: 10.1371/journal.pone.0265883 (PMC8942249; doi:10.1371/journal.pone.0265883)
Supplement: S1 File — (PDF) [file pone.0265883.s002.pdf]

Supplementary file 1: Principal component analysis individual GaitSmart® analysis

Previously, a principal component analysis (PCA) was performed on individual GaitSmart® parameters to explore structure in relationships between GaitSmart® parameters and to reduce the total set of parameters to a limited set of underlying domains(1).

Table S1. Principal component analysis GaitSmart® parameters

		Component				
**Domain**	**Parameter**	**1**	**2**	**3**	**4**	**5**
GS Knee	ROM index knee in swing	0.134	**0.891**	-0.111	-0.034	-0.058
	ROM contralateral knee in swing	0.164	**0.876**	0.062	-0.046	-0.026
	ROM index calf	0.491	**0.764**	-0.163	-0.011	0.047
	ROM contralateral calf	0.526	**0.745**	-0.019	0.021	0.068
	ROM index knee in stance	0.534	**0.478**	-0.007	-0.132	-0.368
	ROM contralateral knee in stance	0.613	**0.346**	-0.057	-0.121	-0.088
GS Hip	ROM index hip	**0.849**	0.208	-0.065	0.080	0.059
	ROM contralateral hip	**0.841**	0.184	0.025	0.164	0.065
	Speed (m/s)	**0.919**	0.207	-0.048	-0.175	-0.028
	Average duration per stride (s)	**-0.565**	0.059	0.014	0.513	0.186
	Stride length (m)	**0.761**	0.281	-0.046	0.087	0.101
GS Difference knee	Difference ROM knees in swing	-0.054	-0.105	**0.803**	0.189	-0.067
	Difference ROM calves	-0.043	-0.005	**0.847**	0.189	-0.067
GS Difference stance	Difference ROM knees in stance	0.069	-0.012	0.126	-0.033	**0.912**
GS Difference hip	Difference ROM hip	0.099	-0.091	0.060	**0.836**	-0.071

GS: GaitSmart™, ROM: range of motion
