# Supplementary material for: GaitSmart motion analysis compared to commonly used function outcome measures in the IMI-APPROACH knee osteoarthritis cohort
Source: PLoS One. 2022 Mar 23;17(3):e0265883. doi: 10.1371/journal.pone.0265883 (PMC8942249; doi:10.1371/journal.pone.0265883)
Supplement: S2 File — (PDF) [file pone.0265883.s003.pdf]

Supplementary file 2: Linear regression analyses using GaitSmart® domains

In addition to the individual GaitSmart® parameters, the five underlying domains identified: GS Knee, GS Hip, GS Difference knee, GS Difference stance, and GS Difference hip, were also used for linear regression analyses.

In the regression models with GaitSmart® domains as independent variables, the following interaction terms were also added to explore synergistic effects:

Interaction 1: GS Knee*GS Hip

Interaction 2: GS Hip*GS Difference hip

Interaction 3: GS Knee*GS Difference knee

Modelling started with a 'full model' including all (possible) predictors and interaction terms. Then all predictors with a p-value >0.2 were removed from the model, starting with the least significant variable. In case the adjusted R^2 diminished relevantly, the variable was retained in the prediction model. If interactions were relevant predictors, the individual parameters of these interactions were retained as well.

Table S2. Linear regression analysis for subjective function (adjusted R^2 0.121)

Independent variable	B (unstand.)	95%CI		Beta (stand.)	p-value
Constant	.010	-.101	.121		.863
GS Hip	.228	.117	.338	.230	.000
GS Knee	.265	.154	.375	.268	.000
GS Difference stance	-.086	-.202	.030	.083	.147

GS: GaitSmart®

Table S3. Linear regression analysis for objective function (adjusted R^2 0.227)

Independent variable	B (unstand.)	95%CI		Beta (stand.)	p-value
Constant	.026	-.075	.126		.616
GS Hip	.411	.311	.512	.431	.000
GS Knee	.221	.121	.321	.231	.000
GS Difference hip	-.048	-.149	.052	-.051	.343
GS Hip*GS Difference hip	-.085	-.183	.012	-.093	.087

GS: GaitSmart®

Table S4. Linear regression analysis for total function (adjusted R^2 0.316)

Independent variable	B (unstand.)	95%CI		Beta (stand.)	p-value
Constant	.024	-.074	.121		.630
GS Hip	.444	346	.541	.452	.000
GS Knee	.345	.248	.442	.351	.000
GS Difference stance	-.072	-.175	.031	-.070	.169
GS Difference hip	-.057	-.154	.040	-.058	.248
GS Hip*GS Difference hip	-.080	-.175	.016	-.084	.101

GS: GaitSmart®
