# Supplementary material for: GaitSmart motion analysis compared to commonly used function outcome measures in the IMI-APPROACH knee osteoarthritis cohort
Source: PLoS One. 2022 Mar 23;17(3):e0265883. doi: 10.1371/journal.pone.0265883 (PMC8942249; doi:10.1371/journal.pone.0265883)
Supplement: S3 File — (PDF) [file pone.0265883.s004.pdf]

Supplementary file 3: Standardized response means, effect sizes and statistical significance by t-tests between those with an in- or decrease by at least the MDC of the KOOS or SF-36 subscales.

Table S5. Standardized response means and effect sizes of different functional outcome measures in subgroups based on MDC (=15.7) of the KOOS daily function subscale

	Worsened KOOS daily function n=28		Improved KOOS daily function n=30		Effect size	T-test
	M6-BL mean (SD)	SRM	M6-BL mean (SD)	SRM	Hedges' *g*	p-value
Chair (# standing up)	-0.11 (1.99)	-0.06	0.00 (3.27)	0.00	0.04	0.882
Walk (s)	-0.18 (4.30)	-0.04	-2.10 (6.75)	-0.31	0.34	0.205
KOOS sports	-18.75 (25.73)	-0.73	15.33 (18.00)	**0.85**	1.54	**<0.001**
SF-36 physical function	-16.25 (16.59)	**-0.98**	7.67 (19.86)	0.39	1.30	**<0.001**
SF-36 role physical	-13.62 (27.33)	-0.50	6.04 (33.42)	0.18	0.64	**0.018**
GS total function	-0.13 (0.47)	-0.27	0.02 (0.42)	0.04	0.32	0.228
GS objective function	0.01 (0.36)	0.03	0.07 (0.36)	0.18	0.15	0.569
GS subjective function	-0.06 (0.28)	-0.21	0.04 (0.33)	0.13	0.33	0.216

SRM: Standardized response mean, M6: 6 months follow-up visit, BL: Baseline, KOOS: Knee injury and Osteoarthritis Outcome Score, SF-36: Short Form 36 Health Survey, GS: GaitSmart®; highest SRM for each in bold; p-values <0.05 in bold.

Table S6. Standardized response means and effect sizes of different functional outcome measures in subgroups based on MDC (=25.1) of the KOOS sports

	Worsened KOOS sports n=19		Improved KOOS sports n=14		Effect size	T-test
	M6-BL mean (SD)	SRM	M6-BL mean (SD)	SRM	Hedges' *g*	p-value
Chair (# standing up)	0.53 (2.07)	0.26	-0.21 (3.66)	-0.06	0.26	0.466
Walk (s)	-1.42 (5.56)	-0.26	1.86 (5.93)	0.31	0.57	0.114
KOOS daily function	-11.54 (11.92)	**-0.96**	15.33 (18.25)	**0.84**	1.80	**<0.001**
SF-36 physical function	-6.58 (12.70)	-0.52	10.00 (23.62)	0.42	0.92	**0.014**
SF-36 role physical	6.25 (19.43)	0.32	5.80 (25.41)	0.22	0.02	0.955
GS total function	-0.12 (0.46)	-0.25	0.00 (0.76)	0.01	0.20	0.570
GS objective function	0.04 (0.27)	0.15	0.09 (0.63)	0.14	0.11	0.765
GS subjective function	-0.02 (0.33)	-0.06	0.05 (0.47)	0.11	0.17	0.627

SRM: Standardized response mean, M6: 6 months follow-up visit, BL: Baseline, KOOS: Knee injury and Osteoarthritis Outcome Score, SF-36: Short Form 36 Health Survey, GS: GaitSmart®; highest SRM for each in bold; p-values <0.05 in bold.

Table S7. Standardized response means and effect sizes of different functional outcome measures in subgroups based on MDC (=-2.46 for worsening, and 2.26 for improvement) of the SF-36 physical function subscale

	Worsened SF-36 physical function n=103		Improved SF-36 physical function n=99		Effect size	T-test
	M6-BL mean (SD)	SRM	M6-BL mean (SD)	SRM	Hedges' *g*	p-value
Chair (# standing up)	0.58 (2.16)	0.27	0.44 (2.84)	0.16	0.06	0.697
Walk (s)	-0.27 (5.77)	-0.05	-0.87 (4.45)	-0.16	0.11	0.597
KOOS daily function	-4.17 (14.60)	-0.29	5.55 (13.49)	**0.41**	0.69	**<0.001**
KOOS sports	-8.01 (22.19)	**-0.36**	2.78 (20.01)	0.14	0.51	**<0.001**
SF-36 role physical	-8.43 (23.30)	**-0.36**	7.20 (20.93)	0.34	1.06	**<0.001**
GS total function	-0.09 (0.47)	-0.18	-0.01 (0.50)	-0.02	0.15	0.277
GS objective function	0.04 (0.37)	0.10	0.10 (0.43)	0.23	0.15	0.274
GS subjective function	-0.04 (0.32)	-0.11	0.03 (0.31)	0.10	0.21	0.129

SRM: Standardized response mean, M6: 6 months follow-up visit, BL: Baseline, KOOS: Knee injury and Osteoarthritis Outcome Score, SF-36: Short Form 36 Health Survey, GS: GaitSmart®; highest SRM for each in bold; p-values <0.05 in bold.

Table S8. Standardized response means and effect sizes of different functional outcome measures in subgroups based on MDC (=-9.61 for worsening, and 10.85 for improvement) of the SF-36 role physical

	Worsened SF-36 role physical n=79		Improved SF-36 role physical n=76		Effect size	T-test
	M6-BL mean (SD)	SRM	M6-BL mean (SD)	SRM	Hedges' *g*	p-value
Chair (# standing up)	0.23 (2.20)	0.10	0.55 (3.09)	0.18	0.12	0.451
Walk (s)	0.15 (5.92)	0.03	-1.17 (4.39)	-0.27	0.25	0.117
KOOS daily function	-3.33 (15.49)	-0.22	5.47 (15.16)	0.36	0.57	**<0.001**
KOOS sports	-6.27 (16.99)	-0.37	0.86 (24.13)	0.04	0.34	**0.036**
SF-36 physical function	-7.97 (16.44)	**-0.49**	7.04 (15.75)	**0.45**	0.93	**<0001**
GS total function	-0.10 (0.54)	-0.19	0.02 (0.47)	0.05	0.25	0.127
GS objective function	0.04 (0.41)	0.09	0.12 (0.41)	0.31	0.21	0.192
GS subjective function	-0.04 (0.32)	-0.12	0.06 (0.31)	0.18	0.30	0.068

SRM: Standardized response mean, M6: 6 months follow-up visit, BL: Baseline, KOOS: Knee injury and Osteoarthritis Outcome Score, SF-36: Short Form 36 Health Survey, GS: GaitSmart®; highest SRM for each in bold; p-values <0.05 in bold.
