# Supplementary material for: GaitSmart motion analysis compared to commonly used function outcome measures in the IMI-APPROACH knee osteoarthritis cohort
Source: PLoS One. 2022 Mar 23;17(3):e0265883. doi: 10.1371/journal.pone.0265883 (PMC8942249; doi:10.1371/journal.pone.0265883)
Supplement: S4 File — (PDF) [file pone.0265883.s005.pdf]

Supplementary file 4: Standardized response means, effect sizes and statistical significance by t-tests between those with low or high predicted progression.

Table S9. Standardized response means and effect sizes of different functional outcome measures in subgroups based on predicted progression probabilities

	Low predicted progression n=33		High predicted progression n=38		Effect size	T-test
	M6-BL mean (SD)	SRM	M6-BL mean (SD)	SRM	Hedges' *g*	p-value
Chair (# standing up)	0.45 (1.62)	0.28	0.39 (2.41)	0.16	0.03	0.904
Walk (s)	0.97 (3.30)	0.29	-2.11 (6.30)	**-0.33**	0.60	**0.014**
KOOS daily function	-1.29 (11.38)	-0.11	4.22 (16.88)	0.25	0.38	0.109
KOOS sports	-1.82 (21.17)	-0.09	1.84 (25.21)	0.07	0.16	0.514
SF-36 physical function	-1.06 (10.29)	-0.10	1.97 (20.97)	0.09	0.18	0.433
SF-36 role physical	-4.73 (17.54)	-0.27	1.15 (27.49)	0.04	0.25	0.280
GS total function	-0.13 (0.44)	**-0.30**	0.10 (0.47)	0.22	0.52	**0.033**
GS objective function	0.05 (0.30)	0.16	0.13 (0.43)	0.29	0.20	0.398
GS subjective function	-0.07 (0.29)	-0.25	0.05 (0.27)	0.18	0.43	0.077

SRM: Standardized response mean, M6: 6 months follow-up visit, BL: Baseline, KOOS: Knee injury and Osteoarthritis Outcome Score, SF-36: Short Form 36 Health Survey, GS: GaitSmart®; highest SRM for each in bold; p-values <0.05 in bold.
