# Supplementary material for: GaitSmart motion analysis compared to commonly used function outcome measures in the IMI-APPROACH knee osteoarthritis cohort
Source: PLoS One. 2022 Mar 23;17(3):e0265883. doi: 10.1371/journal.pone.0265883 (PMC8942249; doi:10.1371/journal.pone.0265883)

Supplementary file 5: Photos of used techniques

Figure S1: Position of IMUs

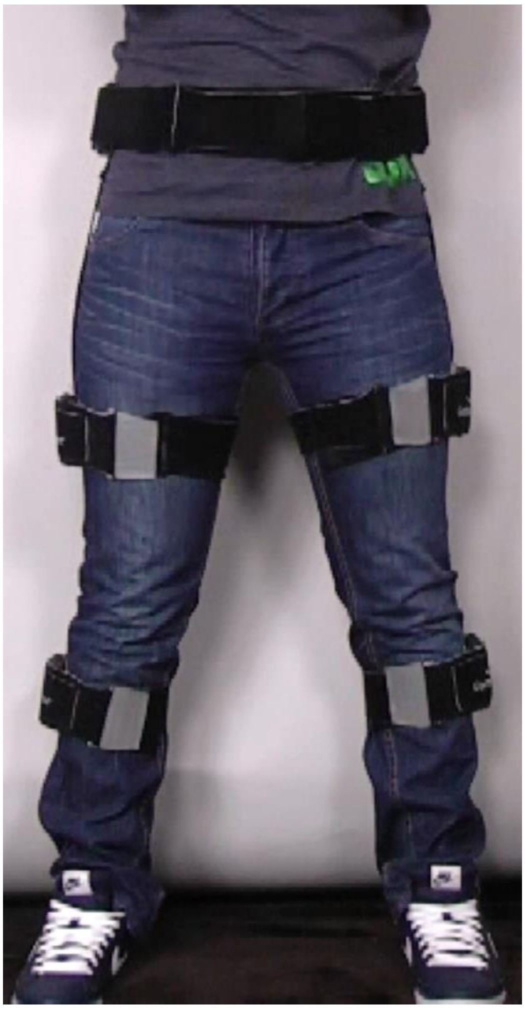

Figure S2: Software to prepare GaitSmart® report

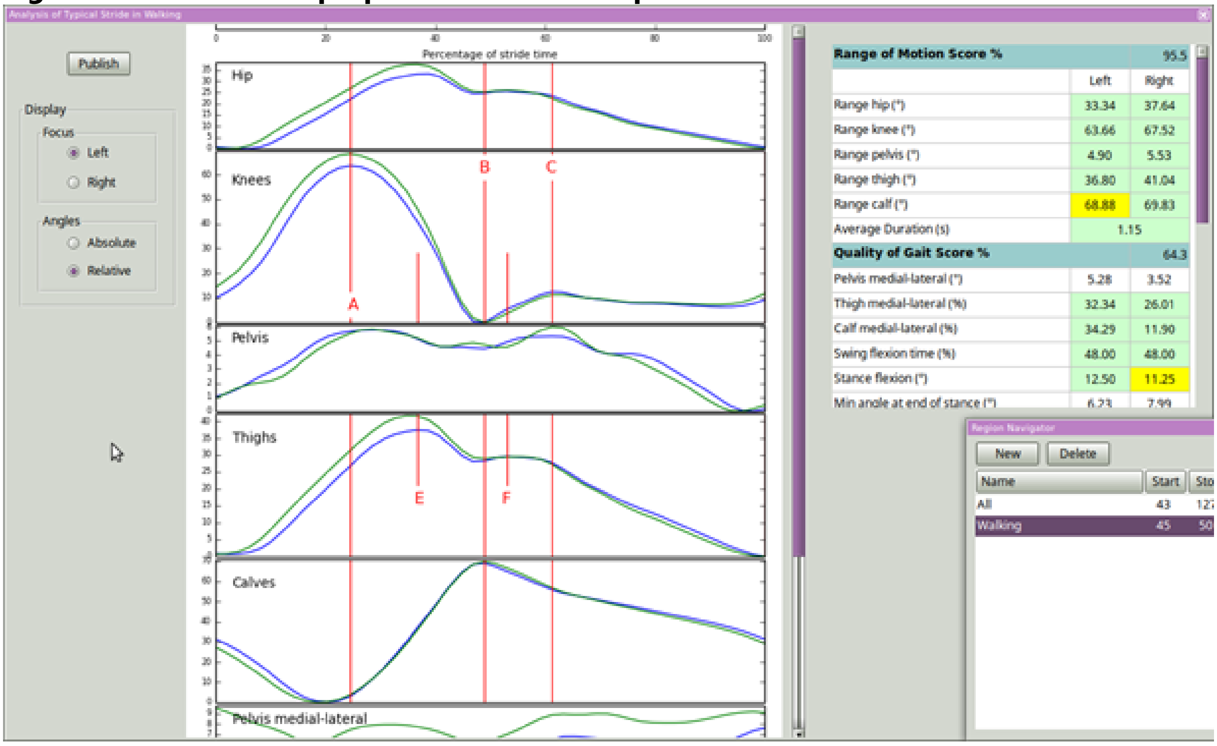

Supplement: S5 File — (PDF) [file pone.0265883.s006.pdf]
