# Supplementary material for: GaitSmart motion analysis compared to commonly used function outcome measures in the IMI-APPROACH knee osteoarthritis cohort
Source: PLoS One. 2022 Mar 23;17(3):e0265883. doi: 10.1371/journal.pone.0265883 (PMC8942249; doi:10.1371/journal.pone.0265883)
Supplement: S6 File — (PDF) [file pone.0265883.s007.pdf]

Subject	Age	Gender	BMI	Indexknee	P	S	GroupPS
1250180110359	63	Female	47,5	1	0,30	0,44	0
1250180110361	75	Female	34,5	1	0,37	0,36	0
1250180110362	72	Female	25,7	1	0,15	0,42	0
1250180110364	67	Female	29,6	2	0,28	0,35	0
1250180110367	75	Female	21,1	1	0,20	0,41	0
1250180110368	77	Female	24,7	1	0,44	0,34	0
1250180110369	71	Female	27,5	1	0,77	0,55	2
1250180110371	71	Female	18,9	1	0,83	0,47	1
1250180110402	72	Female	32,5	1	0,51	0,39	1
1250180110416	77	Female	#LEEG!	2	0,79	0,30	1
1250180110417	69	Female	26,0	1	0,51	0,41	1
1250180110418	58	Female	29,3	2	0,19	0,60	1
1250180110421	72	Female	25,1	1	0,47	0,41	0
1250180110422	70	Female	29,9	1	0,79	0,31	1
1250180110423	62	Female	27,0	1	0,19	0,48	0
1250180110424	46	Female	32,1	1	0,53	0,59	2
1250180110430	57	Female	34,1	1	0,69	0,36	1
1250180110432	58	Female	31,1	1	0,84	0,37	1
1250180110433	51	Female	31,8	2	0,62	0,43	1
1250180110434	73	Female	28,5	1	0,82	0,30	1
1528020110001	73	Female	32,5	2	0,41	0,35	0
1528020110002	71	Female	23,3	1	0,60	0,24	1
1528020110004	73	Male	29,6	2	0,65	0,42	1
1528020110005	69	Male	29,1	2	0,42	0,30	0
1528020110007	62	Female	26,5	1	0,77	0,36	1
1528020110011	73	Female	27,9	1	0,73	0,38	1
1528020110012	71	Female	28,0	1	0,41	0,46	0
1528020110014	68	Female	22,3	2	0,22	0,40	0
1528020110015	66	Female	27,6	2	0,41	0,34	0
1528020110016	62	Female	26,5	2	0,37	0,41	0
1528020110017	70	Male	24,8	2	0,10	0,59	1
1528020110018	62	Female	24,1	2	0,16	0,44	0
1528020110019	70	Male	27,5	2	0,17	0,56	1
1528020110020	69	Male	24,4	2	0,24	0,42	0
1528020110022	77	Female	25,3	2	0,82	0,48	1
1528020110023	68	Female	28,2	2	0,68	0,35	1
1528020110025	68	Male	22,8	2	0,18	0,40	0
1528020110027	72	Female	23,5	1	0,32	0,37	0
1528020110028	79	Female	29,2	2	0,25	0,35	0
1528020110029	62	Male	24,2	1	0,18	0,53	1
1528020110030	71	Female	27,0	1	0,27	0,38	0
1528020110044	62	Female	34,2	2	0,24	0,47	0
1528020110045	65	Male	30,3	2	0,48	0,32	0
1528020110047	78	Female	26,4	2	0,14	0,39	0
1528020110049	74	Female	34,8	2	0,27	0,44	0
1528020110051	63	Female	26,4	2	0,38	0,57	1
1528020110053	77	Female	23,7	1	0,49	0,35	0
1528020110066	64	Female	25,3	2	0,16	0,39	0
1528020110068	62	Female	27,7	1	0,41	0,42	0

1528020110070	72	Female	25,7	1	0,52	0,49	1
1528020110096	76	Female	21,7	2	0,20	0,42	0
1528020110108	68	Female	28,0	1	0,13	0,40	0
1528020110122	62	Male	24,0	2	0,52	0,46	1
1528020110123	62	Male	24,2	1	0,64	0,35	1
1528020110124	69	Female	21,4	2	0,22	0,41	0
1528020110125	73	Female	28,8	1	0,23	0,41	0
1528020110126	61	Female	32,5	1	0,82	0,38	1
1528020110127	68	Male	32,7	1	0,48	0,32	0
1528020110130	73	Female	27,9	2	0,50	0,45	0
1528020110132	69	Female	20,1	2	0,42	0,37	0
1528020110135	71	Female	30,1	1	0,23	0,36	0
1528020110139	62	Female	31,5	1	0,28	0,46	0
1528020110140	79	Male	25,9	1	0,17	0,57	1
1528020110141	69	Male	24,9	1	0,20	0,43	0
1528020110148	68	Female	35,0	1	0,20	0,38	0
1528020110150	68	Female	32,3	1	0,74	0,36	1
1528020110152	72	Male	20,6	2	0,16	0,41	0
1528020110154	67	Male	25,0	1	0,20	0,39	0
1528020110156	63	Female	42,3	2	0,26	0,48	0
1528020110161	68	Male	28,1	2	0,18	0,50	0
1528020110163	62	Female	21,1	2	0,37	0,61	1
1528020110165	63	Female	25,8	2	0,43	0,38	0
1528020110169	66	Female	27,1	1	0,12	0,50	0
1528020110170	64	Female	27,1	2	0,45	0,37	0
1528020110171	67	Female	27,4	2	0,52	0,31	1
1528020110172	80	Female	29,4	1	0,28	0,45	0
1528020110176	75	Female	25,8	1	0,24	0,39	0
1528020110177	66	Female	23,9	1	0,38	0,39	0
1528020110179	77	Female	26,6	2	0,56	0,43	1
1528020110181	69	Female	20,2	2	0,44	0,35	0
1528020110182	72	Female	22,0	2	0,15	0,51	1
1528020110184	66	Female	28,6	1	0,81	0,40	1
1528020110188	75	Male	25,7	1	0,29	0,47	0
1528020110190	69	Female	27,9	2	0,56	0,32	1
1528020110191	68	Female	33,6	1	0,66	0,39	1
1528020110193	76	Female	23,4	1	0,15	0,42	0
1528020110196	68	Female	23,0	1	0,43	0,36	0
1528020110197	70	Male	30,1	2	0,71	0,29	1
1528020110200	67	Female	32,3	2	0,16	0,39	0
1528020110201	67	Female	32,6	1	0,24	0,50	0
1528020110202	69	Female	26,4	1	0,21	0,37	0
1528020110219	72	Female	24,3	1	0,79	0,36	1
1528020110221	62	Female	23,2	2	0,24	0,55	0
1528020110222	78	Female	24,2	2	0,26	0,36	0
1528020110223	72	Female	21,1	1	0,19	0,43	0
1528020110231	59	Female	31,2	1	0,21	0,40	0
1528020110232	69	Male	25,3	1	0,15	0,41	0
1528020110233	67	Male	21,7	1	0,58	0,36	1
1528020110235	75	Male	26,0	1	0,13	0,39	0

1528020110236	60	Female	23,4	2	0,16	0,46	0
1528020110237	75	Female	24,4	2	0,78	0,32	1
1528020110238	63	Female	20,9	2	0,68	0,38	1
1528020110256	65	Female	26,6	2	0,18	0,47	0
1528020110257	73	Female	32,3	2	0,15	0,42	0
1528020110258	70	Male	18,3	1	0,15	0,40	0
1528020110260	72	Female	26,2	1	0,19	0,37	0
1528020110261	75	Female	23,0	1	0,41	0,32	0
1528020110262	70	Female	22,5	1	0,13	0,55	1
1528020110266	74	Female	20,9	2	0,15	0,38	0
1528020110267	72	Male	23,0	2	0,21	0,41	0
1528020110269	67	Male	32,7	1	0,58	0,33	1
1528020110272	71	Female	21,6	1	0,18	0,43	0
1528020110273	70	Male	32,9	2	0,20	0,36	0
1528020110274	62	Female	27,0	2	0,15	0,45	0
1528020110279	76	Male	31,8	2	0,12	0,38	0
1528020110283	67	Female	31,6	2	0,21	0,36	0
1528020110284	70	Female	22,9	1	0,21	0,35	0
1528020110286	66	Female	27,7	2	0,16	0,37	0
1528020110288	65	Female	26,3	1	0,12	0,56	1
1528020110291	79	Female	27,5	1	0,28	0,37	0
1528020110292	65	Female	20,9	1	0,19	0,46	0
1528020110296	63	Male	31,5	2	0,65	0,48	1
1528020110298	60	Male	22,0	1	0,11	0,43	0
1528020110299	72	Female	22,1	1	0,24	0,37	0
1528020110301	72	Female	26,7	1	0,71	0,39	1
1528020110308	64	Female	34,6	2	0,81	0,38	1
1528020110309	68	Female	25,0	1	0,82	0,33	1
1528020110310	67	Female	30,9	2	0,18	0,37	0
1528020110312	65	Female	33,6	1	0,73	0,32	1
1528020110314	60	Female	37,7	1	0,44	0,52	1
1528020110315	68	Female	26,7	2	0,15	0,47	0
1528020110318	61	Female	32,6	1	0,71	0,42	1
1528020110320	64	Female	27,6	2	0,44	0,37	0
1528020110326	69	Female	27,9	1	0,28	0,40	0
1528020110327	67	Female	27,2	1	0,43	0,46	0
1528020110330	72	Female	36,6	1	0,74	0,29	1
1528020110331	68	Female	18,6	2	0,28	0,34	0
1528020110332	64	Female	25,3	2	0,15	0,39	0
1528020110333	64	Male	27,8	1	0,13	0,62	1
1528020110334	64	Male	30,2	2	0,11	0,55	1
1528020110335	67	Female	26,2	2	0,28	0,35	0
1528020110336	68	Female	27,2	2	0,24	0,35	0
1528020110358	68	Female	25,9	1	0,34	0,34	0
1528020110363	69	Female	26,3	1	0,12	0,51	1
1528020110373	62	Male	42,3	1	0,50	0,33	0
1528020110374	47	Male	21,5	2	0,46	0,62	1
1528020110375	57	Male	26,4	1	0,34	0,36	0
1528020110376	58	Female	26,6	1	0,60	0,40	1
1528020110377	76	Male	24,8	2	0,30	0,40	0

1528020110378	66	Female	27,6	1	0,15	0,55	1
1528020110379	69	Male	27,1	1	0,22	0,36	0
1528020110380	65	Female	22,4	2	0,19	0,37	0
1528020110382	78	Male	28,7	1	0,60	0,39	1
1528020110384	59	Female	41,5	2	0,58	0,53	2
1528020110385	58	Female	32,0	1	0,55	0,53	2
1528020110388	61	Female	23,5	1	0,20	0,38	0
1528020110389	70	Female	24,5	2	0,21	0,44	0
1528020110390	69	Male	25,6	2	0,19	0,47	0
1528020110392	59	Male	28,7	2	0,23	0,35	0
1528020110395	51	Male	25,5	2	0,67	0,27	1
1528020110397	72	Female	30,8	2	0,31	0,39	0
1528020110399	67	Female	28,2	1	0,70	0,58	2
1528020110400	65	Female	23,9	2	0,25	0,40	0
1528020110403	58	Male	25,5	1	0,36	0,46	0
1528020110405	44	Male	30,2	1	0,40	0,45	0
1528020110406	67	Female	22,7	2	0,35	0,39	0
1528020110407	77	Male	26,0	2	0,28	0,41	0
1528020110408	50	Female	20,8	1	0,29	0,45	0
1528020110409	66	Male	26,3	2	0,28	0,36	0
1528020110410	71	Female	32,0	1	0,50	0,33	0
1528020110415	51	Male	35,9	1	0,22	0,35	0
1528020110420	48	Female	29,8	1	0,55	0,24	1
1528020110426	82	Female	27,8	2	0,22	0,50	0
1528140110099	63	Female	24,0	1	0,83	0,33	1
1528140110100	61	Female	25,1	2	0,17	0,52	1
1528140110111	74	Female	36,8	1	0,51	0,37	1
1528140110120	67	Female	23,2	1	0,19	0,54	1
1528140110121	52	Female	35,6	1	0,69	0,39	1
1528140110142	67	Female	25,4	1	0,60	0,36	1
1528140110143	71	Male	26,1	2	0,54	0,26	1
1528140110144	65	Female	23,1	1	0,15	0,40	0
1528140110145	60	Male	25,3	1	0,41	0,60	1
1528140110146	70	Female	19,8	1	0,15	0,46	0
1528140110151	69	Male	23,1	2	0,42	0,36	0
1528140110153	66	Female	21,7	1	0,26	0,38	0
1528140110157	58	Female	35,2	1	0,77	0,36	1
1528140110159	68	Male	26,9	1	0,42	0,33	0
1528140110204	70	Female	26,6	2	0,55	0,30	1
1528140110224	74	Female	22,9	2	0,22	0,50	0
1528140110225	71	Female	26,0	1	0,47	0,43	0
1528140110226	64	Male	36,0	1	0,73	0,47	1
1528140110229	63	Female	29,6	1	0,16	0,46	0
1528140110239	72	Female	30,0	2	0,83	0,33	1
1528140110240	74	Male	41,5	1	0,32	0,35	0
1528140110241	60	Female	23,2	1	0,23	0,47	0
1528140110242	61	Female	25,9	2	0,28	0,39	0
1528140110243	66	Female	31,9	2	0,36	0,35	0
1528140110244	72	Female	25,8	2	0,35	0,39	0
1528140110245	53	Female	24,5	2	0,29	0,46	0

1528140110246	55	Female	23,0	1	0,31	0,51	1
1528140110251	62	Female	26,0	1	0,25	0,41	0
1528140110271	59	Male	34,3	2	0,61	0,30	1
1528140110277	48	Female	23,3	2	0,19	0,44	0
1528140110278	74	Female	24,7	1	0,48	0,35	0
1528140110280	63	Female	29,8	1	0,82	0,35	1
1528140110293	68	Male	32,0	1	0,50	0,41	0
1528140110294	59	Female	19,5	1	0,23	0,39	0
1528140110302	64	Male	33,0	1	0,37	0,41	0
1528140110306	66	Female	33,7	1	0,47	0,45	0
1528140110307	61	Female	23,2	1	0,15	0,48	0
1528140110322	63	Female	28,6	2	0,17	0,49	0
1528140110329	78	Female	25,5	1	0,28	0,47	0
1528140110337	75	Female	19,7	2	0,19	0,43	0
1528140110339	72	Female	24,2	2	0,26	0,39	0
1528140110340	63	Female	22,9	1	0,16	0,44	0
1528140110341	72	Female	25,0	2	0,39	0,37	0
1528140110343	76	Female	23,6	1	0,22	0,37	0
1528140110360	56	Female	27,5	1	0,74	0,23	1
1528140110370	69	Female	38,9	2	0,81	0,49	1
1528140110387	66	Female	27,1	2	0,26	0,40	0
1528140110393	53	Male	32,1	2	0,73	0,55	2
1528140110401	59	Male	31,0	2	0,38	0,61	1
1528140110414	56	Female	26,5	2	0,22	0,37	0
1578190110031	73	Female	32,0	1	0,80	0,26	1
1578190110034	58	Male	37,3	2	0,24	0,42	0
1578190110055	70	Female	26,0	1	0,79	0,33	1
1578190110056	74	Male	29,4	2	0,47	0,35	0
1578190110076	72	Female	27,3	1	0,35	0,48	0
1578190110078	70	Female	27,4	2	0,37	0,41	0
1578190110081	71	Female	30,2	1	0,51	0,43	1
1578190110083	71	Female	20,6	2	0,56	0,36	1
1578190110101	68	Female	25,8	1	0,59	0,30	1
1578190110103	81	Female	26,4	1	0,29	0,41	0
1578190110129	51	Female	26,8	1	0,67	0,36	1
1578190110131	68	Female	22,9	1	0,69	0,33	1
1578190110136	62	Female	41,3	1	0,69	0,55	1
1578190110206	59	Male	31,9	2	0,37	0,35	0
1578190110210	71	Male	36,5	2	0,35	0,37	0
1578190110214	54	Female	25,5	2	0,22	0,47	0
1578190110215	72	Male	23,2	1	0,23	0,46	0
1578190110216	50	Female	31,9	2	0,63	0,38	1
1578190110217	70	Female	24,4	2	0,72	0,29	1
1578190110248	60	Female	35,8	2	0,68	0,33	1
1578190110250	70	Male	20,4	1	0,66	0,34	1
1578190110349	62	Female	43,8	2	0,59	0,40	1
1578190110351	61	Female	22,5	1	0,66	0,38	1
1578190110352	52	Female	24,8	2	0,28	0,44	0
1578190110353	71	Female	23,3	1	0,49	0,35	0
1578190110354	46	Male	33,5	2	0,49	0,37	0

1578190110355	52	Female	24,7	1	0,54	0,33	1
1578190110356	75	Female	22,5	2	0,62	0,31	1
1578190110411	57	Male	34,8	1	0,79	0,42	1
1578190110412	61	Female	19,0	1	0,26	0,43	0
1578190110413	71	Female	36,8	2	0,59	0,37	1
1724170110033	51	Female	36,3	1	0,74	0,33	1
1724170110035	62	Male	33,5	1	0,51	0,44	1
1724170110036	77	Female	34,9	2	0,80	0,30	1
1724170110038	68	Female	38,5	1	0,54	0,40	1
1724170110039	64	Female	20,4	1	0,68	0,37	1
1724170110041	59	Female	19,3	1	0,78	0,25	1
1724170110046	60	Male	26,6	1	0,40	0,38	0
1724170110048	68	Female	35,3	1	0,49	0,33	0
1724170110054	58	Female	35,0	1	0,40	0,53	1
1724170110057	62	Female	34,2	1	0,71	0,37	1
1724170110058	64	Female	37,4	2	0,78	0,38	1
1724170110059	64	Female	26,3	1	0,71	0,43	1
1724170110061	55	Female	30,2	1	0,77	0,30	1
1724170110063	78	Female	34,3	1	0,64	0,29	1
1724170110065	62	Female	44,0	1	0,58	0,26	1
1724170110067	59	Female	25,0	1	0,30	0,52	1
1724170110074	67	Female	31,6	1	0,65	0,56	2
1724170110084	77	Female	39,1	2	0,74	0,43	1
1724170110085	81	Male	38,0	1	0,50	0,38	0
1724170110086	69	Female	30,5	1	0,79	0,24	1
1724170110088	67	Female	25,9	1	0,58	0,37	1
1724170110090	56	Female	30,2	2	0,67	0,45	1
1724170110093	70	Female	31,5	2	0,65	0,38	1
1724170110095	69	Female	30,3	2	0,47	0,33	0
1724170110097	64	Female	21,9	1	0,18	0,45	0
1724170110104	70	Female	31,8	1	0,66	0,33	1
1724170110105	74	Female	27,3	1	0,64	0,28	1
1724170110112	71	Female	30,1	1	0,63	0,25	1
1724170110113	65	Female	37,0	1	0,51	0,33	1
1724170110114	68	Female	27,9	2	0,66	0,29	1
1724170110115	73	Male	30,2	1	0,70	0,24	1
1724170110116	58	Female	38,3	1	0,72	0,37	1
1724170110160	73	Female	33,4	1	0,85	0,30	1
1724170110178	64	Female	35,2	2	0,61	0,44	1
1724170110186	67	Female	28,0	1	0,65	0,56	2
1724170110187	64	Female	24,7	1	0,59	0,38	1
1724170110194	72	Female	24,1	1	0,52	0,39	1
1724170110195	70	Female	24,6	1	0,32	0,57	1
1724170110252	55	Female	29,4	2	0,63	0,38	1
1724170110255	62	Female	23,4	2	0,66	0,35	1
1724170110263	62	Female	35,6	1	0,70	0,37	1
1724170110264	78	Female	29,6	1	0,38	0,43	0
1724170110270	64	Female	43,9	1	0,48	0,42	0

GROUPme	BL_Chair	M6_Chair	BL_Adapt_	M6_Adapt_	BL_Walk	M6_Walk	BL_Walking	M6_Walkir
2	8	10	0	0	50	45	0	1
0	10	11	0	0	41	43	0	0
2	13	10	0	0	30	33	0	1
0	13	13	0	0	36	33	0	0
2	11	10	0	0	33	34	0	0
1	10	9	0	0	34	37	0	0
3	7	8	0	0	41	47	0	0
3	8	10	0	0	41	40	0	0
3	8	9	0	0	42	41	0	0
1	4	4	0	0	61	75	1	1
3	9	9	0	0	35	34	0	0
2	10	12	0	0	34	38	0	0
3	6	5	0	0	55	54	0	0
1	8	8	0	0	35	35	0	0
2	13	#LEEG!	0	#LEEG!	32	#LEEG!	0	#LEEG!
3	5	7	0	0	50	40	0	0
1	10	8	0	0	35	39	0	0
1	6	9	0	0	50	59	0	1
3	16	17	0	0	29	30	0	0
1	9	11	0	0	42	35	0	0
1	9	10	0	0	27	27	0	0
1	11	#LEEG!	0	#LEEG!	25	#LEEG!	0	#LEEG!
3	7	8	0	0	37	35	0	0
1	7	4	0	0	26	24	0	0
1	9	9	0	0	30	21	0	0
1	11	8	0	0	27	27	0	0
3	10	10	0	0	31	29	0	0
2	11	11	0	0	26	25	0	0
1	14	9	0	0	30	28	0	0
2	11	13	0	0	25	25	0	0
2	10	8	0	0	30	24	0	0
2	8	12	0	0	25	16	0	0
2	11	17	0	0	27	19	0	0
2	12	12	0	0	21	22	0	0
3	11	11	0	0	27	27	0	0
1	8	6	0	0	40	36	0	0
2	14	13	0	0	20	22	0	0
0	14	13	0	0	30	28	0	0
0	8	#LEEG!	0	#LEEG!	36	#LEEG!	0	#LEEG!
2	8	#LEEG!	0	#LEEG!	33	#LEEG!	0	#LEEG!
0	8	9	0	0	28	25	0	0
2	9	9	0	0	28	24	0	0
1	9	9	0	0	26	22	0	0
0	11	11	0	0	24	26	0	0
2	12	14	0	0	28	24	0	0
2	18	13	0	0	22	24	0	0
1	18	20	0	0	24	24	0	1
2	10	10	0	0	25	26	0	0
3	13	17	0	0	24	25	0	0

3	7	8	0	0	25	24	0	0
2	11	8	0	0	20	21	0	0
2	8	11	0	0	28	27	0	1
3	11	12	0	0	20	20	0	0
1	11	10	0	0	22	28	0	0
2	16	14	0	0	22	24	0	0
2	11	10	0	0	31	27	0	0
1	12	14	0	0	21	21	0	0
1	10	11	0	0	21	24	0	0
3	7	11	0	0	27	25	0	0
1	13	16	0	0	18	21	0	0
0	10	12	0	0	26	29	0	1
2	9	9	0	0	26	30	0	0
2	11	12	0	0	28	26	0	0
2	14	13	0	0	22	25	0	0
0	13	13	0	0	27	30	0	0
1	11	8	0	0	27	26	0	0
2	10	12	0	0	29	27	0	0
2	12	14	0	0	26	21	0	0
2	8	8	0	0	30	30	0	0
2	11	12	0	0	25	26	0	0
2	8	8	0	0	28	24	0	1
1	9	12	0	0	22	21	0	0
2	9	13	0	0	27	26	0	0
1	6	10	0	0	24	46	0	0
1	13	15	0	0	31	27	0	0
2	10	10	0	0	24	24	0	0
0	6	#LEEG!	0	#LEEG!	33	#LEEG!	0	#LEEG!
0	15	17	0	0	22	22	0	0
3	7	9	0	0	31	26	0	0
1	8	8	0	0	32	34	0	0
2	12	17	0	0	19	23	0	0
3	8	#LEEG!	0	#LEEG!	31	#LEEG!	0	#LEEG!
2	9	#LEEG!	0	#LEEG!	25	#LEEG!	0	#LEEG!
1	12	9	0	0	23	22	0	0
1	8	8	0	0	27	29	0	0
2	12	11	0	0	24	21	0	1
1	11	10	0	0	26	24	0	0
1	7	12	0	0	28	21	0	0
2	9	14	0	0	28	25	0	0
2	7	7	0	0	29	28	0	0
0	9	10	0	0	23	19	0	0
1	7	11	0	0	30	29	0	0
2	12	12	0	0	26	25	0	0
0	12	12	0	0	24	24	0	0
2	13	11	0	0	15	21	0	0
2	15	15	0	0	22	23	0	0
2	11	11	0	0	20	22	0	0
1	#LEEG!	#LEEG!	1	1	42	49	0	0
0	12	15	0	0	24	27	0	0

2	12	12	0	0	22	27	0	0
1	#LEEG!	5	1	0	31	34	0	0
1	9	9	0	0	23	20	0	0
2	11	11	0	0	22	25	0	0
2	5	9	0	0	28	28	0	0
2	14	14	0	0	18	20	0	0
0	9	12	0	0	24	22	0	0
1	10	#LEEG!	0	#LEEG!	27	#LEEG!	0	#LEEG!
2	12	12	0	0	21	25	0	0
0	10	12	0	0	24	27	0	0
2	8	9	0	0	30	31	0	0
1	15	12	0	0	24	24	0	0
2	13	13	0	0	24	25	0	0
0	10	9	0	0	27	27	0	0
2	9	14	0	0	27	23	0	1
0	13	12	0	0	21	27	0	0
0	13	13	0	0	26	26	0	0
0	10	13	0	0	23	27	0	0
0	12	12	0	0	21	25	0	0
2	13	20	0	0	22	21	0	0
0	15	14	0	0	27	26	0	0
2	11	12	0	0	20	21	0	0
3	6	8	0	0	36	30	0	0
2	13	11	0	0	26	25	0	0
0	13	10	0	0	23	27	0	0
1	10	10	0	0	24	25	0	0
1	7	10	0	0	28	25	0	0
1	8	8	0	0	30	28	0	0
0	9	7	0	0	28	30	0	0
1	8	7	0	0	33	38	0	0
3	12	11	0	0	22	23	0	1
2	11	13	0	0	25	26	0	0
3	7	11	0	0	27	21	0	0
1	11	11	0	0	24	25	0	0
2	15	13	0	0	22	24	0	0
3	11	10	0	0	25	26	0	0
1	13	12	0	0	27	30	0	0
0	11	12	0	0	29	24	0	0
2	9	11	0	0	26	26	0	0
2	5	#LEEG!	0	#LEEG!	18	#LEEG!	0	#LEEG!
2	7	10	0	0	22	19	0	0
0	13	12	0	0	19	24	0	0
0	11	10	0	0	26	28	0	0
0	8	8	0	0	31	29	0	0
2	#LEEG!	7	1	0	24	25	0	0
1	4	9	0	0	27	26	0	0
3	13	10	0	0	30	30	0	0
0	12	12	0	0	21	20	0	0
3	9	9	0	0	29	23	0	0
2	15	13	0	0	30	32	0	0

2	11	13	0	0	24	24	0	0
0	9	10	0	0	22	21	0	0
0	14	15	0	0	19	23	0	0
3	12	14	0	0	25	26	0	0
3	5	4	0	0	24	42	0	0
3	10	12	0	0	25	22	0	0
0	12	16	0	0	22	24	0	0
2	10	13	0	0	25	23	0	0
2	15	12	0	0	20	24	0	0
0	9	9	0	0	25	29	0	0
1	7	9	0	0	31	25	0	0
2	12	10	0	0	30	30	0	0
3	7	7	0	0	28	28	0	1
2	15	17	0	0	24	28	0	0
2	9	10	0	0	27	25	0	0
2	11	16	0	0	21	21	0	0
0	11	8	0	0	24	34	0	0
2	9	12	0	0	21	24	0	0
2	12	11	0	0	23	24	0	0
0	10	10	0	0	27	22	0	0
1	12	11	0	0	31	25	0	0
0	15	13	0	0	21	25	0	0
1	12	11	0	0	26	29	0	0
2	13	11	0	0	31	32	0	0
1	26	14	0	0	26	24	0	0
2	10	11	0	0	40	30	1	0
1	8	#LEEG!	0	#LEEG!	39	#LEEG!	0	#LEEG!
2	11	11	0	0	11	32	0	0
3	11	11	0	0	26	29	0	0
1	11	9	0	0	10	27	0	0
1	10	10	0	0	30	29	0	0
2	13	14	0	0	26	29	0	0
3	12	11	0	0	25	28	0	0
2	13	15	0	0	22	23	0	0
1	16	12	0	0	30	28	0	0
0	11	14	0	0	26	30	0	0
1	11	14	0	0	32	30	0	0
1	7	#LEEG!	0	#LEEG!	30	#LEEG!	0	#LEEG!
1	13	9	0	0	42	29	0	0
2	16	12	0	0	24	25	1	0
3	19	11	0	0	28	41	0	0
3	7	8	0	0	28	30	0	0
2	7	7	0	0	31	30	0	0
1	9	10	0	0	33	36	0	0
0	7	8	0	0	37	44	0	0
2	14	15	0	0	30	23	0	0
0	10	9	0	0	34	25	0	0
0	13	14	0	0	37	32	0	0
0	7	9	0	0	62	47	1	1
2	12	14	0	0	25	24	0	0

2	9	9	0	0	34	32	0	0
2	10	10	0	0	29	26	0	0
1	12	13	0	0	25	25	0	1
2	14	14	0	0	28	25	0	0
1	9	11	0	0	25	24	0	0
1	8	9	0	0	43	40	0	0
3	10	10	0	0	29	28	0	0
2	10	10	0	0	30	28	0	0
2	9	16	0	0	25	24	0	0
3	10	8	0	0	27	29	0	0
2	10	8	0	0	23	23	0	0
2	11	12	0	0	33	31	0	0
2	12	13	0	0	26	27	0	0
2	16	15	0	0	25	28	1	0
2	15	12	0	0	27	26	0	0
2	10	11	0	0	28	22	0	0
0	10	9	0	0	34	35	0	0
0	9	10	0	0	25	29	0	0
1	8	11	0	0	32	30	0	0
3	5	4	0	0	35	36	0	0
2	8	6	0	0	27	26	0	0
3	4	7	0	0	37	42	0	0
2	8	11	0	0	27	26	0	0
0	14	16	0	0	23	23	0	0
1	11	13	0	0	33	33	0	0
2	13	11	0	0	23	26	0	0
1	13	11	0	0	23	23	0	0
1	9	9	0	0	35	34	0	0
2	7	7	0	0	28	26	0	0
2	17	13	0	0	20	24	0	0
3	9	8	0	0	27	24	0	0
1	15	18	0	0	22	20	0	0
1	13	12	0	0	22	21	0	0
2	15	14	0	0	25	28	0	0
1	9	#LEEG!	0	#LEEG!	33	#LEEG!	0	#LEEG!
1	12	10	0	0	23	26	0	0
3	11	10	0	0	29	27	0	0
0	9	11	0	0	17	14	0	0
0	9	#LEEG!	0	1	26	43	0	0
2	9	9	0	0	23	29	0	0
2	13	12	0	0	22	27	0	0
1	16	13	0	0	28	24	0	0
1	10	18	0	0	22	22	0	0
1	18	27	0	0	18	18	0	0
1	14	16	0	0	18	21	0	0
3	6	15	0	0	36	29	0	0
1	12	15	0	0	19	21	1	0
2	9	10	0	0	20	24	0	0
1	14	14	0	0	17	22	0	0
1	11	11	0	0	19	22	0	0

1	14	#LEEG!	0	#LEEG!	19	#LEEG!	0	#LEEG!
1	11	12	0	0	23	24	0	0
3	5	8	0	0	47	23	0	0
2	13	16	0	0	19	17	0	0
1	6	8	0	0	45	55	0	1
1	13	12	0	0	17	27	0	1
3	11	11	0	0	34	21	0	0
1	8	4	0	0	57	37	0	1
3	10	10	0	0	23	29	0	0
1	10	10	0	0	30	31	0	0
1	8	8	0	0	29	30	0	0
0	10	#LEEG!	0	#LEEG!	29	#LEEG!	0	#LEEG!
1	12	10	0	0	44	43	0	0
2	2	6	0	0	55	29	0	0
1	8	7	0	0	35	29	0	0
1	11	9	0	0	31	24	0	1
3	9	9	0	0	39	23	0	0
1	9	8	0	0	30	36	0	0
1	5	8	0	0	56	42	0	0
1	7	9	0	0	31	26	0	0
2	12	11	0	0	35	31	0	0
3	18	14	0	0	34	27	0	0
3	11	10	0	0	47	45	0	0
1	4	10	0	0	36	43	0	0
1	6	4	0	0	33	45	0	0
1	6	7	0	0	28	28	0	0
3	7	11	0	0	28	28	0	0
1	7	#LEEG!	0	#LEEG!	40	#LEEG!	0	#LEEG!
1	8	11	0	0	36	24	0	1
2	10	7	0	0	32	58	0	0
1	10	9	0	0	33	22	0	0
1	9	9	0	0	33	31	0	0
1	8	7	0	0	30	29	0	0
1	5	7	0	0	30	24	0	0
1	12	8	0	0	25	34	0	1
1	9	14	0	0	30	30	0	0
1	7	10	0	0	40	26	0	1
1	6	5	0	0	45	40	0	0
3	9	10	0	0	32	26	0	0
3	6	10	0	0	42	38	0	0
1	6	8	0	0	34	26	0	1
1	7	10	0	0	27	40	0	0
2	11	12	0	0	24	25	0	0
1	10	#LEEG!	0	#LEEG!	24	#LEEG!	0	#LEEG!
1	5	11	0	0	36	32	0	0
1	9	7	0	0	30	29	0	0
2	7	10	0	0	27	35	0	0
3	7	7	0	0	33	30	0	0

BL_KOOS_	M6_KOOS_	BL_KOOS_	M6_KOOS_	BL_KOOS_	M6_KOOS_	BL_KOOS_	M6_KOOS_	BL_KOOS_
64,70	82,35	69,40	83,33	31,30	75,00	67,90	82,14	25,00
60,30	88,24	69,40	83,33	68,80	62,50	78,60	85,71	25,00
79,40	82,35	83,30	77,78	68,80	87,50	89,30	92,86	40,00
92,60	95,59	91,70	88,89	75,00	75,00	89,30	75,00	90,00
98,50	98,53	86,10	88,89	87,50	100,00	96,40	100,00	70,00
61,80	64,71	72,20	77,78	31,30	25,00	82,10	78,57	70,00
48,50	48,53	47,20	50,00	31,30	75,00	57,10	53,57	25,00
33,80	33,82	22,20	38,89	12,50	12,50	50,00	57,14	10,00
52,90	64,71	52,80	58,33	25,00	37,50	46,40	53,57	25,00
32,40	52,94	38,90	44,44	0,00	6,25	39,30	39,29	45,00
45,60	44,12	61,10	55,56	50,00	31,25	53,60	42,86	25,00
76,50	54,41	72,20	58,33	81,30	56,25	53,60	67,86	55,00
50,00	36,76	55,60	41,67	18,80	12,50	53,60	39,29	20,00
30,90	29,41	30,60	33,33	12,50	12,50	46,40	28,57	5,00
75,00	#LEEG!	72,20	#LEEG!	75,00	#LEEG!	71,40	#LEEG!	75,00
39,70	50,00	36,10	47,22	31,30	12,50	35,70	28,57	5,00
39,70	63,24	33,30	58,33	0,00	18,75	57,10	46,43	15,00
48,50	39,71	50,00	36,11	12,50	0,00	46,40	39,29	0,00
54,40	92,65	55,60	72,22	37,50	75,00	53,60	71,43	25,00
52,90	54,41	77,80	50,00	43,80	43,75	71,40	75,00	25,00
58,80	50,00	52,80	50,00	50,00	43,75	64,30	50,00	0,00
67,60	#LEEG!	66,70	#LEEG!	43,80	#LEEG!	71,40	#LEEG!	65,00
52,90	55,88	55,60	52,78	50,00	43,75	75,00	67,86	40,00
54,40	39,71	63,90	69,44	56,30	37,50	64,30	57,14	5,00
54,40	48,53	55,60	58,33	43,80	43,75	53,60	50,00	30,00
86,80	80,88	91,70	77,78	56,30	62,50	82,10	82,14	70,00
67,60	64,71	52,80	61,11	50,00	56,25	53,60	71,43	65,00
91,20	72,06	61,10	58,33	56,30	56,25	57,10	50,00	50,00
73,50	76,47	80,60	75,00	62,50	68,75	75,00	71,43	55,00
94,10	92,65	80,60	72,22	68,80	68,75	78,60	78,57	70,00
100,00	100,00	100,00	100,00	87,50	75,00	100,00	100,00	100,00
95,60	98,53	97,20	97,22	75,00	75,00	96,40	100,00	100,00
77,90	80,88	91,70	100,00	81,30	81,25	92,90	85,71	75,00
73,50	77,94	69,40	63,89	25,00	37,50	85,70	85,71	90,00
41,20	48,53	47,20	36,11	50,00	56,25	53,60	64,29	20,00
72,10	48,53	55,60	47,22	37,50	37,50	57,10	60,71	25,00
72,10	89,71	72,20	80,56	62,50	81,25	64,30	64,29	50,00
79,40	76,47	69,40	61,11	56,30	37,50	67,90	67,86	30,00
76,50	79,41	94,40	83,33	75,00	75,00	85,70	85,71	40,00
69,10	#LEEG!	69,40	#LEEG!	56,30	#LEEG!	60,70	#LEEG!	15,00
73,50	70,59	52,80	55,56	62,50	68,75	78,60	89,29	55,00
79,40	76,47	77,80	72,22	56,30	50,00	75,00	78,57	40,00
48,50	64,71	55,60	66,67	68,80	62,50	60,70	75,00	35,00
92,60	85,29	94,40	97,22	81,30	75,00	89,30	82,14	70,00
72,10	72,06	66,70	69,44	62,50	68,75	75,00	71,43	70,00
94,10	89,71	72,20	80,56	62,50	68,75	75,00	71,43	55,00
63,20	66,18	61,10	58,33	43,80	56,25	64,30	64,29	45,00
80,90	92,65	83,30	83,33	37,50	50,00	89,30	92,86	10,00
72,10	75,00	72,20	72,22	75,00	81,25	78,60	82,14	60,00

76,50	86,76	50,00	69,44	31,30	68,75	67,90	75,00	10,00
92,60	95,59	94,40	97,22	75,00	81,25	89,30	92,86	85,00
85,30	94,12	77,80	77,78	62,50	75,00	82,10	78,57	75,00
66,20	47,06	58,30	44,44	68,80	25,00	71,40	57,14	30,00
58,80	54,41	58,30	50,00	56,30	43,75	53,60	64,29	10,00
95,60	100,00	75,00	97,22	62,50	81,25	78,60	100,00	80,00
100,00	98,53	100,00	100,00	100,00	93,75	92,90	96,43	90,00
60,30	63,24	44,40	58,33	31,30	37,50	60,70	57,14	15,00
48,50	41,18	38,90	41,67	68,80	43,75	67,90	50,00	35,00
70,60	67,65	69,40	61,11	25,00	37,50	60,70	64,29	0,00
67,60	66,18	66,70	66,67	62,50	62,50	67,90	75,00	30,00
98,50	98,53	91,70	97,22	75,00	87,50	89,30	92,86	40,00
85,30	70,59	75,00	72,22	62,50	56,25	82,10	78,57	60,00
82,40	88,24	91,70	88,89	81,30	68,75	92,90	92,86	55,00
83,80	63,24	75,00	52,78	43,80	50,00	100,00	82,14	60,00
64,70	55,88	66,70	61,11	43,80	50,00	50,00	50,00	65,00
66,20	60,29	58,30	38,89	62,50	62,50	60,70	57,14	65,00
92,60	86,76	83,30	80,56	68,80	75,00	92,90	92,86	95,00
86,80	70,59	88,90	63,89	68,80	56,25	96,40	85,71	75,00
70,60	45,59	61,10	50,00	62,50	43,75	71,40	42,86	50,00
98,50	82,35	100,00	77,78	81,30	75,00	71,40	75,00	70,00
85,30	70,59	86,10	63,89	62,50	43,75	78,60	53,57	50,00
63,20	67,65	50,00	69,44	43,80	31,25	42,90	71,43	5,00
86,80	92,65	72,20	69,44	75,00	68,75	82,10	85,71	65,00
80,90	82,35	88,90	86,11	68,80	62,50	82,10	82,14	60,00
69,10	63,24	63,90	66,67	62,50	56,25	71,40	75,00	30,00
86,80	88,24	88,90	86,11	93,80	93,75	96,40	89,29	55,00
98,50	#LEEG!	83,30	#LEEG!	62,50	#LEEG!	92,90	#LEEG!	75,00
70,60	69,12	66,70	63,89	62,50	68,75	53,60	60,71	45,00
72,10	76,47	61,10	72,22	62,50	56,25	89,30	71,43	55,00
82,40	75,00	75,00	72,22	68,80	56,25	75,00	75,00	75,00
100,00	98,53	100,00	100,00	93,80	87,50	100,00	100,00	75,00
57,40	#LEEG!	69,40	#LEEG!	25,00	#LEEG!	64,30	#LEEG!	0,00
70,60	#LEEG!	52,80	#LEEG!	43,80	#LEEG!	71,40	#LEEG!	25,00
83,80	83,82	86,10	86,11	81,30	87,50	89,30	85,71	50,00
54,40	58,82	52,80	61,11	43,80	43,75	60,70	57,14	70,00
73,50	82,35	69,40	75,00	68,80	75,00	71,40	85,71	75,00
69,10	85,29	72,20	77,78	75,00	75,00	78,60	85,71	50,00
61,80	67,65	66,70	66,67	43,80	43,75	75,00	64,29	35,00
82,40	77,94	86,10	80,56	62,50	62,50	78,60	67,86	70,00
60,30	63,24	58,30	58,33	31,30	43,75	53,60	50,00	45,00
95,60	97,06	88,90	94,44	75,00	75,00	89,30	92,86	75,00
55,90	51,47	50,00	55,56	43,80	43,75	50,00	50,00	55,00
97,10	97,06	80,60	88,89	68,80	87,50	85,70	82,14	55,00
77,90	52,94	69,40	44,44	68,80	56,25	78,60	78,57	60,00
94,10	100,00	88,90	97,22	56,30	43,75	82,10	78,57	60,00
77,90	98,53	72,20	86,11	43,80	68,75	78,60	92,86	30,00
100,00	97,06	97,20	88,89	62,50	56,25	96,40	96,43	65,00
61,80	61,76	72,20	66,67	43,80	43,75	42,90	75,00	5,00
98,50	100,00	94,40	97,22	75,00	75,00	100,00	100,00	80,00

88,20	51,47	83,30	47,22	75,00	43,75	71,40	67,86	65,00
39,70	39,71	36,10	44,44	43,80	31,25	42,90	39,29	25,00
61,80	63,24	55,60	50,00	31,30	25,00	64,30	46,43	30,00
91,20	92,65	72,20	86,11	75,00	87,50	78,60	75,00	55,00
70,60	94,12	66,70	91,67	50,00	43,75	71,40	85,71	40,00
100,00	100,00	100,00	100,00	87,50	100,00	100,00	100,00	90,00
75,00	85,29	72,20	72,22	81,30	68,75	75,00	92,86	60,00
76,50	#LEEG!	58,30	#LEEG!	37,50	#LEEG!	71,40	#LEEG!	15,00
86,80	88,24	94,40	97,22	87,50	81,25	89,30	85,71	50,00
100,00	100,00	100,00	100,00	100,00	100,00	100,00	100,00	90,00
69,10	85,29	66,70	75,00	68,80	62,50	67,90	78,57	50,00
89,70	92,65	100,00	100,00	87,50	100,00	78,60	96,43	80,00
89,70	91,18	69,40	86,11	62,50	81,25	82,10	92,86	70,00
82,40	75,00	80,60	77,78	43,80	56,25	60,70	67,86	50,00
86,80	95,59	88,90	94,44	62,50	75,00	92,90	96,43	90,00
97,10	100,00	94,40	100,00	81,30	100,00	100,00	100,00	95,00
100,00	100,00	100,00	100,00	100,00	100,00	100,00	100,00	100,00
97,10	91,18	88,90	80,56	75,00	62,50	78,60	71,43	60,00
91,20	97,06	77,80	83,33	81,30	75,00	75,00	82,14	70,00
98,50	95,59	97,20	100,00	93,80	100,00	96,40	89,29	95,00
88,20	85,29	77,80	83,33	62,50	56,25	82,10	75,00	75,00
100,00	100,00	91,70	100,00	93,80	93,75	96,40	92,86	95,00
33,80	39,71	38,90	38,89	56,30	62,50	60,70	53,57	40,00
100,00	98,53	100,00	100,00	100,00	81,25	82,10	85,71	100,00
89,70	97,06	91,70	94,44	81,30	87,50	100,00	96,43	75,00
51,50	57,35	61,10	52,78	37,50	68,75	57,10	67,86	10,00
57,40	72,06	47,20	72,22	43,80	62,50	53,60	64,29	20,00
52,90	52,94	47,20	41,67	31,30	25,00	50,00	50,00	60,00
85,30	79,41	77,80	63,89	62,50	56,25	71,40	64,29	25,00
33,80	22,06	30,60	22,22	31,30	37,50	28,60	32,14	15,00
57,40	52,94	44,40	47,22	43,80	50,00	46,40	50,00	25,00
95,60	100,00	94,40	94,44	87,50	93,75	96,40	96,43	95,00
51,50	50,00	50,00	63,89	25,00	37,50	42,90	64,29	10,00
57,40	64,71	63,90	63,89	50,00	50,00	64,30	57,14	50,00
76,50	91,18	69,40	86,11	81,30	87,50	85,70	82,14	70,00
55,90	60,29	61,10	66,67	31,30	18,75	60,70	64,29	35,00
54,40	50,00	55,60	52,78	18,80	25,00	46,40	53,57	10,00
60,30	35,29	50,00	33,33	62,50	12,50	67,90	53,57	65,00
80,90	95,59	80,60	91,67	81,30	81,25	89,30	89,29	40,00
72,10	#LEEG!	88,90	#LEEG!	81,30	#LEEG!	82,10	#LEEG!	70,00
100,00	100,00	100,00	100,00	100,00	100,00	100,00	92,86	100,00
80,90	64,71	66,70	66,67	50,00	50,00	75,00	85,71	10,00
86,80	88,24	83,30	80,56	68,80	62,50	89,30	85,71	70,00
85,30	91,18	83,30	88,89	62,50	93,75	78,60	75,00	60,00
98,50	100,00	91,70	100,00	87,50	100,00	92,90	100,00	80,00
76,50	54,41	75,00	50,00	43,80	37,50	67,90	60,71	5,00
75,00	82,35	58,30	69,44	50,00	56,25	64,30	82,14	70,00
79,40	100,00	80,60	100,00	68,80	62,50	92,90	100,00	75,00
54,40	69,12	50,00	69,44	50,00	50,00	60,70	78,57	55,00
75,00	82,35	77,80	75,00	56,30	56,25	92,90	92,86	65,00

91,20	95,59	83,30	94,44	75,00	75,00	85,70	89,29	95,00
#LEEG!	80,88	#LEEG!	83,33	#LEEG!	37,50	#LEEG!	85,71	#LEEG!
95,60	95,59	83,30	86,11	68,80	68,75	78,60	82,14	50,00
#LEEG!	50,00	#LEEG!	41,67	#LEEG!	50,00	#LEEG!	60,71	#LEEG!
41,20	86,76	44,40	97,22	25,00	75,00	71,40	75,00	15,00
67,60	45,59	58,30	50,00	#LEEG!	25,00	64,30	57,14	100,00
91,20	89,71	83,30	83,33	62,50	62,50	89,30	92,86	75,00
86,80	83,82	88,90	77,78	68,80	62,50	89,30	64,29	65,00
83,80	57,35	80,60	52,78	68,80	50,00	85,70	67,86	50,00
97,10	98,53	100,00	100,00	68,80	75,00	82,10	92,86	65,00
41,20	35,29	50,00	36,11	50,00	37,50	64,30	78,57	25,00
54,40	66,18	47,20	50,00	37,50	50,00	60,70	67,86	30,00
58,80	72,06	52,80	66,67	43,80	43,75	50,00	50,00	15,00
76,50	85,29	72,20	80,56	37,50	50,00	89,30	96,43	10,00
85,30	97,06	86,10	94,44	50,00	75,00	71,40	92,86	40,00
73,50	85,29	63,90	63,89	37,50	43,75	57,10	71,43	30,00
73,50	73,53	66,70	69,44	43,80	50,00	64,30	64,29	10,00
100,00	100,00	100,00	100,00	81,30	87,50	100,00	100,00	100,00
86,80	98,53	61,10	88,89	68,80	75,00	75,00	92,86	55,00
70,60	77,94	94,40	88,89	25,00	43,75	89,30	71,43	5,00
54,40	67,65	58,30	86,11	25,00	50,00	78,60	89,29	5,00
91,20	82,35	88,90	69,44	56,30	50,00	57,10	60,71	60,00
69,10	45,59	52,80	50,00	43,80	37,50	57,10	64,29	30,00
61,80	85,29	72,20	91,67	50,00	62,50	78,60	78,57	60,00
48,50	70,59	44,40	61,11	56,30	43,75	67,90	78,57	50,00
79,40	70,59	63,90	61,11	43,80	31,25	53,60	35,71	25,00
67,60	#LEEG!	72,20	#LEEG!	43,80	#LEEG!	82,10	#LEEG!	20,00
77,90	86,76	83,30	83,33	56,30	68,75	85,70	75,00	35,00
#LEEG!	54,41	#LEEG!	44,44	#LEEG!	12,50	#LEEG!	46,43	#LEEG!
45,60	50,00	50,00	80,56	12,50	25,00	46,40	57,14	0,00
60,30	48,53	50,00	36,11	50,00	18,75	42,90	35,71	40,00
86,80	89,71	83,30	69,44	75,00	68,75	85,70	89,29	85,00
64,70	92,65	55,60	83,33	50,00	62,50	67,90	89,29	5,00
95,60	98,53	97,20	97,22	68,80	87,50	85,70	96,43	80,00
75,00	45,59	61,10	25,00	31,30	31,25	42,90	39,29	10,00
77,90	76,47	66,70	80,56	50,00	50,00	71,40	75,00	45,00
36,80	35,29	30,60	36,11	50,00	31,25	32,10	25,00	0,00
55,90	#LEEG!	47,20	#LEEG!	31,30	#LEEG!	57,10	#LEEG!	30,00
75,00	75,00	83,30	72,22	56,30	62,50	71,40	71,43	45,00
41,20	77,94	38,90	63,89	37,50	56,25	42,90	57,14	25,00
76,50	83,82	72,20	91,67	31,30	31,25	96,40	96,43	10,00
54,40	55,88	58,30	47,22	62,50	56,25	64,30	64,29	40,00
91,20	82,35	83,30	80,56	75,00	62,50	75,00	78,57	75,00
44,10	38,24	47,20	41,67	25,00	43,75	42,90	53,57	0,00
66,20	45,59	94,40	50,00	75,00	31,25	78,60	50,00	15,00
72,10	77,94	69,40	69,44	50,00	56,25	92,90	82,14	40,00
77,90	44,12	63,90	47,22	50,00	18,75	35,70	35,71	35,00
63,20	76,47	75,00	83,33	50,00	62,50	64,30	71,43	0,00
64,70	39,71	75,00	61,11	87,50	50,00	78,60	60,71	20,00
91,20	86,76	86,10	91,67	68,80	81,25	82,10	82,14	85,00

76,50	66,18	58,30	61,11	62,50	43,75	46,40	42,86	85,00
88,20	89,71	86,10	77,78	75,00	68,75	75,00	78,57	50,00
66,20	44,12	52,80	38,89	43,80	50,00	57,10	53,57	20,00
94,10	85,29	88,90	55,56	75,00	68,75	82,10	82,14	65,00
89,70	88,24	80,60	69,44	62,50	68,75	64,30	60,71	55,00
8,80	14,71	22,20	22,22	25,00	25,00	28,60	39,29	0,00
64,70	82,35	86,10	83,33	50,00	75,00	71,40	67,86	30,00
82,40	89,71	58,30	75,00	62,50	81,25	78,60	82,14	55,00
85,30	92,65	88,90	97,22	56,30	62,50	89,30	89,29	40,00
67,60	64,71	66,70	75,00	37,50	50,00	64,30	82,14	40,00
91,20	94,12	88,90	91,67	43,80	50,00	82,10	92,86	45,00
64,70	75,00	61,10	69,44	56,30	62,50	53,60	60,71	55,00
86,80	69,12	75,00	55,56	62,50	56,25	64,30	53,57	85,00
95,60	92,65	88,90	86,11	87,50	75,00	85,70	89,29	80,00
97,10	92,65	94,40	83,33	68,80	62,50	89,30	92,86	60,00
91,20	91,18	80,60	86,11	81,30	68,75	92,90	100,00	90,00
61,80	50,00	50,00	19,44	43,80	18,75	32,10	25,00	20,00
79,40	82,35	80,60	86,11	62,50	68,75	85,70	89,29	45,00
45,60	44,12	47,20	50,00	43,80	62,50	53,60	50,00	15,00
23,50	10,29	25,00	13,89	0,00	0,00	50,00	32,14	0,00
89,70	85,29	75,00	77,78	50,00	43,75	78,60	75,00	0,00
16,20	14,71	33,30	41,67	50,00	12,50	39,30	35,71	5,00
80,90	75,00	69,40	75,00	75,00	56,25	82,10	85,71	55,00
79,40	80,88	63,90	91,67	18,80	25,00	17,90	75,00	50,00
48,50	42,65	44,40	41,67	50,00	43,75	60,70	57,14	20,00
86,80	91,18	86,10	86,11	68,80	68,75	75,00	71,43	100,00
47,10	80,88	50,00	83,33	18,80	81,25	85,70	82,14	35,00
30,90	39,71	41,70	52,78	31,30	31,25	64,30	75,00	0,00
61,80	35,29	58,30	38,89	50,00	31,25	46,40	50,00	25,00
83,80	82,35	77,80	83,33	56,30	43,75	75,00	85,71	45,00
75,00	66,18	69,40	83,33	56,30	62,50	71,40	50,00	45,00
58,80	60,29	44,40	44,44	43,80	43,75	42,90	50,00	30,00
45,60	45,59	30,60	41,67	37,50	18,75	57,10	53,57	10,00
70,60	76,47	75,00	83,33	68,80	68,75	71,40	75,00	50,00
36,80	#LEEG!	33,30	#LEEG!	12,50	#LEEG!	39,30	#LEEG!	0,00
67,60	39,71	47,20	22,22	81,30	37,50	64,30	50,00	45,00
47,10	69,12	63,90	77,78	37,50	62,50	82,10	92,86	0,00
55,90	66,18	61,10	61,11	43,80	50,00	60,70	57,14	30,00
44,10	54,41	36,10	52,78	12,50	12,50	67,90	75,00	0,00
76,50	76,47	72,20	72,22	43,80	37,50	46,40	39,29	45,00
75,00	51,47	80,60	52,78	75,00	18,75	89,30	46,43	50,00
97,10	75,00	80,60	66,67	50,00	43,75	57,10	71,43	60,00
38,20	44,12	22,20	41,67	25,00	37,50	50,00	67,86	0,00
73,50	61,76	72,20	69,44	75,00	68,75	75,00	82,14	65,00
72,10	86,76	47,20	83,33	50,00	62,50	82,10	89,29	35,00
61,80	66,18	66,70	63,89	31,30	43,75	71,40	75,00	0,00
45,60	69,12	58,30	63,89	43,80	50,00	35,70	46,43	10,00
83,80	70,59	69,40	66,67	68,80	37,50	82,10	75,00	45,00
73,50	60,29	75,00	30,56	68,80	50,00	89,30	71,43	55,00
66,20	55,88	50,00	50,00	37,50	37,50	53,60	50,00	50,00

42,60	#LEEG!	50,00	#LEEG!	50,00	#LEEG!	50,00	#LEEG!	30,00
48,50	35,29	41,70	36,11	37,50	37,50	50,00	67,86	30,00
47,10	39,71	30,60	27,78	31,30	25,00	35,70	53,57	25,00
76,50	95,59	63,90	75,00	31,30	43,75	60,70	71,43	40,00
38,20	35,29	50,00	41,67	12,50	12,50	82,10	85,71	10,00
61,80	64,71	69,40	50,00	25,00	31,25	57,10	71,43	40,00
70,60	58,82	77,80	63,89	31,30	37,50	64,30	67,86	45,00
64,70	55,88	66,70	52,78	31,25	37,50	71,40	85,71	60,00
75,00	100,00	72,20	100,00	25,00	18,75	78,60	85,71	55,00
60,30	50,00	58,30	36,11	37,50	37,50	53,60	53,57	50,00
51,50	50,00	58,30	38,89	31,30	31,25	50,00	57,14	25,00
69,10	#LEEG!	66,70	#LEEG!	56,30	#LEEG!	60,70	#LEEG!	50,00
55,90	26,47	44,40	27,78	50,00	37,50	60,70	46,43	25,00
32,40	63,24	38,90	50,00	18,80	25,00	64,30	71,43	25,00
33,80	50,00	25,00	33,33	31,30	25,00	50,00	53,57	25,00
54,40	52,94	50,00	77,78	31,30	12,50	57,10	78,57	25,00
55,90	69,12	58,30	91,67	37,50	43,75	60,70	85,71	25,00
67,60	66,18	69,40	50,00	62,50	50,00	75,00	75,00	30,00
51,50	26,47	41,70	22,22	31,30	6,25	64,30	35,71	25,00
51,50	63,24	44,40	44,44	31,30	25,00	53,60	67,86	25,00
64,70	85,29	63,90	83,33	62,50	50,00	64,30	82,14	50,00
66,20	89,71	63,90	75,00	31,30	62,50	89,30	92,86	25,00
55,90	19,12	47,20	8,33	37,50	0,00	64,30	10,71	25,00
50,00	52,94	55,60	44,44	25,00	6,25	53,60	60,71	25,00
50,00	45,59	52,80	41,67	31,30	6,25	60,70	42,86	25,00
41,20	32,35	38,90	33,33	31,30	6,25	50,00	67,86	25,00
47,10	83,82	38,90	86,11	31,30	43,75	50,00	42,86	25,00
51,50	#LEEG!	47,20	#LEEG!	37,50	#LEEG!	60,70	#LEEG!	25,00
58,80	89,71	66,70	77,78	50,00	68,75	78,60	82,14	25,00
64,70	66,18	58,30	75,00	50,00	75,00	71,40	67,86	25,00
48,50	52,94	38,90	55,56	31,30	12,50	75,00	85,71	25,00
51,50	63,24	52,80	50,00	31,30	43,75	67,90	89,29	25,00
48,50	38,24	50,00	77,78	37,50	6,25	64,30	92,86	25,00
67,60	25,00	72,20	22,22	37,50	0,00	64,30	7,14	40,00
54,40	77,94	52,80	100,00	56,30	43,75	71,40	100,00	25,00
48,50	36,76	58,30	47,22	37,50	43,75	75,00	64,29	25,00
50,00	69,12	52,80	52,78	31,30	25,00	53,60	57,14	25,00
48,50	63,24	47,20	52,78	43,80	81,25	64,30	53,57	25,00
54,40	57,35	55,60	55,56	43,80	50,00	75,00	53,57	25,00
52,90	63,24	50,00	33,33	43,80	31,25	57,10	71,43	25,00
50,00	73,53	58,30	80,56	62,50	25,00	82,10	85,71	20,00
60,30	75,00	61,10	72,22	68,80	62,50	53,60	89,29	30,00
64,70	91,18	63,90	97,22	43,80	37,50	85,70	78,57	25,00
50,00	39,71	30,60	33,33	43,80	50,00	35,70	28,57	0,00
30,90	29,41	30,60	44,44	12,50	12,50	21,40	17,86	0,00
42,60	20,59	50,00	33,33	31,30	43,75	53,60	25,00	25,00
48,50	92,65	27,80	66,67	31,30	100,00	42,90	71,43	30,00
42,60	100,00	47,20	97,22	56,30	93,75	53,60	92,86	25,00

M6_KOOS_	BL_SF36_Q	GroupQ1	BL_SF36_Q	M6_SF36_(	M6_SF36_(	BL_SF36_Q	BL_SF36_Q	M6_SF36_(
10,00	3	1	50,00	3	50,00	4	25,00	3
30,00	3	1	50,00	3	50,00	4	25,00	3
45,00	3	1	50,00	3	50,00	3	50,00	3
55,00	3	1	50,00	3	50,00	3	50,00	3
90,00	1	2	100,00	2	75,00	3	50,00	3
#LEEG!	3	1	50,00	3	50,00	3	50,00	3
25,00	3	1	50,00	3	50,00	3	50,00	3
15,00	3	1	50,00	3	50,00	4	25,00	4
10,00	3	1	50,00	3	50,00	4	25,00	4
#LEEG!	4	0	25,00	4	25,00	5	0,00	3
35,00	3	1	50,00	3	50,00	3	50,00	3
25,00	3	1	50,00	3	50,00	3	50,00	3
5,00	4	0	25,00	3	50,00	4	25,00	3
5,00	4	0	25,00	4	25,00	4	25,00	4
#LEEG!	3	1	50,00	#LEEG!	#LEEG!	2	75,00	#LEEG!
5,00	3	1	50,00	3	50,00	2	75,00	3
10,00	4	0	25,00	3	50,00	5	0,00	4
0,00	4	0	25,00	4	25,00	4	25,00	5
80,00	3	1	50,00	2	75,00	2	75,00	1
45,00	3	1	50,00	4	25,00	4	25,00	4
5,00	4	0	25,00	4	25,00	4	25,00	4
#LEEG!	3	1	50,00	#LEEG!	#LEEG!	3	50,00	#LEEG!
35,00	3	1	50,00	3	50,00	3	50,00	4
5,00	3	1	50,00	3	50,00	3	50,00	4
15,00	4	0	25,00	4	25,00	4	25,00	3
60,00	3	1	50,00	2	75,00	3	50,00	3
50,00	3	1	50,00	4	25,00	3	50,00	3
35,00	3	1	50,00	3	50,00	3	50,00	3
50,00	4	0	25,00	3	50,00	3	50,00	3
50,00	3	1	50,00	3	50,00	3	50,00	3
100,00	3	1	50,00	3	50,00	4	25,00	3
85,00	3	1	50,00	4	25,00	4	25,00	4
80,00	4	0	25,00	3	50,00	5	0,00	3
75,00	3	1	50,00	3	50,00	4	25,00	3
25,00	3	1	50,00	3	50,00	3	50,00	3
25,00	3	1	50,00	4	25,00	4	25,00	4
55,00	2	2	75,00	2	75,00	3	50,00	3
20,00	2	2	75,00	3	50,00	3	50,00	3
50,00	4	0	25,00	4	25,00	4	25,00	4
#LEEG!	4	0	25,00	#LEEG!	#LEEG!	5	0,00	#LEEG!
65,00	3	1	50,00	3	50,00	1	100,00	1
30,00	3	1	50,00	3	50,00	3	50,00	3
45,00	3	1	50,00	3	50,00	4	25,00	3
55,00	3	1	50,00	3	50,00	3	50,00	3
45,00	3	1	50,00	3	50,00	3	50,00	3
70,00	3	1	50,00	3	50,00	3	50,00	3
35,00	3	1	50,00	3	50,00	3	50,00	3
25,00	2	2	75,00	2	75,00	3	50,00	3
55,00	3	1	50,00	3	50,00	3	50,00	3

15,00	2	2	75,00	3	50,00	3	50,00	4
90,00	2	2	75,00	2	75,00	3	50,00	3
85,00	2	2	75,00	1	100,00	3	50,00	3
15,00	2	2	75,00	2	75,00	3	50,00	3
10,00	4	0	25,00	4	25,00	3	50,00	4
95,00	4	0	25,00	3	50,00	4	25,00	2
90,00	1	2	100,00	#LEEG!	#LEEG!	3	50,00	#LEEG!
5,00	3	1	50,00	3	50,00	3	50,00	3
25,00	2	2	75,00	3	50,00	4	25,00	4
5,00	3	1	50,00	3	50,00	4	25,00	2
35,00	3	1	50,00	4	25,00	4	25,00	3
65,00	2	2	75,00	2	75,00	3	50,00	3
45,00	2	2	75,00	3	50,00	3	50,00	4
40,00	2	2	75,00	2	75,00	3	50,00	3
40,00	3	1	50,00	3	50,00	3	50,00	3
20,00	3	1	50,00	3	50,00	3	50,00	3
25,00	3	1	50,00	3	50,00	3	50,00	3
85,00	4	0	25,00	3	50,00	4	25,00	2
50,00	2	2	75,00	3	50,00	3	50,00	4
0,00	3	1	50,00	3	50,00	3	50,00	3
50,00	3	1	50,00	2	75,00	3	50,00	1
35,00	3	1	50,00	2	75,00	3	50,00	3
15,00	3	1	50,00	3	50,00	4	25,00	4
75,00	1	2	100,00	2	75,00	3	50,00	3
50,00	3	1	50,00	3	50,00	1	100,00	2
30,00	4	0	25,00	4	25,00	4	25,00	3
75,00	1	2	100,00	1	100,00	3	50,00	3
#LEEG!	4	0	25,00	#LEEG!	#LEEG!	5	0,00	#LEEG!
20,00	3	1	50,00	3	50,00	2	75,00	4
45,00	2	2	75,00	3	50,00	3	50,00	3
60,00	3	1	50,00	4	25,00	1	100,00	4
70,00	1	2	100,00	1	100,00	3	50,00	3
#LEEG!	4	0	25,00	#LEEG!	#LEEG!	5	0,00	#LEEG!
#LEEG!	3	1	50,00	#LEEG!	#LEEG!	3	50,00	#LEEG!
45,00	3	1	50,00	3	50,00	3	50,00	3
15,00	3	1	50,00	4	25,00	3	50,00	3
70,00	3	1	50,00	3	50,00	3	50,00	3
70,00	3	1	50,00	3	50,00	3	50,00	3
45,00	3	1	50,00	3	50,00	3	50,00	3
40,00	3	1	50,00	3	50,00	3	50,00	4
40,00	3	1	50,00	4	25,00	3	50,00	3
75,00	2	2	75,00	2	75,00	3	50,00	2
25,00	3	1	50,00	3	50,00	3	50,00	3
65,00	2	2	75,00	2	75,00	3	50,00	3
35,00	3	1	50,00	3	50,00	3	50,00	3
85,00	2	2	75,00	2	75,00	3	50,00	3
85,00	1	2	100,00	1	100,00	3	50,00	3
65,00	2	2	75,00	2	75,00	3	50,00	3
20,00	3	1	50,00	3	50,00	3	50,00	3
75,00	3	1	50,00	4	25,00	4	25,00	5

45,00	3	1	50,00	3	50,00	3	50,00	4
20,00	4	0	25,00	4	25,00	4	25,00	3
20,00	3	1	50,00	3	50,00	4	25,00	4
80,00	3	1	50,00	3	50,00	3	50,00	3
45,00	3	1	50,00	4	25,00	3	50,00	4
100,00	2	2	75,00	2	75,00	1	100,00	1
50,00	2	2	75,00	2	75,00	4	25,00	3
#LEEG!	3	1	50,00	#LEEG!	#LEEG!	3	50,00	#LEEG!
10,00	3	1	50,00	3	50,00	3	50,00	3
100,00	3	1	50,00	3	50,00	3	50,00	3
70,00	3	1	50,00	3	50,00	3	50,00	3
75,00	3	1	50,00	3	50,00	3	50,00	3
95,00	1	2	100,00	1	100,00	3	50,00	4
60,00	3	1	50,00	3	50,00	4	25,00	4
90,00	3	1	50,00	3	50,00	4	25,00	4
100,00	2	2	75,00	2	75,00	3	50,00	3
100,00	2	2	75,00	2	75,00	3	50,00	3
60,00	2	2	75,00	3	50,00	3	50,00	3
75,00	2	2	75,00	2	75,00	3	50,00	3
90,00	2	2	75,00	2	75,00	3	50,00	3
70,00	3	1	50,00	3	50,00	4	25,00	3
100,00	3	1	50,00	3	50,00	4	25,00	3
30,00	3	1	50,00	3	50,00	3	50,00	4
80,00	3	1	50,00	3	50,00	3	50,00	3
95,00	3	1	50,00	3	50,00	3	50,00	3
0,00	3	1	50,00	1	100,00	3	50,00	3
60,00	3	1	50,00	3	50,00	2	75,00	3
45,00	3	1	50,00	3	50,00	3	50,00	3
25,00	3	1	50,00	3	50,00	3	50,00	3
0,00	4	0	25,00	4	25,00	5	0,00	4
30,00	3	1	50,00	3	50,00	3	50,00	2
95,00	2	2	75,00	2	75,00	2	75,00	1
10,00	3	1	50,00	3	50,00	4	25,00	3
30,00	3	1	50,00	3	50,00	3	50,00	3
#LEEG!	2	2	75,00	3	50,00	3	50,00	3
5,00	4	0	25,00	4	25,00	4	25,00	4
5,00	4	0	25,00	4	25,00	3	50,00	3
5,00	4	0	25,00	4	25,00	5	0,00	4
75,00	3	1	50,00	3	50,00	4	25,00	3
#LEEG!	4	0	25,00	#LEEG!	#LEEG!	4	25,00	#LEEG!
100,00	3	1	50,00	4	25,00	3	50,00	4
15,00	3	1	50,00	3	50,00	2	75,00	3
55,00	1	2	100,00	3	50,00	2	75,00	3
65,00	3	1	50,00	#LEEG!	#LEEG!	3	50,00	#LEEG!
95,00	3	1	50,00	3	50,00	2	75,00	3
5,00	3	1	50,00	3	50,00	2	75,00	3
95,00	3	1	50,00	3	50,00	4	25,00	#LEEG!
100,00	2	2	75,00	2	75,00	4	25,00	3
40,00	3	1	50,00	3	50,00	2	75,00	3
65,00	2	2	75,00	3	50,00	3	50,00	3

95,00	3	1	50,00	3	50,00	3	50,00	3
25,00	3	1	50,00	4	25,00	3	50,00	4
60,00	3	1	50,00	2	75,00	3	50,00	3
15,00	3	1	50,00	3	50,00	3	50,00	4
#LEEG!	2	2	75,00	3	50,00	4	25,00	3
5,00	3	1	50,00	3	50,00	3	50,00	3
75,00	3	1	50,00	3	50,00	3	50,00	3
70,00	2	2	75,00	2	75,00	3	50,00	3
30,00	3	1	50,00	3	50,00	3	50,00	3
100,00	3	1	50,00	3	50,00	3	50,00	3
25,00	4	0	25,00	4	25,00	5	0,00	4
35,00	3	1	50,00	3	50,00	3	50,00	4
20,00	3	1	50,00	3	50,00	3	50,00	3
10,00	2	2	75,00	1	100,00	3	50,00	3
75,00	2	2	75,00	2	75,00	3	50,00	3
40,00	3	1	50,00	3	50,00	4	25,00	3
30,00	2	2	75,00	2	75,00	2	75,00	3
90,00	2	2	75,00	2	75,00	3	50,00	3
70,00	3	1	50,00	2	75,00	3	50,00	2
0,00	3	1	50,00	1	100,00	3	50,00	2
30,00	2	2	75,00	2	75,00	3	50,00	3
40,00	3	1	50,00	3	50,00	3	50,00	3
30,00	3	1	50,00	3	50,00	3	50,00	3
75,00	2	2	75,00	2	75,00	3	50,00	2
75,00	3	1	50,00	3	50,00	3	50,00	4
20,00	3	1	50,00	2	75,00	4	25,00	4
#LEEG!	5	0	0,00	#LEEG!	#LEEG!	3	50,00	#LEEG!
40,00	3	1	50,00	3	50,00	3	50,00	3
15,00	#LEEG!	#LEEG!	#LEEG!	3	50,00	#LEEG!	#LEEG!	4
20,00	3	1	50,00	3	50,00	3	50,00	3
40,00	4	0	25,00	4	25,00	4	25,00	4
75,00	3	1	50,00	3	50,00	4	25,00	4
50,00	2	2	75,00	2	75,00	3	50,00	2
90,00	1	2	100,00	1	100,00	3	50,00	3
0,00	4	0	25,00	3	50,00	4	25,00	3
40,00	3	1	50,00	3	50,00	4	25,00	3
15,00	2	2	75,00	2	75,00	3	50,00	2
#LEEG!	4	0	25,00	#LEEG!	#LEEG!	4	25,00	#LEEG!
55,00	3	1	50,00	3	50,00	4	25,00	4
60,00	3	1	50,00	3	50,00	4	25,00	4
40,00	3	1	50,00	3	50,00	3	50,00	2
40,00	4	0	25,00	4	25,00	4	25,00	4
40,00	4	0	25,00	4	25,00	4	25,00	2
0,00	2	2	75,00	2	75,00	3	50,00	4
70,00	3	1	50,00	4	25,00	4	25,00	4
30,00	3	1	50,00	3	50,00	4	25,00	3
40,00	1	2	100,00	#LEEG!	#LEEG!	3	50,00	#LEEG!
10,00	4	0	25,00	3	50,00	2	75,00	2
65,00	4	0	25,00	4	25,00	4	25,00	4
85,00	2	2	75,00	2	75,00	3	50,00	3

45,00	3	1	50,00	3	50,00	4	25,00	1
45,00	3	1	50,00	2	75,00	3	50,00	3
25,00	3	1	50,00	3	50,00	4	25,00	4
60,00	3	1	50,00	3	50,00	3	50,00	3
65,00	3	1	50,00	2	75,00	3	50,00	3
0,00	5	0	0,00	5	0,00	5	0,00	5
60,00	3	1	50,00	3	50,00	3	50,00	3
75,00	3	1	50,00	3	50,00	4	25,00	3
90,00	4	0	25,00	4	25,00	4	25,00	3
45,00	3	1	50,00	3	50,00	3	50,00	3
80,00	1	2	100,00	2	75,00	3	50,00	3
50,00	3	1	50,00	3	50,00	3	50,00	3
55,00	3	1	50,00	3	50,00	3	50,00	3
65,00	3	1	50,00	3	50,00	3	50,00	3
75,00	3	1	50,00	3	50,00	3	50,00	3
60,00	3	1	50,00	3	50,00	2	75,00	4
5,00	3	1	50,00	3	50,00	3	50,00	3
70,00	3	1	50,00	3	50,00	3	50,00	3
25,00	3	1	50,00	2	75,00	3	50,00	3
0,00	4	0	25,00	4	25,00	5	0,00	3
5,00	3	1	50,00	3	50,00	3	50,00	3
5,00	5	0	0,00	5	0,00	5	0,00	5
60,00	3	1	50,00	3	50,00	3	50,00	3
30,00	2	2	75,00	4	25,00	4	25,00	5
30,00	3	1	50,00	3	50,00	3	50,00	4
80,00	4	0	25,00	4	25,00	3	50,00	3
60,00	3	1	50,00	2	75,00	4	25,00	3
10,00	3	1	50,00	4	25,00	3	50,00	3
20,00	2	2	75,00	3	50,00	3	50,00	3
20,00	3	1	50,00	3	50,00	3	50,00	4
40,00	4	0	25,00	3	50,00	4	25,00	3
25,00	2	2	75,00	2	75,00	3	50,00	3
15,00	4	0	25,00	4	25,00	3	50,00	4
45,00	2	2	75,00	3	50,00	3	50,00	3
#LEEG!	3	1	50,00	#LEEG!	#LEEG!	2	75,00	#LEEG!
25,00	3	1	50,00	3	50,00	3	50,00	3
10,00	4	0	25,00	4	25,00	3	50,00	3
35,00	3	1	50,00	3	50,00	3	50,00	3
5,00	5	0	0,00	5	0,00	5	0,00	5
20,00	4	0	25,00	4	25,00	4	25,00	3
25,00	2	2	75,00	4	25,00	3	50,00	5
65,00	4	0	25,00	4	25,00	3	50,00	3
5,00	4	0	25,00	4	25,00	3	50,00	4
55,00	2	2	75,00	2	75,00	3	50,00	3
20,00	2	2	75,00	2	75,00	3	50,00	3
20,00	4	0	25,00	3	50,00	4	25,00	4
15,00	3	1	50,00	3	50,00	4	25,00	3
35,00	1	2	100,00	3	50,00	3	50,00	3
15,00	2	2	75,00	4	25,00	4	25,00	5
25,00	3	1	50,00	3	50,00	4	25,00	3

#LEEG!	3	1	50,00	#LEEG!	#LEEG!	4	25,00	#LEEG!
25,00	3	1	50,00	3	50,00	3	50,00	3
20,00	4	0	25,00	4	25,00	5	0,00	4
50,00	4	0	25,00	3	50,00	3	50,00	3
15,00	4	0	25,00	4	25,00	4	25,00	5
30,00	3	1	50,00	4	25,00	3	50,00	3
50,00	5	0	0,00	5	0,00	5	0,00	5
5,00	4	0	25,00	4	25,00	4	25,00	4
50,00	3	1	50,00	4	25,00	3	50,00	3
0,00	4	0	25,00	3	50,00	4	25,00	4
10,00	4	0	25,00	4	25,00	4	25,00	4
#LEEG!	4	0	25,00	#LEEG!	#LEEG!	3	50,00	#LEEG!
0,00	4	0	25,00	5	0,00	3	50,00	3
25,00	5	0	0,00	4	25,00	5	0,00	1
15,00	4	0	25,00	4	25,00	4	25,00	5
5,00	4	0	25,00	4	25,00	3	50,00	2
25,00	4	0	25,00	3	50,00	5	0,00	1
90,00	4	0	25,00	4	25,00	4	25,00	3
5,00	5	0	0,00	4	25,00	5	0,00	5
15,00	4	0	25,00	4	25,00	4	25,00	3
75,00	4	0	25,00	4	25,00	3	50,00	3
55,00	4	0	25,00	3	50,00	3	50,00	2
0,00	4	0	25,00	4	25,00	3	50,00	4
5,00	4	0	25,00	4	25,00	5	0,00	3
0,00	5	0	0,00	5	0,00	4	25,00	5
0,00	4	0	25,00	4	25,00	4	25,00	5
50,00	4	0	25,00	4	25,00	5	0,00	1
#LEEG!	4	0	25,00	#LEEG!	#LEEG!	5	0,00	#LEEG!
30,00	3	1	50,00	1	100,00	4	25,00	3
70,00	4	0	25,00	3	50,00	4	25,00	3
35,00	4	0	25,00	4	25,00	4	25,00	3
35,00	4	0	25,00	4	25,00	4	25,00	2
0,00	4	0	25,00	4	25,00	4	25,00	4
0,00	5	0	0,00	5	0,00	5	0,00	5
25,00	4	0	25,00	3	50,00	4	25,00	5
0,00	4	0	25,00	3	50,00	5	0,00	4
35,00	5	0	0,00	4	25,00	4	25,00	3
20,00	4	0	25,00	4	25,00	4	25,00	4
25,00	4	0	25,00	4	25,00	4	25,00	4
25,00	5	0	0,00	4	25,00	4	25,00	4
30,00	3	1	50,00	4	25,00	3	50,00	2
75,00	2	2	75,00	3	50,00	3	50,00	3
20,00	4	0	25,00	4	25,00	3	50,00	5
5,00	2	2	75,00	3	50,00	3	50,00	3
0,00	4	0	25,00	5	0,00	4	25,00	5
0,00	3	1	50,00	5	0,00	4	25,00	4
55,00	4	0	25,00	4	25,00	4	25,00	4
100,00	3	1	50,00	3	50,00	3	50,00	1

M6_SF36_(	BL_SF36_p	M6_SF36_|	BL_SF36_r(	M6_SF36_|	BL_SF36_p	M6_SF36_|	BL_HOOS_(	BL_HOOS_|
50,00	50,00	65,00	62,50	93,75	57,14	69,39	61,80	97,50
50,00	50,00	45,00	62,50	50,00	34,69	34,69	44,10	45,00
50,00	80,00	80,00	93,75	100,00	89,80	89,80	100,00	100,00
50,00	80,00	90,00	75,00	93,75	89,80	79,59	92,60	95,00
50,00	80,00	100,00	100,00	100,00	89,80	89,80	95,60	95,00
50,00	25,00	50,00	50,00	37,50	57,14	57,14	61,80	97,50
50,00	45,00	45,00	25,00	50,00	22,45	44,90	55,90	60,00
25,00	50,00	55,00	43,75	56,25	0,00	12,24	23,50	35,00
25,00	50,00	50,00	37,50	50,00	57,14	44,90	54,40	70,00
50,00	15,00	#LEEG!	6,25	6,25	0,00	10,20	44,10	32,50
50,00	70,00	55,00	43,75	62,50	77,55	67,35	38,20	52,50
50,00	65,00	65,00	93,75	68,75	57,14	57,14	94,10	97,50
50,00	40,00	45,00	43,75	31,25	44,90	22,45	100,00	100,00
25,00	20,00	30,00	6,25	25,00	22,45	22,45	35,30	20,00
#LEEG!	50,00	#LEEG!	75,00	#LEEG!	100,00	#LEEG!	100,00	100,00
50,00	30,00	30,00	50,00	62,50	44,90	22,45	60,30	62,50
25,00	25,00	30,00	50,00	50,00	22,45	32,65	88,20	100,00
0,00	20,00	35,00	31,25	12,50	22,45	22,45	35,30	37,50
100,00	70,00	90,00	87,50	100,00	46,94	79,59	98,50	90,00
25,00	40,00	45,00	43,75	62,50	44,90	44,90	22,10	15,00
25,00	50,00	40,00	31,25	43,75	32,65	67,35	77,90	77,50
#LEEG!	50,00	#LEEG!	50,00	#LEEG!	67,35	#LEEG!	64,70	60,00
25,00	50,00	45,00	75,00	56,25	67,35	44,90	52,90	55,00
25,00	50,00	45,00	75,00	68,75	77,55	44,90	79,40	90,00
50,00	30,00	30,00	56,25	37,50	57,14	44,90	55,90	60,00
50,00	65,00	75,00	50,00	81,25	67,35	77,55	86,80	87,50
50,00	45,00	50,00	56,25	81,25	57,14	57,14	77,90	77,50
50,00	65,00	50,00	56,25	68,75	57,14	57,14	91,20	87,50
50,00	55,00	60,00	56,25	43,75	44,90	44,90	72,10	75,00
50,00	80,00	75,00	50,00	50,00	67,35	67,35	92,60	85,00
50,00	100,00	100,00	50,00	100,00	100,00	100,00	100,00	100,00
25,00	85,00	85,00	37,50	62,50	100,00	67,35	100,00	97,50
50,00	55,00	75,00	56,25	93,75	55,10	77,55	61,80	67,50
50,00	55,00	60,00	43,75	18,75	44,90	32,65	79,40	72,50
50,00	55,00	65,00	93,75	87,50	57,14	57,14	41,20	62,50
25,00	20,00	45,00	31,25	31,25	32,65	32,65	55,90	45,00
50,00	85,00	80,00	93,75	100,00	79,59	79,59	95,60	100,00
50,00	75,00	70,00	62,50	62,50	67,35	57,14	100,00	100,00
25,00	50,00	45,00	37,50	37,50	44,90	44,90	58,80	57,50
#LEEG!	35,00	#LEEG!	31,25	#LEEG!	10,20	#LEEG!	82,40	82,50
100,00	70,00	75,00	56,25	68,75	67,35	67,35	73,50	65,00
50,00	50,00	55,00	43,75	37,50	67,35	44,90	82,40	82,50
50,00	50,00	60,00	68,75	87,50	67,35	77,55	77,90	80,00
50,00	85,00	70,00	100,00	75,00	89,80	100,00	98,50	95,00
50,00	95,00	90,00	68,75	62,50	79,59	67,35	83,80	87,50
50,00	75,00	75,00	75,00	75,00	67,35	79,59	85,30	62,50
50,00	60,00	60,00	75,00	18,75	67,35	67,35	100,00	100,00
50,00	60,00	65,00	50,00	100,00	67,35	79,59	94,10	100,00
50,00	60,00	50,00	75,00	62,50	77,55	77,55	100,00	75,00

25,00	50,00	50,00	62,50	25,00	44,90	57,14	70,60	50,00
50,00	70,00	60,00	75,00	100,00	100,00	100,00	100,00	100,00
50,00	80,00	85,00	93,75	75,00	79,59	67,35	100,00	100,00
50,00	60,00	40,00	37,50	31,25	44,90	44,90	69,10	52,50
25,00	65,00	50,00	62,50	62,50	44,90	44,90	63,20	60,00
75,00	90,00	80,00	50,00	50,00	67,35	57,14	95,60	87,50
#LEEG!	95,00	#LEEG!	100,00	100,00	100,00	100,00	95,60	97,50
50,00	55,00	60,00	#LEEG!	87,50	69,39	40,00	63,20	57,50
25,00	45,00	25,00	43,75	25,00	44,90	44,90	72,10	70,00
75,00	50,00	55,00	75,00	68,75	44,90	67,35	51,50	45,00
50,00	65,00	55,00	50,00	50,00	55,10	44,90	66,20	70,00
50,00	90,00	90,00	100,00	100,00	100,00	100,00	100,00	100,00
25,00	55,00	65,00	100,00	93,75	77,55	67,35	89,70	92,50
50,00	75,00	80,00	68,75	87,50	77,55	67,35	86,80	95,00
50,00	85,00	65,00	68,75	62,50	57,14	57,14	100,00	100,00
50,00	55,00	50,00	56,25	62,50	55,10	57,14	86,80	80,00
50,00	50,00	40,00	50,00	75,00	67,35	57,14	76,50	72,50
75,00	75,00	70,00	25,00	56,25	67,35	77,55	100,00	100,00
25,00	75,00	55,00	75,00	81,25	67,35	67,35	91,20	87,50
50,00	75,00	55,00	43,75	87,50	44,90	44,90	100,00	100,00
100,00	85,00	70,00	62,50	62,50	44,90	67,35	45,60	30,00
50,00	80,00	75,00	62,50	50,00	67,35	67,35	100,00	100,00
25,00	50,00	50,00	31,25	31,25	22,45	32,65	55,90	65,00
50,00	95,00	90,00	100,00	93,75	89,80	89,80	97,10	97,50
75,00	75,00	80,00	81,25	62,50	67,35	67,35	75,00	77,50
50,00	50,00	50,00	50,00	56,25	44,90	44,90	64,70	57,50
50,00	65,00	80,00	75,00	100,00	77,55	89,80	94,10	87,50
#LEEG!	35,00	#LEEG!	50,00	#LEEG!	44,90	#LEEG!	42,60	40,00
25,00	65,00	60,00	25,00	31,25	77,55	77,55	98,50	92,50
50,00	75,00	60,00	75,00	62,50	77,55	57,14	70,60	72,50
25,00	85,00	60,00	100,00	50,00	79,59	67,35	85,30	90,00
50,00	100,00	90,00	100,00	100,00	100,00	79,59	100,00	100,00
#LEEG!	35,00	#LEEG!	12,50	#LEEG!	20,41	#LEEG!	41,20	62,50
#LEEG!	45,00	#LEEG!	50,00	#LEEG!	67,35	#LEEG!	98,50	97,50
50,00	65,00	70,00	62,50	50,00	67,35	67,35	92,60	92,50
50,00	55,00	45,00	62,50	81,25	57,14	79,59	100,00	97,50
50,00	50,00	70,00	50,00	100,00	55,10	77,55	79,40	75,00
50,00	80,00	90,00	68,75	100,00	79,59	79,59	63,20	55,00
50,00	65,00	60,00	56,25	50,00	44,90	44,90	66,20	67,50
25,00	65,00	55,00	56,25	75,00	67,35	67,35	77,90	72,50
50,00	55,00	60,00	50,00	56,25	57,14	67,35	55,90	45,00
75,00	90,00	90,00	93,75	100,00	89,80	89,80	91,20	90,00
50,00	85,00	65,00	68,75	50,00	57,14	57,14	50,00	52,50
50,00	80,00	90,00	100,00	100,00	79,59	89,80	100,00	100,00
50,00	85,00	65,00	87,50	68,75	79,59	36,73	100,00	97,50
50,00	100,00	100,00	100,00	100,00	57,14	79,59	91,20	92,50
50,00	70,00	80,00	62,50	75,00	67,35	77,55	85,30	95,00
50,00	90,00	85,00	100,00	81,25	89,80	77,55	97,10	90,00
50,00	20,00	20,00	75,00	62,50	44,90	67,35	100,00	97,50
0,00	90,00	80,00	68,75	25,00	57,14	57,14	100,00	100,00

25,00	85,00	75,00	100,00	81,25	79,59	34,69	100,00	100,00
50,00	35,00	45,00	6,25	25,00	22,45	32,65	36,80	37,50
25,00	60,00	50,00	62,50	62,50	44,90	57,14	66,20	67,50
50,00	90,00	90,00	62,50	68,75	79,59	55,10	91,20	92,50
25,00	70,00	60,00	56,25	31,25	67,35	100,00	100,00	100,00
100,00	90,00	100,00	87,50	100,00	77,55	100,00	100,00	100,00
50,00	70,00	90,00	62,50	81,25	77,55	57,14	82,40	85,00
#LEEG!	65,00	#LEEG!	62,50	#LEEG!	79,59	#LEEG!	82,40	77,50
50,00	65,00	70,00	93,75	100,00	79,59	89,80	100,00	100,00
50,00	80,00	80,00	#LEEG!	68,75	89,80	57,14	92,60	90,00
50,00	65,00	60,00	75,00	68,75	67,35	67,35	67,60	62,50
50,00	75,00	90,00	81,25	100,00	79,59	100,00	61,80	62,50
25,00	60,00	#LEEG!	68,75	62,50	67,35	67,35	95,60	95,00
25,00	65,00	45,00	56,25	62,50	67,35	67,35	79,40	85,00
25,00	65,00	65,00	50,00	50,00	44,90	77,55	66,20	57,50
50,00	100,00	100,00	100,00	100,00	89,80	100,00	100,00	100,00
50,00	100,00	100,00	100,00	100,00	100,00	89,80	100,00	100,00
50,00	90,00	75,00	100,00	68,75	89,80	79,59	100,00	97,50
50,00	90,00	90,00	100,00	87,50	89,80	79,59	85,30	92,50
50,00	95,00	100,00	87,50	100,00	79,59	89,80	98,50	97,50
50,00	65,00	70,00	25,00	50,00	34,69	44,90	86,80	82,50
50,00	80,00	85,00	50,00	43,75	77,55	57,14	100,00	97,50
25,00	40,00	45,00	43,75	62,50	32,65	57,14	29,40	27,50
50,00	80,00	90,00	93,75	100,00	67,35	89,80	77,90	72,50
50,00	70,00	80,00	100,00	56,25	89,80	77,55	100,00	100,00
50,00	80,00	75,00	87,50	100,00	67,35	89,80	72,10	75,00
50,00	45,00	50,00	50,00	75,00	57,14	57,14	67,60	55,00
50,00	60,00	60,00	75,00	75,00	67,35	57,14	58,80	52,50
50,00	85,00	70,00	100,00	62,50	89,80	67,35	100,00	100,00
25,00	0,00	0,00	6,25	6,25	22,45	34,69	72,10	85,00
75,00	60,00	60,00	62,50	50,00	57,14	44,90	61,80	60,00
100,00	95,00	100,00	100,00	100,00	79,59	79,59	95,60	82,50
50,00	55,00	55,00	75,00	93,75	67,35	67,35	69,10	70,00
50,00	55,00	60,00	68,75	50,00	67,35	67,35	69,10	67,50
50,00	65,00	85,00	100,00	100,00	79,59	69,39	92,60	97,50
25,00	55,00	55,00	62,50	75,00	67,35	67,35	100,00	97,50
50,00	25,00	35,00	31,25	18,75	44,90	44,90	55,90	70,00
25,00	45,00	40,00	37,50	25,00	57,14	22,45	57,40	47,50
50,00	80,00	70,00	75,00	37,50	77,55	44,90	88,20	87,50
#LEEG!	60,00	#LEEG!	43,75	#LEEG!	44,90	#LEEG!	73,50	87,50
25,00	80,00	80,00	75,00	50,00	57,14	34,69	100,00	100,00
50,00	75,00	80,00	100,00	75,00	67,35	67,35	92,60	90,00
50,00	55,00	70,00	62,50	62,50	44,90	67,35	80,90	62,50
#LEEG!	65,00	#LEEG!	75,00	#LEEG!	57,14	#LEEG!	#LEEG!	#LEEG!
50,00	45,00	80,00	75,00	87,50	89,80	89,80	80,90	87,50
50,00	55,00	40,00	50,00	43,75	67,35	57,14	73,50	85,00
#LEEG!	55,00	70,00	50,00	62,50	57,14	34,69	86,80	75,00
50,00	100,00	100,00	100,00	100,00	77,55	57,14	100,00	100,00
50,00	60,00	75,00	50,00	100,00	67,35	79,59	100,00	100,00
50,00	#LEEG!	90,00	75,00	75,00	77,55	89,80	100,00	97,50

50,00	30,00	25,00	50,00	56,25	67,35	67,35	70,60	60,00
25,00	60,00	65,00	56,25	56,25	77,55	77,55	#LEEG!	#LEEG!
50,00	85,00	85,00	100,00	100,00	89,80	79,59	100,00	95,00
25,00	60,00	60,00	43,75	25,00	22,45	44,90	#LEEG!	#LEEG!
50,00	50,00	20,00	56,25	100,00	44,90	53,06	42,60	72,50
50,00	65,00	45,00	56,25	50,00	67,35	44,90	72,10	72,50
50,00	90,00	90,00	87,50	100,00	67,35	67,35	100,00	100,00
50,00	80,00	50,00	87,50	62,50	89,80	67,35	100,00	100,00
50,00	85,00	70,00	100,00	62,50	89,80	67,35	100,00	100,00
50,00	55,00	65,00	81,25	100,00	79,59	100,00	#LEEG!	#LEEG!
25,00	35,00	40,00	12,50	6,25	34,69	22,45	100,00	100,00
25,00	70,00	80,00	100,00	81,25	77,55	67,35	92,60	90,00
50,00	55,00	55,00	56,25	75,00	57,14	67,35	83,80	82,50
50,00	65,00	65,00	68,75	87,50	67,35	67,35	69,10	60,00
50,00	60,00	90,00	87,50	100,00	89,80	89,80	100,00	100,00
50,00	65,00	75,00	62,50	62,50	57,14	79,59	100,00	100,00
50,00	60,00	60,00	50,00	75,00	67,35	67,35	91,20	72,50
50,00	100,00	85,00	87,50	87,50	100,00	100,00	100,00	100,00
75,00	65,00	85,00	93,75	100,00	67,35	89,80	100,00	100,00
75,00	60,00	85,00	75,00	100,00	77,55	89,80	100,00	97,50
50,00	30,00	50,00	68,75	56,25	32,65	44,90	100,00	100,00
50,00	85,00	75,00	87,50	75,00	89,80	79,59	100,00	100,00
50,00	100,00	55,00	100,00	100,00	100,00	57,14	100,00	100,00
75,00	55,00	50,00	68,75	50,00	77,55	67,35	100,00	100,00
25,00	50,00	55,00	50,00	75,00	57,14	57,14	63,20	60,00
25,00	50,00	70,00	50,00	100,00	44,90	44,90	95,60	100,00
#LEEG!	15,00	#LEEG!	37,50	#LEEG!	44,90	#LEEG!	54,40	40,00
50,00	80,00	70,00	81,25	75,00	67,35	89,80	95,60	87,50
25,00	#LEEG!	35,00	#LEEG!	75,00	#LEEG!	57,14	#LEEG!	#LEEG!
50,00	55,00	60,00	37,50	31,25	22,45	44,90	54,40	65,00
25,00	55,00	50,00	56,25	37,50	44,90	22,45	100,00	100,00
25,00	70,00	75,00	50,00	62,50	67,35	67,35	91,20	85,00
75,00	65,00	80,00	68,75	100,00	67,35	89,80	64,70	55,00
50,00	95,00	90,00	100,00	100,00	89,80	89,80	95,60	90,00
50,00	45,00	45,00	0,00	43,75	0,00	57,14	75,00	80,00
50,00	55,00	60,00	56,25	50,00	57,14	57,14	75,00	70,00
75,00	50,00	45,00	68,75	62,50	77,55	57,14	98,50	92,50
#LEEG!	45,00	#LEEG!	50,00	#LEEG!	44,90	#LEEG!	69,10	65,00
25,00	70,00	70,00	56,25	50,00	67,35	57,14	85,30	95,00
25,00	55,00	70,00	56,25	68,75	44,90	44,90	100,00	100,00
75,00	55,00	70,00	56,25	68,75	55,10	67,35	92,60	97,50
25,00	80,00	70,00	31,25	25,00	44,90	55,10	58,80	47,50
75,00	50,00	60,00	25,00	56,25	44,90	67,35	88,20	90,00
25,00	50,00	50,00	68,75	43,75	57,14	44,90	41,20	35,00
25,00	40,00	30,00	25,00	25,00	67,35	44,90	72,10	72,50
50,00	65,00	70,00	18,75	18,75	44,90	44,90	97,10	100,00
#LEEG!	55,00	#LEEG!	62,50	#LEEG!	44,90	#LEEG!	94,10	85,00
75,00	30,00	25,00	43,75	50,00	44,90	67,35	41,20	52,50
25,00	35,00	15,00	25,00	31,25	32,65	32,65	20,60	20,00
50,00	80,00	70,00	68,75	56,25	67,35	67,35	98,50	92,50

100,00	65,00	50,00	50,00	75,00	67,35	67,35	73,50	75,00
50,00	75,00	80,00	87,50	87,50	77,55	67,35	75,00	77,50
25,00	40,00	15,00	50,00	25,00	55,10	44,90	73,50	70,00
50,00	85,00	75,00	100,00	100,00	67,35	57,14	94,10	82,50
50,00	90,00	85,00	68,75	93,75	67,35	77,55	100,00	97,50
0,00	5,00	5,00	0,00	6,25	22,45	22,45	4,40	0,00
50,00	55,00	65,00	56,25	81,25	67,35	89,80	64,70	67,50
50,00	50,00	75,00	0,00	25,00	22,45	22,45	41,20	40,00
50,00	55,00	70,00	31,25	43,75	55,10	67,35	100,00	100,00
50,00	75,00	55,00	68,75	50,00	79,59	44,90	69,10	70,00
50,00	75,00	70,00	81,25	100,00	67,35	77,55	100,00	100,00
50,00	75,00	65,00	50,00	50,00	67,35	67,35	82,40	90,00
50,00	85,00	75,00	50,00	62,50	67,35	67,35	88,20	97,50
50,00	80,00	90,00	68,75	87,50	67,35	77,55	100,00	100,00
50,00	90,00	85,00	75,00	87,50	79,59	79,59	98,50	95,00
25,00	80,00	95,00	81,25	93,75	79,59	69,39	92,60	87,50
50,00	50,00	60,00	75,00	75,00	57,14	34,69	100,00	100,00
50,00	75,00	75,00	75,00	75,00	89,80	89,80	91,20	97,50
50,00	35,00	50,00	62,50	50,00	44,90	44,90	23,50	27,50
50,00	5,00	35,00	6,25	18,75	10,20	32,65	22,10	45,00
50,00	60,00	60,00	100,00	62,50	67,35	57,14	61,80	50,00
0,00	45,00	0,00	37,50	0,00	32,65	22,45	16,20	20,00
50,00	65,00	55,00	68,75	68,75	67,35	57,14	69,10	77,50
0,00	55,00	60,00	87,50	43,75	57,14	34,69	100,00	100,00
25,00	60,00	70,00	62,50	75,00	55,10	44,90	61,80	55,00
50,00	65,00	80,00	68,75	43,75	67,35	34,69	100,00	100,00
50,00	50,00	60,00	81,25	93,75	57,14	59,18	54,40	45,00
50,00	35,00	45,00	87,50	68,75	57,14	44,90	100,00	100,00
50,00	80,00	40,00	62,50	56,25	57,14	44,90	100,00	100,00
25,00	65,00	30,00	81,25	75,00	89,80	44,90	95,60	100,00
50,00	55,00	65,00	62,50	50,00	57,14	57,14	73,50	55,00
50,00	70,00	70,00	81,25	75,00	57,14	57,14	97,10	95,00
25,00	60,00	50,00	50,00	50,00	34,69	22,45	91,20	82,50
50,00	85,00	90,00	75,00	68,75	67,35	77,55	100,00	100,00
#LEEG!	35,00	#LEEG!	18,75	#LEEG!	32,65	#LEEG!	89,70	72,50
50,00	75,00	75,00	100,00	75,00	69,39	34,69	100,00	100,00
50,00	50,00	70,00	50,00	81,25	44,90	57,14	66,20	72,50
50,00	75,00	70,00	87,50	87,50	67,35	57,14	97,10	97,50
0,00	30,00	25,00	18,75	18,75	22,45	12,24	97,10	100,00
50,00	65,00	70,00	75,00	75,00	57,14	34,69	86,80	82,50
0,00	85,00	45,00	100,00	31,25	89,80	67,35	100,00	100,00
50,00	85,00	75,00	75,00	62,50	77,55	34,69	97,10	87,50
25,00	55,00	70,00	50,00	87,50	57,14	44,90	50,00	50,00
50,00	80,00	75,00	87,50	100,00	59,18	69,39	79,40	67,50
50,00	90,00	85,00	93,75	100,00	57,14	79,59	100,00	100,00
25,00	40,00	55,00	43,75	43,75	44,90	22,45	54,40	65,00
50,00	25,00	45,00	68,75	93,75	34,69	67,35	100,00	100,00
50,00	80,00	80,00	75,00	62,50	57,14	34,69	73,50	70,00
0,00	60,00	55,00	43,75	31,25	44,90	22,45	100,00	97,50
50,00	60,00	60,00	0,00	12,50	20,41	32,65	100,00	100,00

#LEEG!	20,00	#LEEG!	50,00	#LEEG!	32,65	#LEEG!	100,00	95,00
50,00	80,00	45,00	68,75	50,00	44,90	34,69	77,90	82,50
25,00	45,00	25,00	37,50	25,00	20,41	32,65	55,90	62,50
50,00	75,00	85,00	81,25	75,00	44,90	57,14	69,10	82,50
0,00	10,00	5,00	0,00	0,00	22,45	22,45	44,10	50,00
50,00	55,00	50,00	31,25	0,00	32,65	0,00	89,70	87,50
0,00	25,00	50,00	25,00	25,00	32,65	44,90	89,70	90,00
25,00	25,00	20,00	50,00	43,75	44,90	22,45	63,20	52,50
50,00	50,00	30,00	43,75	87,50	32,65	22,45	73,50	75,00
25,00	50,00	65,00	50,00	62,50	22,45	44,90	72,10	77,50
25,00	55,00	65,00	62,50	50,00	44,90	22,45	100,00	100,00
#LEEG!	65,00	#LEEG!	87,50	#LEEG!	44,90	#LEEG!	100,00	100,00
50,00	55,00	20,00	100,00	81,25	69,39	0,00	100,00	100,00
100,00	25,00	35,00	50,00	50,00	0,00	22,45	61,80	67,50
0,00	40,00	40,00	87,50	50,00	34,69	34,69	100,00	100,00
75,00	30,00	0,00	6,25	0,00	32,65	10,20	66,20	70,00
100,00	30,00	20,00	0,00	18,75	32,65	100,00	100,00	100,00
50,00	70,00	45,00	87,50	87,50	46,94	46,94	66,20	62,50
0,00	40,00	5,00	25,00	0,00	22,45	0,00	72,10	77,50
50,00	45,00	55,00	31,25	37,50	32,65	34,69	100,00	100,00
50,00	55,00	80,00	18,75	75,00	22,45	67,35	100,00	100,00
75,00	75,00	70,00	100,00	50,00	57,14	67,35	97,10	85,00
25,00	60,00	25,00	100,00	6,25	69,39	0,00	89,70	82,50
50,00	40,00	20,00	25,00	37,50	22,45	22,45	100,00	100,00
0,00	45,00	0,00	25,00	0,00	22,45	0,00	92,60	87,50
0,00	45,00	25,00	50,00	0,00	34,69	12,24	100,00	100,00
100,00	40,00	70,00	31,25	50,00	44,90	69,39	95,60	80,00
#LEEG!	30,00	#LEEG!	31,25	#LEEG!	34,69	#LEEG!	100,00	100,00
50,00	65,00	35,00	68,75	100,00	57,14	22,45	100,00	100,00
50,00	50,00	30,00	43,75	50,00	44,90	55,10	100,00	100,00
50,00	55,00	5,00	50,00	25,00	22,45	12,24	100,00	100,00
75,00	60,00	60,00	81,25	50,00	69,39	44,90	100,00	100,00
25,00	55,00	10,00	31,25	0,00	22,45	12,24	100,00	100,00
0,00	35,00	0,00	25,00	0,00	22,45	0,00	72,10	70,00
0,00	60,00	60,00	87,50	50,00	69,39	44,90	100,00	100,00
25,00	30,00	55,00	68,75	87,50	42,86	0,00	70,60	80,00
50,00	35,00	45,00	25,00	25,00	10,20	22,45	85,30	80,00
25,00	40,00	25,00	50,00	18,75	44,90	12,24	75,00	77,50
25,00	35,00	55,00	31,25	25,00	57,14	44,90	100,00	100,00
25,00	30,00	45,00	50,00	43,75	57,14	22,45	75,00	70,00
75,00	45,00	55,00	75,00	62,50	57,14	87,76	100,00	100,00
50,00	55,00	70,00	75,00	56,25	100,00	67,35	100,00	100,00
0,00	35,00	10,00	100,00	0,00	79,59	0,00	88,20	77,50
50,00	50,00	35,00	100,00	68,75	100,00	55,10	100,00	100,00
0,00	20,00	20,00	43,75	25,00	22,45	22,45	70,60	80,00
25,00	20,00	30,00	56,25	25,00	34,69	10,20	100,00	100,00
25,00	35,00	75,00	50,00	100,00	44,90	59,18	100,00	100,00
100,00	25,00	100,00	62,50	100,00	22,45	89,80	100,00	100,00

BL_HOOS_(	BL_HOOS_!	BL_HOOS_!	BL_GS_Spe	M6_GS_Sp	BL_GS_Ran	M6_GS_Ra	BL_GS_Ran	M6_GS_Ra
100,00	95,00	37,50	0,51	0,73	33,47	45,98	27,33	42,29
50,00	75,00	37,50	0,72	0,69	60,28	47,64	55,87	52,65
#LEEG!	85,00	#LEEG!	1,13	1,05	61,61	51,49	57,76	48,32
100,00	95,00	100,00	#LEEG!	#LEEG!	#LEEG!	#LEEG!	#LEEG!	#LEEG!
100,00	95,00	87,50	1,14	0,95	68,07	72,31	66,16	60,17
93,80	85,00	81,30	#LEEG!	#LEEG!	#LEEG!	#LEEG!	#LEEG!	#LEEG!
62,50	50,00	50,00	0,95	0,78	57,77	49,36	56,21	54,66
25,00	50,00	18,80	1,11	0,96	60,35	56,65	57,00	62,96
62,50	70,00	25,00	#LEEG!	#LEEG!	#LEEG!	#LEEG!	#LEEG!	#LEEG!
6,30	30,00	56,30	#LEEG!	#LEEG!	#LEEG!	#LEEG!	#LEEG!	#LEEG!
50,00	60,00	31,30	0,74	0,82	54,53	55,31	55,53	53,62
100,00	80,00	100,00	0,90	0,82	66,77	64,21	63,00	65,60
100,00	95,00	100,00	0,73	0,69	58,89	47,01	57,02	50,29
18,80	35,00	31,30	0,78	0,89	44,01	45,42	52,79	55,97
100,00	100,00	100,00	1,26	#LEEG!	62,96	#LEEG!	60,98	#LEEG!
87,50	75,00	68,80	0,82	1,11	53,03	54,23	58,82	49,86
100,00	85,00	25,00	#LEEG!	#LEEG!	#LEEG!	#LEEG!	#LEEG!	#LEEG!
18,80	25,00	0,00	0,83	0,44	50,94	53,41	55,22	53,23
100,00	85,00	93,80	#LEEG!	#LEEG!	#LEEG!	#LEEG!	#LEEG!	#LEEG!
6,30	50,00	6,30	0,77	0,84	56,09	53,13	56,47	58,42
87,50	75,00	62,50	0,97	0,98	57,32	59,33	55,28	52,29
37,50	80,00	62,50	0,65	#LEEG!	61,55	#LEEG!	57,84	#LEEG!
62,50	35,00	43,80	0,79	0,80	53,13	48,13	62,36	51,25
75,00	85,00	50,00	1,09	1,19	59,63	55,29	59,80	59,38
50,00	50,00	43,80	0,88	1,07	66,95	66,62	65,80	65,80
81,30	80,00	87,50	0,86	0,58	52,51	49,86	59,88	50,74
68,80	75,00	56,30	0,86	0,77	60,73	58,77	63,75	59,14
62,50	100,00	75,00	0,96	1,32	57,20	56,73	62,72	64,88
56,30	65,00	50,00	1,04	0,70	69,67	62,70	68,18	65,02
87,50	95,00	93,80	0,91	1,20	57,99	60,68	61,23	64,42
93,80	100,00	100,00	0,77	1,08	55,57	55,47	58,79	63,69
100,00	95,00	100,00	0,80	1,00	72,56	72,04	66,96	69,71
43,80	40,00	50,00	0,84	0,84	56,52	66,12	60,39	61,05
31,30	85,00	75,00	1,11	1,11	68,49	62,54	68,51	66,37
37,50	65,00	31,30	0,42	1,00	54,23	56,16	52,35	63,08
25,00	55,00	43,80	0,86	0,94	53,78	64,51	63,91	63,33
93,80	95,00	87,50	1,26	1,29	62,37	59,03	62,27	62,84
100,00	100,00	100,00	0,97	0,96	58,57	56,37	62,06	61,59
56,30	60,00	31,30	0,63	#LEEG!	55,22	#LEEG!	52,54	#LEEG!
62,50	90,00	62,50	1,06	#LEEG!	57,95	#LEEG!	57,69	#LEEG!
68,80	70,00	68,80	0,84	1,09	69,50	69,69	72,26	70,88
68,80	80,00	81,30	0,94	1,08	57,89	60,65	60,42	61,79
87,50	90,00	75,00	1,10	1,13	52,15	66,29	53,92	63,38
93,80	100,00	93,80	1,26	1,15	53,30	43,85	55,72	50,94
81,30	90,00	62,50	1,03	1,34	55,08	55,79	55,00	70,27
62,50	50,00	56,30	1,23	1,06	57,85	67,17	57,42	66,26
100,00	100,00	100,00	0,59	1,11	44,31	52,54	47,59	63,87
68,80	100,00	68,80	1,05	1,24	62,60	63,45	60,41	61,97
81,30	70,00	100,00	0,64	0,96	58,28	63,42	54,19	64,33

37,50	80,00	6,30	0,87	0,99	58,78	58,63	57,52	56,10
100,00	100,00	100,00	1,17	1,22	60,45	54,52	59,05	52,39
100,00	90,00	100,00	0,74	0,91	55,40	56,55	50,08	54,19
50,00	70,00	37,50	0,91	1,05	62,69	68,24	65,66	66,82
75,00	60,00	25,00	1,07	0,78	64,40	72,21	66,18	69,94
68,80	95,00	87,50	1,32	1,22	59,25	58,51	59,75	58,44
93,80	100,00	100,00	0,62	1,11	57,78	60,38	54,68	56,95
43,80	55,00	25,00	1,16	1,21	69,52	70,08	57,79	64,62
81,30	60,00	75,00	1,07	0,95	60,29	67,37	66,83	67,26
25,00	35,00	0,00	0,87	1,01	57,77	56,83	56,29	53,92
37,50	80,00	43,80	1,19	1,34	61,61	64,50	64,81	62,17
100,00	100,00	100,00	1,13	0,89	56,90	56,67	54,67	63,86
93,80	70,00	100,00	1,12	0,94	54,70	50,40	57,90	52,52
81,30	90,00	68,80	1,27	1,00	61,11	60,94	62,62	56,40
81,30	100,00	100,00	1,04	1,13	52,32	56,54	57,32	58,33
68,80	80,00	62,50	0,99	0,91	62,64	58,25	55,55	59,73
68,80	45,00	81,30	1,26	1,06	63,28	63,53	65,45	67,50
#LEEG!	100,00	#LEEG!	0,91	0,71	73,05	61,53	71,97	64,26
81,30	80,00	75,00	1,07	1,27	68,36	56,16	72,39	64,08
100,00	100,00	100,00	1,05	0,95	57,18	54,10	49,11	54,28
18,80	25,00	12,50	0,96	1,03	71,52	68,39	63,36	54,78
100,00	100,00	100,00	0,76	1,06	52,84	52,36	52,49	54,95
37,50	55,00	37,50	0,98	1,03	55,72	62,15	61,35	64,53
93,80	90,00	100,00	1,06	1,26	51,58	67,07	58,87	63,57
56,30	55,00	62,50	1,02	0,54	58,84	51,73	58,55	56,20
50,00	45,00	37,50	0,89	1,02	58,07	55,77	62,50	60,53
87,50	90,00	81,30	1,00	1,11	54,30	60,89	60,27	65,63
12,50	50,00	0,00	0,65	#LEEG!	52,41	#LEEG!	54,14	#LEEG!
100,00	95,00	100,00	0,96	1,06	58,88	65,15	57,89	61,10
68,80	75,00	50,00	0,81	0,82	54,51	68,30	58,02	67,73
87,50	90,00	87,50	0,63	0,59	52,37	51,12	54,31	55,13
100,00	100,00	100,00	1,19	1,09	65,60	52,79	61,28	60,48
18,80	60,00	6,30	0,81	#LEEG!	52,47	#LEEG!	51,02	#LEEG!
100,00	95,00	100,00	0,87	#LEEG!	55,26	#LEEG!	72,54	#LEEG!
93,80	100,00	93,80	1,06	1,38	67,57	52,51	55,20	55,58
100,00	65,00	100,00	1,01	1,04	50,27	54,99	49,74	56,66
62,50	55,00	68,80	1,12	1,23	64,37	61,33	67,54	67,07
56,30	50,00	50,00	0,98	1,07	59,27	61,73	66,21	69,20
62,50	60,00	56,30	1,08	1,44	60,58	50,70	59,99	56,33
62,50	80,00	81,30	1,18	1,25	74,21	65,48	66,71	61,00
37,50	45,00	25,00	0,90	0,76	52,54	57,44	47,80	52,06
87,50	95,00	81,30	1,21	1,23	58,02	58,67	58,76	59,35
43,80	55,00	56,30	0,83	0,89	48,20	58,09	49,23	60,62
100,00	100,00	100,00	1,09	0,87	64,51	67,24	71,26	71,80
100,00	100,00	100,00	0,98	1,08	56,42	53,59	58,18	62,05
62,50	80,00	75,00	1,27	1,36	56,56	62,94	55,48	61,79
87,50	80,00	56,30	#LEEG!	#LEEG!	#LEEG!	#LEEG!	#LEEG!	#LEEG!
56,30	90,00	68,80	1,08	1,22	66,55	66,63	67,49	65,96
100,00	100,00	100,00	0,91	0,82	48,72	51,42	54,18	57,60
100,00	100,00	100,00	#LEEG!	#LEEG!	#LEEG!	#LEEG!	#LEEG!	#LEEG!

100,00	95,00	100,00	0,96	1,00	58,99	66,18	64,64	67,74
43,80	50,00	25,00	0,89	0,90	51,22	55,31	49,24	53,06
31,30	60,00	50,00	1,35	1,45	60,60	65,93	63,24	59,93
100,00	95,00	81,30	1,10	1,12	58,20	54,52	57,56	54,58
100,00	100,00	100,00	1,09	0,91	59,07	58,12	60,32	57,71
100,00	100,00	100,00	1,17	1,11	59,52	68,33	66,32	70,40
81,30	90,00	68,80	1,08	1,14	60,40	62,26	61,70	62,88
100,00	75,00	37,50	1,01	#LEEG!	57,90	#LEEG!	57,79	#LEEG!
100,00	100,00	100,00	1,36	1,28	61,03	67,77	65,53	66,09
100,00	95,00	100,00	1,09	1,20	64,61	71,66	59,83	67,79
62,50	70,00	56,30	0,84	1,11	66,60	67,02	63,69	66,15
56,30	70,00	68,80	0,89	0,91	69,34	67,22	68,95	63,20
93,80	100,00	93,80	1,24	1,22	71,20	74,07	63,91	70,62
62,50	85,00	68,80	0,89	0,88	64,94	55,98	59,08	58,55
37,50	60,00	75,00	1,02	1,13	60,73	61,11	64,54	63,74
100,00	100,00	100,00	1,13	1,09	71,36	59,41	68,44	65,36
100,00	100,00	100,00	1,15	1,19	57,01	53,20	59,94	57,68
100,00	100,00	93,80	1,04	1,12	56,27	68,10	66,00	69,36
87,50	70,00	62,50	1,04	1,07	66,25	58,89	58,10	57,92
93,80	100,00	100,00	1,05	0,94	64,68	58,35	62,11	55,13
50,00	80,00	75,00	1,09	1,31	56,02	56,80	61,05	62,70
100,00	95,00	100,00	0,96	0,86	74,88	72,12	75,07	67,09
37,50	50,00	31,30	0,97	#LEEG!	58,01	#LEEG!	59,74	#LEEG!
62,50	70,00	68,80	1,10	1,27	63,65	60,75	64,56	65,23
100,00	95,00	100,00	1,03	1,07	66,91	67,32	64,57	63,80
62,50	85,00	75,00	1,29	1,31	66,41	56,09	58,60	60,01
62,50	60,00	75,00	0,68	1,38	41,69	51,15	43,52	52,37
31,30	35,00	62,50	0,97	0,91	55,75	64,28	57,03	65,84
100,00	100,00	100,00	0,84	1,19	42,49	48,50	48,63	51,62
87,50	65,00	56,30	0,89	0,87	52,62	46,93	56,44	60,39
56,30	45,00	37,50	0,97	0,97	57,19	47,60	56,75	45,78
68,80	90,00	93,80	1,38	1,38	59,83	58,74	59,87	63,16
56,30	65,00	68,80	0,87	1,22	55,12	59,18	51,33	56,34
81,30	75,00	56,30	1,21	1,25	59,22	51,97	59,17	52,69
75,00	100,00	87,50	1,38	1,19	58,66	46,74	61,40	48,49
100,00	90,00	87,50	1,21	1,30	56,39	57,68	55,33	51,91
50,00	70,00	37,50	1,14	1,18	51,71	52,28	49,38	55,26
68,80	65,00	87,50	1,38	1,08	47,75	52,57	49,98	58,85
75,00	55,00	81,30	0,96	1,14	70,68	67,58	67,90	67,68
87,50	75,00	62,50	1,13	#LEEG!	43,24	#LEEG!	51,62	#LEEG!
100,00	100,00	100,00	1,11	1,21	68,46	61,62	69,34	69,67
50,00	95,00	75,00	1,14	1,08	43,08	46,59	52,28	48,32
50,00	70,00	37,50	1,09	1,11	61,60	51,56	57,55	54,78
#LEEG!	#LEEG!	#LEEG!	1,05	0,82	61,89	54,99	60,13	56,24
93,80	90,00	87,50	1,08	1,15	55,23	61,61	53,59	59,41
56,30	80,00	43,80	1,02	1,05	43,75	36,08	48,90	62,99
81,30	85,00	81,30	0,90	1,11	63,30	57,74	52,08	41,38
100,00	100,00	100,00	1,14	1,43	61,18	54,32	69,74	56,84
100,00	100,00	100,00	0,76	1,00	48,86	47,36	60,69	55,99
100,00	100,00	100,00	0,85	0,84	52,28	56,78	60,92	57,30

43,80	50,00	62,50	0,77	1,06	54,58	64,46	54,96	65,62
#LEEG!	#LEEG!	#LEEG!	1,22	1,37	61,30	62,44	65,40	67,79
81,30	100,00	100,00	1,48	1,28	58,44	52,53	52,58	52,87
#LEEG!	#LEEG!	#LEEG!	0,99	1,09	56,67	54,51	61,91	54,22
93,80	100,00	25,00	0,71	0,88	39,82	42,36	52,29	51,00
68,80	80,00	68,80	0,82	0,93	62,97	65,35	56,66	64,96
93,80	100,00	100,00	1,19	1,32	61,51	66,37	56,10	66,53
100,00	100,00	100,00	1,32	1,33	67,31	58,84	61,91	58,42
100,00	100,00	100,00	0,95	0,85	66,09	64,34	65,27	62,40
#LEEG!	#LEEG!	#LEEG!	#LEEG!	#LEEG!	#LEEG!	#LEEG!	#LEEG!	#LEEG!
100,00	100,00	100,00	0,79	0,87	59,86	52,51	56,25	60,12
93,80	95,00	75,00	0,74	0,98	52,85	47,47	60,91	66,11
75,00	70,00	56,30	#LEEG!	#LEEG!	#LEEG!	#LEEG!	#LEEG!	#LEEG!
50,00	80,00	31,30	0,91	1,08	65,21	63,47	71,34	71,08
100,00	100,00	100,00	0,84	1,24	44,04	53,51	49,53	62,38
100,00	100,00	100,00	0,89	0,98	64,74	66,60	69,25	67,21
68,80	100,00	93,80	0,71	0,78	55,49	58,83	57,20	60,28
100,00	100,00	100,00	1,25	1,18	52,96	44,00	49,80	44,22
100,00	100,00	100,00	0,92	1,05	50,57	48,64	62,26	56,47
62,50	60,00	100,00	0,95	1,16	48,94	47,98	49,40	43,35
100,00	100,00	100,00	0,83	1,08	55,83	55,08	54,77	57,47
100,00	95,00	100,00	1,36	1,19	59,04	52,78	63,19	69,46
100,00	100,00	100,00	1,33	1,00	52,77	54,82	48,21	51,97
100,00	100,00	100,00	0,80	0,92	46,57	47,25	47,54	49,70
50,00	85,00	56,30	1,24	1,45	60,33	63,63	64,82	64,84
100,00	85,00	100,00	1,12	1,29	52,14	56,75	55,80	60,62
37,50	70,00	12,50	0,87	#LEEG!	59,76	#LEEG!	58,13	#LEEG!
81,30	90,00	87,50	1,08	1,10	61,83	60,04	62,53	67,62
#LEEG!	#LEEG!	#LEEG!	1,14	#LEEG!	49,56	#LEEG!	61,12	#LEEG!
81,30	50,00	12,50	0,78	0,99	65,66	58,83	63,48	65,75
100,00	100,00	100,00	1,00	1,01	61,68	60,82	64,17	59,05
81,30	90,00	93,80	1,18	1,22	67,17	68,30	67,59	66,64
62,50	50,00	31,30	1,07	1,03	60,66	63,33	68,50	72,62
100,00	85,00	87,50	1,21	1,26	56,47	62,07	54,67	57,76
100,00	90,00	75,00	1,01	1,06	66,02	65,93	65,87	60,75
56,30	60,00	62,50	1,10	1,28	68,08	55,98	65,46	58,32
100,00	85,00	100,00	0,98	0,90	54,29	50,77	59,44	58,57
62,50	70,00	43,80	1,55	#LEEG!	61,21	#LEEG!	63,24	#LEEG!
75,00	95,00	81,30	1,13	0,99	64,82	60,99	64,15	54,70
100,00	100,00	100,00	0,95	1,11	54,85	64,46	57,46	62,70
93,80	85,00	93,80	1,02	0,78	62,02	56,96	54,28	53,18
68,80	50,00	43,80	0,99	1,02	66,77	60,52	60,41	59,79
81,30	70,00	93,80	0,88	0,87	61,04	67,67	58,55	55,89
43,80	40,00	0,00	#LEEG!	#LEEG!	#LEEG!	#LEEG!	#LEEG!	#LEEG!
62,50	60,00	50,00	0,84	0,78	59,52	53,73	64,23	65,11
87,50	95,00	62,50	1,22	1,28	65,29	68,71	69,74	69,79
81,30	55,00	81,30	#LEEG!	#LEEG!	#LEEG!	#LEEG!	#LEEG!	#LEEG!
43,80	45,00	0,00	1,00	0,95	54,95	56,99	57,87	59,54
25,00	40,00	75,00	#LEEG!	#LEEG!	#LEEG!	#LEEG!	#LEEG!	#LEEG!
87,50	90,00	100,00	1,16	1,22	64,27	62,56	66,39	65,33

50,00	55,00	81,30	0,98	1,15	61,57	72,70	67,81	69,76
68,80	90,00	56,30	1,13	1,25	69,70	73,03	77,90	69,14
56,30	50,00	68,80	1,06	1,09	57,52	54,65	60,39	52,13
87,50	75,00	81,30	1,15	1,28	64,48	63,15	64,15	57,83
100,00	90,00	62,50	1,18	1,07	49,90	56,44	50,29	58,15
6,30	0,00	0,00	0,74	0,77	63,91	62,24	64,83	57,36
62,50	50,00	56,30	0,94	0,99	58,02	62,85	63,46	61,17
18,80	15,00	25,00	0,93	1,00	62,06	58,86	57,62	66,17
100,00	100,00	100,00	1,17	1,21	71,89	68,48	70,50	73,58
43,80	70,00	68,80	1,13	1,16	59,38	60,61	53,18	60,30
100,00	100,00	100,00	1,23	1,26	66,21	61,61	65,48	60,90
93,80	70,00	68,80	1,01	0,99	59,17	55,91	67,64	56,22
75,00	90,00	93,80	1,16	1,12	59,40	56,13	63,88	60,31
100,00	80,00	100,00	0,90	0,95	58,08	60,44	58,33	58,74
75,00	90,00	75,00	1,03	#LEEG!	58,19	#LEEG!	62,78	#LEEG!
87,50	95,00	93,80	1,35	1,36	67,65	69,64	60,42	67,05
100,00	100,00	100,00	0,91	0,81	49,19	51,41	57,88	62,40
93,80	85,00	93,80	1,20	1,04	60,31	64,39	60,29	62,20
18,80	25,00	6,30	1,03	1,01	63,29	59,02	64,21	64,84
25,00	35,00	12,50	0,95	0,94	45,00	47,31	39,01	45,75
43,80	75,00	62,50	1,12	1,31	58,99	65,63	62,07	59,50
37,50	35,00	12,50	0,55	0,65	58,27	44,40	51,66	49,97
56,30	80,00	50,00	0,85	1,15	62,79	67,17	63,73	65,41
100,00	100,00	100,00	1,21	1,26	64,05	65,33	66,70	63,95
50,00	65,00	50,00	0,95	1,00	54,88	53,88	58,03	55,00
100,00	100,00	100,00	0,89	0,83	60,59	59,02	61,18	59,35
43,80	55,00	56,30	1,16	1,05	63,95	62,10	62,78	65,53
100,00	100,00	100,00	0,85	0,82	66,60	60,44	62,34	66,44
100,00	100,00	100,00	1,15	0,87	51,29	46,66	52,73	56,04
93,80	85,00	81,30	0,96	0,94	59,93	61,51	61,85	64,04
43,80	45,00	43,80	0,88	1,13	48,35	49,84	46,79	49,84
100,00	100,00	100,00	1,15	1,25	50,90	49,65	56,37	56,78
87,50	90,00	93,80	1,03	0,92	60,31	61,60	60,76	64,10
100,00	95,00	100,00	0,87	0,92	63,82	58,56	59,09	56,10
50,00	70,00	68,80	0,98	#LEEG!	53,87	#LEEG!	62,78	#LEEG!
100,00	95,00	100,00	0,99	1,14	58,62	63,22	62,62	67,63
62,50	90,00	31,30	1,05	0,93	58,79	56,45	57,89	53,64
87,50	85,00	81,30	0,88	1,07	66,38	66,94	69,60	69,19
100,00	100,00	68,80	0,78	0,68	53,81	40,07	54,49	50,79
50,00	70,00	62,50	1,02	1,12	62,87	56,62	58,21	61,45
100,00	100,00	100,00	1,18	1,25	64,93	54,65	70,21	60,89
81,30	95,00	100,00	0,92	0,99	56,03	61,71	60,82	64,99
68,80	65,00	37,50	1,01	0,86	58,64	62,44	61,11	62,55
56,30	35,00	81,30	1,11	#LEEG!	63,70	#LEEG!	71,67	#LEEG!
100,00	100,00	100,00	1,00	0,96	64,83	60,97	67,47	66,81
43,80	60,00	18,80	0,71	#LEEG!	51,21	#LEEG!	50,03	#LEEG!
100,00	90,00	100,00	0,83	0,80	61,65	70,14	64,81	62,97
68,80	65,00	68,80	0,95	0,90	65,32	62,57	63,51	62,06
100,00	95,00	100,00	1,31	1,31	57,51	51,29	63,56	61,86
100,00	85,00	100,00	1,24	1,02	74,02	72,31	71,23	64,99

68,80	100,00	81,30	1,18	#LEEG!	59,07	#LEEG!	67,85	#LEEG!
75,00	80,00	75,00	0,72	0,85	59,35	60,07	59,03	55,99
43,80	55,00	50,00	1,03	1,12	61,95	60,89	62,01	64,83
50,00	65,00	62,50	0,84	1,01	54,82	58,44	60,35	62,87
43,80	45,00	18,80	0,56	0,48	43,19	37,72	49,76	50,53
68,80	80,00	81,30	1,28	1,10	52,17	51,86	55,67	46,06
62,50	65,00	87,50	0,73	0,91	58,02	47,03	63,03	52,38
43,80	75,00	62,50	0,68	0,64	64,47	44,54	59,26	37,33
43,80	75,00	75,00	1,01	1,06	58,60	49,32	62,63	44,51
50,00	90,00	75,00	0,81	0,65	62,56	57,18	64,30	65,73
100,00	100,00	100,00	0,82	0,83	50,39	60,48	59,71	66,45
100,00	100,00	100,00	0,98	#LEEG!	63,71	#LEEG!	64,80	#LEEG!
100,00	100,00	100,00	0,73	0,65	60,96	52,14	55,63	52,86
43,80	80,00	56,30	0,62	0,81	59,26	54,96	52,90	58,96
100,00	100,00	100,00	0,90	0,97	53,08	49,42	49,56	52,11
37,50	60,00	50,00	0,75	0,73	52,39	45,38	36,20	50,01
100,00	100,00	100,00	0,66	0,88	50,33	45,20	64,91	54,26
75,00	100,00	56,30	0,79	0,56	65,00	42,29	66,01	44,17
50,00	85,00	75,00	0,59	0,51	51,49	42,38	43,28	47,81
100,00	100,00	100,00	0,94	1,03	54,10	47,30	52,54	49,37
100,00	100,00	100,00	0,91	0,84	50,41	48,15	56,81	54,72
68,80	100,00	87,50	0,93	0,91	59,94	60,43	68,19	56,34
68,80	100,00	68,80	0,97	0,73	35,10	50,18	46,42	47,51
100,00	100,00	100,00	0,69	0,68	37,69	52,63	43,35	54,83
68,80	100,00	93,80	0,51	0,43	54,97	48,32	55,98	50,27
100,00	100,00	100,00	0,90	0,74	45,86	55,35	59,05	53,82
68,80	95,00	75,00	0,90	0,89	40,56	49,39	51,99	52,87
100,00	100,00	100,00	0,75	#LEEG!	44,28	#LEEG!	37,92	#LEEG!
100,00	100,00	100,00	0,80	0,76	45,06	51,34	48,89	53,54
100,00	100,00	100,00	0,97	#LEEG!	44,80	#LEEG!	51,38	#LEEG!
100,00	100,00	100,00	1,03	0,98	57,14	59,90	55,02	61,21
100,00	100,00	100,00	0,70	0,74	47,78	44,78	55,42	50,93
100,00	100,00	100,00	0,59	0,69	57,12	50,03	56,93	64,71
37,50	80,00	62,50	0,59	0,83	61,53	60,72	61,87	63,53
100,00	100,00	100,00	1,08	0,70	62,26	55,16	63,61	29,86
68,80	90,00	50,00	0,94	0,86	50,98	63,72	50,94	60,80
68,80	95,00	75,00	0,90	0,82	45,01	55,19	43,57	50,36
56,30	80,00	50,00	0,55	0,59	42,74	19,43	45,07	38,94
100,00	100,00	100,00	0,66	0,76	54,15	55,71	47,64	56,27
62,50	80,00	62,50	0,58	0,53	48,92	50,92	46,41	50,24
100,00	100,00	100,00	0,93	0,94	54,91	60,91	54,00	60,95
87,50	100,00	75,00	0,72	0,77	62,13	54,88	57,42	51,13
68,80	95,00	81,30	0,92	0,93	61,08	55,02	59,37	62,38
100,00	100,00	100,00	1,02	#LEEG!	49,03	#LEEG!	54,29	#LEEG!
50,00	80,00	56,30	0,72	0,83	47,30	47,06	42,05	48,20
100,00	70,00	56,30	1,04	0,80	52,55	31,29	56,96	51,30
100,00	100,00	100,00	0,96	0,94	65,23	46,17	57,50	53,02
100,00	100,00	100,00	0,79	0,92	51,49	45,47	52,51	44,18

BL_GS_Diff	M6_GS_Dif	BL_GS_Ran	M6_GS_Ra	BL_GS_Ran	M6_GS_Ra	BL_GS_Diff	M6_GS_Dif	BL_GS_Ave
6,14	3,69	40,37	50,47	39,37	51,91	1,00	1,44	1,2
4,41	5,01	67,05	61,82	64,97	64,21	2,07	2,39	1,1
3,85	3,17	77,67	69,74	72,93	61,77	4,73	7,97	1,2
#LEEG!	#LEEG!	#LEEG!	#LEEG!	#LEEG!	#LEEG!	#LEEG!	#LEEG!	#LEEG!
1,91	12,13	80,08	78,16	76,58	72,74	3,51	5,42	1,0
#LEEG!	#LEEG!	#LEEG!	#LEEG!	#LEEG!	#LEEG!	#LEEG!	#LEEG!	#LEEG!
1,56	5,30	72,01	58,79	72,33	65,77	0,32	6,98	1,1
3,36	6,31	73,52	63,18	66,00	68,68	7,53	5,50	1,1
#LEEG!	#LEEG!	#LEEG!	#LEEG!	#LEEG!	#LEEG!	#LEEG!	#LEEG!	#LEEG!
#LEEG!	#LEEG!	#LEEG!	#LEEG!	#LEEG!	#LEEG!	#LEEG!	#LEEG!	#LEEG!
1,00	1,69	62,29	64,99	63,81	61,94	1,52	3,05	1,0
3,77	1,39	77,57	74,17	76,48	76,60	1,09	2,43	1,1
1,87	3,28	68,92	59,78	69,52	64,94	0,60	5,17	1,2
8,78	10,55	56,72	57,33	60,42	66,46	3,69	9,13	0,9
1,98	#LEEG!	79,66	#LEEG!	73,85	#LEEG!	5,81	#LEEG!	1,0
5,79	4,38	61,41	59,86	60,30	61,78	1,11	1,92	1,3
#LEEG!	#LEEG!	#LEEG!	#LEEG!	#LEEG!	#LEEG!	#LEEG!	#LEEG!	#LEEG!
4,27	0,17	68,98	63,49	64,54	56,23	4,45	7,26	1,1
#LEEG!	#LEEG!	#LEEG!	#LEEG!	#LEEG!	#LEEG!	#LEEG!	#LEEG!	#LEEG!
0,38	5,28	68,98	66,40	72,28	69,63	3,31	3,23	1,2
2,04	7,04	70,07	72,72	70,16	69,04	0,10	3,68	1,0
3,71	#LEEG!	73,16	#LEEG!	69,33	#LEEG!	3,83	#LEEG!	1,2
9,23	3,12	67,58	61,02	69,39	65,99	1,81	4,97	1,2
0,17	4,09	73,45	72,72	76,18	74,81	2,73	2,09	1,1
1,15	0,82	80,31	84,18	77,65	79,58	2,66	4,60	1,2
7,37	0,88	67,39	61,82	69,89	59,51	2,50	2,31	1,0
3,02	0,37	72,86	65,76	73,24	70,35	0,38	4,60	1,0
5,52	8,15	68,85	77,63	72,34	79,01	3,49	1,39	1,1
1,49	2,32	79,03	71,39	76,28	72,87	2,74	1,49	1,2
3,24	3,74	72,56	77,19	76,93	79,73	4,37	2,54	1,2
3,22	8,22	68,23	77,15	73,90	81,84	5,67	4,70	1,4
5,60	2,34	68,94	76,76	71,91	73,90	2,97	2,87	1,2
3,88	5,07	71,49	73,81	73,27	73,48	1,78	0,33	1,1
0,02	3,83	81,70	77,59	85,31	80,96	3,62	3,37	1,2
1,89	6,91	64,97	72,13	62,78	78,42	2,20	6,30	1,6
10,14	1,18	67,53	77,43	70,72	75,29	3,20	2,15	1,2
0,10	3,81	79,40	76,95	77,95	80,20	1,45	3,25	1,0
3,49	5,22	68,90	71,83	69,01	71,58	0,11	0,24	1,0
2,68	#LEEG!	65,53	#LEEG!	66,00	#LEEG!	0,47	#LEEG!	1,1
0,26	#LEEG!	71,58	#LEEG!	73,77	#LEEG!	2,19	#LEEG!	1,2
2,76	1,18	77,33	80,26	78,59	82,56	1,26	2,30	1,3
2,52	1,15	74,20	73,82	72,57	74,03	1,63	0,21	1,0
1,77	2,90	75,02	76,35	78,34	74,80	3,32	1,54	1,1
2,42	7,09	74,94	65,49	74,36	69,48	0,58	3,99	1,0
0,08	14,48	71,70	78,58	68,98	78,40	2,72	0,18	1,1
0,43	0,92	74,32	78,33	74,06	76,36	0,26	1,97	1,0
3,27	11,33	53,53	72,47	65,51	77,86	11,98	5,39	1,3
2,19	1,48	78,16	83,93	77,81	80,71	0,36	3,22	1,2
4,09	0,91	67,25	74,88	61,42	76,30	5,83	1,43	1,3

1,25	2,53	74,65	74,61	73,02	74,17	1,63	0,44	1,1
1,40	2,13	77,04	74,06	76,18	73,44	0,86	0,62	1,0
5,32	2,37	69,46	70,19	65,42	67,05	4,04	3,15	1,1
2,97	1,42	73,48	79,13	74,76	75,57	1,28	3,56	1,2
1,78	2,26	82,11	77,79	81,99	75,59	0,12	2,20	1,2
0,50	0,07	77,97	73,14	78,60	72,95	0,63	0,20	1,0
3,10	3,43	64,90	74,66	60,47	75,72	4,43	1,06	1,2
11,74	5,47	81,39	82,49	72,49	76,07	8,90	6,42	1,1
6,53	0,11	81,24	79,72	84,41	82,55	3,17	2,83	1,2
1,49	2,91	71,02	71,27	72,35	74,34	1,33	3,07	1,1
3,20	2,33	76,69	80,33	78,78	74,73	2,09	5,61	1,0
2,23	7,19	71,94	69,27	77,97	73,42	6,02	4,15	1,0
3,20	2,12	69,21	64,31	74,46	68,60	5,25	4,30	1,2
1,51	4,54	78,85	74,16	77,58	72,19	1,28	1,98	1,1
4,99	1,79	74,65	76,23	76,14	76,03	1,49	0,20	1,1
7,08	1,48	75,69	70,36	73,71	69,58	1,98	0,78	1,0
2,16	3,97	75,55	73,89	76,46	77,61	0,91	3,72	1,0
1,08	2,73	75,97	71,94	78,12	71,23	2,15	0,70	1,2
4,04	7,91	79,57	75,48	78,27	77,97	1,31	2,49	1,1
8,07	0,18	74,08	72,23	70,56	74,01	3,51	1,78	1,0
8,16	13,61	84,01	77,77	78,06	72,01	5,95	5,76	1,1
0,35	2,59	66,13	70,06	65,58	69,40	0,55	0,66	1,3
5,63	2,38	69,72	72,91	75,74	73,68	6,02	0,77	1,0
7,30	3,49	66,61	82,73	70,11	76,36	3,49	6,37	1,1
0,30	4,47	75,28	62,26	74,53	63,90	0,75	1,64	1,2
4,43	4,76	69,75	73,16	70,40	73,71	0,65	0,55	1,0
5,97	4,74	67,80	75,30	71,10	74,60	3,30	0,70	1,0
1,73	#LEEG!	66,91	#LEEG!	68,55	#LEEG!	1,64	#LEEG!	1,1
0,99	4,05	73,45	78,47	73,48	75,07	0,03	3,40	1,2
3,50	0,57	71,99	76,94	73,03	78,46	1,04	1,52	1,3
1,94	4,01	66,22	63,47	68,13	64,42	1,91	0,95	1,2
4,32	7,69	75,70	69,18	74,93	70,47	0,77	1,29	1,0
1,46	#LEEG!	70,60	#LEEG!	68,29	#LEEG!	2,31	#LEEG!	1,2
17,27	#LEEG!	65,11	#LEEG!	71,83	#LEEG!	6,72	#LEEG!	1,2
12,36	3,08	68,00	76,06	74,25	74,57	6,25	1,49	1,1
0,53	1,68	66,76	69,19	63,01	67,18	3,75	2,01	1,0
3,17	5,74	78,27	74,99	77,36	76,41	0,91	1,42	1,1
6,94	7,46	73,52	73,04	76,26	75,76	2,74	2,72	1,1
0,59	5,64	75,95	79,93	78,02	78,50	2,07	1,43	1,2
7,50	4,48	83,93	76,37	80,48	75,31	3,45	1,06	1,1
4,74	5,38	68,42	68,68	67,36	68,04	1,07	0,65	1,1
0,74	0,68	70,24	76,34	69,57	73,38	0,67	2,96	1,1
1,04	2,53	64,72	72,19	65,63	70,24	0,91	1,95	1,0
6,75	4,57	78,99	74,50	84,75	81,57	5,76	7,07	1,0
1,76	8,45	71,91	69,09	72,54	73,24	0,63	4,15	1,0
1,08	1,14	76,48	84,09	76,00	80,72	0,48	3,37	0,9
#LEEG!	#LEEG!	#LEEG!	#LEEG!	#LEEG!	#LEEG!	#LEEG!	#LEEG!	#LEEG!
0,94	0,67	79,61	79,73	78,92	82,27	0,69	2,54	1,2
5,46	6,19	65,52	62,33	67,98	60,97	2,45	1,36	1,2
#LEEG!	#LEEG!	#LEEG!	#LEEG!	#LEEG!	#LEEG!	#LEEG!	#LEEG!	#LEEG!

5,65	1,56	71,33	77,89	73,96	78,27	2,63	0,39	1,2
1,98	2,26	67,98	66,07	66,89	64,00	1,10	2,07	1,2
2,64	6,00	77,40	82,48	77,14	79,45	0,26	3,03	1,0
0,63	0,06	75,35	74,99	73,78	72,91	1,57	2,08	1,0
1,25	0,41	72,41	69,68	72,06	68,13	0,35	1,55	1,0
6,80	2,08	81,70	80,22	81,21	80,32	0,49	0,10	1,1
1,30	0,62	79,68	80,23	79,00	78,45	0,69	1,78	1,0
0,11	#LEEG!	65,76	#LEEG!	76,12	#LEEG!	10,36	#LEEG!	1,1
4,50	1,68	77,01	79,85	77,14	78,35	0,13	1,51	1,0
4,77	3,87	78,70	85,94	73,97	81,08	4,73	4,87	1,1
2,91	0,86	75,96	81,78	74,15	81,39	1,81	0,39	1,3
0,38	4,02	83,22	80,93	77,10	75,02	6,12	5,91	1,3
7,29	3,45	83,50	83,03	80,05	79,49	3,45	3,55	1,0
5,86	2,57	75,77	71,64	75,34	69,33	0,43	2,32	1,0
3,82	2,63	73,78	74,05	76,77	77,70	2,98	3,65	1,0
2,92	5,95	77,25	77,34	81,38	78,19	4,13	0,85	1,1
2,93	4,48	75,65	76,22	73,12	74,44	2,53	1,78	1,0
9,73	1,26	68,47	79,73	74,11	80,79	5,64	1,06	1,0
8,15	0,97	76,62	74,93	72,23	73,24	4,39	1,69	1,1
2,57	3,22	70,26	69,29	75,05	67,58	4,79	1,72	1,0
5,03	5,90	77,54	76,05	79,43	72,48	1,90	3,57	1,0
0,19	5,03	81,42	81,66	80,54	77,17	0,88	4,49	1,0
1,74	#LEEG!	77,23	#LEEG!	76,16	#LEEG!	1,07	#LEEG!	1,1
0,91	4,48	81,34	79,98	80,12	82,43	1,21	2,45	1,1
2,35	3,52	79,56	79,11	77,15	80,15	2,40	1,05	1,1
7,81	3,93	80,63	75,78	73,29	79,15	7,33	3,37	1,0
1,83	1,22	54,16	75,39	56,25	73,75	2,09	1,65	1,2
1,28	1,55	67,29	75,85	66,40	76,14	0,89	0,30	1,1
6,13	3,12	60,02	70,49	62,19	68,83	2,18	1,66	1,2
3,81	13,46	71,13	68,34	70,71	75,59	0,42	7,25	1,1
0,44	1,82	70,47	66,44	69,32	68,07	1,15	1,63	1,1
0,04	4,42	79,49	76,91	78,94	80,47	0,54	3,57	1,0
3,79	2,84	62,12	72,29	62,13	69,79	0,01	2,50	1,1
0,05	0,73	79,80	79,41	80,28	76,44	0,47	2,97	1,1
2,74	1,75	75,43	66,00	73,46	67,11	1,96	1,11	0,9
1,06	5,78	75,30	73,94	74,67	77,05	0,64	3,11	1,0
2,32	2,98	71,36	74,47	73,73	72,34	2,38	2,13	0,9
2,23	6,28	66,08	71,35	64,93	71,94	1,15	0,59	0,9
2,78	0,10	78,14	77,47	75,08	77,86	3,06	0,39	1,0
8,38	#LEEG!	80,40	#LEEG!	82,46	#LEEG!	2,06	#LEEG!	1,2
0,88	8,05	80,98	81,21	78,35	79,40	2,63	1,82	1,1
9,20	1,73	67,18	68,62	69,69	69,65	2,51	1,03	1,0
4,04	3,22	71,50	69,68	70,84	70,04	0,65	0,36	1,0
1,76	1,24	75,29	69,40	77,05	65,99	1,76	3,41	1,0
1,63	2,20	73,64	78,67	74,37	75,66	0,72	3,02	1,1
5,15	26,90	68,49	64,86	69,87	69,87	1,39	5,01	1,1
11,22	16,36	72,48	75,84	68,82	68,88	3,66	6,96	1,3
8,55	2,53	79,02	84,87	83,57	85,39	4,55	0,53	1,1
11,84	8,64	57,11	63,35	63,75	68,97	6,64	5,62	1,1
8,63	0,53	68,85	71,60	72,42	69,34	3,57	2,27	1,1

0,38	1,16	71,74	78,75	63,76	79,39	7,99	0,63	1,1
4,10	5,36	80,02	84,60	85,95	86,18	5,92	1,58	1,1
5,86	0,34	69,90	70,66	71,26	69,53	1,36	1,13	0,9
5,25	0,29	68,85	67,82	74,17	73,12	5,33	5,30	1,1
12,48	8,64	52,24	63,14	60,27	64,79	8,03	1,65	1,0
6,31	0,39	74,62	76,61	69,92	77,32	4,70	0,71	1,2
5,42	0,16	73,49	80,06	74,54	82,49	1,05	2,43	1,1
5,40	0,42	89,26	85,66	84,80	84,02	4,46	1,64	1,1
0,82	1,94	75,19	71,13	73,87	69,73	1,32	1,41	1,1
#LEEG!	#LEEG!	#LEEG!	#LEEG!	#LEEG!	#LEEG!	#LEEG!	#LEEG!	#LEEG!
3,61	7,61	69,74	67,50	69,48	68,85	0,26	1,35	1,2
8,05	18,64	63,96	59,41	70,26	72,38	6,30	12,98	1,2
#LEEG!	#LEEG!	#LEEG!	#LEEG!	#LEEG!	#LEEG!	#LEEG!	#LEEG!	#LEEG!
6,13	7,61	75,52	80,21	76,51	80,69	0,99	0,48	1,1
5,49	8,87	64,77	75,74	70,37	75,40	5,60	0,34	1,3
4,51	0,61	71,94	74,91	76,13	79,47	4,18	4,56	1,2
1,71	1,46	67,77	72,72	71,92	75,89	4,15	3,18	1,2
3,17	0,21	74,97	67,10	70,13	66,02	4,84	1,08	1,1
11,68	7,83	61,74	63,89	70,59	65,86	8,85	1,96	1,1
0,45	4,63	62,52	66,58	66,78	63,39	4,25	3,19	1,0
1,06	2,40	65,17	70,43	64,83	71,26	0,34	0,83	1,1
4,14	16,69	90,73	86,23	92,42	89,19	1,69	2,96	1,1
4,56	2,85	75,72	71,05	73,31	70,33	2,41	0,72	1,0
0,97	2,45	60,92	62,81	62,86	67,97	1,94	5,16	1,1
4,48	1,21	76,31	75,05	76,60	78,11	0,28	3,06	1,0
3,66	3,86	68,06	67,84	70,34	71,29	2,29	3,45	1,0
1,63	#LEEG!	70,10	#LEEG!	68,47	#LEEG!	1,63	#LEEG!	1,0
0,70	7,59	71,98	74,63	70,38	79,59	1,60	4,96	1,2
11,56	#LEEG!	71,63	#LEEG!	75,00	#LEEG!	3,37	#LEEG!	1,0
2,18	6,92	75,41	74,35	72,32	74,18	3,09	0,17	1,2
2,48	1,77	74,96	71,91	76,47	71,18	1,51	0,73	1,2
0,41	1,66	80,94	82,83	81,50	82,24	0,56	0,59	1,0
7,85	9,29	77,01	75,64	81,59	80,43	4,58	4,79	1,2
1,80	4,31	72,05	74,54	72,25	75,62	0,20	1,08	1,0
0,16	5,17	75,21	76,51	79,18	77,44	3,97	0,93	1,2
2,62	2,34	79,73	73,01	76,90	74,73	2,83	1,72	1,0
5,15	7,80	65,45	62,90	69,02	64,35	3,57	1,44	1,1
2,03	#LEEG!	78,47	#LEEG!	80,22	#LEEG!	1,75	#LEEG!	1,0
0,67	6,29	76,52	71,57	74,53	70,89	1,98	0,68	1,1
2,61	1,76	66,90	75,18	72,05	77,20	5,15	2,02	1,2
7,74	3,78	73,46	69,53	67,86	69,94	5,61	0,41	0,9
6,36	0,73	71,01	72,97	67,72	70,90	3,29	2,06	1,1
2,49	11,78	69,15	71,92	69,99	67,52	0,84	4,40	1,1
#LEEG!	#LEEG!	#LEEG!	#LEEG!	#LEEG!	#LEEG!	#LEEG!	#LEEG!	#LEEG!
4,71	11,38	67,55	65,73	70,26	67,36	2,70	1,63	1,1
4,45	1,08	79,36	81,67	80,91	83,49	1,55	1,82	1,0
#LEEG!	#LEEG!	#LEEG!	#LEEG!	#LEEG!	#LEEG!	#LEEG!	#LEEG!	#LEEG!
2,93	2,55	73,27	73,70	74,20	72,68	0,93	1,01	0,9
#LEEG!	#LEEG!	#LEEG!	#LEEG!	#LEEG!	#LEEG!	#LEEG!	#LEEG!	#LEEG!
2,12	2,77	77,73	80,38	80,01	79,39	2,28	0,99	1,0

6,24	2,94	80,85	87,91	79,88	88,52	0,97	0,62	1,1
8,20	3,89	79,95	81,10	84,28	81,30	4,34	0,20	1,0
2,87	2,53	73,91	70,76	78,46	71,92	4,54	1,15	1,2
0,34	5,32	70,38	76,32	75,11	77,03	4,72	0,71	1,1
0,39	1,71	66,43	71,63	64,75	73,63	1,67	2,00	1,2
0,91	4,88	71,83	72,93	67,25	68,61	4,57	4,32	1,0
5,44	1,68	67,74	71,11	66,44	67,99	1,30	3,12	1,1
4,44	7,30	75,20	75,45	73,07	79,16	2,13	3,71	1,1
1,39	5,10	79,20	83,14	79,23	83,93	0,03	0,79	1,1
6,20	0,31	72,38	73,36	68,53	71,59	3,85	1,76	1,0
0,72	0,71	75,33	76,13	75,97	75,63	0,64	0,50	1,0
8,46	0,31	75,51	72,50	75,91	73,34	0,40	0,84	1,1
4,48	4,18	78,05	71,41	78,54	66,07	0,48	5,34	1,0
0,25	1,69	68,98	71,45	67,15	69,73	1,83	1,71	1,0
4,59	#LEEG!	72,31	#LEEG!	75,37	#LEEG!	3,05	#LEEG!	1,1
7,23	2,59	76,37	75,22	72,50	73,88	3,87	1,34	1,0
8,68	10,99	64,92	62,36	66,35	66,22	1,43	3,86	1,1
0,02	2,19	73,82	72,51	76,37	71,57	2,55	0,94	0,9
0,92	5,83	70,42	73,45	71,17	75,84	0,75	2,39	1,0
5,99	1,56	63,11	61,93	64,00	61,06	0,89	0,88	1,0
3,09	6,13	73,68	77,84	73,00	81,38	0,68	3,54	1,0
6,61	5,56	66,47	66,59	64,04	67,36	2,43	0,77	1,4
0,94	1,76	75,06	82,57	75,29	83,64	0,23	1,07	1,3
2,65	1,38	77,54	79,82	78,64	76,81	1,09	3,01	1,0
3,15	1,11	69,25	73,62	66,97	67,23	2,28	6,39	1,0
0,59	0,32	69,68	68,39	67,85	68,68	1,83	0,29	1,1
1,17	3,43	77,41	75,76	78,19	75,37	0,78	0,39	1,0
4,26	6,00	75,09	68,86	64,85	66,12	10,24	2,74	1,3
1,44	9,39	69,25	69,31	67,77	70,95	1,48	1,64	0,9
1,92	2,54	72,57	74,85	71,66	75,56	0,91	0,71	1,1
1,56	0,00	65,67	66,90	67,22	69,89	1,55	2,99	1,1
5,46	7,14	70,81	68,66	70,44	70,34	0,37	1,68	0,9
0,45	2,50	72,77	71,36	72,84	72,25	0,07	0,89	1,1
4,73	2,46	77,90	72,94	73,90	71,83	4,00	1,11	1,0
8,90	#LEEG!	73,18	#LEEG!	74,57	#LEEG!	1,39	#LEEG!	1,1
4,00	4,41	73,25	79,63	74,38	80,70	1,14	1,07	1,1
0,89	2,81	71,65	68,68	68,01	64,13	3,64	4,56	1,1
3,21	2,25	74,31	76,99	78,52	80,19	4,22	3,20	1,2
0,69	10,72	59,76	56,43	61,87	56,66	2,11	0,23	1,1
4,67	4,82	76,68	76,29	73,53	75,94	3,15	0,35	1,2
5,28	6,24	75,51	75,52	78,23	74,09	2,72	1,44	1,1
4,79	3,28	73,75	76,39	72,59	75,50	1,16	0,90	1,1
2,46	0,11	73,36	72,50	74,03	75,09	0,67	2,59	1,0
7,96	#LEEG!	81,01	#LEEG!	84,12	#LEEG!	3,11	#LEEG!	1,1
2,65	5,84	76,45	73,07	81,23	77,43	4,78	4,36	1,3
1,18	#LEEG!	69,30	#LEEG!	66,98	#LEEG!	2,32	#LEEG!	1,5
3,16	7,17	77,61	79,34	77,14	76,02	0,47	3,32	1,3
1,81	0,51	76,24	73,07	76,00	73,83	0,25	0,77	1,0
6,04	10,58	75,93	73,47	78,88	77,58	2,95	4,11	1,0
2,79	7,32	83,35	74,99	80,68	72,75	2,67	2,24	1,2

8,78	#LEEG!	71,81	#LEEG!	81,76	#LEEG!	9,95	#LEEG!	1,1
0,32	4,08	70,14	70,62	71,09	68,46	0,94	2,16	1,3
0,06	3,94	73,15	71,76	70,78	75,12	2,37	3,36	1,0
5,53	4,43	67,57	70,87	69,74	73,63	2,17	2,75	1,2
6,58	12,81	50,96	44,97	59,14	51,57	8,18	6,61	1,3
3,51	5,80	72,47	72,37	77,39	71,31	4,92	1,06	1,0
5,01	5,35	73,57	70,49	71,52	69,12	2,04	1,37	1,4
5,20	7,21	74,38	60,12	74,39	52,90	0,01	7,23	1,4
4,03	4,81	80,99	70,11	79,10	71,30	1,90	1,20	1,2
1,74	8,55	72,29	64,76	70,22	71,10	2,06	6,34	1,1
9,32	5,98	68,60	71,78	68,65	73,61	0,05	1,83	1,2
1,09	#LEEG!	79,87	#LEEG!	80,63	#LEEG!	0,76	#LEEG!	1,1
5,33	0,72	72,37	63,72	71,99	68,40	0,38	4,68	1,2
6,36	4,00	67,53	73,02	67,56	74,28	0,03	1,26	1,3
3,52	2,69	70,21	71,21	65,69	71,82	4,52	0,61	1,0
16,19	4,62	67,45	58,13	70,58	62,86	3,13	4,73	1,1
14,58	9,06	65,33	69,98	71,21	74,38	5,88	4,40	1,4
1,02	1,88	71,48	70,09	73,48	66,41	2,00	3,68	1,1
8,21	5,43	66,83	52,99	60,35	57,73	6,48	4,74	1,3
1,56	2,07	70,22	63,68	70,67	62,41	0,44	1,27	1,1
6,41	6,56	61,14	55,80	58,78	68,79	2,35	13,00	1,1
8,24	4,08	73,77	72,59	69,42	70,65	4,35	1,93	1,0
11,32	2,68	72,67	67,31	68,71	63,77	3,96	3,54	1,2
5,67	2,20	62,91	61,99	60,54	63,80	2,37	1,81	1,2
1,01	1,95	66,66	60,47	62,24	60,23	4,42	0,24	1,5
13,20	1,53	59,45	62,97	67,79	61,24	8,35	1,74	1,1
11,43	3,49	63,51	70,09	71,23	69,66	7,72	0,44	1,2
6,36	#LEEG!	62,47	#LEEG!	64,60	#LEEG!	2,13	#LEEG!	1,2
3,83	2,20	63,89	67,33	65,00	67,41	1,11	0,09	1,1
6,58	#LEEG!	65,72	#LEEG!	68,17	#LEEG!	2,45	#LEEG!	1,1
2,12	1,31	74,65	77,58	76,57	76,80	1,92	0,77	1,1
7,65	6,16	64,35	69,02	67,65	69,00	3,30	0,01	1,3
0,19	14,68	66,48	63,04	67,82	63,01	1,34	0,03	1,4
0,34	2,81	65,27	73,57	65,64	70,17	0,36	3,40	1,2
1,35	25,31	73,94	56,39	76,24	34,63	2,31	21,75	1,0
0,03	2,91	74,03	78,29	76,14	75,68	2,11	2,61	1,3
1,44	4,82	57,53	65,69	62,12	65,05	4,59	0,64	1,1
2,33	19,51	53,10	51,61	50,81	52,61	2,29	1,00	1,2
6,50	0,57	62,75	71,60	64,95	68,12	2,20	3,48	1,2
2,51	0,68	63,61	63,51	62,10	63,39	1,51	0,12	1,3
0,91	0,03	62,21	72,69	67,15	72,00	4,93	0,69	1,2
4,71	3,75	75,20	71,17	72,90	65,40	2,30	5,77	1,2
1,70	7,37	69,89	67,88	68,30	70,21	1,59	2,33	1,1
5,25	#LEEG!	71,93	#LEEG!	72,97	#LEEG!	1,03	#LEEG!	1,0
5,25	1,14	60,05	64,30	58,67	59,50	1,38	4,80	1,2
4,41	20,01	68,75	50,86	70,43	67,30	1,68	16,43	1,0
7,73	6,85	70,65	67,98	73,64	71,06	3,00	3,07	1,1
1,02	1,29	69,00	65,75	67,62	69,71	1,38	3,96	1,5

M6_GS_Av	BL_GS_Sta	M6_GS_Sta	BL_GS_Sta	M6_GS_Sta	BL_GS_Diff	M6_GS_Dif	BL_GS_Ran	M6_GS_Ra
1,1	4,67	9,74	5,78	6,32	1,11	3,42	14,57	17,56
1,1	15,06	9,90	12,43	11,89	2,63	1,99	21,72	20,10
1,2	20,07	18,56	21,53	16,73	1,46	1,84	49,04	50,93
#LEEG!	#LEEG!	#LEEG!	#LEEG!	#LEEG!	#LEEG!	#LEEG!	#LEEG!	#LEEG!
1,1	22,64	19,10	21,95	18,05	0,69	1,05	41,25	28,81
#LEEG!	#LEEG!	#LEEG!	#LEEG!	#LEEG!	#LEEG!	#LEEG!	#LEEG!	#LEEG!
1,1	18,39	17,31	12,00	14,27	6,39	3,04	33,43	28,77
1,1	14,56	10,22	9,62	10,49	4,95	0,27	42,23	33,05
#LEEG!	#LEEG!	#LEEG!	#LEEG!	#LEEG!	#LEEG!	#LEEG!	#LEEG!	#LEEG!
#LEEG!	#LEEG!	#LEEG!	#LEEG!	#LEEG!	#LEEG!	#LEEG!	#LEEG!	#LEEG!
1,0	12,42	14,57	11,53	11,67	0,88	2,91	22,89	24,85
1,2	17,98	17,50	13,90	13,96	4,08	3,54	34,76	22,40
1,2	13,24	9,73	13,37	11,78	0,13	2,06	28,23	26,56
1,0	12,30	9,25	11,24	12,59	1,05	3,34	17,80	27,65
#LEEG!	20,07	#LEEG!	22,07	#LEEG!	2,00	#LEEG!	48,99	#LEEG!
1,1	4,99	5,85	7,95	7,02	2,96	1,17	32,26	38,28
#LEEG!	#LEEG!	#LEEG!	#LEEG!	#LEEG!	#LEEG!	#LEEG!	#LEEG!	#LEEG!
1,2	10,44	12,12	17,92	11,98	7,49	0,14	25,91	15,11
#LEEG!	#LEEG!	#LEEG!	#LEEG!	#LEEG!	#LEEG!	#LEEG!	#LEEG!	#LEEG!
1,2	10,77	10,68	14,60	15,92	3,83	5,24	35,44	33,80
1,0	17,41	16,37	12,17	12,65	5,24	3,72	32,18	34,73
#LEEG!	19,75	#LEEG!	15,75	#LEEG!	4,00	#LEEG!	29,37	#LEEG!
1,1	22,07	17,78	21,38	18,97	0,69	1,19	24,88	27,01
1,0	18,65	19,10	19,05	15,71	0,40	3,38	34,62	33,38
1,1	16,13	18,85	14,46	17,78	1,68	1,07	27,01	37,88
1,1	14,39	11,66	20,57	14,93	6,18	3,27	29,98	20,81
1,1	14,67	10,36	22,67	20,22	7,99	9,87	27,02	24,33
1,0	15,73	18,41	22,08	23,78	6,36	5,37	35,70	44,23
1,3	15,55	11,96	18,75	10,33	3,20	1,64	29,56	25,98
1,1	12,80	18,80	15,32	19,55	2,53	0,75	35,67	45,05
1,1	14,90	18,89	16,64	24,56	1,75	5,67	32,61	40,94
1,2	7,78	4,42	9,88	12,61	2,10	8,19	35,04	45,52
1,1	15,79	19,17	16,99	16,07	1,20	3,11	31,71	36,96
1,2	23,36	22,17	23,20	21,40	0,16	0,78	43,27	32,49
1,0	11,22	21,40	10,22	21,62	1,00	0,22	18,50	35,19
1,1	11,49	14,63	11,98	15,15	0,49	0,51	28,92	36,16
1,0	17,11	15,56	16,88	22,96	0,24	7,40	40,34	41,71
1,0	13,49	16,02	15,87	18,99	2,38	2,98	26,91	41,89
#LEEG!	11,34	#LEEG!	7,29	#LEEG!	4,05	#LEEG!	23,84	#LEEG!
#LEEG!	22,34	#LEEG!	19,22	#LEEG!	3,13	#LEEG!	42,92	#LEEG!
1,0	21,26	24,49	17,21	17,85	4,05	6,65	30,18	31,70
1,0	15,99	19,05	15,81	17,05	0,18	2,00	28,42	33,80
1,0	10,34	15,54	15,47	15,70	5,13	0,16	34,56	34,49
1,1	14,29	11,15	16,59	13,57	2,30	2,42	42,58	40,51
1,0	18,31	21,18	13,84	16,06	4,47	5,12	39,84	30,62
1,0	22,89	23,80	24,90	27,65	2,01	3,85	37,03	33,92
1,1	4,00	6,25	11,66	14,12	7,67	7,87	15,40	44,03
1,1	17,53	19,25	18,86	19,42	1,33	0,17	40,25	43,33
1,0	9,77	17,26	11,88	15,19	2,11	2,08	31,48	30,46

1,0	15,27	19,35	18,18	16,60	2,91	2,75	27,95	30,94
1,0	17,28	13,77	18,64	12,80	1,36	0,97	42,24	39,50
1,1	15,71	14,18	10,22	14,98	5,49	0,80	31,22	33,41
1,1	13,59	19,00	22,51	23,39	8,92	4,40	26,92	28,04
1,2	20,37	26,12	24,86	19,98	4,49	6,14	37,36	26,38
1,0	19,28	19,34	21,72	19,85	2,44	0,51	58,58	38,32
0,9	12,67	19,15	12,90	19,07	0,23	0,08	24,26	30,88
1,1	23,30	19,86	11,81	18,35	11,49	1,51	36,66	39,15
1,2	22,03	19,48	21,20	20,31	0,83	0,83	37,38	26,94
1,0	13,17	18,12	13,55	16,20	0,38	1,92	43,01	42,96
1,0	24,11	24,71	20,03	21,05	4,08	3,65	37,95	36,60
1,1	17,79	13,20	15,14	10,88	2,65	2,32	41,23	31,30
1,2	15,71	10,53	16,70	13,78	0,99	3,25	36,58	24,01
1,2	24,59	22,24	25,69	21,11	1,10	1,13	40,84	23,64
1,1	9,98	12,46	16,56	13,76	6,58	1,30	34,11	33,99
1,0	19,83	15,92	14,50	17,72	5,32	1,80	30,16	25,58
1,1	16,70	17,97	19,50	16,31	2,80	1,67	45,01	39,03
1,2	19,00	12,24	14,71	9,77	4,29	2,47	34,06	23,08
1,0	16,18	15,94	17,78	19,32	1,60	3,38	36,64	45,72
1,1	7,93	10,53	16,27	19,71	8,34	9,19	33,72	28,03
1,2	21,18	25,64	14,88	16,30	6,30	9,34	36,57	36,01
1,1	11,11	21,91	9,01	17,20	2,10	4,71	32,67	35,39
1,1	14,10	19,19	20,49	21,43	6,39	2,24	29,53	32,87
1,1	18,60	20,51	24,02	23,74	5,42	3,23	30,42	53,42
1,4	23,26	16,84	25,74	18,44	2,47	1,61	41,19	21,64
1,0	18,62	15,19	22,75	19,03	4,14	3,85	25,46	33,36
1,0	16,77	17,91	23,06	24,16	6,29	6,25	32,64	28,65
#LEEG!	8,10	#LEEG!	12,40	#LEEG!	4,30	#LEEG!	27,44	#LEEG!
1,1	14,56	18,32	17,66	17,85	3,10	0,47	35,10	42,23
1,3	13,09	20,57	14,04	19,65	0,95	0,92	33,13	30,82
1,3	17,04	13,36	11,41	10,13	5,63	3,23	22,78	17,36
1,0	24,33	15,63	20,63	16,11	3,69	0,48	45,40	32,69
#LEEG!	12,77	#LEEG!	13,47	#LEEG!	0,71	#LEEG!	32,65	#LEEG!
#LEEG!	13,33	#LEEG!	10,25	#LEEG!	3,08	#LEEG!	26,17	#LEEG!
1,0	19,23	14,89	5,98	8,43	13,25	6,46	35,74	43,80
1,1	9,53	8,68	11,09	16,80	1,56	8,12	29,18	34,83
1,0	18,58	18,45	23,39	22,63	4,80	4,18	34,72	36,36
1,1	15,43	11,96	13,97	19,96	1,46	8,00	34,45	37,19
1,0	18,54	12,04	15,39	17,60	3,14	5,56	39,54	45,89
1,0	20,16	23,75	21,90	23,66	1,74	0,10	37,33	23,55
1,2	12,92	16,81	17,66	19,31	4,74	2,50	32,95	30,26
1,0	17,22	19,84	17,62	18,58	0,39	1,27	36,13	41,54
1,0	10,93	12,34	13,73	14,93	2,80	2,59	23,29	29,79
1,1	13,28	17,98	15,85	19,68	2,57	1,70	43,12	32,47
1,0	17,06	16,09	20,23	21,37	3,17	5,28	39,72	38,98
0,9	22,99	24,47	18,15	21,68	4,84	2,79	41,33	48,47
#LEEG!	#LEEG!	#LEEG!	#LEEG!	#LEEG!	#LEEG!	#LEEG!	#LEEG!	#LEEG!
1,1	23,16	16,36	16,23	18,89	6,94	2,53	30,64	41,40
1,3	4,82	8,17	22,39	25,98	17,57	17,82	30,76	14,56
#LEEG!	#LEEG!	#LEEG!	#LEEG!	#LEEG!	#LEEG!	#LEEG!	#LEEG!	#LEEG!

1,1	18,39	21,68	19,30	25,29	0,90	3,61	23,31	20,40
1,1	14,24	11,22	7,60	6,62	6,64	4,60	30,09	22,36
0,9	14,69	15,43	21,92	24,06	7,23	8,63	54,42	48,86
1,0	13,81	14,80	15,61	15,05	1,81	0,25	37,01	33,70
1,1	17,68	16,34	22,37	16,14	4,69	0,20	39,84	33,45
1,2	16,87	24,37	23,06	28,00	6,19	3,63	40,96	44,01
1,0	13,71	18,56	18,55	18,02	4,84	0,54	41,63	39,55
#LEEG!	16,94	#LEEG!	20,07	#LEEG!	3,12	#LEEG!	37,19	#LEEG!
1,0	18,86	18,84	23,10	22,55	4,25	3,71	44,22	39,59
1,0	25,93	30,25	18,05	22,54	7,88	7,71	44,12	38,96
1,2	21,51	25,87	18,89	23,53	2,62	2,34	33,81	38,26
1,2	17,98	17,32	22,68	22,69	4,69	5,38	34,61	26,52
1,0	22,64	23,14	21,94	18,58	0,70	4,56	44,02	50,38
1,1	17,03	14,35	11,53	10,45	5,50	3,90	25,15	27,59
1,0	21,60	20,30	22,17	20,07	0,56	0,24	35,53	26,26
1,1	20,16	15,87	17,09	15,83	3,08	0,04	40,49	29,29
1,0	16,36	13,54	16,03	13,11	0,34	0,43	38,14	42,88
1,0	15,83	19,52	17,03	22,33	1,20	2,81	32,98	25,63
1,0	15,90	18,07	13,56	16,25	2,35	1,82	34,63	33,29
1,1	20,48	20,15	20,60	20,77	0,12	0,61	38,05	26,64
0,9	17,28	19,03	18,98	24,31	1,71	5,28	34,90	43,18
1,1	19,38	24,84	18,95	16,57	0,43	8,26	33,83	32,25
#LEEG!	19,80	#LEEG!	16,22	#LEEG!	3,58	#LEEG!	32,17	#LEEG!
1,0	22,56	23,10	21,12	25,82	1,44	2,73	36,05	41,67
1,1	18,99	20,30	22,61	20,99	3,62	0,70	41,59	39,97
1,0	25,05	23,95	24,38	20,14	0,67	3,81	42,51	38,36
1,0	9,84	21,56	11,46	16,51	1,62	5,05	24,09	42,72
1,2	16,92	18,90	18,12	18,91	1,21	0,01	24,46	35,94
1,1	15,85	15,60	13,08	17,49	2,77	1,89	29,01	30,82
1,1	10,09	9,81	12,83	14,77	2,75	4,96	32,62	28,04
1,1	13,18	8,89	15,28	19,01	2,10	10,13	34,07	32,35
1,0	29,15	27,74	23,77	18,98	5,38	8,76	46,86	41,84
1,0	11,56	11,45	13,81	12,96	2,25	1,51	22,16	32,20
1,1	23,62	21,32	22,17	21,17	1,45	0,15	38,33	40,36
1,0	16,82	12,12	20,52	11,32	3,69	0,80	31,93	35,28
0,9	16,07	14,99	11,71	12,83	4,37	2,16	37,95	40,49
1,0	16,16	22,44	15,41	19,72	0,75	2,71	34,78	41,02
1,0	25,19	15,19	25,29	19,88	0,10	4,69	48,03	36,45
1,0	16,51	21,14	15,01	22,80	1,51	1,66	32,05	41,46
#LEEG!	20,94	#LEEG!	20,78	#LEEG!	0,16	#LEEG!	44,21	#LEEG!
1,1	19,78	19,39	19,68	22,90	0,10	3,52	30,60	36,62
1,0	7,74	12,24	12,33	12,84	4,59	0,60	42,36	31,69
1,0	16,78	17,09	20,80	18,78	4,02	1,70	39,74	39,16
1,1	18,28	16,81	15,02	14,32	3,27	2,49	33,95	25,86
1,1	15,90	15,92	13,87	19,20	2,03	3,28	38,36	40,54
1,1	12,17	7,15	13,41	21,40	1,24	14,25	23,92	35,38
1,1	15,30	15,91	12,70	11,43	2,60	4,48	33,84	39,78
1,1	20,49	20,11	23,31	23,79	2,82	3,67	38,05	51,18
1,1	12,83	17,70	21,44	23,99	8,61	6,29	24,71	31,22
1,1	10,86	9,32	15,14	13,50	4,27	4,18	29,72	18,14

1,1	13,87	20,50	13,16	24,34	0,71	3,84	29,25	29,69
1,1	21,60	20,49	27,19	28,69	5,59	8,20	44,15	46,36
0,9	18,94	17,63	23,73	19,08	4,79	1,46	46,21	33,89
1,1	12,19	13,08	18,03	14,32	5,84	1,24	30,18	31,74
1,0	6,97	5,54	11,67	7,90	4,71	2,36	25,24	33,54
1,1	20,20	20,35	18,01	18,79	2,19	1,56	27,96	28,15
1,0	14,31	17,95	13,15	17,47	1,16	0,48	42,33	34,72
1,0	26,20	22,29	33,33	25,74	7,13	3,45	53,96	52,52
1,0	11,02	5,53	4,36	4,49	6,67	1,04	30,34	25,55
#LEEG!	#LEEG!	#LEEG!	#LEEG!	#LEEG!	#LEEG!	#LEEG!	#LEEG!	#LEEG!
1,1	16,91	14,88	21,52	24,73	4,60	9,85	28,24	29,39
1,2	10,39	9,45	9,24	7,15	1,14	2,31	28,15	34,49
#LEEG!	#LEEG!	#LEEG!	#LEEG!	#LEEG!	#LEEG!	#LEEG!	#LEEG!	#LEEG!
1,0	20,41	17,50	21,67	18,58	1,26	1,09	27,32	43,30
1,2	9,45	12,15	12,63	28,49	3,18	16,34	29,28	33,13
1,2	15,14	18,50	15,79	15,68	0,64	2,82	29,58	27,77
1,2	17,77	14,45	15,19	18,42	2,58	3,97	26,03	31,81
1,0	14,83	15,17	22,42	13,76	7,58	1,41	40,10	36,98
1,0	15,45	15,14	20,88	22,00	5,42	6,86	26,49	31,37
1,0	7,30	9,88	8,82	6,13	1,52	3,75	26,49	36,87
1,0	12,68	11,71	13,66	15,49	0,98	3,78	21,80	35,99
1,2	23,58	22,43	22,09	23,79	1,49	1,37	52,48	38,16
1,1	29,28	19,62	27,37	21,02	1,91	1,40	29,81	33,89
1,0	10,06	11,02	10,91	12,38	0,85	1,35	25,17	28,66
1,0	16,83	14,11	11,84	12,60	5,00	1,51	44,69	49,38
1,0	12,02	12,46	15,18	16,17	3,16	3,71	34,22	41,41
#LEEG!	12,83	#LEEG!	14,67	#LEEG!	1,84	#LEEG!	23,45	#LEEG!
1,1	17,63	17,69	19,36	23,21	1,73	5,52	43,97	42,28
#LEEG!	12,12	#LEEG!	8,13	#LEEG!	3,99	#LEEG!	33,89	#LEEG!
1,1	16,06	14,10	13,73	13,26	2,33	0,84	#LEEG!	43,60
1,1	14,87	12,31	19,47	12,01	4,60	0,30	36,90	33,16
1,0	23,08	20,71	20,18	19,64	2,90	1,07	33,66	41,48
1,2	22,43	22,22	29,09	33,44	6,66	11,22	39,09	35,26
1,0	22,92	27,61	19,25	16,13	3,67	11,48	40,02	36,94
1,2	20,37	16,70	28,13	25,43	7,76	8,73	33,52	34,52
1,0	19,44	15,93	18,85	17,05	0,59	1,12	39,31	50,10
1,1	14,65	12,16	18,73	17,11	4,08	4,94	36,82	33,43
#LEEG!	22,23	#LEEG!	21,42	#LEEG!	0,81	#LEEG!	51,81	#LEEG!
1,0	22,15	24,26	22,88	18,98	0,72	5,28	42,51	38,08
1,1	17,56	22,71	20,09	19,47	2,53	3,24	31,28	40,34
1,1	16,66	13,91	9,18	10,67	7,48	3,24	31,86	29,02
1,1	23,98	18,86	20,91	19,89	3,07	1,03	28,35	30,72
1,1	14,02	17,34	13,11	13,19	0,91	4,15	25,29	28,47
#LEEG!	#LEEG!	#LEEG!	#LEEG!	#LEEG!	#LEEG!	#LEEG!	#LEEG!	#LEEG!
1,1	16,66	8,59	16,52	14,81	0,15	6,22	24,23	22,65
1,0	23,10	25,82	23,87	22,56	0,76	3,25	43,45	42,48
#LEEG!	#LEEG!	#LEEG!	#LEEG!	#LEEG!	#LEEG!	#LEEG!	#LEEG!	#LEEG!
0,9	17,73	14,51	19,78	20,12	2,06	5,61	32,63	31,91
#LEEG!	#LEEG!	#LEEG!	#LEEG!	#LEEG!	#LEEG!	#LEEG!	#LEEG!	#LEEG!
1,0	20,23	18,06	22,53	23,66	2,30	5,60	34,46	43,10

1,1	14,38	26,40	25,27	30,76	10,89	4,35	36,03	42,55
1,0	21,26	19,64	16,36	15,49	4,89	4,15	33,30	42,37
1,1	16,50	14,93	19,51	17,18	3,01	2,25	37,47	30,91
1,1	25,30	22,52	21,39	25,31	3,91	2,79	39,19	35,17
1,2	18,66	15,59	28,86	23,68	10,20	8,10	47,44	39,25
1,1	17,18	15,95	19,44	16,54	2,26	0,60	24,08	28,24
1,1	10,82	16,40	11,62	8,81	0,80	7,59	31,67	29,85
1,1	16,21	17,53	14,42	20,69	1,78	3,16	39,67	41,78
1,0	21,97	26,40	23,47	24,35	1,50	2,06	35,38	39,39
0,9	15,17	16,87	14,62	19,32	0,55	2,45	35,56	37,99
1,0	17,21	19,94	21,92	17,30	4,71	2,64	49,54	36,88
1,0	16,76	17,69	20,19	17,39	3,43	0,30	34,55	32,94
1,0	20,45	18,10	23,41	15,82	2,97	2,27	35,83	40,14
1,0	15,60	14,20	13,89	18,89	1,71	4,69	32,46	35,87
#LEEG!	14,16	#LEEG!	16,64	#LEEG!	2,48	#LEEG!	36,78	#LEEG!
1,0	15,45	18,17	14,35	14,14	1,10	4,03	48,56	43,87
1,1	10,96	9,67	16,71	20,10	5,74	10,42	33,20	25,87
1,1	21,83	20,56	18,03	22,87	3,81	2,30	37,12	43,81
1,0	17,12	15,60	22,29	19,55	5,17	3,95	35,18	36,95
1,1	12,27	11,02	17,00	15,52	4,73	4,50	31,67	33,00
1,0	16,54	28,97	19,31	25,48	2,77	3,49	33,29	39,67
1,3	15,21	14,30	14,76	14,66	0,45	0,36	20,40	27,86
1,2	19,25	23,83	19,13	21,23	0,12	2,60	31,94	40,80
0,9	21,36	22,83	23,45	22,09	2,09	0,75	40,59	41,71
1,0	9,89	12,94	12,88	11,18	2,99	1,77	37,52	36,11
1,1	16,30	19,89	19,58	14,55	3,27	5,34	29,95	21,45
1,1	17,31	12,27	19,83	19,83	2,52	7,57	39,00	33,42
1,3	13,06	8,95	18,37	18,33	5,31	9,38	32,24	33,39
1,3	13,28	9,72	19,91	18,44	6,63	8,72	31,91	40,01
1,1	17,60	19,37	22,46	25,72	4,86	6,35	34,71	31,86
1,1	13,61	18,83	15,02	14,39	1,41	4,44	34,23	37,64
1,0	8,26	13,27	21,98	23,27	13,72	10,00	36,01	45,59
1,1	15,89	15,40	18,90	17,09	3,02	1,69	34,44	29,23
1,0	20,54	17,92	20,35	17,25	0,18	0,67	26,16	24,35
#LEEG!	9,82	#LEEG!	15,61	#LEEG!	5,80	#LEEG!	36,20	#LEEG!
1,1	18,67	16,57	17,29	19,45	1,37	2,88	37,90	36,42
1,2	18,68	16,23	16,68	13,43	2,00	2,80	39,60	37,71
1,1	13,35	14,38	17,17	26,17	3,82	11,78	24,73	31,87
1,2	5,73	2,82	8,41	11,75	2,68	8,92	22,54	19,81
1,1	14,20	17,40	17,03	21,16	2,83	3,76	32,23	43,28
1,1	21,80	10,17	25,15	22,07	3,35	11,90	34,56	38,68
1,1	17,72	21,18	23,59	23,41	5,87	2,23	37,06	38,62
1,0	20,04	22,64	24,53	20,94	4,49	1,70	30,57	28,46
#LEEG!	18,52	#LEEG!	16,30	#LEEG!	2,22	#LEEG!	33,37	#LEEG!
1,2	19,06	17,18	19,41	20,99	0,36	3,80	41,24	38,88
#LEEG!	8,95	#LEEG!	8,07	#LEEG!	0,87	#LEEG!	38,48	#LEEG!
1,3	11,30	16,49	9,44	16,56	1,86	0,06	34,14	34,99
1,1	23,37	16,46	23,31	20,53	0,06	4,07	27,61	28,54
1,0	13,78	10,81	16,23	12,83	2,45	2,02	42,62	47,46
1,2	8,92	7,82	21,09	17,34	12,17	9,52	36,76	40,52

#LEEG!	20,91	#LEEG!	16,30	#LEEG!	4,60	#LEEG!	44,96	#LEEG!
1,1	10,55	13,80	12,86	15,83	2,31	2,03	29,98	30,94
1,0	15,89	17,27	14,54	19,89	1,35	2,62	25,75	29,89
1,0	10,82	16,99	17,40	22,03	6,58	5,04	33,66	39,01
1,1	9,31	9,07	8,65	8,08	0,67	0,99	15,89	8,41
1,1	14,62	15,24	14,10	13,82	0,52	1,43	28,59	36,81
1,2	1,98	4,62	12,76	12,36	10,78	7,74	26,35	30,87
1,2	13,50	10,90	3,29	7,96	10,21	2,94	26,09	28,34
1,1	18,32	13,79	22,49	16,30	4,17	2,51	39,21	41,83
1,1	11,08	18,02	9,00	13,39	2,08	4,62	24,22	14,48
1,2	9,47	15,92	8,05	20,51	1,42	4,60	29,76	39,57
#LEEG!	6,78	#LEEG!	18,41	#LEEG!	11,63	#LEEG!	30,82	#LEEG!
1,2	16,56	15,60	18,01	15,68	1,46	0,08	27,99	23,54
1,3	15,35	18,11	12,77	13,89	2,58	4,21	17,62	27,11
1,0	15,79	12,85	17,96	15,83	2,16	2,98	25,74	37,62
1,1	15,98	14,76	0,00	18,46	15,98	3,70	24,97	23,56
1,2	6,51	7,23	12,18	9,89	5,68	2,66	25,27	38,45
1,6	19,46	3,29	16,23	7,53	3,23	4,24	25,65	32,32
1,5	11,06	14,53	11,99	10,85	0,93	3,69	21,18	27,27
1,0	10,70	10,02	7,65	14,24	3,05	4,22	28,43	32,54
1,1	6,37	12,95	9,68	4,85	3,31	8,10	31,76	27,65
1,2	17,12	12,57	11,63	5,58	5,49	6,99	23,90	41,29
1,3	12,63	11,68	14,67	14,39	2,04	2,71	39,29	29,96
1,2	12,40	14,72	16,33	18,54	3,93	3,82	25,80	25,95
1,5	10,46	11,88	3,33	11,18	7,14	0,70	26,36	19,58
1,2	14,17	12,37	14,44	13,98	0,26	1,61	32,41	28,50
1,2	5,96	10,62	15,48	14,10	9,52	3,48	36,81	32,90
#LEEG!	11,21	#LEEG!	10,04	#LEEG!	1,17	#LEEG!	26,97	#LEEG!
1,2	11,26	10,04	13,45	9,64	2,19	0,39	23,42	32,36
#LEEG!	17,90	#LEEG!	19,57	#LEEG!	1,67	#LEEG!	28,03	#LEEG!
1,2	8,54	9,89	10,78	6,77	2,24	3,12	29,50	37,46
1,3	12,97	13,36	14,57	13,45	1,60	0,09	28,45	26,57
1,2	3,19	10,53	2,35	5,54	0,85	4,99	24,44	23,09
1,2	17,57	14,82	15,11	17,06	2,46	2,24	16,11	33,28
1,0	20,72	8,43	17,71	12,20	3,02	3,77	29,35	22,13
1,3	13,18	16,62	16,28	24,02	3,10	7,40	32,38	22,91
1,1	17,47	15,04	13,27	15,10	4,20	0,06	29,38	24,83
1,3	5,11	5,62	9,19	9,25	4,08	3,63	23,21	34,94
1,3	11,95	14,69	10,01	16,96	1,94	2,27	17,98	32,11
1,3	9,93	10,83	11,15	11,18	1,22	0,35	20,51	18,84
1,2	14,78	15,20	8,82	13,68	5,95	1,52	34,48	33,42
1,3	13,72	12,08	11,37	5,81	2,35	6,27	22,16	24,45
1,1	12,49	9,55	12,51	9,58	0,02	0,02	25,37	28,05
#LEEG!	9,82	#LEEG!	20,67	#LEEG!	10,85	#LEEG!	34,85	#LEEG!
1,2	12,81	13,99	9,63	14,47	3,17	0,48	27,51	35,50
1,1	14,77	12,45	15,50	13,18	0,73	0,72	28,53	24,50
1,0	20,44	13,44	14,99	14,45	5,45	1,01	38,40	35,47
1,3	10,75	12,27	17,27	18,33	6,52	6,06	29,99	30,52

BL_GS_Ran	M6_GS_Ra	BL_GS_Diff	M6_GS_Dif	BL_GS_Stri	M6_GS_Str	Change_Ch	Change_W	Change_KC
14,93	31,92	0,36	14,36	0,60	0,77	2	-5	17,65
22,08	15,83	0,36	4,28	0,78	0,79	1	2	27,94
45,08	36,18	3,96	14,75	0,58	1,24	-3	3	2,95
#LEEG!	#LEEG!	#LEEG!	#LEEG!	#LEEG!	#LEEG!	0	-3	2,99
39,00	34,12	2,26	5,30	1,17	1,00	-1	1	0,03
#LEEG!	#LEEG!	#LEEG!	#LEEG!	#LEEG!	#LEEG!	-1	3	2,91
36,13	34,14	2,70	5,37	0,42	0,88	1	6	0,03
46,08	40,25	3,85	7,20	0,41	1,09	2	-1	0,02
#LEEG!	#LEEG!	#LEEG!	#LEEG!	#LEEG!	#LEEG!	1	-1	11,81
#LEEG!	#LEEG!	#LEEG!	#LEEG!	#LEEG!	#LEEG!	0	14	20,54
18,57	19,33	4,33	5,52	0,77	0,35	0	-1	-1,48
26,11	33,87	8,66	11,47	1,02	0,42	2	4	-22,09
32,69	37,18	4,46	10,62	0,91	0,35	-1	-1	-13,24
25,29	35,16	7,49	7,51	0,74	0,40	0	0	-1,49
43,38	#LEEG!	5,60	#LEEG!	1,21	#LEEG!	#LEEG!	#LEEG!	#LEEG!
31,72	38,30	0,54	0,02	1,04	0,63	2	-10	10,30
#LEEG!	#LEEG!	#LEEG!	#LEEG!	#LEEG!	#LEEG!	-2	4	23,54
36,50	14,69	10,59	0,43	0,88	0,23	3	9	-8,79
#LEEG!	#LEEG!	#LEEG!	#LEEG!	#LEEG!	#LEEG!	1	1	38,25
25,09	28,17	10,36	5,63	0,96	0,48	2	-7	1,51
30,02	30,55	2,16	4,18	0,93	0,96	1	0	-8,80
26,78	#LEEG!	2,59	#LEEG!	0,78	#LEEG!	#LEEG!	#LEEG!	#LEEG!
24,28	27,66	0,60	0,66	0,92	0,92	1	-2	2,98
37,30	36,41	2,68	3,03	1,17	1,22	-3	-2	-14,69
30,77	36,27	3,76	1,61	1,02	1,14	0	-9	-5,87
26,42	18,37	3,56	2,44	0,89	0,61	-3	0	-5,92
29,77	28,05	2,75	3,72	0,88	0,82	0	-2	-2,89
31,92	40,30	3,78	3,93	1,02	1,28	0	-1	-19,14
27,98	25,73	1,58	0,25	1,23	0,90	-5	-2	2,97
39,73	43,37	4,06	1,68	1,08	1,27	2	0	-1,45
35,70	35,06	3,10	5,88	1,06	1,22	-2	-6	0,00
26,44	40,11	8,60	5,41	0,98	1,18	4	-9	2,93
20,91	18,94	10,80	18,02	0,92	0,95	6	-8	2,98
41,39	34,85	1,88	2,35	1,31	1,29	0	1	4,44
23,47	34,35	4,97	0,85	0,65	0,98	0	0	7,33
37,76	34,03	8,83	2,13	1,06	1,04	-2	-4	-23,57
35,73	33,29	4,61	8,43	1,30	1,26	-1	2	17,61
27,87	31,69	0,96	10,20	0,97	0,95	-1	-2	-2,93
25,01	#LEEG!	1,17	#LEEG!	0,69	#LEEG!	#LEEG!	#LEEG!	2,91
33,19	#LEEG!	9,73	#LEEG!	1,28	#LEEG!	#LEEG!	#LEEG!	#LEEG!
35,20	34,08	5,02	2,38	1,06	1,06	1	-3	-2,91
29,16	33,26	0,74	0,53	0,96	1,10	0	-4	-2,93
34,34	34,09	0,22	0,39	1,18	1,14	0	-4	16,21
44,40	43,93	1,82	3,42	1,30	1,22	0	2	-7,31
31,91	31,03	7,93	0,40	1,09	1,37	2	-4	-0,04
38,48	38,92	1,45	5,00	1,22	1,11	-5	2	-4,39
35,53	43,01	20,13	1,02	0,78	1,17	2	0	2,98
42,08	49,84	1,82	6,51	1,25	1,35	0	1	11,75
27,84	25,40	3,65	5,06	0,80	1,00	4	1	2,90

31,06	33,78	3,11	2,85	0,92	1,03	1	-1	10,26
36,01	36,80	6,23	2,70	1,22	1,25	-3	1	2,99
26,05	32,71	5,17	0,70	0,82	0,99	3	-1	8,82
29,43	29,40	2,51	1,36	1,05	1,20	1	0	-19,14
37,73	32,65	0,36	6,27	1,26	0,95	-1	6	-4,39
49,86	34,33	8,72	3,99	1,32	1,23	-2	2	4,40
22,56	33,12	1,70	2,24	0,71	1,03	-1	-4	-1,47
37,35	39,58	0,68	0,44	1,25	1,30	2	0	2,94
37,45	29,78	0,07	2,84	1,23	1,19	1	3	-7,32
25,76	30,15	17,25	12,81	0,97	1,03	4	-2	-2,95
43,18	40,89	5,22	4,29	1,20	1,33	3	3	-1,42
37,82	33,40	3,41	2,10	1,16	0,96	2	3	0,03
39,34	37,57	2,75	13,56	1,30	1,08	0	4	-14,71
48,71	22,90	7,87	0,74	1,38	1,22	1	-2	5,84
36,97	38,71	2,86	4,73	1,15	1,22	-1	3	-20,56
37,44	24,20	7,28	1,39	1,00	0,95	0	3	-8,82
33,15	38,38	11,86	0,65	1,24	1,14	-3	-1	-5,91
34,13	28,81	0,07	5,73	1,05	0,85	2	-2	-5,84
37,86	36,65	1,22	9,07	1,16	#LEEG!	2	-5	-16,21
34,42	34,33	0,70	6,30	1,10	1,04	0	0	-25,01
24,55	35,17	12,02	0,84	1,05	1,21	1	1	-16,15
32,77	39,87	0,11	4,48	0,99	1,15	0	-4	-14,71
32,83	33,33	3,30	0,47	0,99	1,10	3	-1	4,45
43,18	49,17	12,76	4,25	1,11	1,33	4	-1	5,85
37,72	17,95	3,47	3,69	1,20	0,35	4	22	1,45
32,80	38,51	7,33	5,14	0,89	1,00	2	-4	-5,86
36,00	30,33	3,36	1,68	1,04	1,09	0	0	1,44
22,71	#LEEG!	4,73	#LEEG!	0,72	#LEEG!	#LEEG!	#LEEG!	#LEEG!
39,51	34,93	4,41	7,30	1,13	1,19	2	0	-1,48
28,66	32,20	4,47	1,38	1,07	1,05	2	-5	4,37
26,89	23,26	4,11	5,89	0,75	0,79	0	2	-7,40
38,46	32,06	6,94	0,63	1,15	1,09	5	4	-1,47
30,56	#LEEG!	2,08	#LEEG!	1,00	#LEEG!	#LEEG!	#LEEG!	#LEEG!
32,20	#LEEG!	6,03	#LEEG!	1,04	#LEEG!	#LEEG!	#LEEG!	#LEEG!
32,70	41,54	3,04	2,26	1,14	1,42	-3	-1	0,02
26,36	37,19	2,82	2,37	1,05	1,13	0	2	4,42
38,70	41,11	3,99	4,75	1,18	1,19	-1	-3	8,85
36,77	44,11	2,33	6,92	1,09	1,17	-1	-2	16,19
39,82	42,40	0,28	3,48	1,27	1,46	5	-7	5,85
37,10	21,77	0,22	1,78	1,24	1,27	5	-3	-4,46
33,46	25,10	0,50	5,16	1,02	0,91	0	-1	2,94
40,82	34,39	4,69	7,15	1,27	1,24	1	-4	1,46
28,99	30,33	5,70	0,54	0,81	0,92	4	-1	-4,43
40,72	31,32	2,40	1,16	1,12	0,96	0	-1	-0,04
36,16	37,90	3,56	1,08	1,01	1,09	0	0	-24,96
44,90	46,42	3,57	2,05	1,19	1,26	-2	6	5,90
#LEEG!	#LEEG!	#LEEG!	#LEEG!	#LEEG!	#LEEG!	0	1	20,63
36,07	45,06	5,43	3,66	1,26	1,37	0	2	-2,94
33,73	12,18	2,96	2,38	1,12	1,04	#LEEG!	7	-0,04
#LEEG!	#LEEG!	#LEEG!	#LEEG!	#LEEG!	#LEEG!	3	3	1,50

30,18	27,47	6,87	7,08	1,12	1,09	0	5	-36,73
33,10	20,34	3,01	2,02	1,07	1,02	#LEEG!	3	0,01
49,00	52,01	5,42	3,15	1,32	1,36	0	-3	1,44
32,54	28,91	4,46	4,79	1,11	1,10	0	3	1,45
35,04	32,98	4,80	0,47	1,14	1,04	4	0	23,52
40,82	39,12	0,14	4,89	1,30	1,31	0	2	0,00
39,88	38,21	1,75	1,34	1,12	1,11	3	-2	10,29
38,89	#LEEG!	1,70	#LEEG!	1,12	#LEEG!	#LEEG!	#LEEG!	#LEEG!
42,53	41,36	1,69	1,77	1,32	1,27	0	4	1,44
37,89	36,62	6,23	2,34	1,16	1,20	2	3	0,00
35,37	40,62	1,55	2,36	1,09	1,34	1	1	16,19
30,78	21,21	3,84	5,31	1,13	1,06	-3	0	2,95
48,19	44,72	4,17	5,66	1,24	1,26	0	1	1,48
28,89	29,39	3,74	1,81	0,93	0,94	-1	0	-7,40
33,93	21,77	1,59	4,49	1,06	1,12	5	-4	8,79
40,48	26,23	0,01	3,06	1,29	1,22	-1	6	2,90
38,15	36,39	0,01	6,49	1,13	1,17	0	0	0,00
42,85	31,92	9,88	6,29	1,06	1,10	3	4	-5,92
30,72	34,14	3,91	0,84	1,09	1,10	0	4	5,86
37,32	27,64	0,74	1,00	1,10	1,01	7	-1	-2,91
32,44	51,78	2,46	8,60	1,09	1,22	-1	-1	-2,91
37,70	34,07	3,88	1,82	1,01	0,92	1	1	0,00
35,41	#LEEG!	3,24	#LEEG!	1,10	#LEEG!	2	-6	5,91
39,15	46,09	3,10	4,41	1,18	1,29	-2	-1	-1,47
33,82	45,74	7,77	5,77	1,17	1,16	-3	4	7,36
53,29	45,45	10,78	7,09	1,34	1,30	0	1	5,85
21,11	37,48	2,98	5,24	0,84	1,39	3	-3	14,66
22,85	33,84	1,62	2,10	1,12	1,08	0	-2	0,04
28,34	38,54	0,67	7,72	1,01	1,30	-2	2	-5,89
35,60	25,05	2,98	2,99	1,00	0,99	-1	5	-11,74
28,90	32,45	5,17	0,10	1,04	1,02	-1	1	-4,46
44,77	45,84	2,08	4,01	1,34	1,32	2	1	4,40
31,07	34,72	8,91	2,52	0,98	1,26	4	-6	-1,50
45,19	38,86	6,86	1,51	1,32	1,33	0	1	7,31
33,74	34,79	1,81	0,49	1,21	1,14	-2	2	14,68
38,91	42,36	0,96	1,87	1,18	1,23	-1	1	4,39
34,75	34,16	0,04	6,86	1,06	1,13	-1	3	-4,40
49,25	44,96	1,22	8,51	1,30	1,11	1	-5	-25,01
31,35	37,98	0,70	3,48	1,01	1,15	2	0	14,69
39,25	#LEEG!	4,96	#LEEG!	1,32	#LEEG!	#LEEG!	#LEEG!	#LEEG!
32,78	38,15	2,18	1,53	1,23	1,31	3	-3	0,00
35,15	24,97	7,21	6,72	1,18	1,09	-1	5	-16,19
40,07	42,09	0,34	2,92	1,10	1,15	-1	2	1,44
40,55	35,16	6,59	9,30	1,05	0,86	0	-2	5,88
40,38	37,35	2,02	3,19	1,19	1,24	#LEEG!	1	1,50
25,23	27,70	1,31	7,69	1,15	1,15	5	-1	-22,09
37,13	37,95	3,29	1,84	1,13	1,26	-3	0	7,35
32,21	46,67	5,84	4,50	1,22	1,50	0	-1	20,60
22,57	31,02	2,15	0,20	0,80	1,05	0	-6	14,72
37,18	21,75	7,47	3,61	0,95	0,91	-2	2	7,35

28,12	28,84	1,13	0,85	0,89	1,11	2	0	4,39
47,99	53,41	3,84	7,04	1,37	1,48	1	-1	#LEEG!
45,88	34,03	0,33	0,13	1,35	1,13	1	4	-0,01
33,30	37,51	3,12	5,77	1,05	1,14	2	1	#LEEG!
26,98	27,26	1,74	6,28	0,72	0,88	-1	18	45,56
25,28	35,96	2,68	7,81	0,98	1,05	2	-3	-22,01
44,30	42,64	1,98	7,92	1,26	0,53	4	2	-1,49
55,27	55,70	1,32	3,18	1,40	1,37	3	-2	-2,98
33,65	28,80	3,31	3,25	1,00	0,89	-3	4	-26,45
#LEEG!	#LEEG!	#LEEG!	#LEEG!	#LEEG!	#LEEG!	0	4	1,43
35,43	33,60	7,19	4,21	0,95	0,95	2	-6	-5,91
32,91	44,35	4,76	9,86	0,86	1,12	-2	0	11,78
#LEEG!	#LEEG!	#LEEG!	#LEEG!	#LEEG!	#LEEG!	0	0	13,26
25,62	37,59	1,70	5,71	1,00	0,44	2	4	8,79
29,02	42,50	0,26	9,38	1,06	1,47	1	-2	11,76
29,46	27,33	0,12	0,44	1,08	0,66	5	0	11,79
28,90	28,06	2,87	3,75	0,87	#LEEG!	-3	10	0,03
36,24	33,55	3,86	3,43	1,31	0,58	3	3	0,00
22,20	31,63	4,30	0,25	1,02	0,40	-1	1	11,73
28,59	35,17	2,10	1,69	0,99	0,55	0	-5	7,34
22,45	31,12	0,65	4,88	0,89	0,54	-1	-6	13,25
44,94	24,83	7,54	13,33	1,51	0,88	-2	4	-8,85
29,56	28,88	0,25	5,01	1,38	0,57	-1	3	-23,51
31,60	40,87	6,43	12,21	0,86	0,42	-2	1	23,49
36,15	49,25	8,55	0,13	1,25	1,40	-12	-2	22,09
37,76	44,38	3,54	2,96	1,09	1,27	1	-10	-8,81
25,75	#LEEG!	2,30	#LEEG!	0,88	#LEEG!	#LEEG!	#LEEG!	#LEEG!
41,93	38,54	2,04	3,74	1,25	1,19	0	21	8,86
36,27	#LEEG!	2,38	#LEEG!	1,16	#LEEG!	0	3	#LEEG!
#LEEG!	33,74	#LEEG!	9,86	0,90	1,05	-2	17	4,40
38,70	37,64	1,80	4,49	1,20	0,52	0	-1	-11,77
44,96	49,82	11,30	8,34	1,14	0,45	1	3	2,91
39,96	36,87	0,87	1,60	1,31	1,24	-1	3	27,95
37,73	43,14	2,29	6,20	1,27	1,21	2	1	2,93
37,53	39,50	4,01	4,99	1,17	1,24	-4	-2	-29,41
39,10	40,76	0,21	9,34	1,13	1,26	3	4	-1,43
31,31	28,28	5,51	5,15	1,05	0,96	3	-2	-1,51
48,01	#LEEG!	3,79	#LEEG!	1,52	#LEEG!	#LEEG!	#LEEG!	#LEEG!
39,13	31,95	3,38	6,13	1,20	1,03	-4	-13	0,00
38,88	35,65	7,60	4,70	1,13	0,49	-4	1	36,74
31,82	31,99	0,04	2,97	0,93	0,34	-8	13	7,32
28,64	27,89	0,28	2,83	1,09	1,10	1	2	1,48
27,45	29,55	2,17	1,08	0,93	0,94	0	-1	-8,85
#LEEG!	#LEEG!	#LEEG!	#LEEG!	#LEEG!	#LEEG!	1	3	-5,86
25,98	22,63	1,75	0,02	0,91	0,88	1	7	-20,61
46,55	41,28	3,10	1,21	1,20	1,25	1	-7	5,84
#LEEG!	#LEEG!	#LEEG!	#LEEG!	#LEEG!	#LEEG!	-1	-9	-33,78
30,04	28,89	2,59	3,02	0,92	0,42	1	-5	13,27
#LEEG!	#LEEG!	#LEEG!	#LEEG!	#LEEG!	#LEEG!	2	-15	-24,99
37,59	37,23	3,13	5,87	1,18	1,21	2	-1	-4,44

38,98	46,74	2,95	4,19	1,07	0,47	0	-2	-10,32
40,71	45,36	7,41	2,99	1,12	0,46	0	-3	1,51
38,77	33,29	1,30	2,38	1,30	0,76	1	0	-22,08
44,98	47,80	5,79	12,63	1,23	1,36	0	-3	-8,81
46,75	42,79	0,68	3,55	1,37	1,27	2	-1	-1,46
24,37	22,34	0,29	5,91	0,78	0,83	1	-3	5,91
30,32	32,94	1,35	3,10	1,04	1,10	0	-1	17,65
37,01	42,86	2,66	1,08	1,02	0,36	0	-2	7,31
31,72	40,90	3,67	1,51	1,26	0,74	7	-1	7,35
29,85	35,21	5,70	2,78	1,10	1,10	-2	2	-2,89
45,82	44,79	3,72	7,91	1,28	1,29	-2	0	2,92
31,13	35,18	3,42	2,24	1,06	0,49	1	-2	10,30
41,70	35,74	5,87	4,40	1,18	1,07	1	1	-17,68
31,67	29,32	0,78	6,55	0,92	0,95	-1	3	-2,95
39,63	#LEEG!	2,85	#LEEG!	1,09	#LEEG!	-3	-1	-4,45
44,90	46,77	3,66	2,90	1,32	1,38	1	-6	-0,02
35,52	33,78	2,32	7,91	0,97	0,89	-1	1	-11,80
40,02	34,78	2,90	9,03	1,12	1,09	1	4	2,95
42,40	33,44	7,22	3,50	1,08	0,48	3	-2	-1,48
24,44	26,74	7,22	6,26	1,00	1,03	-1	1	-13,21
38,23	45,49	4,93	5,82	0,50	0,56	-2	-1	-4,41
22,03	28,37	1,63	0,51	0,75	0,48	3	5	-1,49
30,17	43,03	1,78	2,24	1,07	0,80	3	-1	-5,90
38,38	33,69	2,22	8,02	0,50	0,50	2	0	1,48
33,46	35,32	4,06	0,79	0,97	1,02	2	0	-5,85
32,40	24,54	2,45	3,09	0,96	0,89	-2	3	4,38
38,81	44,82	0,20	11,40	1,18	1,15	-2	0	33,78
40,45	36,62	8,21	3,23	1,08	1,06	0	-1	8,81
29,46	34,28	2,46	5,73	1,08	1,15	0	-2	-26,51
32,34	35,68	2,37	3,81	1,02	1,06	-4	4	-1,45
26,91	35,23	7,32	2,41	1,00	1,21	-1	-3	-8,82
43,00	44,15	6,99	1,44	1,09	1,21	3	-2	1,49
30,68	30,39	3,76	1,16	1,08	1,02	-1	-1	-0,01
28,43	31,97	2,27	7,62	0,85	0,90	-1	3	5,87
36,06	#LEEG!	0,14	#LEEG!	1,08	#LEEG!	#LEEG!	#LEEG!	#LEEG!
45,49	44,26	7,59	7,84	1,27	1,22	-2	3	-27,89
31,22	33,95	8,37	3,76	1,16	1,15	-1	-2	22,02
29,71	36,37	4,97	4,50	1,08	0,66	2	-3	10,28
23,58	24,66	1,04	4,85	0,89	0,48	#LEEG!	17	10,31
38,85	39,37	6,62	3,91	1,24	1,27	0	6	-0,03
39,29	42,48	4,73	3,80	1,16	1,34	-1	5	-23,53
34,76	34,67	2,30	3,95	1,03	1,07	-3	-4	-22,10
32,34	25,52	1,77	2,94	0,98	0,35	8	0	5,92
35,73	#LEEG!	2,36	#LEEG!	1,18	#LEEG!	9	0	-11,74
41,21	33,75	0,03	5,13	1,30	0,42	2	3	14,66
31,30	#LEEG!	7,18	#LEEG!	1,03	#LEEG!	9	-7	4,38
37,10	37,19	2,96	2,19	1,08	0,40	3	2	23,52
30,44	31,15	2,83	2,61	0,99	0,42	1	4	-13,21
42,49	49,84	0,13	2,38	1,27	0,54	0	5	-13,21
33,57	38,43	3,19	2,10	1,43	0,70	0	3	-10,32

44,91	#LEEG!	0,05	#LEEG!	1,33	#LEEG!	#LEEG!	#LEEG!	#LEEG!
32,55	29,32	2,57	1,62	0,90	0,35	1	1	-13,21
26,36	33,58	0,62	3,69	1,07	0,73	3	-24	-7,39
26,69	32,00	6,97	7,01	0,97	0,33	3	-2	19,09
24,86	21,22	8,97	12,81	0,72	0,33	2	10	-2,91
34,67	39,07	6,08	2,26	1,27	1,16	-1	10	2,91
23,12	24,95	3,24	5,92	1,06	1,11	0	-13	-11,78
27,69	23,46	1,60	4,88	0,97	0,74	-4	-20	-8,82
33,91	28,49	5,30	13,34	1,25	1,21	0	6	25,00
31,83	29,93	7,61	15,45	0,91	0,72	0	1	-10,30
27,06	23,21	2,70	16,36	0,97	0,97	0	1	-1,50
31,91	#LEEG!	1,09	#LEEG!	1,05	#LEEG!	#LEEG!	#LEEG!	#LEEG!
34,79	31,70	6,80	8,16	0,84	0,74	-2	-1	-29,43
22,54	35,11	4,92	8,00	0,81	1,07	4	-26	30,84
22,41	45,84	3,33	8,23	0,94	0,99	-1	-6	16,20
29,96	16,22	4,99	7,34	0,85	0,82	-2	-7	-1,46
26,44	42,69	1,17	4,24	0,89	1,02	0	-16	13,22
23,78	32,07	1,87	0,25	0,86	0,89	-1	6	-1,42
25,41	28,11	4,23	0,83	0,78	0,77	3	-14	-25,03
40,04	30,62	11,61	1,92	1,05	1,04	2	-5	11,74
32,83	32,43	1,08	4,78	0,99	0,94	-1	-4	20,59
28,93	40,26	5,02	1,03	0,97	1,05	-4	-7	23,51
42,93	26,84	3,64	3,12	1,17	0,91	-1	-2	-36,78
23,11	22,97	2,69	2,98	0,86	0,82	6	7	2,94
22,58	18,23	3,79	1,35	0,76	0,64	-2	12	-4,41
36,95	41,54	4,54	13,04	1,03	0,90	1	0	-8,85
33,73	33,94	3,09	1,04	1,11	1,04	4	0	36,72
32,78	#LEEG!	5,81	#LEEG!	0,91	#LEEG!	#LEEG!	#LEEG!	#LEEG!
22,41	23,17	1,02	9,19	0,89	0,90	3	-12	30,91
33,37	#LEEG!	5,34	#LEEG!	1,03	#LEEG!	-3	26	1,48
32,60	47,15	3,10	9,69	1,11	1,21	-1	-11	4,44
24,75	28,18	3,70	1,61	0,89	0,97	0	-2	11,74
21,34	22,23	3,11	0,86	0,80	0,86	-1	-1	-10,26
19,79	31,54	3,68	1,74	0,72	0,96	2	-6	-42,60
30,92	16,51	1,57	5,63	1,09	0,70	-4	9	23,54
36,81	28,00	4,43	5,08	1,19	1,07	5	0	-11,74
31,91	24,83	2,53	0,00	0,99	0,90	3	-14	19,12
24,98	21,79	1,78	13,15	0,65	0,76	-1	-5	14,74
17,89	26,83	0,09	5,28	0,81	0,97	1	-6	2,95
19,61	18,99	0,89	0,15	0,75	0,70	4	-4	10,34
37,12	29,57	2,64	3,86	1,11	1,13	2	-8	23,53
28,61	27,50	6,45	3,05	0,88	0,96	3	13	14,70
25,55	28,80	0,18	0,74	0,97	1,02	1	1	26,48
35,62	#LEEG!	0,77	#LEEG!	1,02	#LEEG!	#LEEG!	#LEEG!	-10,29
34,13	33,28	6,62	2,22	0,84	0,97	6	-4	-1,49
32,21	30,89	3,69	6,40	1,01	0,88	-2	-1	-22,01
38,02	33,94	0,38	1,53	1,00	0,98	3	8	44,15
42,44	37,90	12,45	7,38	1,21	1,24	0	-3	57,40

Change_KC	Change_KC	Change_KC	Change_KC	Change_SF	Change_SF	Change_SF	Change_SF	Change_SF
13,93	43,70	14,24	-15,00	0,00	-1,00	0,00	25,00	15,00
13,93	-6,30	7,11	5,00	0,00	-1,00	0,00	25,00	-5,00
-5,52	18,70	3,56	5,00	0,00	0,00	0,00	0,00	0,00
-2,81	0,00	-14,30	-35,00	0,00	0,00	0,00	0,00	10,00
2,79	12,50	3,60	20,00	1,00	0,00	-25,00	0,00	20,00
5,58	-6,30	-3,53	#LEEG!	0,00	0,00	0,00	0,00	25,00
2,80	43,70	-3,53	0,00	0,00	0,00	0,00	0,00	0,00
16,69	0,00	7,14	5,00	0,00	0,00	0,00	0,00	5,00
5,53	12,50	7,17	-15,00	0,00	0,00	0,00	0,00	0,00
5,54	6,25	-0,01	#LEEG!	0,00	-2,00	0,00	50,00	#LEEG!
-5,54	-18,75	-10,74	10,00	0,00	0,00	0,00	0,00	-15,00
-13,87	-25,05	14,26	-30,00	0,00	0,00	0,00	0,00	0,00
-13,93	-6,30	-14,31	-15,00	-1,00	-1,00	25,00	25,00	5,00
2,73	0,00	-17,83	0,00	0,00	0,00	0,00	0,00	10,00
#LEEG!	#LEEG!	#LEEG!	#LEEG!	#LEEG!	#LEEG!	#LEEG!	#LEEG!	#LEEG!
11,12	-18,80	-7,13	0,00	0,00	1,00	0,00	-25,00	0,00
25,03	18,75	-10,67	-5,00	-1,00	-1,00	25,00	25,00	5,00
-13,89	-12,50	-7,11	0,00	0,00	1,00	0,00	-25,00	15,00
16,62	37,50	17,83	55,00	-1,00	-1,00	25,00	25,00	20,00
-27,80	-0,05	3,60	20,00	1,00	0,00	-25,00	0,00	5,00
-2,80	-6,25	-14,30	5,00	0,00	0,00	0,00	0,00	-10,00
#LEEG!	#LEEG!	#LEEG!	#LEEG!	#LEEG!	#LEEG!	#LEEG!	#LEEG!	#LEEG!
-2,82	-6,25	-7,14	-5,00	0,00	1,00	0,00	-25,00	-5,00
5,54	-18,80	-7,16	0,00	0,00	1,00	0,00	-25,00	-5,00
2,73	-0,05	-3,60	-15,00	0,00	-1,00	0,00	25,00	0,00
-13,92	6,20	0,04	-10,00	-1,00	0,00	25,00	0,00	10,00
8,31	6,25	17,83	-15,00	1,00	0,00	-25,00	0,00	5,00
-2,77	-0,05	-7,10	-15,00	0,00	0,00	0,00	0,00	-15,00
-5,60	6,25	-3,57	-5,00	-1,00	0,00	25,00	0,00	5,00
-8,38	-0,05	-0,03	-20,00	0,00	0,00	0,00	0,00	-5,00
0,00	-12,50	0,00	0,00	0,00	-1,00	0,00	25,00	0,00
0,02	0,00	3,60	-15,00	1,00	0,00	-25,00	0,00	0,00
8,30	-0,05	-7,19	5,00	-1,00	-2,00	25,00	50,00	20,00
-5,51	12,50	0,01	-15,00	0,00	-1,00	0,00	25,00	5,00
-11,09	6,25	10,69	5,00	0,00	0,00	0,00	0,00	10,00
-8,38	0,00	3,61	0,00	1,00	0,00	-25,00	0,00	25,00
8,36	18,75	-0,01	5,00	0,00	0,00	0,00	0,00	-5,00
-8,29	-18,80	-0,04	-10,00	1,00	0,00	-25,00	0,00	-5,00
-11,07	0,00	0,01	10,00	0,00	0,00	0,00	0,00	-5,00
#LEEG!	#LEEG!	#LEEG!	#LEEG!	#LEEG!	#LEEG!	#LEEG!	#LEEG!	#LEEG!
2,76	6,25	10,69	10,00	0,00	0,00	0,00	0,00	5,00
-5,58	-6,30	3,57	-10,00	0,00	0,00	0,00	0,00	5,00
11,07	-6,30	14,30	10,00	0,00	-1,00	0,00	25,00	10,00
2,82	-6,30	-7,16	-15,00	0,00	0,00	0,00	0,00	-15,00
2,74	6,25	-3,57	-25,00	0,00	0,00	0,00	0,00	-5,00
8,36	6,25	-3,57	15,00	0,00	0,00	0,00	0,00	0,00
-2,77	12,45	-0,01	-10,00	0,00	0,00	0,00	0,00	0,00
0,03	12,50	3,56	15,00	0,00	0,00	0,00	0,00	5,00
0,02	6,25	3,54	-5,00	0,00	0,00	0,00	0,00	-10,00

19,44	37,45	7,10	5,00	1,00	1,00	-25,00	-25,00	0,00
2,82	6,25	3,56	5,00	0,00	0,00	0,00	0,00	-10,00
-0,02	12,50	-3,53	10,00	-1,00	0,00	25,00	0,00	5,00
-13,86	-43,80	-14,26	-15,00	0,00	0,00	0,00	0,00	-20,00
-8,30	-12,55	10,69	0,00	0,00	1,00	0,00	-25,00	-15,00
22,22	18,75	21,40	15,00	-1,00	-2,00	25,00	50,00	-10,00
0,00	-6,25	3,53	0,00	#LEEG!	#LEEG!	#LEEG!	#LEEG!	#LEEG!
13,93	6,20	-3,56	-10,00	0,00	0,00	0,00	0,00	5,00
2,77	-25,05	-17,90	-10,00	1,00	0,00	-25,00	0,00	-20,00
-8,29	12,50	3,59	5,00	0,00	-2,00	0,00	50,00	5,00
-0,03	0,00	7,10	5,00	1,00	-1,00	-25,00	25,00	-10,00
5,52	12,50	3,56	25,00	0,00	0,00	0,00	0,00	0,00
-2,78	-6,25	-3,53	-15,00	1,00	1,00	-25,00	-25,00	10,00
-2,81	-12,55	-0,04	-15,00	0,00	0,00	0,00	0,00	5,00
-22,22	6,20	-17,86	-20,00	0,00	0,00	0,00	0,00	-20,00
-5,59	6,20	0,00	-45,00	0,00	0,00	0,00	0,00	-5,00
-19,41	0,00	-3,56	-40,00	0,00	0,00	0,00	0,00	-10,00
-2,74	6,20	-0,04	-10,00	-1,00	-2,00	25,00	50,00	-5,00
-25,01	-12,55	-10,69	-25,00	1,00	1,00	-25,00	-25,00	-20,00
-11,10	-18,75	-28,54	-50,00	0,00	0,00	0,00	0,00	-20,00
-22,22	-6,30	3,60	-20,00	-1,00	-2,00	25,00	50,00	-15,00
-22,21	-18,75	-25,03	-15,00	-1,00	0,00	25,00	0,00	-5,00
19,44	-12,55	28,53	10,00	0,00	0,00	0,00	0,00	0,00
-2,76	-6,25	3,61	10,00	1,00	0,00	-25,00	0,00	-5,00
-2,79	-6,30	0,04	-10,00	0,00	1,00	0,00	-25,00	5,00
2,77	-6,25	3,60	0,00	0,00	-1,00	0,00	25,00	0,00
-2,79	-0,05	-7,11	20,00	0,00	0,00	0,00	0,00	15,00
#LEEG!	#LEEG!	#LEEG!	#LEEG!	#LEEG!	#LEEG!	#LEEG!	#LEEG!	#LEEG!
-2,81	6,25	7,11	-25,00	0,00	2,00	0,00	-50,00	-5,00
11,12	-6,25	-17,87	-10,00	1,00	0,00	-25,00	0,00	-15,00
-2,78	-12,55	0,00	-15,00	1,00	3,00	-25,00	-75,00	-25,00
0,00	-6,30	0,00	-5,00	0,00	0,00	0,00	0,00	-10,00
#LEEG!	#LEEG!	#LEEG!	#LEEG!	#LEEG!	#LEEG!	#LEEG!	#LEEG!	#LEEG!
#LEEG!	#LEEG!	#LEEG!	#LEEG!	#LEEG!	#LEEG!	#LEEG!	#LEEG!	#LEEG!
0,01	6,20	-3,59	-5,00	0,00	0,00	0,00	0,00	5,00
8,31	-0,05	-3,56	-55,00	1,00	0,00	-25,00	0,00	-10,00
5,60	6,20	14,31	-5,00	0,00	0,00	0,00	0,00	20,00
5,58	0,00	7,11	20,00	0,00	0,00	0,00	0,00	10,00
-0,03	-0,05	-10,71	10,00	0,00	0,00	0,00	0,00	-5,00
-5,54	0,00	-10,74	-30,00	0,00	1,00	0,00	-25,00	-10,00
0,03	12,45	-3,60	-5,00	1,00	0,00	-25,00	0,00	5,00
5,54	0,00	3,56	0,00	0,00	-1,00	0,00	25,00	0,00
5,56	-0,05	0,00	-30,00	0,00	0,00	0,00	0,00	-20,00
8,29	18,70	-3,56	10,00	0,00	0,00	0,00	0,00	10,00
-24,96	-12,55	-0,03	-25,00	0,00	0,00	0,00	0,00	-20,00
8,32	-12,55	-3,53	25,00	0,00	0,00	0,00	0,00	0,00
13,91	24,95	14,26	55,00	0,00	0,00	0,00	0,00	10,00
-8,31	-6,25	0,03	0,00	0,00	0,00	0,00	0,00	-5,00
-5,53	-0,05	32,10	15,00	0,00	0,00	0,00	0,00	0,00
2,82	0,00	0,00	-5,00	1,00	1,00	-25,00	-25,00	-10,00

-36,08	-31,25	-3,54	-20,00	0,00	1,00	0,00	-25,00	-10,00
8,34	-12,55	-3,61	-5,00	0,00	-1,00	0,00	25,00	10,00
-5,60	-6,30	-17,87	-10,00	0,00	0,00	0,00	0,00	-10,00
13,91	12,50	-3,60	25,00	0,00	0,00	0,00	0,00	0,00
24,97	-6,25	14,31	5,00	1,00	1,00	-25,00	-25,00	-10,00
0,00	12,50	0,00	10,00	0,00	0,00	0,00	0,00	10,00
0,02	-12,55	17,86	-10,00	0,00	-1,00	0,00	25,00	20,00
#LEEG!	#LEEG!	#LEEG!	#LEEG!	#LEEG!	#LEEG!	#LEEG!	#LEEG!	#LEEG!
2,82	-6,25	-3,59	-40,00	0,00	0,00	0,00	0,00	5,00
0,00	0,00	0,00	10,00	0,00	0,00	0,00	0,00	0,00
8,30	-6,30	10,67	20,00	0,00	0,00	0,00	0,00	-5,00
0,00	12,50	17,83	-5,00	0,00	0,00	0,00	0,00	15,00
16,71	18,75	10,76	25,00	0,00	1,00	0,00	-25,00	#LEEG!
-2,82	12,45	7,16	10,00	0,00	0,00	0,00	0,00	-20,00
5,54	12,50	3,53	0,00	0,00	0,00	0,00	0,00	0,00
5,60	18,70	0,00	5,00	0,00	0,00	0,00	0,00	0,00
0,00	0,00	0,00	0,00	0,00	0,00	0,00	0,00	0,00
-8,34	-12,50	-7,17	0,00	1,00	0,00	-25,00	0,00	-15,00
5,53	-6,30	7,14	5,00	0,00	0,00	0,00	0,00	0,00
2,80	6,20	-7,11	-5,00	0,00	0,00	0,00	0,00	5,00
5,53	-6,25	-7,10	-5,00	0,00	-1,00	0,00	25,00	5,00
8,30	-0,05	-3,54	5,00	0,00	-1,00	0,00	25,00	5,00
-0,01	6,20	-7,13	-10,00	0,00	1,00	0,00	-25,00	5,00
0,00	-18,75	3,61	-20,00	0,00	0,00	0,00	0,00	10,00
2,74	6,20	-3,57	20,00	0,00	0,00	0,00	0,00	10,00
-8,32	31,25	10,76	-10,00	-2,00	0,00	50,00	0,00	-5,00
25,02	18,70	10,69	40,00	0,00	1,00	0,00	-25,00	5,00
-5,53	-6,30	0,00	-15,00	0,00	0,00	0,00	0,00	0,00
-13,91	-6,25	-7,11	0,00	0,00	0,00	0,00	0,00	-15,00
-8,38	6,20	3,54	-15,00	0,00	-1,00	0,00	25,00	0,00
2,82	6,20	3,60	5,00	0,00	-1,00	0,00	25,00	0,00
0,04	6,25	0,03	0,00	0,00	-1,00	0,00	25,00	5,00
13,89	12,50	21,39	0,00	0,00	-1,00	0,00	25,00	0,00
-0,01	0,00	-7,16	-20,00	0,00	0,00	0,00	0,00	5,00
16,71	6,20	-3,56	#LEEG!	1,00	0,00	-25,00	0,00	20,00
5,57	-12,55	3,59	-30,00	0,00	0,00	0,00	0,00	0,00
-2,82	6,20	7,17	-5,00	0,00	0,00	0,00	0,00	10,00
-16,67	-50,00	-14,33	-60,00	0,00	-1,00	0,00	25,00	-5,00
11,07	-0,05	-0,01	35,00	0,00	-1,00	0,00	25,00	-10,00
#LEEG!	#LEEG!	#LEEG!	#LEEG!	#LEEG!	#LEEG!	#LEEG!	#LEEG!	#LEEG!
0,00	0,00	-7,14	0,00	1,00	1,00	-25,00	-25,00	0,00
-0,03	0,00	10,71	5,00	0,00	1,00	0,00	-25,00	5,00
-2,74	-6,30	-3,59	-15,00	2,00	1,00	-50,00	-25,00	15,00
5,59	31,25	-3,60	5,00	#LEEG!	#LEEG!	#LEEG!	#LEEG!	#LEEG!
8,30	12,50	7,10	15,00	0,00	1,00	0,00	-25,00	35,00
-25,00	-6,30	-7,19	0,00	0,00	1,00	0,00	-25,00	-15,00
11,14	6,25	17,84	25,00	0,00	#LEEG!	0,00	#LEEG!	15,00
19,40	-6,30	7,10	25,00	0,00	-1,00	0,00	25,00	0,00
19,44	0,00	17,87	-15,00	0,00	1,00	0,00	-25,00	15,00
-2,80	-0,05	-0,04	0,00	1,00	0,00	-25,00	0,00	#LEEG!

11,14	0,00	3,59	0,00	0,00	0,00	0,00	0,00	-5,00
#LEEG!	#LEEG!	#LEEG!	#LEEG!	1,00	1,00	-25,00	-25,00	5,00
2,81	-0,05	3,54	10,00	-1,00	0,00	25,00	0,00	0,00
#LEEG!	#LEEG!	#LEEG!	#LEEG!	0,00	1,00	0,00	-25,00	0,00
52,82	50,00	3,60	#LEEG!	1,00	-1,00	-25,00	25,00	-30,00
-8,30	#LEEG!	-7,16	-95,00	0,00	0,00	0,00	0,00	-20,00
0,03	0,00	3,56	0,00	0,00	0,00	0,00	0,00	0,00
-11,12	-6,30	-25,01	5,00	0,00	0,00	0,00	0,00	-30,00
-27,82	-18,80	-17,84	-20,00	0,00	0,00	0,00	0,00	-15,00
0,00	6,20	10,76	35,00	0,00	0,00	0,00	0,00	10,00
-13,89	-12,50	14,27	0,00	0,00	-1,00	0,00	25,00	5,00
2,80	12,50	7,16	5,00	0,00	1,00	0,00	-25,00	10,00
13,87	-0,05	0,00	5,00	0,00	0,00	0,00	0,00	0,00
8,36	12,50	7,13	0,00	-1,00	0,00	25,00	0,00	0,00
8,34	25,00	21,46	35,00	0,00	0,00	0,00	0,00	30,00
-0,01	6,25	14,33	10,00	0,00	-1,00	0,00	25,00	10,00
2,74	6,20	-0,01	20,00	0,00	1,00	0,00	-25,00	0,00
0,00	6,20	0,00	-10,00	0,00	0,00	0,00	0,00	-15,00
27,79	6,20	17,86	15,00	-1,00	-1,00	25,00	25,00	20,00
-5,51	18,75	-17,87	-5,00	-2,00	-1,00	50,00	25,00	25,00
27,81	25,00	10,69	25,00	0,00	0,00	0,00	0,00	20,00
-19,46	-6,30	3,61	-20,00	0,00	0,00	0,00	0,00	-10,00
-2,80	-6,30	7,19	0,00	0,00	0,00	0,00	0,00	-45,00
19,47	12,50	-0,03	15,00	0,00	-1,00	0,00	25,00	-5,00
16,71	-12,55	10,67	25,00	0,00	1,00	0,00	-25,00	5,00
-2,79	-12,55	-17,89	-5,00	-1,00	0,00	25,00	0,00	20,00
#LEEG!	#LEEG!	#LEEG!	#LEEG!	#LEEG!	#LEEG!	#LEEG!	#LEEG!	#LEEG!
0,03	12,45	-10,70	5,00	0,00	0,00	0,00	0,00	-10,00
#LEEG!	#LEEG!	#LEEG!	#LEEG!	#LEEG!	#LEEG!	#LEEG!	#LEEG!	#LEEG!
30,56	12,50	10,74	20,00	0,00	0,00	0,00	0,00	5,00
-13,89	-31,25	-7,19	0,00	0,00	0,00	0,00	0,00	-5,00
-13,86	-6,25	3,59	-10,00	0,00	0,00	0,00	0,00	5,00
27,73	12,50	21,39	45,00	0,00	-1,00	0,00	25,00	15,00
0,02	18,70	10,73	10,00	0,00	0,00	0,00	0,00	-5,00
-36,10	-0,05	-3,61	-10,00	-1,00	-1,00	25,00	25,00	0,00
13,86	0,00	3,60	-5,00	0,00	-1,00	0,00	25,00	5,00
5,51	-18,75	-7,10	15,00	0,00	-1,00	0,00	25,00	-5,00
#LEEG!	#LEEG!	#LEEG!	#LEEG!	#LEEG!	#LEEG!	#LEEG!	#LEEG!	#LEEG!
-11,08	6,20	0,03	10,00	0,00	0,00	0,00	0,00	0,00
24,99	18,75	14,24	35,00	0,00	0,00	0,00	0,00	15,00
19,47	-0,05	0,03	30,00	0,00	-1,00	0,00	25,00	15,00
-11,08	-6,25	-0,01	0,00	0,00	0,00	0,00	0,00	-10,00
-2,74	-12,50	3,57	-35,00	0,00	-2,00	0,00	50,00	10,00
-5,53	18,75	10,67	0,00	0,00	1,00	0,00	-25,00	0,00
-44,40	-43,75	-28,60	55,00	1,00	0,00	-25,00	0,00	-10,00
0,04	6,25	-10,76	-10,00	0,00	-1,00	0,00	25,00	5,00
-16,68	-31,25	0,01	5,00	#LEEG!	#LEEG!	#LEEG!	#LEEG!	#LEEG!
8,33	12,50	7,13	10,00	-1,00	0,00	25,00	0,00	-5,00
-13,89	-37,50	-17,89	45,00	0,00	0,00	0,00	0,00	-20,00
5,57	12,45	0,04	0,00	0,00	0,00	0,00	0,00	-10,00

2,81	-18,75	-3,54	-40,00	0,00	-3,00	0,00	75,00	-15,00
-8,32	-6,25	3,57	-5,00	-1,00	0,00	25,00	0,00	5,00
-13,91	6,20	-3,53	5,00	0,00	0,00	0,00	0,00	-25,00
-33,34	-6,25	0,04	-5,00	0,00	0,00	0,00	0,00	-10,00
-11,16	6,25	-3,59	10,00	-1,00	0,00	25,00	0,00	-5,00
0,02	0,00	10,69	0,00	0,00	0,00	0,00	0,00	0,00
-2,77	25,00	-3,54	30,00	0,00	0,00	0,00	0,00	10,00
16,70	18,75	3,54	20,00	0,00	-1,00	0,00	25,00	25,00
8,32	6,20	-0,01	50,00	0,00	-1,00	0,00	25,00	15,00
8,30	12,50	17,84	5,00	0,00	0,00	0,00	0,00	-20,00
2,77	6,20	10,76	35,00	1,00	0,00	-25,00	0,00	-5,00
8,34	6,20	7,11	-5,00	0,00	0,00	0,00	0,00	-10,00
-19,44	-6,25	-10,73	-30,00	0,00	0,00	0,00	0,00	-10,00
-2,79	-12,50	3,59	-15,00	0,00	0,00	0,00	0,00	10,00
-11,07	-6,30	3,56	15,00	0,00	0,00	0,00	0,00	-5,00
5,51	-12,55	7,10	-30,00	0,00	2,00	0,00	-50,00	15,00
-30,56	-25,05	-7,10	-15,00	0,00	0,00	0,00	0,00	10,00
5,51	6,25	3,59	25,00	0,00	0,00	0,00	0,00	0,00
2,80	18,70	-3,60	10,00	-1,00	0,00	25,00	0,00	15,00
-11,11	0,00	-17,86	0,00	0,00	-2,00	0,00	50,00	30,00
2,78	-6,25	-3,60	5,00	0,00	0,00	0,00	0,00	0,00
8,37	-37,50	-3,59	0,00	0,00	0,00	0,00	0,00	-45,00
5,60	-18,75	3,61	5,00	0,00	0,00	0,00	0,00	-10,00
27,77	6,20	57,10	-20,00	2,00	1,00	-50,00	-25,00	5,00
-2,73	-6,25	-3,56	10,00	0,00	1,00	0,00	-25,00	10,00
0,01	-0,05	-3,57	-20,00	0,00	0,00	0,00	0,00	15,00
33,33	62,45	-3,56	25,00	-1,00	-1,00	25,00	25,00	10,00
11,08	-0,05	10,70	10,00	1,00	0,00	-25,00	0,00	10,00
-19,41	-18,75	3,60	-5,00	1,00	0,00	-25,00	0,00	-40,00
5,53	-12,55	10,71	-25,00	0,00	1,00	0,00	-25,00	-35,00
13,93	6,20	-21,40	-5,00	-1,00	-1,00	25,00	25,00	10,00
0,04	-0,05	7,10	-5,00	0,00	0,00	0,00	0,00	0,00
11,07	-18,75	-3,53	5,00	0,00	1,00	0,00	-25,00	-10,00
8,33	-0,05	3,60	-5,00	1,00	0,00	-25,00	0,00	5,00
#LEEG!	#LEEG!	#LEEG!	#LEEG!	#LEEG!	#LEEG!	#LEEG!	#LEEG!	#LEEG!
-24,98	-43,80	-14,30	-20,00	0,00	0,00	0,00	0,00	0,00
13,88	25,00	10,76	10,00	0,00	0,00	0,00	0,00	20,00
0,01	6,20	-3,56	5,00	0,00	0,00	0,00	0,00	-5,00
16,68	0,00	7,10	5,00	0,00	0,00	0,00	0,00	-5,00
0,02	-6,30	-7,11	-25,00	0,00	-1,00	0,00	25,00	5,00
-27,82	-56,25	-42,87	-25,00	2,00	2,00	-50,00	-50,00	-40,00
-13,93	-6,25	14,33	5,00	0,00	0,00	0,00	0,00	-10,00
19,47	12,50	17,86	5,00	0,00	1,00	0,00	-25,00	15,00
-2,76	-6,25	7,14	-10,00	0,00	0,00	0,00	0,00	-5,00
36,13	12,50	7,19	-15,00	0,00	0,00	0,00	0,00	-5,00
-2,81	12,45	3,60	20,00	-1,00	0,00	25,00	0,00	15,00
5,59	6,20	10,73	5,00	0,00	-1,00	0,00	25,00	20,00
-2,73	-31,30	-7,10	-10,00	2,00	0,00	-50,00	0,00	0,00
-44,44	-18,80	-17,87	-40,00	2,00	1,00	-50,00	-25,00	-5,00
0,00	0,00	-3,60	-25,00	0,00	-1,00	0,00	25,00	0,00

#LEEG!	#LEEG!	#LEEG!	#LEEG!	#LEEG!	#LEEG!	#LEEG!	#LEEG!	#LEEG!
-5,59	0,00	17,86	-5,00	0,00	0,00	0,00	0,00	-35,00
-2,82	-6,30	17,87	-5,00	0,00	-1,00	0,00	25,00	-20,00
11,10	12,45	10,73	10,00	-1,00	0,00	25,00	0,00	10,00
-8,33	0,00	3,61	5,00	0,00	1,00	0,00	-25,00	-5,00
-19,40	6,25	14,33	-10,00	1,00	0,00	-25,00	0,00	-5,00
-13,91	6,20	3,56	5,00	0,00	0,00	0,00	0,00	25,00
-13,92	6,25	14,31	-55,00	0,00	0,00	0,00	0,00	-5,00
27,80	-6,25	7,11	-5,00	1,00	0,00	-25,00	0,00	-20,00
-22,19	0,00	-0,03	-50,00	-1,00	0,00	25,00	0,00	15,00
-19,41	-0,05	7,14	-15,00	0,00	0,00	0,00	0,00	10,00
#LEEG!	#LEEG!	#LEEG!	#LEEG!	#LEEG!	#LEEG!	#LEEG!	#LEEG!	#LEEG!
-16,62	-12,50	-14,27	-25,00	1,00	0,00	-25,00	0,00	-35,00
11,10	6,20	7,13	0,00	-1,00	-4,00	25,00	100,00	10,00
8,33	-6,30	3,57	-10,00	0,00	1,00	0,00	-25,00	0,00
27,78	-18,80	21,47	-20,00	0,00	-1,00	0,00	25,00	-30,00
33,37	6,25	25,01	0,00	-1,00	-4,00	25,00	100,00	-10,00
-19,40	-12,50	0,00	60,00	0,00	-1,00	0,00	25,00	-25,00
-19,48	-25,05	-28,59	-20,00	-1,00	0,00	25,00	0,00	-35,00
0,04	-6,30	14,26	-10,00	0,00	-1,00	0,00	25,00	10,00
19,43	-12,50	17,84	25,00	0,00	0,00	0,00	0,00	25,00
11,10	31,20	3,56	30,00	-1,00	-1,00	25,00	25,00	-5,00
-38,87	-37,50	-53,59	-25,00	0,00	1,00	0,00	-25,00	-35,00
-11,16	-18,75	7,11	-20,00	0,00	-2,00	0,00	50,00	-20,00
-11,13	-25,05	-17,84	-25,00	0,00	1,00	0,00	-25,00	-45,00
-5,57	-25,05	17,86	-25,00	0,00	1,00	0,00	-25,00	-20,00
47,21	12,45	-7,14	25,00	0,00	-4,00	0,00	100,00	30,00
#LEEG!	#LEEG!	#LEEG!	#LEEG!	#LEEG!	#LEEG!	#LEEG!	#LEEG!	#LEEG!
11,08	18,75	3,54	5,00	-2,00	-1,00	50,00	25,00	-30,00
16,70	25,00	-3,54	45,00	-1,00	-1,00	25,00	25,00	-20,00
16,66	-18,80	10,71	10,00	0,00	-1,00	0,00	25,00	-50,00
-2,80	12,45	21,39	10,00	0,00	-2,00	0,00	50,00	0,00
27,78	-31,25	28,56	-25,00	0,00	0,00	0,00	0,00	-45,00
-49,98	-37,50	-57,16	-40,00	0,00	0,00	0,00	0,00	-35,00
47,20	-12,55	28,60	0,00	-1,00	1,00	25,00	-25,00	0,00
-11,08	6,25	-10,71	-25,00	-1,00	-1,00	25,00	25,00	25,00
-0,02	-6,30	3,54	10,00	-1,00	-1,00	25,00	25,00	10,00
5,58	37,45	-10,73	-5,00	0,00	0,00	0,00	0,00	-15,00
-0,04	6,20	-21,43	0,00	0,00	0,00	0,00	0,00	20,00
-16,67	-12,55	14,33	0,00	-1,00	0,00	25,00	0,00	15,00
22,26	-37,50	3,61	10,00	1,00	-1,00	-25,00	25,00	10,00
11,12	-6,30	35,69	45,00	1,00	0,00	-25,00	0,00	15,00
33,32	-6,30	-7,13	-5,00	0,00	2,00	0,00	-50,00	-25,00
2,73	6,20	-7,13	5,00	1,00	0,00	-25,00	0,00	-15,00
13,84	0,00	-3,54	0,00	1,00	1,00	-25,00	-25,00	0,00
-16,67	12,45	-28,60	-25,00	2,00	0,00	-50,00	0,00	10,00
38,87	68,70	28,53	25,00	0,00	0,00	0,00	0,00	40,00
50,02	37,45	39,26	75,00	0,00	-2,00	0,00	50,00	75,00

Change_SF	Change_SF	Change_GS	Change_GS	Change_GS	Change_GS	Change_GS	Change_GS	Change_GS
31,25	12,24	0,22	12,51	14,96	-2,45	10,10	12,54	0,44
-12,50	0,00	-0,03	-12,64	-3,22	0,60	-5,24	-0,76	0,32
6,25	0,00	-0,08	-10,12	-9,45	-0,68	-7,93	-11,16	3,24
18,75	-10,20	#LEEG!	#LEEG!	#LEEG!	#LEEG!	#LEEG!	#LEEG!	#LEEG!
0,00	0,00	-0,19	4,24	-5,99	10,22	-1,92	-3,84	1,91
-12,50	0,00	#LEEG!	#LEEG!	#LEEG!	#LEEG!	#LEEG!	#LEEG!	#LEEG!
25,00	22,45	-0,17	-8,41	-1,56	3,74	-13,22	-6,56	6,66
12,50	12,24	-0,15	-3,70	5,96	2,95	-10,34	2,68	-2,03
12,50	-12,24	#LEEG!	#LEEG!	#LEEG!	#LEEG!	#LEEG!	#LEEG!	#LEEG!
0,00	10,20	#LEEG!	#LEEG!	#LEEG!	#LEEG!	#LEEG!	#LEEG!	#LEEG!
18,75	-10,20	0,08	0,78	-1,91	0,69	2,70	-1,88	1,53
-25,00	0,00	-0,08	-2,56	2,60	-2,38	-3,40	0,12	1,34
-12,50	-22,45	-0,04	-11,88	-6,74	1,41	-9,14	-4,58	4,57
18,75	0,00	0,11	1,41	3,18	1,77	0,61	6,04	5,44
#LEEG!	#LEEG!	#LEEG!	#LEEG!	#LEEG!	#LEEG!	#LEEG!	#LEEG!	#LEEG!
12,50	-22,45	0,29	1,20	-8,96	-1,41	-1,55	1,48	0,81
0,00	10,20	#LEEG!	#LEEG!	#LEEG!	#LEEG!	#LEEG!	#LEEG!	#LEEG!
-18,75	0,00	-0,39	2,47	-1,99	-4,10	-5,49	-8,31	2,81
12,50	32,65	#LEEG!	#LEEG!	#LEEG!	#LEEG!	#LEEG!	#LEEG!	#LEEG!
18,75	0,00	0,07	-2,96	1,95	4,90	-2,58	-2,65	-0,08
12,50	34,69	0,01	2,01	-2,99	5,00	2,65	-1,12	3,58
#LEEG!	#LEEG!	#LEEG!	#LEEG!	#LEEG!	#LEEG!	#LEEG!	#LEEG!	#LEEG!
-18,75	-22,45	0,01	-5,00	-11,11	-6,11	-6,57	-3,41	3,16
-6,25	-32,65	0,10	-4,34	-0,42	3,92	-0,73	-1,37	-0,64
-18,75	-12,24	0,19	-0,33	0,00	-0,33	3,87	1,93	1,94
31,25	10,20	-0,28	-2,65	-9,14	-6,49	-5,58	-10,38	-0,19
25,00	0,00	-0,09	-1,96	-4,61	-2,65	-7,10	-2,89	4,22
12,50	0,00	0,36	-0,47	2,16	2,63	8,78	6,67	-2,10
-12,50	0,00	-0,34	-6,97	-3,16	0,83	-7,65	-3,41	-1,25
0,00	0,00	0,29	2,69	3,19	0,50	4,63	2,80	-1,83
50,00	0,00	0,31	-0,10	4,90	5,00	8,91	7,94	-0,97
25,00	-32,65	0,20	-0,52	2,75	-3,26	7,82	1,99	-0,10
37,50	22,45	0,00	9,60	0,66	1,19	2,32	0,21	-1,45
-25,00	-12,24	0,00	-5,95	-2,14	3,81	-4,11	-4,35	-0,25
-6,25	0,00	0,58	1,93	10,73	5,02	7,16	15,64	4,10
0,00	0,00	0,08	10,73	-0,58	-8,96	9,90	4,57	-1,05
6,25	0,00	0,03	-3,34	0,57	3,71	-2,45	2,25	1,80
0,00	-10,20	-0,01	-2,21	-0,47	1,73	2,93	2,57	0,13
0,00	0,00	#LEEG!	#LEEG!	#LEEG!	#LEEG!	#LEEG!	#LEEG!	#LEEG!
#LEEG!	#LEEG!	#LEEG!	#LEEG!	#LEEG!	#LEEG!	#LEEG!	#LEEG!	#LEEG!
12,50	0,00	0,25	0,19	-1,38	-1,58	2,93	3,97	1,04
-6,25	-22,45	0,14	2,76	1,37	-1,37	-0,38	1,46	-1,42
18,75	10,20	0,03	14,14	9,46	1,13	1,33	-3,54	-1,78
-25,00	10,20	-0,11	-9,45	-4,78	4,67	-9,45	-4,88	3,41
-6,25	-12,24	0,31	0,71	15,27	14,40	6,88	9,42	-2,54
0,00	12,24	-0,17	9,32	8,84	0,49	4,01	2,30	1,71
-56,25	0,00	0,52	8,23	16,28	8,06	18,94	12,35	-6,59
50,00	12,24	0,19	0,85	1,56	-0,72	5,77	2,90	2,86
-12,50	0,00	0,32	5,14	10,14	-3,18	7,63	14,88	-4,40

-37,50	12,24	0,12	-0,15	-1,42	1,28	-0,04	1,15	-1,19
25,00	0,00	0,05	-5,93	-6,66	0,73	-2,98	-2,74	-0,24
-18,75	-12,24	0,17	1,15	4,11	-2,96	0,73	1,63	-0,90
-6,25	0,00	0,14	5,55	1,16	-1,55	5,65	0,81	2,28
0,00	0,00	-0,29	7,81	3,76	0,48	-4,32	-6,40	2,08
0,00	-10,20	-0,10	-0,74	-1,31	-0,43	-4,83	-5,66	-0,43
0,00	0,00	0,49	2,60	2,27	0,33	9,76	15,25	-3,37
#LEEG!	-29,39	0,05	0,56	6,83	-6,28	1,10	3,58	-2,48
-18,75	0,00	-0,12	7,08	0,43	-6,42	-1,52	-1,86	-0,34
-6,25	22,45	0,14	-0,94	-2,37	1,42	0,25	1,99	1,74
0,00	-10,20	0,15	2,89	-2,64	-0,87	3,64	-4,06	3,52
0,00	0,00	-0,24	-0,23	9,19	4,96	-2,67	-4,55	-1,87
-6,25	-10,20	-0,19	-4,30	-5,38	-1,09	-4,90	-5,86	-0,95
18,75	-10,20	-0,27	-0,17	-6,22	3,03	-4,69	-5,39	0,70
-6,25	0,00	0,09	4,22	1,01	-3,20	1,58	-0,11	-1,29
6,25	2,04	-0,08	-4,39	4,18	-5,60	-5,33	-4,13	-1,20
25,00	-10,20	-0,21	0,25	2,05	1,81	-1,66	1,15	2,81
31,25	10,20	-0,20	-11,52	-7,72	1,65	-4,03	-6,89	-1,45
6,25	0,00	0,20	-12,20	-8,31	3,87	-4,09	-0,30	1,18
43,75	0,00	-0,10	-3,08	5,17	-7,89	-1,85	3,45	-1,73
0,00	22,45	0,06	-3,13	-8,58	5,45	-6,24	-6,05	-0,19
-12,50	0,00	0,30	-0,48	2,46	2,24	3,93	3,82	0,11
0,00	10,20	0,05	6,43	3,18	-3,25	3,19	-2,06	-5,25
-6,25	0,00	0,20	15,49	4,70	-3,81	16,12	6,25	2,88
-18,75	0,00	-0,48	-7,11	-2,35	4,17	-13,02	-10,63	0,89
6,25	0,00	0,13	-2,30	-1,97	0,33	3,41	3,31	-0,10
25,00	12,24	0,11	6,59	5,36	-1,23	7,50	3,50	-2,60
#LEEG!	#LEEG!	#LEEG!	#LEEG!	#LEEG!	#LEEG!	#LEEG!	#LEEG!	#LEEG!
6,25	0,00	0,10	6,27	3,21	3,06	5,02	1,59	3,37
-12,50	-20,41	0,01	13,79	9,71	-2,93	4,95	5,43	0,48
-50,00	-12,24	-0,04	-1,25	0,82	2,07	-2,75	-3,71	-0,96
0,00	-20,41	-0,10	-12,81	-0,80	3,37	-6,52	-4,46	0,52
#LEEG!	#LEEG!	#LEEG!	#LEEG!	#LEEG!	#LEEG!	#LEEG!	#LEEG!	#LEEG!
#LEEG!	#LEEG!	#LEEG!	#LEEG!	#LEEG!	#LEEG!	#LEEG!	#LEEG!	#LEEG!
-12,50	0,00	0,32	-15,06	0,38	-9,28	8,06	0,32	-4,76
18,75	22,45	0,03	4,72	6,92	1,15	2,43	4,17	-1,74
50,00	22,45	0,11	-3,04	-0,47	2,57	-3,28	-0,95	0,51
31,25	0,00	0,09	2,46	2,99	0,52	-0,48	-0,50	-0,02
-6,25	0,00	0,36	-9,88	-3,66	5,05	3,98	0,48	-0,64
18,75	0,00	0,07	-8,74	-5,72	-3,02	-7,56	-5,17	-2,39
6,25	10,20	-0,14	4,90	4,26	0,64	0,26	0,68	-0,42
6,25	0,00	0,02	0,65	0,59	-0,06	6,10	3,81	2,29
-18,75	0,00	0,06	9,89	11,39	1,49	7,47	4,61	1,04
0,00	10,20	-0,23	2,73	0,54	-2,18	-4,49	-3,18	1,31
-18,75	-42,86	0,10	-2,83	3,87	6,69	-2,82	0,70	3,52
0,00	22,45	0,09	6,38	6,31	0,06	7,61	4,72	2,89
12,50	10,20	#LEEG!	#LEEG!	#LEEG!	#LEEG!	#LEEG!	#LEEG!	#LEEG!
-18,75	-12,24	0,14	0,08	-1,53	-0,27	0,12	3,35	1,85
-12,50	22,45	-0,09	2,70	3,42	0,73	-3,19	-7,01	-1,09
-43,75	0,00	#LEEG!	#LEEG!	#LEEG!	#LEEG!	#LEEG!	#LEEG!	#LEEG!

-18,75	-44,90	0,04	7,19	3,10	-4,09	6,56	4,31	-2,24
18,75	10,20	0,01	4,09	3,82	0,28	-1,91	-2,89	0,97
0,00	12,24	0,10	5,33	-3,31	3,36	5,08	2,30	2,77
6,25	-24,49	0,02	-3,68	-2,98	-0,57	-0,36	-0,87	0,51
-25,00	32,65	-0,18	-0,95	-2,61	-0,84	-2,73	-3,93	1,20
12,50	22,45	-0,06	8,81	4,08	-4,73	-1,48	-0,89	-0,39
18,75	-20,41	0,06	1,86	1,18	-0,68	0,55	-0,55	1,09
#LEEG!	#LEEG!	#LEEG!	#LEEG!	#LEEG!	#LEEG!	#LEEG!	#LEEG!	#LEEG!
6,25	10,20	-0,08	6,74	0,56	-2,82	2,84	1,21	1,38
#LEEG!	-32,65	0,11	7,05	7,96	-0,90	7,24	7,11	0,14
-6,25	0,00	0,27	0,42	2,46	-2,05	5,82	7,24	-1,42
18,75	20,41	0,02	-2,12	-5,75	3,64	-2,29	-2,08	-0,21
-6,25	0,00	-0,02	2,87	6,71	-3,84	-0,47	-0,56	0,10
6,25	0,00	-0,01	-8,96	-0,53	-3,29	-4,13	-6,02	1,89
0,00	32,65	0,11	0,38	-0,80	-1,19	0,27	0,93	0,67
0,00	10,20	-0,04	-11,95	-3,08	3,03	0,09	-3,19	-3,28
0,00	-10,20	0,04	-3,81	-2,26	1,55	0,57	1,32	-0,75
-31,25	-10,20	0,08	11,83	3,36	-8,47	11,26	6,68	-4,58
-12,50	-10,20	0,03	-7,37	-0,19	-7,18	-1,69	1,01	-2,70
12,50	10,20	-0,11	-6,33	-6,99	0,65	-0,97	-7,47	-3,08
25,00	10,20	0,22	0,78	1,65	0,87	-1,49	-6,96	1,67
-6,25	-20,41	-0,10	-2,76	-7,99	4,84	0,24	-3,37	3,61
18,75	24,49	#LEEG!	#LEEG!	#LEEG!	#LEEG!	#LEEG!	#LEEG!	#LEEG!
6,25	22,45	0,17	-2,90	0,67	3,57	-1,36	2,31	1,24
-43,75	-12,24	0,04	0,41	-0,77	1,17	-0,45	3,00	-1,36
12,50	22,45	0,02	-10,33	1,41	-3,88	-4,85	5,86	-3,96
25,00	0,00	0,70	9,46	8,85	-0,62	21,23	17,50	-0,44
0,00	-10,20	-0,06	8,53	8,80	0,27	8,56	9,74	-0,59
-37,50	-22,45	0,35	6,01	2,99	-3,01	10,47	6,64	-0,52
0,00	12,24	-0,02	-5,69	3,95	9,65	-2,79	4,88	6,83
-12,50	-12,24	0,00	-9,59	-10,97	1,38	-4,03	-1,25	0,48
0,00	0,00	0,00	-1,09	3,29	4,38	-2,58	1,53	3,03
18,75	0,00	0,35	4,06	5,01	-0,95	10,17	7,66	2,49
-18,75	0,00	0,04	-7,25	-6,48	0,68	-0,39	-3,84	2,50
0,00	-10,20	-0,19	-11,92	-12,91	-0,99	-9,43	-6,35	-0,85
12,50	0,00	0,09	1,29	-3,43	4,72	-1,36	2,38	2,47
-12,50	0,00	0,04	0,57	5,88	0,66	3,11	-1,39	-0,25
-12,50	-34,69	-0,30	4,82	8,87	4,05	5,27	7,01	-0,56
-37,50	-32,65	0,18	-3,10	-0,22	-2,68	-0,67	2,78	-2,67
#LEEG!	#LEEG!	#LEEG!	#LEEG!	#LEEG!	#LEEG!	#LEEG!	#LEEG!	#LEEG!
-25,00	-22,45	0,10	-6,84	0,33	7,17	0,23	1,05	-0,81
-25,00	0,00	-0,06	3,51	-3,96	-7,47	1,44	-0,04	-1,48
0,00	22,45	0,02	-10,04	-2,77	-0,82	-1,82	-0,80	-0,29
#LEEG!	#LEEG!	-0,23	-6,90	-3,89	-0,52	-5,89	-11,06	1,65
12,50	0,00	0,07	6,38	5,82	0,57	5,03	1,29	2,30
-6,25	-10,20	0,03	-7,67	14,09	21,75	-3,63	0,00	3,62
12,50	-22,45	0,21	-5,56	-10,70	5,14	3,36	0,06	3,30
0,00	-20,41	0,29	-6,87	-12,90	-6,02	5,85	1,82	-4,02
50,00	12,24	0,24	-1,51	-4,70	-3,20	6,24	5,22	-1,02
0,00	12,24	-0,01	4,50	-3,62	-8,10	2,75	-3,09	-1,30

6,25	0,00	0,29	9,88	10,66	0,78	7,01	15,63	-7,36
0,00	0,00	0,15	1,14	2,39	1,26	4,58	0,23	-4,34
0,00	-10,20	-0,20	-5,91	0,29	-5,52	0,76	-1,73	-0,23
-18,75	22,45	0,10	-2,16	-7,69	-4,96	-1,04	-1,06	-0,03
43,75	8,16	0,17	2,54	-1,29	-3,84	10,90	4,52	-6,38
-6,25	-22,45	0,11	2,38	8,30	-5,92	1,99	7,40	-3,99
12,50	0,00	0,13	4,86	10,43	-5,26	6,57	7,95	1,38
-25,00	-22,45	0,00	-8,47	-3,49	-4,98	-3,60	-0,78	-2,82
-37,50	-22,45	-0,10	-1,75	-2,87	1,12	-4,06	-4,15	0,09
18,75	20,41	#LEEG!	#LEEG!	#LEEG!	#LEEG!	#LEEG!	#LEEG!	#LEEG!
-6,25	-12,24	0,08	-7,35	3,87	4,00	-2,24	-0,63	1,09
-18,75	-10,20	0,24	-5,38	5,20	10,59	-4,55	2,12	6,68
18,75	10,20	#LEEG!	#LEEG!	#LEEG!	#LEEG!	#LEEG!	#LEEG!	#LEEG!
18,75	0,00	0,17	-1,74	-0,26	1,48	4,69	4,18	-0,51
12,50	0,00	0,40	9,47	12,85	3,38	10,97	5,03	-5,26
0,00	22,45	0,09	1,86	-2,04	-3,90	2,97	3,34	0,38
25,00	0,00	0,07	3,34	3,08	-0,25	4,95	3,97	-0,97
0,00	0,00	-0,07	-8,96	-5,58	-2,96	-7,87	-4,11	-3,77
6,25	22,45	0,13	-1,93	-5,79	-3,85	2,15	-4,74	-6,89
25,00	12,24	0,21	-0,96	-6,05	4,18	4,06	-3,39	-1,06
-12,50	12,24	0,25	-0,75	2,70	1,34	5,26	6,43	0,49
-12,50	-10,20	-0,18	-6,26	6,27	12,55	-4,50	-3,23	1,27
0,00	-42,86	-0,33	2,05	3,76	-1,71	-4,67	-2,98	-1,69
-18,75	-10,20	0,12	0,68	2,16	1,48	1,89	5,11	3,22
25,00	0,00	0,21	3,30	0,02	-3,27	-1,26	1,51	2,78
50,00	0,00	0,17	4,61	4,82	0,20	-0,22	0,95	1,16
#LEEG!	#LEEG!	#LEEG!	#LEEG!	#LEEG!	#LEEG!	#LEEG!	#LEEG!	#LEEG!
-6,25	22,45	0,02	-1,79	5,09	6,89	2,65	9,21	3,36
#LEEG!	#LEEG!	#LEEG!	#LEEG!	#LEEG!	#LEEG!	#LEEG!	#LEEG!	#LEEG!
-6,25	22,45	0,21	-6,83	2,27	4,74	-1,06	1,86	-2,92
-18,75	-22,45	0,01	-0,86	-5,12	-0,71	-3,05	-5,29	-0,78
12,50	0,00	0,04	1,13	-0,95	1,25	1,89	0,74	0,03
31,25	22,45	-0,05	2,67	4,12	1,44	-1,37	-1,16	0,21
0,00	0,00	0,05	5,60	3,09	2,51	2,49	3,37	0,88
43,75	57,14	0,05	-0,09	-5,12	5,01	1,30	-1,74	-3,04
-6,25	0,00	0,18	-12,10	-7,14	-0,28	-6,72	-2,18	-1,11
-6,25	-20,41	-0,08	-3,52	-0,87	2,65	-2,55	-4,67	-2,13
#LEEG!	#LEEG!	#LEEG!	#LEEG!	#LEEG!	#LEEG!	#LEEG!	#LEEG!	#LEEG!
-6,25	-10,20	-0,14	-3,83	-9,45	5,62	-4,95	-3,64	-1,30
12,50	0,00	0,16	9,61	5,24	-0,85	8,28	5,15	-3,13
12,50	12,24	-0,24	-5,06	-1,10	-3,96	-3,93	2,08	-5,20
-6,25	10,20	0,03	-6,25	-0,62	-5,63	1,96	3,18	-1,23
31,25	22,45	-0,01	6,63	-2,66	9,29	2,77	-2,47	3,56
-25,00	-12,24	#LEEG!	#LEEG!	#LEEG!	#LEEG!	#LEEG!	#LEEG!	#LEEG!
0,00	-22,45	-0,06	-5,79	0,88	6,67	-1,82	-2,90	-1,07
0,00	0,00	0,06	3,42	0,05	-3,37	2,31	2,58	0,27
#LEEG!	#LEEG!	#LEEG!	#LEEG!	#LEEG!	#LEEG!	#LEEG!	#LEEG!	#LEEG!
6,25	22,45	-0,05	2,04	1,67	-0,38	0,43	-1,52	0,08
6,25	0,00	#LEEG!	#LEEG!	#LEEG!	#LEEG!	#LEEG!	#LEEG!	#LEEG!
-12,50	0,00	0,06	-1,71	-1,06	0,65	2,65	-0,62	-1,29

25,00	0,00	0,17	11,13	1,95	-3,30	7,06	8,64	-0,35
0,00	-10,20	0,12	3,33	-8,76	-4,31	1,15	-2,98	-4,14
-25,00	-10,20	0,03	-2,87	-8,26	-0,34	-3,15	-6,54	-3,39
0,00	-10,20	0,13	-1,33	-6,32	4,98	5,94	1,92	-4,01
25,00	10,20	-0,11	6,54	7,86	1,32	5,20	8,88	0,33
6,25	0,00	0,03	-1,67	-7,47	3,97	1,10	1,36	-0,25
25,00	22,45	0,05	4,83	-2,29	-3,76	3,37	1,55	1,82
25,00	0,00	0,07	-3,20	8,55	2,86	0,25	6,09	1,58
12,50	12,24	0,04	-3,41	3,08	3,71	3,94	4,70	0,76
-18,75	-34,69	0,03	1,22	7,12	-5,89	0,98	3,06	-2,09
18,75	10,20	0,02	-4,60	-4,59	-0,01	0,80	-0,34	-0,14
0,00	0,00	-0,02	-3,26	-11,42	-8,15	-3,01	-2,57	0,44
12,50	0,00	-0,04	-3,27	-3,57	-0,30	-6,64	-12,47	4,86
18,75	10,20	0,05	2,36	0,41	1,44	2,47	2,58	-0,12
12,50	0,00	#LEEG!	#LEEG!	#LEEG!	#LEEG!	#LEEG!	#LEEG!	#LEEG!
12,50	-10,20	0,01	1,99	6,63	-4,64	-1,15	1,38	-2,53
0,00	-22,45	-0,10	2,22	4,52	2,31	-2,56	-0,13	2,43
0,00	0,00	-0,16	4,08	1,91	2,17	-1,31	-4,80	-1,61
-12,50	0,00	-0,02	-4,28	0,63	4,91	3,03	4,67	1,64
12,50	22,45	-0,01	2,31	6,74	-4,44	-1,18	-2,94	-0,02
-37,50	-10,20	0,19	6,64	-2,57	3,04	4,16	8,38	2,86
-37,50	-10,20	0,10	-13,87	-1,69	-1,05	0,12	3,32	-1,66
0,00	-10,20	0,30	4,38	1,68	0,82	7,51	8,35	0,84
-43,75	-22,45	0,05	1,28	-2,75	-1,27	2,28	-1,83	1,92
12,50	-10,20	0,05	-1,00	-3,03	-2,04	4,37	0,25	4,11
-25,00	-32,65	-0,06	-1,57	-1,84	-0,27	-1,29	0,83	-1,54
12,50	2,04	-0,11	-1,85	2,75	2,26	-1,65	-2,82	-0,39
-18,75	-12,24	-0,03	-6,16	4,10	1,74	-6,23	1,27	-7,50
-6,25	-12,24	-0,28	-4,63	3,31	7,95	0,06	3,18	0,16
-6,25	-44,90	-0,02	1,58	2,19	0,62	2,28	3,90	-0,20
-12,50	0,00	0,25	1,49	3,05	-1,56	1,23	2,67	1,44
-6,25	0,00	0,10	-1,25	0,41	1,68	-2,15	-0,10	1,31
0,00	-12,24	-0,11	1,29	3,34	2,05	-1,41	-0,59	0,82
-6,25	10,20	0,05	-5,26	-2,99	-2,27	-4,97	-2,07	-2,89
#LEEG!	#LEEG!	#LEEG!	#LEEG!	#LEEG!	#LEEG!	#LEEG!	#LEEG!	#LEEG!
-25,00	-34,69	0,15	4,60	5,01	0,41	6,38	6,32	-0,07
31,25	12,24	-0,12	-2,34	-4,25	1,92	-2,97	-3,89	0,92
0,00	-10,20	0,19	0,56	-0,41	-0,96	2,68	1,67	-1,02
0,00	-10,20	-0,10	-13,74	-3,70	10,03	-3,33	-5,21	-1,88
0,00	-22,45	0,10	-6,25	3,24	0,15	-0,39	2,41	-2,80
-68,75	-22,45	0,07	-10,28	-9,32	0,96	0,01	-4,15	-1,28
-12,50	-42,86	0,07	5,68	4,17	-1,51	2,64	2,91	-0,26
37,50	-12,24	-0,15	3,80	1,44	-2,35	-0,86	1,06	1,92
12,50	10,20	#LEEG!	#LEEG!	#LEEG!	#LEEG!	#LEEG!	#LEEG!	#LEEG!
6,25	22,45	-0,04	-3,86	-0,66	3,19	-3,38	-3,80	-0,42
0,00	-22,45	#LEEG!	#LEEG!	#LEEG!	#LEEG!	#LEEG!	#LEEG!	#LEEG!
25,00	32,65	-0,03	8,49	-1,84	4,01	1,73	-1,12	2,85
-12,50	-22,45	-0,05	-2,75	-1,45	-1,30	-3,17	-2,17	0,52
-12,50	-22,45	0,00	-6,22	-1,70	4,54	-2,46	-1,30	1,16
12,50	12,24	-0,22	-1,71	-6,24	4,53	-8,36	-7,93	-0,43

#LEEG!	#LEEG!	#LEEG!	#LEEG!	#LEEG!	#LEEG!	#LEEG!	#LEEG!	#LEEG!
-18,75	-10,20	0,13	0,72	-3,04	3,76	0,48	-2,63	1,22
-12,50	12,24	0,09	-1,06	2,82	3,88	-1,39	4,34	0,99
-6,25	12,24	0,17	3,62	2,52	-1,10	3,30	3,89	0,58
0,00	0,00	-0,08	-5,47	0,77	6,23	-5,99	-7,57	-1,57
-31,25	-32,65	-0,18	-0,31	-9,61	2,29	-0,10	-6,08	-3,86
0,00	12,24	0,18	-10,99	-10,65	0,34	-3,08	-2,41	-0,67
-6,25	-22,45	-0,04	-19,93	-21,93	2,01	-14,26	-21,50	7,22
43,75	-10,20	0,05	-9,28	-18,12	0,78	-10,89	-7,80	-0,70
12,50	22,45	-0,16	-5,38	1,43	6,81	-7,53	0,88	4,28
-12,50	-22,45	0,01	10,09	6,74	-3,35	3,18	4,96	1,78
#LEEG!	#LEEG!	#LEEG!	#LEEG!	#LEEG!	#LEEG!	#LEEG!	#LEEG!	#LEEG!
-18,75	-69,39	-0,08	-8,82	-2,77	-4,61	-8,65	-3,59	4,30
0,00	22,45	0,19	-4,30	6,06	-2,36	5,49	6,72	1,23
-37,50	0,00	0,07	-3,66	2,55	-0,83	1,00	6,13	-3,91
-6,25	-22,45	-0,02	-7,01	13,81	-11,57	-9,32	-7,72	1,60
18,75	67,35	0,22	-5,14	-10,65	-5,52	4,65	3,17	-1,48
0,00	0,00	-0,23	-22,71	-21,85	0,86	-1,39	-7,07	1,68
-25,00	-22,45	-0,08	-9,11	4,53	-2,78	-13,84	-2,62	-1,74
6,25	2,04	0,09	-6,80	-3,17	0,51	-6,54	-8,26	0,83
56,25	44,90	-0,07	-2,26	-2,09	0,15	-5,35	10,01	10,65
-50,00	10,20	-0,03	0,49	-11,85	-4,16	-1,19	1,23	-2,42
-93,75	-69,39	-0,25	15,08	1,09	-8,64	-5,36	-4,94	-0,42
12,50	0,00	-0,01	14,94	11,48	-3,47	-0,92	3,26	-0,56
-25,00	-22,45	-0,08	-6,65	-5,71	0,94	-6,19	-2,01	-4,18
-50,00	-22,45	-0,16	9,49	-5,23	-11,67	3,52	-6,55	-6,61
18,75	24,49	-0,01	8,83	0,88	-7,95	6,58	-1,57	-7,29
#LEEG!	#LEEG!	#LEEG!	#LEEG!	#LEEG!	#LEEG!	#LEEG!	#LEEG!	#LEEG!
31,25	-34,69	-0,04	6,28	4,65	-1,63	3,44	2,41	-1,02
6,25	10,20	#LEEG!	#LEEG!	#LEEG!	#LEEG!	#LEEG!	#LEEG!	#LEEG!
-25,00	-10,20	-0,05	2,76	6,19	-0,81	2,93	0,23	-1,15
-31,25	-24,49	0,04	-3,01	-4,49	-1,49	4,67	1,35	-3,29
-31,25	-10,20	0,10	-7,09	7,78	14,49	-3,44	-4,81	-1,31
-25,00	-22,45	0,24	-0,81	1,66	2,47	8,30	4,53	3,04
-37,50	-24,49	-0,38	-7,10	-33,76	23,96	-17,56	-41,61	19,44
18,75	-42,86	-0,08	12,74	9,86	2,88	4,26	-0,46	0,50
0,00	12,24	-0,08	10,18	6,79	3,38	8,16	2,93	-3,95
-31,25	-32,65	0,04	-23,31	-6,14	17,18	-1,49	1,80	-1,29
-6,25	-12,24	0,10	1,56	8,63	-5,93	8,85	3,17	1,28
-6,25	-34,69	-0,05	2,00	3,83	-1,83	-0,10	1,29	-1,39
-12,50	30,61	0,01	6,00	6,95	-0,88	10,48	4,85	-4,24
-18,75	-32,65	0,05	-7,25	-6,29	-0,96	-4,03	-7,50	3,47
-100,00	-79,59	0,01	-6,07	3,01	5,67	-2,01	1,91	0,74
-31,25	-44,90	#LEEG!	#LEEG!	#LEEG!	#LEEG!	#LEEG!	#LEEG!	#LEEG!
-18,75	0,00	0,11	-0,24	6,15	-4,11	4,25	0,83	3,42
-31,25	-24,49	-0,24	-21,26	-5,66	15,60	-17,89	-3,14	14,75
50,00	14,29	-0,02	-19,06	-4,48	-0,88	-2,67	-2,58	0,07
37,50	67,35	0,13	-6,02	-8,33	0,27	-3,25	2,09	2,58

Change_GS	Change_GS	Change_GS	Change_GS	Change_GS	Change_GS	Change_GS	Change_GS	GShip
-0,14	5,07	0,54	2,31	2,99	16,99	14,00	0,17	-1,96464
0,03	-5,16	-0,54	-0,64	-1,62	-6,26	3,92	0,01	-1,62946
-0,02	-1,51	-4,80	0,38	1,89	-8,90	10,79	0,66	0,56907
#LEEG!	#LEEG!	#LEEG!	#LEEG!	#LEEG!	#LEEG!	#LEEG!	#LEEG!	#LEEG!
0,05	-3,54	-3,90	0,36	-12,44	-4,89	3,04	-0,17	0,75921
#LEEG!	#LEEG!	#LEEG!	#LEEG!	#LEEG!	#LEEG!	#LEEG!	#LEEG!	#LEEG!
0,04	-1,08	2,27	-3,35	-4,66	-1,99	2,67	0,46	-0,66264
0,03	-4,34	0,87	-4,68	-9,18	-5,83	3,35	0,68	0,04134
#LEEG!	#LEEG!	#LEEG!	#LEEG!	#LEEG!	#LEEG!	#LEEG!	#LEEG!	#LEEG!
#LEEG!	#LEEG!	#LEEG!	#LEEG!	#LEEG!	#LEEG!	#LEEG!	#LEEG!	#LEEG!
-0,03	2,15	0,14	2,03	1,96	0,76	1,19	-0,42	-1,38234
0,08	-0,48	0,06	-0,54	-12,36	7,76	2,81	-0,60	-0,51166
0,04	-3,52	-1,59	1,93	-1,67	4,49	6,16	-0,56	-0,97563
0,12	-3,05	1,35	2,29	9,85	9,87	0,01	-0,34	-0,70570
#LEEG!	#LEEG!	#LEEG!	#LEEG!	#LEEG!	#LEEG!	#LEEG!	#LEEG!	1,65918
-0,22	0,86	-0,93	-1,79	6,02	6,58	-0,52	-0,41	-1,02480
#LEEG!	#LEEG!	#LEEG!	#LEEG!	#LEEG!	#LEEG!	#LEEG!	#LEEG!	#LEEG!
0,14	1,68	-5,94	-7,35	-10,80	-21,81	-10,16	-0,65	-0,10823
#LEEG!	#LEEG!	#LEEG!	#LEEG!	#LEEG!	#LEEG!	#LEEG!	#LEEG!	#LEEG!
0,00	-0,09	1,32	1,41	-1,64	3,08	-4,74	-0,48	-0,62168
-0,02	-1,04	0,48	-1,52	2,55	0,53	2,02	0,03	-0,18576
#LEEG!	#LEEG!	#LEEG!	#LEEG!	#LEEG!	#LEEG!	#LEEG!	#LEEG!	-1,30836
-0,05	-4,29	-2,41	0,50	2,13	3,38	0,06	0,00	-1,00159
-0,08	0,45	-3,34	2,98	-1,24	-0,89	0,35	0,05	0,43030
-0,14	2,72	3,32	-0,61	10,87	5,50	-2,15	0,12	-1,03563
0,05	-2,73	-5,64	-2,91	-9,17	-8,05	-1,12	-0,28	-0,30737
0,07	-4,32	-2,45	1,88	-2,69	-1,72	0,97	-0,06	-0,53637
-0,13	2,68	1,70	-0,99	8,53	8,38	0,15	0,26	0,08245
0,08	-3,59	-8,43	-1,57	-3,58	-2,25	-1,33	-0,33	-0,67050
-0,14	6,00	4,23	-1,78	9,38	3,64	-2,39	0,19	-0,03740
-0,27	3,99	7,92	3,92	8,33	-0,65	2,78	0,16	-0,68764
-0,02	-3,36	2,73	6,09	10,48	13,67	-3,19	0,20	-1,28712
0,02	3,38	-0,92	1,91	5,25	-1,97	7,22	0,03	-0,62543
-0,04	-1,19	-1,80	0,62	-10,78	-6,54	0,47	-0,02	0,66172
-0,62	10,18	11,40	-0,78	16,69	10,88	-4,12	0,33	-2,87025
-0,09	3,14	3,17	0,02	7,24	-3,73	-6,70	-0,02	-0,33369
-0,02	-1,55	6,08	7,16	1,37	-2,44	3,82	-0,04	0,95612
-0,01	2,53	3,12	0,60	14,98	3,82	9,24	-0,02	-0,57137
#LEEG!	#LEEG!	#LEEG!	#LEEG!	#LEEG!	#LEEG!	#LEEG!	#LEEG!	-1,65761
#LEEG!	#LEEG!	#LEEG!	#LEEG!	#LEEG!	#LEEG!	#LEEG!	#LEEG!	0,82396
-0,32	3,23	0,63	2,60	1,52	-1,12	-2,64	0,00	-0,93133
0,02	3,06	1,24	1,82	5,38	4,10	-0,21	0,14	-0,46609
-0,09	5,20	0,23	-4,97	-0,08	-0,25	0,17	-0,04	0,40350
0,05	-3,14	-3,02	0,12	-2,07	-0,47	1,60	-0,08	1,55246
-0,08	2,87	2,22	0,65	-9,22	-0,88	-7,53	0,28	0,47873
0,04	0,91	2,75	1,84	-3,11	0,44	3,55	-0,11	1,20985
-0,24	2,25	2,46	0,20	28,63	7,48	-19,11	0,39	-0,87185
-0,12	1,72	0,56	-1,16	3,08	7,76	4,69	0,10	0,52969
-0,26	7,49	3,31	-0,03	-1,02	-2,44	1,41	0,20	-1,44606

-0,06	4,08	-1,58	-0,16	2,99	2,72	-0,27	0,11	-0,54763
0,02	-3,51	-5,84	-0,39	-2,74	0,79	-3,53	0,03	1,03117
-0,01	-1,53	4,76	-4,70	2,19	6,66	-4,47	0,17	-0,75891
-0,06	5,41	0,88	-4,53	1,12	-0,03	-1,16	0,15	-0,72849
0,01	5,75	-4,88	1,65	-10,98	-5,08	5,91	-0,31	0,34369
0,01	0,06	-1,87	-1,93	-20,26	-15,53	-4,73	-0,09	2,55044
-0,27	6,48	6,17	-0,15	6,62	10,56	0,54	0,32	-1,83807
-0,02	-3,44	6,54	-9,98	2,49	2,23	-0,24	0,05	0,51834
0,05	-2,55	-0,89	0,00	-10,44	-7,67	2,77	-0,04	0,32187
-0,09	4,95	2,65	1,54	-0,05	4,39	-4,44	0,06	0,09749
-0,01	0,60	1,02	-0,43	-1,35	-2,29	-0,93	0,13	1,16205
0,08	-4,59	-4,26	-0,33	-9,93	-4,43	-1,31	-0,20	1,00105
-0,04	-5,18	-2,92	2,26	-12,58	-1,77	10,81	-0,22	0,65895
0,11	-2,35	-4,58	0,03	-17,21	-25,82	-7,13	-0,16	1,81188
-0,02	2,48	-2,80	-5,28	-0,13	1,74	1,87	0,07	0,41180
0,04	-3,91	3,22	-3,52	-4,58	-13,24	-5,90	-0,05	0,27058
0,08	1,27	-3,19	-1,13	-5,99	5,23	-11,21	-0,10	1,16842
-0,01	-6,76	-4,94	-1,82	-10,98	-5,32	5,66	-0,20	-0,90238
-0,08	-0,24	1,54	1,78	9,08	-1,21	7,85	#LEEG!	-0,04502
0,10	2,60	3,44	0,85	-5,70	-0,09	5,60	-0,06	0,42616
0,08	4,46	1,42	3,04	-0,56	10,62	-11,18	0,16	-0,27063
-0,21	10,80	8,19	2,61	2,72	7,10	4,37	0,16	-0,90951
0,07	5,09	0,94	-4,15	3,34	0,50	-2,83	0,11	0,07923
-0,05	1,91	-0,28	-2,19	23,00	5,99	-8,51	0,22	1,07939
0,18	-6,42	-7,30	-0,86	-19,55	-19,77	0,22	-0,85	0,72811
-0,03	-3,43	-3,72	-0,29	7,90	5,71	-2,19	0,11	-0,18425
-0,02	1,14	1,10	-0,04	-3,99	-5,67	-1,68	0,04	0,50558
#LEEG!	#LEEG!	#LEEG!	#LEEG!	#LEEG!	#LEEG!	#LEEG!	#LEEG!	-1,34738
-0,08	3,76	0,18	-2,63	7,13	-4,58	2,89	0,06	0,15750
-0,02	7,48	5,61	-0,03	-2,31	3,54	-3,09	-0,02	-0,73397
0,12	-3,68	-1,28	-2,40	-5,42	-3,63	1,78	0,04	-1,41225
0,00	-8,70	-4,52	-3,22	-12,71	-6,40	-6,31	-0,06	1,22394
#LEEG!	#LEEG!	#LEEG!	#LEEG!	#LEEG!	#LEEG!	#LEEG!	#LEEG!	-0,51169
#LEEG!	#LEEG!	#LEEG!	#LEEG!	#LEEG!	#LEEG!	#LEEG!	#LEEG!	-0,83733
-0,08	-4,34	2,45	-6,79	8,06	8,84	-0,78	0,28	0,05600
0,09	-0,85	5,71	6,56	5,65	10,83	-0,45	0,08	-0,12595
-0,13	-0,13	-0,77	-0,62	1,64	2,41	0,76	0,01	0,46435
-0,01	-3,47	5,99	6,54	2,74	7,34	4,59	0,08	-0,07873
-0,19	-6,51	2,21	2,42	6,35	2,58	3,20	0,19	0,44604
-0,08	3,59	1,76	-1,65	-13,78	-15,33	1,56	0,03	0,33160
0,10	3,89	1,65	-2,24	-2,69	-8,36	4,66	-0,11	0,07712
-0,09	2,62	0,96	0,88	5,41	-6,43	2,46	-0,03	0,90808
0,04	1,41	1,20	-0,21	6,50	1,34	-5,16	0,11	-0,49341
0,10	4,70	3,83	-0,88	-10,65	-9,40	-1,24	-0,17	0,52863
0,01	-0,97	1,14	2,11	-0,74	1,74	-2,49	0,08	0,55272
0,03	1,48	3,53	-2,05	7,14	1,52	-1,52	0,07	1,75972
#LEEG!	#LEEG!	#LEEG!	#LEEG!	#LEEG!	#LEEG!	#LEEG!	#LEEG!	#LEEG!
-0,08	-6,80	2,66	-4,42	10,76	8,99	-1,77	0,11	0,00119
0,07	3,35	3,59	0,25	-16,21	-21,55	-0,58	-0,08	0,12100
#LEEG!	#LEEG!	#LEEG!	#LEEG!	#LEEG!	#LEEG!	#LEEG!	#LEEG!	#LEEG!

-0,12	3,29	5,99	2,71	-2,91	-2,71	0,21	-0,03	-0,50833
-0,07	-3,02	-0,98	-2,04	-7,73	-12,76	-0,99	-0,05	-0,30717
-0,06	0,74	2,14	1,40	-5,57	3,01	-2,27	0,04	2,21943
-0,02	0,99	-0,56	-1,56	-3,31	-3,63	0,33	-0,01	0,43045
0,14	-1,34	-6,23	-4,49	-6,39	-2,06	-4,33	-0,10	0,80846
0,08	7,50	4,94	-2,56	3,05	-1,70	4,75	0,01	0,95739
-0,03	4,85	-0,53	-4,30	-2,09	-1,67	-0,41	-0,01	0,71796
#LEEG!	#LEEG!	#LEEG!	#LEEG!	#LEEG!	#LEEG!	#LEEG!	#LEEG!	0,52897
-0,01	-0,02	-0,55	-0,54	-4,63	-1,17	0,08	-0,05	1,58700
-0,10	4,32	4,49	-0,17	-5,16	-1,27	-3,89	0,04	0,91830
-0,09	4,36	4,64	-0,28	4,45	5,25	0,81	0,25	-0,61612
-0,13	-0,66	0,01	0,69	-8,09	-9,57	1,47	-0,07	-0,67256
0,03	0,50	-3,36	3,86	6,36	-3,47	1,49	0,02	1,44536
0,07	-2,68	-1,08	-1,60	2,44	0,50	-1,94	0,01	-0,74467
-0,01	-1,30	-2,10	-0,32	-9,27	-12,16	2,90	0,06	0,29254
0,02	-4,29	-1,26	-3,04	-11,20	-14,25	3,05	-0,07	0,44265
-0,02	-2,82	-2,92	0,09	4,74	-1,76	6,48	0,04	0,65374
-0,01	3,69	5,30	1,61	-7,36	-10,93	-3,59	0,04	0,69673
-0,07	2,17	2,69	-0,53	-1,34	3,42	-3,07	0,01	-0,14194
0,08	-0,33	0,16	0,49	-11,41	-9,68	0,26	-0,09	0,45788
-0,07	1,75	5,33	3,57	8,28	19,34	6,14	0,13	0,41338
0,07	5,46	-2,38	7,83	-1,58	-3,63	-2,06	-0,09	-0,35497
#LEEG!	#LEEG!	#LEEG!	#LEEG!	#LEEG!	#LEEG!	#LEEG!	#LEEG!	0,07434
-0,08	0,54	4,70	1,29	5,62	6,94	1,31	0,11	0,51117
-0,02	1,31	-1,62	-2,92	-1,62	11,92	-2,00	-0,01	0,47597
-0,01	-1,10	-4,24	3,14	-4,15	-7,84	-3,69	-0,04	2,25291
-0,19	11,72	5,05	3,43	18,63	16,37	2,26	0,55	-1,15244
0,08	1,98	0,79	-1,20	11,48	10,99	0,48	-0,04	-0,57169
-0,11	-0,25	4,41	-0,88	1,81	10,20	7,05	0,29	-0,35774
0,04	-0,28	1,94	2,21	-4,58	-10,55	0,01	-0,01	-0,17520
-0,04	-4,29	3,73	8,03	-1,72	3,55	-5,07	-0,02	-0,17334
-0,04	-1,41	-4,79	3,38	-5,02	1,07	1,93	-0,02	2,10526
-0,07	-0,11	-0,85	-0,74	10,04	3,65	-6,39	0,28	-0,47586
-0,04	-2,30	-1,00	-1,31	2,03	-6,34	-5,36	0,01	1,43847
0,06	-4,70	-9,20	-2,89	3,35	1,05	-1,32	-0,07	0,98747
-0,06	-1,09	1,12	-2,21	2,54	3,45	0,91	0,05	0,84967
0,06	6,28	4,31	1,96	6,24	-0,59	6,82	0,07	0,81542
0,13	-10,00	-5,41	4,59	-11,58	-4,30	7,29	-0,19	2,78550
0,01	4,63	7,79	0,15	9,41	6,63	2,78	0,14	-0,60577
#LEEG!	#LEEG!	#LEEG!	#LEEG!	#LEEG!	#LEEG!	#LEEG!	#LEEG!	1,56143
-0,02	-0,39	3,22	3,42	6,02	5,37	-0,65	0,08	-0,13113
0,01	4,50	0,51	-3,99	-10,67	-10,18	-0,49	-0,09	1,24153
0,03	0,31	-2,02	-2,32	-0,58	2,02	2,58	0,05	0,76848
0,05	-1,47	-0,71	-0,78	-8,09	-5,39	2,71	-0,19	0,49073
-0,03	0,02	5,33	1,25	2,18	-3,03	1,17	0,05	0,70801
-0,01	-5,02	7,99	13,01	11,46	2,47	6,38	0,00	-0,14942
-0,17	0,61	-1,28	1,88	5,94	0,82	-1,45	0,13	-0,27440
-0,05	-0,38	0,48	0,85	13,13	14,46	-1,34	0,28	0,56421
-0,05	4,87	2,55	-2,32	6,51	8,45	-1,95	0,25	-0,92530
-0,02	-1,54	-1,64	-0,09	-11,58	-15,43	-3,86	-0,04	-0,14255

-0,05	6,63	11,18	3,13	0,44	0,72	-0,28	0,22	-0,84114
-0,02	-1,11	1,50	2,61	2,21	5,42	3,20	0,11	1,73047
-0,01	-1,32	-4,65	-3,33	-12,32	-11,86	-0,20	-0,22	2,43068
-0,05	0,89	-3,71	-4,60	1,56	4,21	2,65	0,09	-0,11084
0,01	-1,43	-3,77	-2,35	8,30	0,28	4,54	0,16	-0,73989
-0,08	0,15	0,78	-0,63	0,19	10,68	5,13	0,06	-0,90574
-0,09	3,64	4,32	-0,68	-7,61	-1,66	5,94	-0,73	0,98450
-0,07	-3,91	-7,59	-3,68	-1,44	0,43	1,86	-0,03	2,55505
-0,05	-5,49	0,13	-5,63	-4,79	-4,85	-0,06	-0,11	-0,85974
#LEEG!	#LEEG!	#LEEG!	#LEEG!	#LEEG!	#LEEG!	#LEEG!	#LEEG!	#LEEG!
-0,11	-2,03	3,21	5,25	1,15	-1,83	-2,98	0,00	-0,38462
-0,05	-0,94	-2,09	1,17	6,34	11,44	5,10	0,26	-1,01081
#LEEG!	#LEEG!	#LEEG!	#LEEG!	#LEEG!	#LEEG!	#LEEG!	#LEEG!	#LEEG!
-0,05	-2,91	-3,09	-0,17	15,98	11,97	4,01	-0,56	-0,89504
-0,11	2,70	15,86	13,16	3,85	13,48	9,12	0,41	-0,57588
-0,03	3,36	-0,11	2,18	-1,81	-2,13	0,32	-0,42	-1,00509
-0,02	-3,32	3,23	1,39	5,78	-0,84	0,88	#LEEG!	-1,06810
-0,10	0,34	-8,66	-6,17	-3,12	-2,69	-0,43	-0,73	1,41251
-0,08	-0,31	1,12	1,44	4,88	9,43	-4,05	-0,62	-0,41921
-0,02	2,58	-2,69	2,23	10,38	6,58	-0,41	-0,44	-0,33841
-0,14	-0,97	1,83	2,80	14,19	8,67	4,23	-0,35	-1,26907
0,09	-1,15	1,70	-0,12	-14,32	-20,11	5,79	-0,63	2,28044
0,10	-9,66	-6,35	-0,51	4,08	-0,68	4,76	-0,82	1,40982
-0,08	0,96	1,47	0,50	3,49	9,27	5,78	-0,44	-0,50117
-0,03	-2,73	0,76	-3,49	4,69	13,10	-8,42	0,15	1,07293
-0,02	0,44	0,99	0,55	7,19	6,62	-0,58	0,18	0,66283
#LEEG!	#LEEG!	#LEEG!	#LEEG!	#LEEG!	#LEEG!	#LEEG!	#LEEG!	-0,94620
-0,12	0,06	3,85	3,79	-1,69	-3,39	1,70	-0,06	0,67134
#LEEG!	#LEEG!	#LEEG!	#LEEG!	#LEEG!	#LEEG!	#LEEG!	#LEEG!	0,51331
-0,13	-1,96	-0,47	-1,49	#LEEG!	#LEEG!	#LEEG!	0,15	#LEEG!
-0,08	-2,56	-7,46	-4,30	-3,74	-1,06	2,69	-0,68	0,13968
0,04	-2,37	-0,54	-1,83	7,82	4,86	-2,96	-0,69	0,96312
0,01	-0,21	4,35	4,56	-3,83	-3,10	0,73	-0,07	0,79538
-0,04	4,69	-3,12	7,81	-3,08	5,41	3,91	-0,06	1,29938
-0,03	-3,67	-2,70	0,97	1,00	1,97	0,98	0,07	0,23164
-0,01	-3,51	-1,81	0,53	10,79	1,66	9,13	0,13	0,43617
-0,04	-2,49	-1,63	0,86	-3,39	-3,03	-0,36	-0,09	0,21038
#LEEG!	#LEEG!	#LEEG!	#LEEG!	#LEEG!	#LEEG!	#LEEG!	#LEEG!	2,57363
-0,06	2,11	-3,90	4,56	-4,43	-7,18	2,75	-0,17	0,79795
-0,11	5,15	-0,62	0,71	9,06	-3,23	-2,90	-0,64	0,32279
0,21	-2,75	1,49	-4,24	-2,84	0,17	2,93	-0,59	-0,05328
-0,02	-5,12	-1,02	-2,04	2,37	-0,75	2,55	0,01	-0,36254
-0,01	3,32	0,08	3,24	3,18	2,10	-1,09	0,01	-0,99053
#LEEG!	#LEEG!	#LEEG!	#LEEG!	#LEEG!	#LEEG!	#LEEG!	#LEEG!	#LEEG!
0,02	-8,07	-1,71	6,07	-1,58	-3,35	-1,73	-0,03	-1,10322
-0,03	2,72	-1,31	2,49	-0,97	-5,27	-1,89	0,05	1,31610
#LEEG!	#LEEG!	#LEEG!	#LEEG!	#LEEG!	#LEEG!	#LEEG!	#LEEG!	#LEEG!
0,03	-3,22	0,33	3,55	-0,72	-1,15	0,43	-0,50	0,21510
#LEEG!	#LEEG!	#LEEG!	#LEEG!	#LEEG!	#LEEG!	#LEEG!	#LEEG!	#LEEG!
-0,01	-2,17	1,13	3,30	8,64	-0,36	2,74	0,03	0,62687

-0,01	12,02	5,49	-6,54	6,52	7,76	1,24	-0,60	0,29559
-0,02	-1,62	-0,87	-0,74	9,07	4,65	-4,43	-0,66	0,31292
-0,10	-1,57	-2,33	-0,76	-6,56	-5,48	1,08	-0,54	0,53259
-0,04	-2,78	3,92	-1,12	-4,02	2,82	6,84	0,13	1,08924
-0,01	-3,07	-5,18	-2,11	-8,19	-3,96	2,87	-0,10	1,98862
0,07	-1,23	-2,90	-1,67	4,16	-2,03	5,62	0,05	-1,34379
0,00	5,58	-2,81	6,79	-1,82	2,62	1,75	0,05	-0,61197
0,01	1,32	6,27	1,38	2,11	5,85	-1,58	-0,66	0,07550
-0,06	4,43	0,88	0,56	4,01	9,18	-2,17	-0,52	0,17482
-0,06	1,70	4,70	1,90	2,43	5,36	-2,92	0,00	0,43422
0,03	2,73	-4,62	-2,07	-12,66	-1,04	4,19	0,01	1,53764
-0,06	0,93	-2,80	-3,13	-1,61	4,05	-1,18	-0,57	-0,07826
-0,04	-2,35	-7,59	-0,70	4,31	-5,96	-1,47	-0,11	1,07969
0,01	-1,40	5,00	2,98	3,41	-2,35	5,77	0,03	-0,36221
#LEEG!	#LEEG!	#LEEG!	#LEEG!	#LEEG!	#LEEG!	#LEEG!	#LEEG!	0,31280
0,01	2,72	-0,21	2,93	-4,69	1,87	-0,76	0,05	1,54624
0,00	-1,29	3,39	4,68	-7,33	-1,74	5,59	-0,08	0,02487
0,15	-1,27	4,84	-1,51	6,69	-5,24	6,13	-0,03	1,06741
0,05	-1,53	-2,74	-1,22	1,77	-8,96	-3,72	-0,60	0,66645
0,09	-1,25	-1,48	-0,24	1,33	2,30	-0,96	0,03	0,43980
-0,05	12,43	6,17	0,72	6,38	7,26	0,89	0,06	-0,06325
-0,08	-0,91	-0,10	-0,09	7,46	6,34	-1,12	-0,27	-2,21996
-0,08	4,58	2,10	2,48	8,86	12,86	0,46	-0,27	-0,84466
-0,08	1,47	-1,36	-1,34	1,12	-4,69	5,80	0,00	0,19466
0,01	3,05	-1,70	-1,22	-1,41	1,86	-3,27	0,04	0,07495
-0,03	3,59	-5,04	2,07	-8,50	-7,86	0,64	-0,07	-0,45325
0,09	-5,04	0,00	5,05	-5,58	6,01	11,20	-0,03	0,68742
-0,02	-4,11	-0,05	4,07	1,15	-3,83	-4,98	-0,03	-0,35010
0,42	-3,56	-1,47	2,09	8,10	4,82	3,27	0,07	0,68239
0,03	1,77	3,26	1,49	-2,85	3,34	1,44	0,04	-0,03273
-0,03	5,22	-0,63	3,03	3,41	8,32	-4,91	0,21	0,05098
0,06	5,01	1,29	-3,72	9,58	1,15	-5,55	0,12	1,42777
0,00	-0,49	-1,81	-1,33	-5,21	-0,29	-2,60	-0,06	-0,02698
-0,02	-2,62	-3,10	0,49	-1,81	3,54	5,35	0,05	-0,63287
#LEEG!	#LEEG!	#LEEG!	#LEEG!	#LEEG!	#LEEG!	#LEEG!	#LEEG!	0,06013
-0,03	-2,10	2,16	1,51	-1,48	-1,23	0,25	-0,05	0,88024
0,13	-2,45	-3,25	0,80	-1,89	2,73	-4,62	-0,01	0,48363
-0,10	1,03	9,00	7,96	7,14	6,66	-0,47	-0,42	-1,03893
0,11	-2,91	3,34	6,24	-2,73	1,08	3,81	-0,41	-1,42081
-0,07	3,20	4,13	0,93	11,05	0,52	-2,72	0,03	0,22093
-0,03	-11,64	-3,08	8,55	4,12	3,19	-0,93	0,18	0,59830
-0,01	3,46	-0,18	-3,64	1,56	-0,09	1,65	0,04	0,24483
-0,01	2,60	-3,59	-2,79	-2,11	-6,82	1,17	-0,63	0,13855
#LEEG!	#LEEG!	#LEEG!	#LEEG!	#LEEG!	#LEEG!	#LEEG!	#LEEG!	0,04375
-0,13	-1,88	1,58	3,44	-2,36	-7,46	5,10	-0,88	0,17428
#LEEG!	#LEEG!	#LEEG!	#LEEG!	#LEEG!	#LEEG!	#LEEG!	#LEEG!	-0,89579
0,02	5,19	7,12	-1,80	0,85	0,09	-0,77	-0,69	-0,83121
0,13	-6,91	-2,79	4,01	0,93	0,71	-0,22	-0,57	-0,27906
0,00	-2,98	-3,40	-0,43	4,84	7,35	2,25	-0,73	1,25386
0,04	-1,10	-3,75	-2,65	3,76	4,86	-1,09	-0,74	0,18379

#LEEG!	#LEEG!	#LEEG!	#LEEG!	#LEEG!	#LEEG!	#LEEG!	#LEEG!	1,20611
-0,16	3,25	2,97	-0,29	0,96	-3,23	-0,95	-0,55	-1,25637
0,02	1,38	5,35	1,27	4,14	7,22	3,07	-0,35	-0,57019
-0,18	6,17	4,63	-1,54	5,35	5,31	0,04	-0,64	-0,55396
-0,19	-0,24	-0,57	0,32	-7,48	-3,64	3,84	-0,39	-1,74348
0,05	0,62	-0,29	0,91	8,22	4,40	-3,82	-0,11	0,86607
-0,18	2,64	-0,40	-3,04	4,52	1,83	2,68	0,05	-1,74218
-0,24	-2,60	4,67	-7,27	2,25	-4,23	3,28	-0,23	-1,93484
-0,05	-4,53	-6,19	-1,66	2,62	-5,42	8,04	-0,04	0,44235
0,01	6,94	4,39	2,54	-9,74	-1,90	7,84	-0,19	-1,11009
-0,03	6,45	12,46	3,18	9,81	-3,86	13,66	0,00	-0,99499
#LEEG!	#LEEG!	#LEEG!	#LEEG!	#LEEG!	#LEEG!	#LEEG!	#LEEG!	-0,48264
-0,05	-0,96	-2,33	-1,38	-4,45	-3,09	1,36	-0,10	-0,74112
0,02	2,76	1,12	1,63	9,49	12,57	3,08	0,26	-1,95315
0,02	-2,94	-2,13	0,82	11,88	23,43	4,90	0,05	-0,39540
0,02	-1,22	18,46	-12,28	-1,41	-13,74	2,35	-0,03	-0,52676
-0,24	0,72	-2,29	-3,03	13,18	16,25	3,07	0,13	-1,77835
0,49	-16,17	-8,70	1,01	6,67	8,29	-1,62	0,03	-1,36407
0,20	3,47	-1,14	2,76	6,09	2,70	-3,40	-0,01	-1,53931
-0,08	-0,68	6,59	1,17	4,11	-9,42	-9,69	-0,01	0,13175
0,02	6,58	-4,83	4,79	-4,11	-0,40	3,70	-0,05	-0,41676
0,16	-4,55	-6,05	1,50	17,39	11,33	-3,99	0,08	-0,69630
0,06	-0,95	-0,28	0,67	-9,33	-16,09	-0,53	-0,26	1,14598
0,01	2,32	2,21	-0,11	0,15	-0,14	0,29	-0,04	-0,71011
-0,02	1,42	7,85	-6,45	-6,78	-4,36	-2,44	-0,12	-2,48252
0,10	-1,80	-0,46	1,35	-3,91	4,59	8,50	-0,13	0,22670
-0,03	4,66	-1,38	-6,04	-3,91	0,21	-2,06	-0,07	0,36434
#LEEG!	#LEEG!	#LEEG!	#LEEG!	#LEEG!	#LEEG!	#LEEG!	#LEEG!	-0,32799
0,08	-1,22	-3,81	-1,80	8,94	0,76	8,17	0,01	-0,89249
#LEEG!	#LEEG!	#LEEG!	#LEEG!	#LEEG!	#LEEG!	#LEEG!	#LEEG!	0,36864
0,13	1,35	-4,01	0,88	7,96	14,55	6,59	0,10	-0,21234
0,00	0,39	-1,12	-1,51	-1,88	3,43	-2,09	0,08	-1,14051
-0,16	7,34	3,19	4,14	-1,35	0,89	-2,25	0,06	-2,55132
-0,05	-2,75	1,95	-0,22	17,17	11,75	-1,94	0,24	-2,27500
0,01	-12,29	-5,51	0,75	-7,22	-14,41	4,06	-0,39	-0,03481
-0,05	3,44	7,74	4,30	-9,47	-8,82	0,65	-0,12	0,11039
0,00	-2,43	1,83	-4,14	-4,55	-7,08	-2,53	-0,09	0,13420
0,09	0,51	0,06	-0,45	11,73	-3,19	11,37	0,11	-1,62076
0,07	2,74	6,95	0,33	14,13	8,94	5,19	0,16	-1,91996
0,03	0,90	0,03	-0,87	-1,67	-0,62	-0,74	-0,05	-1,99737
0,01	0,42	4,86	-4,43	-1,06	-7,56	1,22	0,02	-0,05991
0,05	-1,64	-5,56	3,92	2,29	-1,11	-3,40	0,08	-1,37772
0,00	-2,94	-2,93	0,00	2,68	3,25	0,56	0,05	-1,08254
#LEEG!	#LEEG!	#LEEG!	#LEEG!	#LEEG!	#LEEG!	#LEEG!	#LEEG!	0,59085
-0,03	1,18	4,84	-2,69	7,99	-0,85	-4,40	0,13	-0,49129
0,11	-2,32	-2,32	-0,01	-4,03	-1,32	2,71	-0,13	0,09081
-0,06	-7,00	-0,54	-4,44	-2,93	-4,08	1,15	-0,02	0,09438
-0,16	1,52	1,06	-0,46	0,53	-4,54	-5,07	0,03	-0,08864

GSknee	GSdiff_kne	GSdiff_star	GSdiff_hip	BL_Functio	M6_Functi	Change_Fu	BL_Functio	M6_Functi
-4,41792	-0,39314	-0,71918	-1,28016	-1,01922	-0,27273	0,75	0,06986	0,58587
0,03953	-0,06505	-0,48095	-1,41783	-0,72360	-0,51924	0,20	-0,38855	0,12349
0,29282	0,60164	-0,94108	0,61850	0,75272	0,53371	-0,22	0,30768	1,03478
#LEEG!	#LEEG!	#LEEG!	#LEEG!	1,02390	1,00743	-0,02	1,71125	1,26221
1,05367	0,10678	-1,27872	-0,52228	1,10508	1,30133	0,20	1,59209	2,13538
#LEEG!	#LEEG!	#LEEG!	#LEEG!	-0,57233	#LEEG!	#LEEG!	0,06790	#LEEG!
-0,04337	-1,04862	0,58193	-0,58735	-1,42691	-1,30570	0,12	-0,55910	-0,30860
-0,30677	1,26431	-0,11391	0,38724	-1,46877	-1,11233	0,36	-1,17741	-0,73265
#LEEG!	#LEEG!	#LEEG!	#LEEG!	-1,17036	-0,96665	0,20	-0,42865	-0,36036
#LEEG!	#LEEG!	#LEEG!	#LEEG!	-2,71281	#LEEG!	#LEEG!	-0,25555	#LEEG!
-0,78233	-0,76092	-1,25788	-0,61444	-0,72054	-0,68247	0,04	-0,56874	-0,32249
1,02472	-0,50549	0,08294	0,98896	0,36477	-0,40534	-0,77	0,68920	-0,18166
-0,00907	-0,98852	-0,72512	0,60075	-1,78408	-2,09822	-0,31	-0,26112	-0,61973
-1,97348	1,38308	-1,53932	-0,42326	-2,07659	-1,77425	0,30	-1,92703	-1,56145
0,12182	0,62937	-0,95181	0,66084	0,39356	#LEEG!	#LEEG!	0,54774	#LEEG!
-0,82384	-0,33642	0,96968	-0,11659	-2,04591	-1,40567	0,64	-0,93825	-0,69166
#LEEG!	#LEEG!	#LEEG!	#LEEG!	-1,33957	-1,22461	0,11	-1,52854	-0,62685
-0,95196	0,44761	0,87032	1,68753	-2,19062	-2,27388	-0,08	-1,08279	-1,08340
#LEEG!	#LEEG!	#LEEG!	#LEEG!	0,32382	1,51755	1,19	-0,96894	1,30263
-0,19359	-0,62697	0,09551	2,21420	-1,18689	-0,50967	0,68	-0,61930	-0,25830
-0,45595	-1,04314	0,36629	-1,03195	-0,89366	-0,94816	-0,05	-1,22051	-1,19713
0,71597	0,29016	-0,27604	-0,18759	0,07554	#LEEG!	#LEEG!	0,13104	#LEEG!
0,36219	0,95246	-1,12543	-1,32947	-0,66928	-0,78722	-0,12	-0,07220	-0,25295
0,21189	-0,51862	-1,05644	0,00313	-0,64518	-1,07060	-0,43	-0,98552	-0,75612
1,60664	-0,48982	-0,38661	0,41297	-0,83547	-0,95836	-0,12	-0,84166	-1,35562
-0,39887	0,64490	0,54133	-1,03841	0,50855	0,46774	-0,04	0,90023	0,93320
0,50350	-0,75570	1,22341	-1,14171	-0,15304	-0,04198	0,11	0,29443	0,15607
0,09867	0,57998	0,69954	-0,26887	0,48170	-0,01816	-0,50	0,58813	-0,18586
1,70194	-0,42983	0,19123	-0,29187	0,23228	-0,11090	-0,34	0,00559	0,12268
0,34038	0,33746	0,06201	0,87548	0,84782	0,60634	-0,24	1,20879	0,34363
0,40690	0,67785	-0,25805	1,27796	1,23307	1,53872	0,31	2,38714	2,19085
1,11554	0,25517	0,08215	1,57593	0,89617	1,34522	0,45	2,12694	1,00416
0,12650	-0,22141	-1,02873	1,40250	0,35557	1,42321	1,07	0,68783	0,63007
1,82882	-0,24082	-0,98243	0,42108	0,53332	0,22343	-0,31	0,65193	-0,09300
0,21225	-0,78148	-0,25567	1,93118	-0,27273	-0,10797	0,16	-1,18330	-0,25357
-0,14040	1,31804	-0,49499	1,57554	-1,29339	-1,29092	0,00	-0,41655	-0,61512
0,31620	-0,96253	-0,88175	0,16416	1,12179	1,14281	0,02	0,10587	0,87522
-0,07825	-0,69444	-0,25692	-1,50280	0,41146	0,13527	-0,28	-0,10475	-0,16247
-0,54382	-0,94375	0,31878	-0,96075	-0,57725	#LEEG!	#LEEG!	0,28408	#LEEG!
-0,11222	-0,76940	-0,48267	2,09694	-1,04100	#LEEG!	#LEEG!	-0,65493	#LEEG!
2,13520	-0,48081	0,28129	0,93661	0,12132	0,38855	0,27	0,63188	0,60710
0,17403	-0,41402	-1,09036	-1,30157	-0,21866	-0,28035	-0,06	0,10885	-0,34109
-0,38934	-0,37567	1,06756	-0,63436	-0,41292	0,20461	0,62	-0,68887	0,19841
-0,91649	-0,89199	0,16519	-0,45419	1,32628	0,56266	-0,76	1,32264	0,61787
-0,64764	-0,72148	-0,09996	1,25035	0,87871	0,67243	-0,21	0,91559	0,20115
-0,26734	-1,12060	-0,78448	-0,99169	1,35789	0,95551	-0,40	0,26687	0,78450
-1,75378	2,34387	0,37655	5,40023	0,62373	0,15029	-0,47	-0,78404	-1,19858
0,60361	-1,00669	-0,01515	0,20314	-0,12747	0,49624	0,62	-0,47288	0,60508
-0,19397	0,70117	-0,30455	0,81797	0,56961	0,45881	-0,11	0,12703	-0,29630

0,23918	-0,78304	-0,21815	-0,26633	-0,38685	-0,48954	-0,10	-0,42749	-0,50352
-0,08295	-0,94029	-0,61470	0,47064	1,15912	1,00986	-0,15	1,29965	1,41499
-0,63431	0,49805	0,22331	0,13043	0,87393	1,06196	0,19	1,51105	1,34935
1,04091	-0,63492	1,92429	-0,38655	-0,07215	-0,71833	-0,65	-0,62157	-1,50620
1,47292	-1,08184	0,75828	-0,51910	-0,11797	-0,61262	-0,49	-1,03604	-0,72225
-0,48846	-1,16505	-0,19906	1,64870	1,44874	1,19568	-0,25	1,09940	1,01199
-0,22456	0,31785	-1,26814	-0,38466	1,52996	#LEEG!	#LEEG!	2,17245	#LEEG!
1,01055	3,05016	1,96723	-0,99442	#LEEG!	0,16578	#LEEG!	#LEEG!	-0,63565
1,45758	0,77148	-0,50665	-0,59599	-0,49044	-1,09010	-0,60	-0,99446	-1,59062
-0,51828	-0,93456	-1,15023	3,59643	-0,49687	-0,25388	0,24	-0,69413	-0,56790
0,50368	-0,02947	-0,27818	0,08192	0,28219	0,19505	-0,09	-0,70595	-0,77594
-0,40155	0,72824	-0,63596	0,03187	1,11252	1,21787	0,11	1,03693	1,47243
-0,28385	0,64694	-0,60606	0,53967	0,59816	0,18491	-0,41	0,78833	0,58632
0,29751	-0,62516	-0,98808	1,39498	0,58166	0,75006	0,17	0,68192	0,67587
-0,36659	-0,31695	1,65090	-0,21701	1,10162	0,15167	-0,95	0,56534	-0,24617
0,06404	0,39380	0,20954	0,25936	0,21660	-0,39911	-0,62	0,02189	-0,70379
0,18425	-0,75465	-0,35281	1,73470	0,00442	-0,49900	-0,50	0,14259	-0,36934
2,07773	-0,53051	0,40270	-0,51060	0,59963	0,70223	0,10	1,73226	0,78965
1,55298	-0,23593	-0,10697	-0,60308	0,96530	0,50784	-0,46	1,07630	-0,08273
-0,80539	0,65245	2,00534	-1,30258	-0,04936	-0,70196	-0,65	0,54258	-0,50933
1,65806	1,61271	0,21061	1,74668	1,07175	0,44234	-0,63	1,39824	0,32319
-0,69030	-1,44498	0,31849	0,06147	0,41505	-0,03199	-0,45	0,93771	0,07397
0,03902	1,37541	0,46330	-0,64030	-0,66324	-0,37624	0,29	-1,12764	-1,03197
-0,83527	0,98823	-0,01221	2,09569	1,13853	1,30703	0,17	1,43914	1,32185
0,27171	-1,13074	-0,43154	0,40605	0,51528	-0,04911	-0,56	1,09262	0,98391
0,02362	-0,26809	-0,38347	0,01606	-0,22902	-0,09440	0,13	-0,57341	-0,70350
-0,43104	0,68816	0,47273	-0,74821	0,61088	1,07055	0,46	0,66639	1,31749
-0,58765	-0,83686	0,37736	0,05517	-0,12679	#LEEG!	#LEEG!	1,43369	#LEEG!
0,01807	-1,36765	0,37974	0,75920	0,25232	0,04966	-0,20	-0,46246	-1,08243
0,13934	-0,67752	-0,12643	0,90320	0,18165	0,04820	-0,13	0,84831	0,21036
-0,27086	-0,57708	0,33267	0,15898	0,84473	-0,20329	-1,05	1,60708	0,47175
0,27676	-0,31949	-0,38538	0,33551	1,84520	1,68306	-0,16	1,58845	1,12355
-0,64447	-0,69675	-0,52556	0,13461	-1,41927	#LEEG!	#LEEG!	-1,31027	#LEEG!
0,64614	3,74874	0,05108	0,34621	-0,39095	#LEEG!	#LEEG!	-0,50947	#LEEG!
0,32182	2,39851	2,75333	-0,60790	0,57214	0,25492	-0,32	0,28614	0,24408
-1,53939	-0,40342	-0,70298	-0,49077	-0,12284	-0,56343	-0,44	0,30484	-0,32696
1,00312	-0,53399	0,49699	-0,06618	0,32040	0,97142	0,65	0,36574	0,91255
0,58040	0,68742	-0,28899	-0,37473	0,48006	1,11210	0,63	0,29453	1,33773
0,50413	-0,77958	0,29877	-0,10143	-0,31893	0,13163	0,45	-0,02131	-0,33274
1,88183	0,93093	-0,31366	-1,04856	0,35719	0,36704	0,01	0,99646	-0,07373
-1,12491	-0,48805	0,70636	-0,98440	-0,46140	-0,36215	0,10	0,03150	0,03167
-0,54926	-1,04913	-0,85111	0,47677	1,32746	1,41962	0,09	1,68039	1,48166
-1,54193	-1,04462	-0,43003	-0,04244	0,06807	-0,42100	-0,49	0,56564	-0,55015
1,26281	1,52345	0,02687	-0,32673	1,20716	1,29981	0,09	1,02331	1,36603
-0,40192	-0,89448	-0,20037	-0,36784	1,00629	0,00632	-1,00	0,67610	-0,37046
-0,64601	-0,98352	0,03083	-0,57044	1,80871	1,65826	-0,15	0,98206	1,76754
#LEEG!	#LEEG!	#LEEG!	#LEEG!	0,59722	1,37674	0,78	-0,45590	1,04116
1,48770	-1,01805	0,98948	0,69589	1,55222	1,11142	-0,44	1,36538	1,10393
-1,01597	-0,21654	4,96155	-0,18480	#LEEG!	#LEEG!	#LEEG!	#LEEG!	#LEEG!
#LEEG!	#LEEG!	#LEEG!	#LEEG!	1,35259	0,81608	-0,54	1,57315	0,50914

0,67862	0,46568	-0,94905	0,76844	1,33854	0,21109	-1,13	0,99643	0,05241
-1,02304	-1,00815	1,26954	0,22000	#LEEG!	-1,49299	#LEEG!	#LEEG!	-0,86510
-0,26275	-0,98512	1,65620	0,50100	-0,12270	-0,28083	-0,16	-0,44115	-0,56675
-0,26924	-0,92793	-0,42493	-0,01457	0,99479	1,06279	0,07	0,92410	1,22238
-0,24178	-1,07459	0,19966	-0,14846	-0,21584	-0,03674	0,18	0,50450	0,20923
0,84631	-0,11895	1,55322	-1,04630	1,88248	1,96893	0,09	1,55800	1,76333
0,31306	-1,08117	0,87312	-0,59011	0,39694	0,96207	0,57	0,61291	0,73749
-0,07088	1,67925	-0,89798	0,20552	-0,03231	#LEEG!	#LEEG!	-0,34009	#LEEG!
0,21856	-0,49508	0,51717	-0,93270	0,92007	0,51612	-0,40	0,37889	0,27620
0,60366	0,76803	0,63812	0,65652	#LEEG!	1,20028	#LEEG!	#LEEG!	1,44433
1,37128	-0,39042	-0,12282	0,15285	0,05974	0,28105	0,22	0,45254	0,83181
2,16114	0,28828	0,25455	0,96432	1,32474	1,35163	0,03	0,97136	1,40166
1,09088	0,98123	-0,94193	-0,02408	0,83368	#LEEG!	#LEEG!	0,71853	#LEEG!
0,64608	-0,28177	0,57976	-0,87236	0,27965	-0,05026	-0,33	0,48933	0,20246
0,59592	0,36715	-1,34389	-1,02635	0,55347	0,95080	0,40	1,46636	0,66457
1,54929	0,39514	-0,04352	-0,60247	1,97570	1,68421	-0,29	1,88940	2,02382
-0,17552	-0,11569	-0,84096	-1,11066	1,92360	1,76422	-0,16	2,16586	1,94563
-0,15016	2,16540	-1,10135	1,22867	1,33537	0,76837	-0,57	1,34406	0,68019
0,54623	1,16392	-0,28677	-0,14930	1,50858	1,28055	-0,23	1,20612	1,36369
0,41212	0,63826	-1,55461	-0,91051	1,80257	2,14067	0,34	1,85447	1,24474
0,25599	0,09030	-0,48043	-0,86548	0,59888	0,64921	0,05	0,65607	0,37817
2,12951	-0,88945	-1,06468	-0,19118	1,26085	1,20945	-0,05	1,74052	1,12788
0,37800	-0,78778	0,00456	-0,11705	-1,33772	-0,84888	0,49	-0,64136	-0,61022
1,07761	-0,79057	-0,70333	-0,03109	1,61947	1,38305	-0,24	1,90555	1,63525
1,00588	-0,34742	-0,12057	1,09425	1,28266	0,91472	-0,37	0,98894	1,32177
0,13427	2,20227	-1,74714	1,89142	0,06785	0,00997	-0,06	-0,83949	-0,15595
-2,33952	-0,67541	-0,64695	-0,08411	-0,80353	0,17740	0,98	-0,68571	0,25013
-0,35106	-0,89463	-0,89957	-0,96716	-0,13741	-0,24235	-0,10	0,22238	0,06743
-1,71289	0,23362	-0,18372	-0,75935	0,64049	-0,16927	-0,81	0,49414	0,33255
-0,61694	-0,76670	0,39462	-0,13281	-2,15723	-2,53520	-0,38	-1,94876	-2,20102
-0,40093	-1,11792	-0,34108	0,38270	-0,01895	-0,27212	-0,25	-0,90717	-0,65382
0,11952	-1,08377	0,07668	-0,62671	1,66973	1,72406	0,05	2,04214	1,88272
-1,28038	-0,83816	-0,41578	1,00204	-0,60605	-0,12869	0,48	-0,86726	-0,60958
0,24447	-1,18579	-0,74144	1,13678	0,06085	-0,17934	-0,24	-0,32528	-0,42705
-0,19780	-0,26507	-0,17962	-1,48493	1,14736	#LEEG!	#LEEG!	0,34064	#LEEG!
-0,53189	-1,12307	0,55091	-0,85118	-0,15845	-0,37331	-0,21	-0,65475	-0,54139
-1,18660	-0,29498	-0,99825	-1,56711	-0,95156	-1,17075	-0,22	-1,75060	-1,57049
-2,25900	-0,44659	-1,67834	-1,09180	-0,29196	-1,09088	-0,80	-0,03761	-1,79793
1,39953	0,04705	-0,69414	-1,16148	0,48886	0,54939	0,06	0,53743	0,64705
-0,61365	0,56770	-0,52087	0,76465	0,08196	#LEEG!	#LEEG!	0,77726	#LEEG!
1,62166	-0,36984	-1,10781	-0,36510	1,21794	1,14023	-0,08	2,26257	1,20028
-2,01066	0,72382	0,88942	0,50669	0,80534	0,21431	-0,59	-0,55266	-0,17856
-0,30396	-0,51689	0,28272	-1,26622	0,51052	0,39387	-0,12	0,78212	0,63513
0,19159	-0,53218	-0,29577	0,55684	0,33006	#LEEG!	#LEEG!	1,04733	#LEEG!
-0,56927	-1,02293	-0,01630	-0,09547	#LEEG!	1,08399	#LEEG!	#LEEG!	1,81887
-1,35598	-0,23003	-0,31924	-0,97816	-0,69562	-0,92485	-0,23	-0,20802	-1,09121
0,11729	1,35139	0,31835	0,47545	0,26423	0,59394	0,33	0,41058	1,08739
1,31818	1,55693	-0,35174	0,24065	1,51898	1,85878	0,34	1,16061	1,87808
-0,65349	2,66258	0,97475	-1,34631	-0,27745	0,46876	0,75	-0,00115	0,54817
-0,34822	1,10771	0,42313	0,82673	#LEEG!	0,80283	#LEEG!	#LEEG!	0,96673

-0,32076	0,87054	-1,43080	-0,31200	0,40634	0,49086	0,08	1,00936	0,35153
0,96027	1,10523	0,46967	0,49326	#LEEG!	0,12969	#LEEG!	#LEEG!	-0,13268
-1,28287	0,03990	0,37214	-1,60651	1,51138	1,39820	-0,11	0,63541	1,00146
0,09393	1,02587	0,68151	-0,23333	#LEEG!	-0,53703	#LEEG!	#LEEG!	-1,25474
-2,13954	3,13058	-0,01023	-1,33009	-0,95778	#LEEG!	#LEEG!	-1,00792	#LEEG!
0,73153	0,99513	-0,77126	-0,30689	0,54222	-0,65823	-1,20	1,11839	-1,35649
-0,30901	-0,26810	0,02380	-0,11725	1,42077	1,53630	0,12	1,30198	1,12593
1,22810	0,78979	1,06951	-0,16489	0,95794	0,51578	-0,44	1,10260	0,21900
0,81541	-1,03961	1,44335	0,04449	1,38722	0,02854	-1,36	0,30636	-0,36330
#LEEG!	#LEEG!	#LEEG!	#LEEG!	0,66853	1,04013	0,37	1,09711	1,76102
0,00345	-0,78299	0,29164	0,97898	-1,49004	-1,26977	0,22	-1,09453	-1,54559
-0,17857	1,82584	-0,60469	0,67793	0,20495	0,18281	-0,02	-0,49576	0,39994
#LEEG!	#LEEG!	#LEEG!	#LEEG!	-0,64797	-0,32525	0,32	-0,61788	0,04622
1,61331	0,18408	-0,80688	-1,20475	0,35128	0,52260	0,17	-0,89705	-0,18752
-1,02579	0,91301	0,42663	-0,07903	0,37026	1,26999	0,90	0,44021	1,60659
1,39387	0,67850	-0,64409	-0,62294	0,26627	0,80138	0,54	-0,31689	-0,02520
0,18226	0,11206	-0,65599	0,10565	-0,14066	-0,26292	-0,12	-0,74481	0,32749
-0,98315	0,25004	1,20334	0,16134	1,71718	1,40289	-0,31	2,35303	1,51306
-0,19381	3,33486	-0,14043	-0,54817	0,90822	1,26892	0,36	0,52361	1,42383
-1,61295	-0,23034	-0,60047	-0,53976	-0,15307	0,45181	0,60	-0,71200	0,23500
-0,51489	-1,16393	-0,74476	-1,19015	-0,78623	-0,21138	0,57	-1,54086	-0,44629
0,99416	-0,21239	-0,19742	1,46356	1,43840	0,60036	-0,84	0,67270	0,31211
-0,65551	0,22881	-1,34454	-1,83277	0,88482	-0,17119	-1,06	0,12365	-0,19666
-1,86636	-0,79389	-0,89428	0,78178	0,13458	0,13957	0,00	-0,03137	0,47901
0,05263	-0,56279	0,66794	0,88302	0,55662	0,58406	0,03	-1,79964	0,23020
-1,07771	-0,13648	0,02509	-0,32785	-0,54579	0,20276	0,75	-0,00032	0,28589
-0,06793	-0,65346	-0,67995	-1,01765	-1,37973	#LEEG!	#LEEG!	-0,69062	#LEEG!
0,15405	-0,85410	-0,23143	0,35328	0,97108	0,31035	-0,66	-0,10729	0,57719
-0,58677	1,60485	0,66585	-0,92241	#LEEG!	-0,61892	#LEEG!	#LEEG!	-0,76207
#LEEG!	#LEEG!	#LEEG!	#LEEG!	-0,39052	-0,74739	-0,36	-1,97941	-0,82008
0,71043	-0,62506	0,88058	0,03527	-0,30060	-0,66701	-0,37	-0,27670	-0,67277
1,03377	-0,96950	-0,68342	1,72554	0,83782	0,82646	-0,01	1,08194	0,76599
1,32050	1,39911	0,96284	-0,53605	-0,04905	0,87849	0,93	-1,00404	1,09822
-0,67818	-0,96937	-0,21046	-0,75294	1,74425	1,73357	-0,01	1,51177	1,48993
1,33129	-0,21705	0,94331	0,48141	-0,58255	-0,89783	-0,32	-1,27709	-1,35855
1,03014	-0,02066	-0,93719	-1,04582	0,13782	0,08407	-0,05	0,05924	-0,18428
-0,55956	0,52249	-0,00482	0,30514	-0,89285	-0,69249	0,20	-1,69540	-1,21270
-0,02029	-0,41794	-0,72461	0,16005	-0,79464	#LEEG!	#LEEG!	-0,47419	#LEEG!
0,65295	-0,61037	-1,07051	0,13613	-0,01335	0,05378	0,07	0,36503	0,37692
-0,31377	0,56583	-0,69069	1,59888	-0,16557	0,54396	0,71	-1,63274	0,42290
-0,25750	1,49662	0,72579	-1,93573	0,25301	0,00047	-0,25	-1,28354	0,65426
0,68773	0,79411	-0,75331	-1,47205	-0,39126	-0,57767	-0,19	0,04142	-0,29065
0,13484	-0,73548	-0,69228	-0,66130	-0,12706	-0,18040	-0,05	1,27319	0,37389
#LEEG!	#LEEG!	#LEEG!	#LEEG!	-0,93987	-1,23784	-0,30	-1,34495	-1,12806
0,48204	0,37333	-1,27412	-0,93488	-1,23130	-1,28041	-0,05	-0,50517	-0,30177
1,00742	0,09670	-0,94088	-0,37037	-0,08847	0,11968	0,21	-0,28767	-0,68092
#LEEG!	#LEEG!	#LEEG!	#LEEG!	-0,16136	#LEEG!	#LEEG!	0,12564	#LEEG!
-0,32977	-0,50391	-0,81037	-1,31845	-1,01310	-0,61796	0,40	-1,44480	-0,97352
#LEEG!	#LEEG!	#LEEG!	#LEEG!	-1,93029	-1,53799	0,39	0,04473	-0,58233
0,91538	-0,20078	-0,61266	-0,51316	1,14350	0,88720	-0,26	1,40563	0,61245

1,12717	-0,14928	2,71938	-0,51220	0,19214	-0,24402	-0,44	1,28205	0,17204
2,01409	1,58937	0,07049	0,41023	0,68625	0,69789	0,01	0,85671	0,87665
0,37586	0,35252	0,13021	0,10971	-0,37671	-1,08672	-0,71	-1,01539	-1,76785
0,46166	0,24089	-0,70702	0,91438	1,37712	1,05093	-0,33	1,10061	0,80892
-1,56703	-1,07361	2,11781	-0,11686	0,82944	1,05423	0,22	1,13028	1,13500
0,82482	0,21681	-1,18056	-1,52163	-2,86183	-2,50527	0,36	-2,55820	-2,32495
-0,14214	-0,08891	-0,34112	-0,78071	-0,29722	0,45331	0,75	-0,40007	0,73285
0,18762	-0,10372	-0,28587	-0,11221	-0,40821	0,33230	0,74	0,34895	0,59020
1,70996	-1,00681	-0,65629	-0,25202	-0,10046	1,00932	1,11	0,21312	0,52510
-0,62800	0,70896	-1,10973	-0,00017	0,23040	-0,38256	-0,61	0,09883	-0,04770
0,28042	-1,08119	0,56883	0,06602	0,79188	0,97930	0,19	0,67995	1,43725
0,75209	0,26352	0,30572	-0,64110	0,06187	0,07023	0,01	0,34521	0,11876
0,34214	-0,33793	-0,25886	0,07231	0,96871	0,41103	-0,56	1,34782	0,20623
-0,36528	-0,79062	-0,78216	-1,06402	1,39678	1,23718	-0,16	1,08200	1,05886
0,15494	0,29224	-0,03519	-0,04594	1,32027	1,14342	-0,18	0,97309	1,25037
-0,02447	0,91570	-0,41322	-0,00485	1,08986	1,21705	0,13	1,76080	1,20625
-1,00940	0,45705	1,09719	-0,71873	-0,45831	-0,71881	-0,26	-0,58083	-0,39370
-0,08057	-0,47987	-0,54449	-0,75035	0,47260	0,57638	0,10	0,52075	0,94496
0,03124	-0,93155	0,19505	0,61192	-1,07143	-0,71123	0,36	-1,14684	-0,83926
-2,47662	-0,36438	0,21469	0,02966	-2,58197	-2,28247	0,30	-2,14020	-1,67408
0,05266	-0,52770	-0,59533	-0,45268	0,14672	-0,33146	-0,48	-0,16030	0,01794
0,24609	0,26681	-0,73258	-0,03006	-1,98810	-2,73367	-0,75	-1,52052	-2,23451
1,17764	-1,18064	-0,70363	0,14592	0,28486	0,25211	-0,03	0,74481	0,26052
0,91195	-0,37263	-0,94641	-1,13967	0,70936	0,29212	-0,42	-0,04539	-0,56530
-0,81843	-0,30419	0,02162	-0,15103	-0,52559	-0,21940	0,31	-0,89147	-0,33634
0,14106	-0,74824	-0,30657	-0,42132	1,12867	0,70337	-0,43	1,29887	0,83422
0,52411	-0,93450	-0,08375	-1,10670	-0,01503	0,65612	0,67	-1,08240	0,61162
0,80250	2,05665	0,18315	2,37491	-1,20420	-1,00443	0,20	-1,74793	-0,77187
-1,31442	-0,80905	0,67791	-1,32889	-0,15940	-1,04665	-0,89	-0,02496	-0,96032
0,29444	-0,80568	0,33133	-0,57003	1,05795	-0,06400	-1,12	-0,24375	-0,54333
-1,55911	-0,85954	-0,75535	0,90913	0,00639	-0,16274	-0,17	0,18980	-0,06204
-1,38902	-0,56321	3,37161	-0,10944	0,49678	0,55382	0,06	-0,85240	-0,67933
0,17336	-1,34580	-0,04004	-0,10556	-0,34108	-0,49422	-0,15	-1,58848	-1,18672
0,81944	0,69786	-1,67084	-1,09116	0,87611	0,67593	-0,20	0,05910	0,41788
-0,01647	0,43233	1,59568	-1,13315	-1,63550	#LEEG!	#LEEG!	-1,80537	#LEEG!
-0,00353	-0,29757	-0,51968	1,33070	0,74940	-0,22713	-0,98	0,01888	-0,35435
-0,33618	-0,24541	-0,95302	1,35690	-0,83056	-0,02505	0,81	-1,56567	-0,06037
1,60718	0,37376	0,21122	0,59327	0,35066	0,56923	0,22	-0,46770	-0,05604
-1,03279	-0,83429	0,01330	-0,78489	-1,41795	#LEEG!	#LEEG!	-1,85255	#LEEG!
0,37011	0,13637	0,15134	1,27828	0,36008	-0,01038	-0,37	0,28742	0,21599
1,16082	0,53088	-0,33631	0,01236	1,10003	-0,70038	-1,80	0,31016	-1,10050
0,10358	-0,25380	0,76094	-0,62570	1,28963	0,63745	-0,65	0,85239	0,36207
0,23540	-0,65151	-0,08602	-1,27887	-0,76061	0,24688	1,01	-1,77939	-1,01550
1,64840	1,03259	-0,08249	-0,53369	1,43604	1,61614	0,18	-0,00069	-0,51567
1,48796	0,46887	-0,53189	0,33224	1,11555	1,07479	-0,04	-0,17942	0,26030
-0,60915	-1,02296	0,18349	2,84856	-1,29105	-0,22983	1,06	-0,80965	-0,80480
1,03826	-0,91311	0,61835	0,82189	-0,61063	0,24605	0,86	-1,96431	-0,60498
0,97455	-0,77289	-1,59887	-0,98438	0,72561	0,23650	-0,49	0,57418	0,15215
-0,00224	0,52956	0,23076	-1,05082	0,53469	-0,32149	-0,86	-0,24308	-1,17688
1,96360	-0,51589	3,57603	0,19559	-0,18988	-0,55050	-0,36	-0,32004	-1,07576

0,72975	3,20428	0,05533	-0,45994	-0,58665	#LEEG!	#LEEG!	-1,89232	#LEEG!
0,36204	-1,28516	0,29649	0,60877	0,12247	-0,67228	-0,79	-0,64681	-1,22522
0,36725	-0,64551	-0,90018	-1,30032	-1,61860	-1,28838	0,33	-0,27172	-1,52535
-0,15657	0,00547	1,19019	0,84492	0,83757	1,32338	0,49	-0,08715	0,43704
-1,63651	2,05954	-1,42570	1,91176	-2,49689	-2,61429	-0,12	-1,41625	-1,50692
-0,65633	0,66458	-1,08323	0,38983	0,03288	-0,68999	-0,72	-0,82220	-1,08494
0,82700	-0,48965	3,58362	0,66245	-0,80432	-0,30697	0,50	-0,37133	-0,73720
1,24863	-0,87669	3,03361	0,21347	-1,33685	-1,69168	-0,35	0,59480	-0,81131
0,81236	-0,26766	0,50737	0,74993	0,03505	0,20060	0,17	0,12180	0,51610
0,48048	-0,54941	-0,35565	1,04051	-0,33363	-0,64501	-0,31	-0,14966	-0,66064
-0,39041	0,11120	0,29706	-0,34024	-0,57990	-0,74949	-0,17	-0,62815	-0,56905
1,08897	-1,32048	3,33538	-0,78132	0,30597	#LEEG!	#LEEG!	0,29622	#LEEG!
0,26589	-0,43474	-0,50927	0,89791	-0,37318	-1,60869	-1,24	-0,44256	-1,17165
0,22095	-0,43703	-0,08126	0,30046	-2,35140	-0,90614	1,45	-0,41111	-0,46682
-0,84601	0,44074	-1,06392	-0,82096	-0,95795	-1,03314	-0,08	-1,03340	-0,79348
-1,35426	1,88197	3,89723	-0,54360	-1,19353	-1,71126	-0,52	-1,19088	-2,12272
0,49975	2,66551	1,61150	-0,14483	-1,55253	-0,94521	0,61	-0,83416	-1,18609
1,19274	-0,51270	-0,54204	-0,86259	0,10149	0,00352	-0,10	0,02165	0,94224
-0,93478	1,56997	-0,82360	0,61852	-1,96194	-2,47732	-0,52	-0,06367	-2,04214
-1,01704	-1,19724	0,14902	2,32862	-1,07335	-0,61108	0,46	-0,69483	-0,69626
-1,43652	0,25069	0,48277	-0,77357	-0,48101	0,71611	1,20	-0,10759	1,10316
0,63898	1,42771	0,14097	-0,56451	0,63252	0,55734	-0,08	-0,66278	0,27978
-2,01378	1,47367	0,26660	0,50926	-0,44758	-2,28649	-1,84	-0,23470	-1,94975
-2,04902	0,03236	0,13278	-0,37456	-1,51556	-1,59628	-0,08	-0,45303	-1,14094
0,22314	-0,37847	1,48038	1,49843	-1,25578	-2,63961	-1,38	-0,61831	-1,57714
-1,11876	3,50287	-1,29131	0,12597	-1,03697	-1,91722	-0,88	-0,87451	-1,98757
-1,46461	2,50758	2,36784	0,18135	-1,11629	0,25561	1,37	-0,92259	0,31717
-2,17489	0,02961	-0,42720	0,85472	-1,49945	#LEEG!	#LEEG!	-0,68379	#LEEG!
-1,41648	-0,53666	-0,26922	-1,13447	-0,48911	0,25332	0,74	-0,17162	0,13407
-1,36045	0,51306	-0,86294	0,06408	-0,58214	-1,23587	-0,65	-0,52459	0,62725
-0,16400	-0,70942	0,30097	0,03107	-0,70927	-1,16245	-0,45	-0,79830	-1,46584
-0,50719	0,78096	-0,32207	0,33464	-0,40948	-0,41677	-0,01	-0,51948	-0,14759
0,18106	-1,38145	0,30377	1,05436	-0,90157	-2,04697	-1,15	-0,74268	-2,06844
0,65202	-1,15921	-0,77603	-0,28029	-1,00655	-2,20023	-1,19	-0,02010	-2,51035
0,64115	-0,32961	-0,59575	-1,10352	0,06685	-0,44883	-0,52	-0,85631	0,07764
-0,39500	-1,08257	0,47088	1,31027	-0,85231	-0,47852	0,37	-1,05074	-0,99245
-2,13858	0,12473	-0,47268	-0,24785	-1,50723	-0,54113	0,97	-0,66996	-0,66722
-2,41434	-0,62090	0,30420	-0,32763	-1,44964	-1,64217	-0,19	-0,39869	-0,69326
-0,62292	0,19105	-0,32347	-1,22889	-1,06984	-0,64665	0,42	-0,89166	-0,84861
-0,94666	-0,81753	-0,35348	-0,35501	-1,43891	-0,84260	0,60	-0,48676	-0,44344
-0,81568	0,05278	0,65762	0,51065	-0,91384	-0,22174	0,69	-0,58937	-0,03201
0,67975	-0,07188	-0,19096	0,84241	-0,32645	0,04501	0,37	-0,26134	0,86429
0,16008	-0,68322	-0,87870	-1,10020	-0,04297	-0,86951	-0,83	-0,84299	-1,35428
-0,94809	-0,41752	2,68792	-1,39267	-0,31073	#LEEG!	#LEEG!	-1,41918	#LEEG!
-2,09623	-0,32538	-0,07309	0,98258	-2,01349	-1,69184	0,32	-1,68277	-1,98849
-0,73485	-0,07010	-0,93851	-0,59249	-1,15943	-1,82677	-0,67	-1,33622	-1,83064
0,36535	0,81953	0,53099	-1,02145	-0,94069	0,63028	1,57	-0,83230	1,29947
-0,62679	-1,26878	1,63211	4,08410	-1,24159	1,33401	2,58	-1,03402	2,37315

Change_Fu	BL_Functio	M6_Functi	Change_Fu	Interaction	Interaction	Interaction	BL_GS_tota	BL_GS_obj
0,52	-1,64080	-1,62244	0,02	8,68	2,52	1,74	-1,94	-1,78
0,51	-0,65727	-1,29588	-0,64	-0,06	2,31	0,00	-0,42	-0,56
0,73	0,79552	-0,76796	-1,56	0,17	0,35	0,18	0,23	0,44
-0,45	-0,41556	-0,20046	0,22	#LEEG!	#LEEG!	#LEEG!	#LEEG!	#LEEG!
0,54	-0,15327	-1,17167	-1,02	0,80	-0,40	0,11	0,94	0,62
#LEEG!	-0,95459	#LEEG!	#LEEG!	#LEEG!	#LEEG!	#LEEG!	#LEEG!	#LEEG!
0,25	-1,53606	-2,13947	-0,60	0,03	0,39	0,05	-0,36	-0,17
0,44	-0,88377	-0,97350	-0,09	-0,01	0,02	-0,39	-0,18	0,51
0,07	-1,29456	-1,34551	-0,05	#LEEG!	#LEEG!	#LEEG!	#LEEG!	#LEEG!
#LEEG!	-3,85573	#LEEG!	#LEEG!	#LEEG!	#LEEG!	#LEEG!	#LEEG!	#LEEG!
0,25	-0,44382	-0,82691	-0,38	1,08	0,85	0,60	-0,54	-0,55
-0,87	-0,24022	-0,50897	-0,27	-0,52	-0,51	-0,52	0,38	-0,06
-0,36	-2,42792	-3,21426	-0,79	0,01	-0,59	0,01	-0,34	-0,58
0,37	-0,94553	-0,84271	0,10	1,39	0,30	-2,73	-0,92	-0,44
#LEEG!	-0,03226	#LEEG!	#LEEG!	0,20	1,10	0,08	0,94	0,84
0,25	-2,04414	-1,65356	0,39	0,84	0,12	0,28	-0,65	-0,33
0,90	-0,27924	-1,39667	-1,12	#LEEG!	#LEEG!	#LEEG!	#LEEG!	#LEEG!
0,00	-2,09816	-2,73899	-0,64	0,10	-0,18	-0,43	-0,65	-0,24
2,27	1,61817	0,78029	-0,84	#LEEG!	#LEEG!	#LEEG!	#LEEG!	#LEEG!
0,36	-1,09897	-0,58597	0,51	0,12	-1,38	0,12	-0,27	-0,38
0,02	0,04633	0,20525	0,16	0,08	0,19	0,48	0,05	-0,16
#LEEG!	-0,03621	#LEEG!	#LEEG!	-0,94	0,25	0,21	-0,14	-0,57
-0,18	-0,94065	-1,16899	-0,23	-0,36	1,33	0,34	-0,34	-0,26
0,23	0,15437	-0,84486	-1,00	0,09	0,00	-0,11	0,30	0,32
-0,51	-0,30353	0,47067	0,77	-1,66	-0,43	-0,79	0,04	0,05
0,03	-0,26467	-0,72062	-0,46	0,12	0,32	-0,26	-0,27	-0,15
-0,14	-0,57533	-0,36883	0,21	-0,27	0,61	-0,38	-0,10	-0,15
-0,77	0,05583	0,29845	0,24	0,01	-0,02	0,06	0,06	0,17
0,12	0,34902	-0,45086	-0,80	-1,14	0,20	-0,73	0,33	0,44
-0,87	-0,10291	0,63143	0,73	-0,01	-0,03	0,11	-0,06	0,07
-0,20	-0,87851	-0,78157	0,10	-0,28	-0,88	0,28	-0,36	-0,21
-1,12	-1,09264	0,96380	2,06	-1,44	-2,03	0,28	0,14	-0,07
-0,06	-0,25272	1,80114	2,05	-0,08	-0,88	-0,03	-0,14	-0,21
-0,74	0,06093	0,62967	0,57	1,21	0,28	-0,44	0,94	0,63
0,93	0,95352	0,23533	-0,72	-0,61	-5,54	-0,17	-1,38	-1,25
-0,20	-1,49686	-1,55489	-0,06	0,05	-0,53	-0,19	-0,41	-0,01
0,77	1,59417	0,77874	-0,82	0,30	0,16	-0,30	0,68	0,67
-0,06	0,75107	0,57314	-0,18	0,04	0,86	0,05	-0,14	0,01
#LEEG!	-1,21257	#LEEG!	#LEEG!	0,90	1,59	0,51	-0,69	-0,90
#LEEG!	-0,83442	#LEEG!	#LEEG!	-0,09	1,73	0,09	0,56	0,18
-0,02	-0,54640	-0,29474	0,25	-1,99	-0,87	-1,03	0,28	0,07
-0,45	-0,46076	0,03741	0,50	-0,08	0,61	-0,07	-0,05	-0,02
0,89	0,16614	0,06403	-0,10	-0,16	-0,26	0,15	-0,16	0,22
-0,70	0,49743	0,04548	-0,45	-1,42	-0,71	0,82	0,39	0,46
-0,71	0,28406	1,02550	0,74	-0,31	0,60	0,47	0,23	0,07
0,52	1,76900	0,55580	-1,21	-0,32	-1,20	0,30	0,57	0,43
-0,41	1,86294	2,47705	0,61	1,53	-4,71	-4,11	-1,58	-0,57
1,08	0,35277	-0,06862	-0,42	0,32	0,11	-0,61	0,47	0,14
-0,42	0,72459	1,48340	0,76	0,28	-1,18	-0,14	-0,56	-0,59

-0,08	-0,09677	-0,10111	0,00	-0,13	0,15	-0,19	-0,19	-0,23
0,12	0,26824	-0,47162	-0,74	-0,09	0,49	0,08	0,62	0,37
-0,16	-0,41314	-0,24533	0,17	0,48	-0,10	-0,32	-0,27	-0,58
-0,88	0,60970	1,23872	0,63	-0,76	0,28	-0,66	-0,14	0,04
0,31	1,01976	0,04003	-0,98	0,51	-0,18	-1,59	0,54	0,32
-0,09	0,94350	0,64074	-0,30	-1,25	4,20	0,57	1,14	0,68
#LEEG!	-0,17539	#LEEG!	#LEEG!	0,41	0,71	-0,07	-0,65	-0,68
#LEEG!	#LEEG!	1,49150	#LEEG!	0,52	-0,52	3,08	0,85	0,70
-0,60	0,40155	0,62331	0,22	0,47	-0,19	1,12	0,44	0,48
0,13	0,04376	0,50208	0,46	-0,05	0,35	0,48	0,15	-0,28
-0,07	1,24976	1,80556	0,56	0,59	0,10	-0,01	0,73	0,62
0,44	0,50130	-0,14574	-0,65	-0,40	0,03	-0,29	0,46	0,42
-0,20	0,00212	-0,67786	-0,68	-0,19	0,36	-0,18	0,12	0,45
-0,01	0,10016	0,32775	0,23	0,54	2,53	-0,19	0,84	0,69
-0,81	1,03099	0,75830	-0,27	-0,15	-0,09	0,12	-0,20	0,10
-0,73	0,30613	0,44772	0,14	0,02	0,07	0,03	0,28	-0,03
-0,51	-0,15843	-0,36320	-0,20	0,22	2,03	-0,14	0,77	0,73
-0,94	-1,08923	0,02326	1,11	-1,87	0,46	-1,10	0,51	0,25
-1,16	0,23037	1,19864	0,97	-0,07	0,03	-0,37	0,54	0,54
-1,05	-0,70416	-0,52942	0,17	-0,34	-0,56	-0,53	-0,06	0,00
-1,08	0,02031	0,32959	0,31	-0,45	-0,47	2,67	0,69	0,30
-0,86	-0,45118	-0,19979	0,25	0,63	-0,06	1,00	-0,50	-0,64
0,10	0,29139	1,08819	0,80	0,00	-0,05	0,05	-0,12	0,29
-0,12	0,07513	0,31065	0,24	-0,90	2,26	-0,83	-0,10	0,27
-0,11	-0,47726	-1,87998	-1,40	0,20	0,30	-0,31	0,51	0,05
-0,13	0,31384	1,07666	0,76	0,00	0,00	-0,01	-0,10	-0,11
0,65	0,16287	-0,16998	-0,33	-0,22	-0,38	-0,30	0,03	0,18
#LEEG!	-1,85506	#LEEG!	#LEEG!	0,79	-0,07	0,49	-0,75	-0,77
-0,62	0,92196	2,05923	1,14	0,00	0,12	-0,02	0,04	-0,12
-0,64	-0,70482	-0,28067	0,42	-0,10	-0,66	-0,09	-0,32	-0,37
-1,14	-0,56909	-1,27271	-0,70	0,38	-0,22	0,16	-0,69	-0,79
-0,46	0,98366	1,44589	0,46	0,34	0,41	-0,09	1,01	0,47
#LEEG!	-0,65408	#LEEG!	#LEEG!	0,33	-0,07	0,45	-0,36	-0,49
#LEEG!	-0,00808	#LEEG!	#LEEG!	-0,54	-0,29	2,42	-0,40	0,40
-0,04	0,54412	0,08540	-0,46	0,02	-0,03	0,77	0,56	0,31
-0,63	-0,54118	-0,57291	-0,03	0,19	0,06	0,62	-0,38	-0,05
0,55	0,06662	0,35720	0,29	0,47	-0,03	-0,54	0,45	0,50
1,04	0,39347	-0,12075	-0,51	-0,05	0,03	0,40	0,13	0,27
-0,31	-0,46343	0,87339	1,34	0,22	-0,05	-0,39	0,43	0,27
-1,07	-0,60780	0,89151	1,50	0,62	-0,35	1,75	0,91	0,72
0,00	-0,74265	-0,80537	-0,06	-0,09	-0,08	0,55	-0,24	-0,45
-0,20	0,08477	0,25435	0,17	-0,50	0,43	0,58	0,33	0,44
-1,12	-0,55115	0,12476	0,68	0,76	0,02	1,61	-0,74	-0,56
0,34	0,66193	0,21587	-0,45	0,67	-0,17	1,92	0,57	0,76
-1,05	0,75677	0,68270	-0,07	-0,22	-0,20	0,36	0,27	-0,04
0,79	1,63036	0,23056	-1,40	-1,14	-1,00	0,64	0,72	0,47
1,50	1,44220	0,96203	-0,48	#LEEG!	#LEEG!	#LEEG!	#LEEG!	#LEEG!
-0,26	0,79371	0,30046	-0,49	0,00	0,00	-1,51	0,51	0,40
#LEEG!	#LEEG!	#LEEG!	#LEEG!	-0,12	-0,02	0,22	-0,66	-0,20
-1,06	0,24747	0,76553	0,52	#LEEG!	#LEEG!	#LEEG!	#LEEG!	#LEEG!

-0,94	0,89414	0,34133	-0,55	-0,34	-0,39	0,32	-0,16	0,18
#LEEG!	#LEEG!	-1,52045	#LEEG!	0,31	-0,07	1,03	-0,37	-0,43
-0,13	0,32331	0,44434	0,12	-0,58	1,11	0,26	0,94	0,82
0,30	0,45184	-0,01409	-0,47	-0,12	-0,01	0,25	0,23	0,23
-0,30	-0,91483	-0,45410	0,46	-0,20	-0,12	0,26	0,47	0,22
0,21	1,07599	0,87997	-0,20	0,81	-1,00	-0,10	0,51	0,55
0,12	-0,10260	0,65434	0,76	0,22	-0,42	-0,34	0,42	0,25
#LEEG!	0,34457	#LEEG!	#LEEG!	-0,04	0,11	-0,12	0,23	0,47
-0,10	0,96915	0,56692	-0,40	0,35	-1,48	-0,11	0,81	0,89
#LEEG!	#LEEG!	-0,13129	#LEEG!	0,55	0,60	0,46	0,90	0,42
0,38	-0,43286	-0,92301	-0,49	-0,84	-0,09	-0,54	0,32	-0,13
0,43	0,90207	0,25851	-0,64	-1,45	-0,65	0,62	0,35	0,31
#LEEG!	0,44343	#LEEG!	#LEEG!	1,58	-0,03	1,07	1,16	0,76
-0,29	-0,13893	-0,46978	-0,33	-0,48	0,65	-0,18	0,08	-0,21
-0,80	-0,85181	0,76286	1,61	0,17	-0,30	0,22	0,46	0,31
0,13	0,83471	-0,17908	-1,01	0,69	-0,27	0,61	0,94	0,69
-0,22	0,43468	0,12756	-0,31	-0,11	-0,73	0,02	0,31	0,44
-0,66	0,48654	0,35777	-0,13	-0,10	0,86	-0,33	0,09	0,52
0,16	0,91144	0,18030	-0,73	-0,08	0,02	0,64	0,36	0,25
-0,61	0,61021	2,17217	1,56	0,19	-0,42	0,26	0,64	0,40
-0,28	0,15645	0,65754	0,50	0,11	-0,36	0,02	0,20	0,31
-0,61	-0,08684	0,45969	0,55	-0,76	0,07	-1,89	0,73	0,37
0,03	-1,30426	-0,65057	0,65	0,03	-0,01	-0,30	0,17	0,00
-0,27	0,27082	-0,09882	-0,37	0,55	-0,02	-0,85	0,58	0,39
0,33	0,81729	-0,49964	-1,32	0,48	0,52	-0,35	0,71	0,31
0,68	1,07644	0,30248	-0,77	0,30	4,26	0,30	1,12	0,91
0,94	-0,43531	-0,08568	0,35	2,70	0,10	1,58	-1,12	-0,96
-0,15	-0,46795	-0,62253	-0,15	0,20	0,55	0,31	-0,17	-0,08
-0,16	0,40774	-0,95081	-1,36	0,61	0,27	-0,40	-0,66	-0,50
-0,25	-1,04376	-1,25859	-0,21	0,11	0,02	0,47	-0,34	-0,28
0,25	1,02201	0,61972	-0,40	0,07	-0,07	0,45	-0,04	-0,07
-0,16	0,18948	0,15829	-0,03	0,25	-1,32	-0,13	1,16	0,80
0,26	0,07726	0,83603	0,76	0,61	-0,48	1,07	-0,51	-0,47
-0,10	0,46999	0,40147	-0,07	0,35	1,64	-0,29	0,64	0,44
#LEEG!	1,36132	#LEEG!	#LEEG!	-0,20	-1,47	0,05	0,37	0,91
0,11	0,51607	0,20746	-0,31	-0,45	-0,72	0,60	0,32	0,35
0,18	0,57186	0,42034	-0,15	-0,97	-1,28	0,35	0,11	0,13
-1,76	-0,40325	0,99647	1,40	-6,29	-3,04	1,01	0,78	0,58
0,11	0,12552	-0,03470	-0,16	-0,85	0,70	0,07	0,48	0,28
#LEEG!	-0,77506	#LEEG!	#LEEG!	-0,96	1,19	-0,35	0,14	0,16
-1,06	-0,75734	0,18583	0,94	-0,21	0,05	-0,60	0,54	0,60
0,37	1,87282	0,76549	-1,11	-2,50	0,63	-1,46	-0,21	0,22
-0,15	-0,12482	-0,33442	-0,21	-0,23	-0,97	0,16	0,48	0,16
#LEEG!	-0,70826	#LEEG!	#LEEG!	0,09	0,27	-0,10	0,35	0,20
#LEEG!	#LEEG!	-1,04854	#LEEG!	-0,40	-0,07	0,58	0,20	0,04
-0,88	-0,82360	0,06196	0,89	0,20	0,15	0,31	-0,66	-0,17
0,68	-0,07129	-0,73864	-0,67	-0,03	-0,13	0,16	0,07	-0,22
0,72	0,98009	0,44500	-0,54	0,74	0,14	2,05	0,53	0,77
0,55	-0,42329	-0,02252	0,40	0,60	1,25	-1,74	-0,73	-0,14
#LEEG!	#LEEG!	-0,08899	#LEEG!	0,05	-0,12	-0,39	-0,44	-0,09

-0,66	-0,54752	0,37858	0,93	0,27	0,26	-0,28	-0,34	-0,20
#LEEG!	#LEEG!	0,50776	#LEEG!	1,66	0,85	1,06	0,84	0,88
0,37	1,57693	1,07812	-0,50	-3,12	-3,90	-0,05	0,91	0,86
#LEEG!	#LEEG!	1,15872	#LEEG!	-0,01	0,03	0,10	-0,18	0,30
#LEEG!	-0,29809	#LEEG!	#LEEG!	1,58	0,98	-6,70	-1,12	-0,39
-2,47	-0,46590	1,09225	1,56	-0,66	0,28	0,73	0,03	-0,21
-0,18	0,66599	1,13844	0,47	-0,30	-0,12	0,08	0,50	0,35
-0,88	0,18865	0,66962	0,48	3,14	-0,42	0,97	1,43	0,92
-0,67	1,76812	0,71567	-1,05	-0,70	-0,04	-0,85	0,03	0,11
0,66	-0,24791	-1,03457	-0,79	#LEEG!	#LEEG!	#LEEG!	#LEEG!	#LEEG!
-0,45	-1,01233	0,17077	1,18	0,00	-0,38	0,00	-0,17	-0,49
0,90	0,88803	-0,34530	-1,23	0,18	-0,69	-0,33	-0,64	-0,19
0,66	-0,27583	-0,75546	-0,48	#LEEG!	#LEEG!	#LEEG!	#LEEG!	#LEEG!
0,71	1,57691	1,41857	-0,16	-1,44	1,08	0,30	0,20	0,18
1,17	0,05665	-0,28058	-0,34	0,59	0,05	-0,94	-0,85	-0,32
0,29	0,77464	1,70104	0,93	-1,40	0,63	0,95	0,05	0,23
1,07	0,64764	-1,13512	-1,78	-0,19	-0,11	0,02	-0,40	-0,45
-0,84	-0,09807	0,16303	0,26	-1,39	0,23	-0,25	0,24	0,48
0,90	0,78335	0,04757	-0,74	0,08	0,23	-0,65	-0,39	0,33
0,95	0,59063	0,50857	-0,08	0,55	0,18	0,37	-0,60	-0,16
1,09	0,58190	0,37006	-0,21	0,65	1,51	0,60	-0,53	-0,45
-0,36	1,42203	0,67604	-0,75	2,27	3,34	-0,21	1,15	0,90
-0,32	1,21092	0,00186	-1,21	-0,92	-2,58	-0,15	0,54	0,49
0,51	0,24232	-0,57756	-0,82	0,94	-0,39	1,48	-0,82	-0,62
2,03	2,93687	0,79045	-2,15	0,06	0,95	-0,03	0,69	0,64
0,29	-0,83494	-0,09792	0,74	-0,71	-0,22	0,15	-0,09	0,26
#LEEG!	-1,31148	#LEEG!	#LEEG!	0,06	0,96	0,04	-0,28	-0,22
0,68	1,61048	-0,40222	-2,01	0,10	0,24	-0,13	0,54	0,32
#LEEG!	#LEEG!	0,09898	#LEEG!	-0,30	-0,47	-0,94	-0,12	0,49
1,16	1,69559	-0,06156	-1,76	#LEEG!	#LEEG!	#LEEG!	#LEEG!	-0,19
-0,40	-0,13948	-0,16178	-0,02	0,10	0,00	-0,44	0,24	0,20
-0,32	0,02874	0,32268	0,29	1,00	1,66	-1,00	0,70	0,60
2,10	1,08818	-0,17039	-1,26	1,05	-0,43	1,85	0,57	0,59
-0,02	0,91800	0,88797	-0,03	-0,88	-0,98	0,66	0,64	0,31
-0,08	0,58803	0,60102	0,01	0,31	0,11	-0,29	0,42	0,38
-0,24	0,14229	0,50679	0,36	0,45	-0,46	-0,02	0,77	0,49
0,48	0,59776	0,76156	0,16	-0,12	0,06	-0,29	-0,01	0,13
#LEEG!	-0,66677	#LEEG!	#LEEG!	-0,05	0,41	0,01	1,26	1,29
0,01	-0,44333	-0,57023	-0,13	0,52	0,11	-0,40	0,79	0,48
2,06	1,63823	0,35928	-1,28	-0,10	0,52	-0,18	-0,09	0,09
1,94	1,87428	-1,18168	-3,06	0,01	0,10	-0,39	0,26	0,17
-0,33	-0,64679	-0,66796	-0,02	-0,25	0,53	0,55	0,45	0,16
-0,90	-1,66953	-1,04853	0,62	-0,13	0,66	-0,10	-0,22	-0,22
0,22	0,11979	-0,51800	-0,64	#LEEG!	#LEEG!	#LEEG!	#LEEG!	#LEEG!
0,20	-1,29917	-2,09958	-0,80	-0,53	1,03	0,18	-0,23	-0,07
-0,39	0,19789	1,47809	1,28	1,33	-0,49	0,10	0,95	0,79
#LEEG!	-0,39251	#LEEG!	#LEEG!	#LEEG!	#LEEG!	#LEEG!	#LEEG!	#LEEG!
0,47	0,12340	0,48319	0,36	-0,07	-0,28	0,17	0,09	0,00
-0,63	-3,00603	-2,12455	0,88	#LEEG!	#LEEG!	#LEEG!	#LEEG!	#LEEG!
-0,79	0,12155	0,72570	0,60	0,57	-0,32	-0,18	0,59	0,61

-1,11	-1,19128	-0,81508	0,38	0,33	-0,15	-0,17	0,20	0,22
0,02	0,05771	-0,14295	-0,20	0,63	0,13	3,20	0,70	0,95
-0,75	0,59985	0,95067	0,35	0,20	0,06	0,13	0,25	0,36
-0,29	0,83249	0,70879	-0,12	0,50	1,00	0,11	0,80	0,65
0,00	-0,04008	0,12617	0,17	-3,12	-0,23	1,68	0,40	0,21
0,23	-1,41605	-0,97274	0,44	-1,11	2,04	0,18	-0,09	-0,17
1,13	0,00863	-0,38828	-0,40	0,09	0,48	0,01	-0,15	0,05
0,24	-1,02901	-0,38039	0,65	0,01	-0,01	-0,02	0,32	-0,09
0,31	-0,40066	1,13586	1,54	0,30	-0,04	-1,72	0,85	0,63
-0,15	0,23812	-0,70406	-0,94	-0,27	0,00	-0,45	0,26	0,28
0,76	0,42417	-0,57498	-1,00	0,43	0,10	-0,30	1,03	0,65
-0,23	-0,30526	-0,06958	0,24	-0,06	0,05	0,20	0,15	0,25
-1,14	-0,07898	0,47632	0,56	0,37	0,08	-0,12	0,48	0,46
-0,02	0,88414	0,64174	-0,24	0,13	0,39	0,29	0,02	-0,14
0,28	0,89321	0,10187	-0,79	0,05	-0,01	0,05	0,12	0,29
-0,55	-0,37203	0,33373	0,71	-0,04	-0,01	-0,02	1,02	0,92
0,19	-0,02849	-0,77326	-0,74	-0,03	-0,02	-0,46	-0,42	-0,15
0,42	0,11997	-0,51746	-0,64	-0,09	-0,80	0,04	0,64	0,55
0,31	-0,31108	0,04782	0,36	0,02	0,41	-0,03	0,41	0,22
0,47	-1,47201	-1,68900	-0,22	-1,09	0,01	0,90	-0,43	-0,59
0,18	0,41026	-0,71717	-1,13	0,00	0,03	-0,03	-0,23	0,34
-0,71	-1,28106	-1,60805	-0,33	-0,55	0,07	0,07	-0,82	-0,99
-0,48	-0,42694	0,04988	0,48	-0,99	-0,12	-1,39	0,07	-0,18
-0,52	1,13823	1,62530	0,49	0,18	-0,22	-0,34	0,26	0,67
0,56	0,22844	0,15474	-0,07	-0,06	-0,01	0,25	-0,08	-0,03
-0,46	0,22254	-0,05493	-0,28	-0,06	0,19	-0,11	-0,02	-0,09
1,69	1,23103	0,24982	-0,98	0,36	-0,76	-0,49	0,61	0,45
0,98	0,18211	-0,67970	-0,86	-0,28	-0,83	1,65	0,02	0,26
-0,94	-0,21503	-0,42626	-0,21	-0,90	-0,91	1,06	-0,05	0,20
-0,30	1,90154	0,84994	-1,05	-0,01	0,02	-0,24	0,17	0,03
-0,25	-0,21011	-0,22405	-0,01	-0,08	0,05	1,34	-0,32	-0,50
0,17	1,74785	2,37206	0,62	-1,98	-0,16	0,78	-0,09	0,24
0,40	1,31835	1,12418	-0,19	0,00	0,00	-0,23	0,17	0,10
0,36	1,27236	0,64096	-0,63	-0,52	0,69	0,57	0,12	-0,08
#LEEG!	-0,41140	#LEEG!	#LEEG!	0,00	-0,07	-0,01	-0,16	0,11
-0,37	1,12505	0,17132	-0,95	0,00	1,17	0,00	0,43	0,12
1,51	0,54280	0,05738	-0,49	-0,16	0,66	0,08	0,40	0,23
0,41	1,07853	1,27721	0,20	-1,67	-0,62	0,60	-0,07	0,22
#LEEG!	-0,02380	#LEEG!	#LEEG!	1,47	1,12	0,86	-0,78	-0,47
-0,07	0,21810	-0,41187	-0,63	0,08	0,28	0,05	0,16	0,16
-1,41	1,32421	0,54244	-0,78	0,69	0,01	0,62	0,54	0,77
-0,49	0,98616	0,66235	-0,32	0,03	-0,15	-0,03	0,12	-0,07
0,76	0,89746	2,34566	1,45	0,03	-0,18	-0,15	0,16	0,10
-0,51	2,19859	4,27077	2,07	0,07	-0,02	1,70	0,39	0,69
0,44	1,91516	1,74977	-0,17	0,26	0,06	0,70	0,54	0,43
0,00	-1,03785	0,98000	2,02	0,55	-2,55	0,62	-0,63	-0,72
1,36	1,34124	1,60188	0,26	-0,86	-0,68	-0,95	-0,13	-0,18
-0,42	0,44529	0,21352	-0,23	-0,27	0,27	-0,75	0,39	0,02
-0,93	1,09993	1,46322	0,36	0,00	-1,32	0,00	0,48	0,87
-0,76	0,08019	0,80736	0,73	0,36	0,04	-1,01	0,61	0,92

#LEEG!	1,29454	#LEEG!	#LEEG!	0,88	-0,55	2,34	0,74	1,05
-0,58	0,93679	0,82595	-0,11	-0,45	-0,76	-0,47	-0,46	-0,53
-1,25	-2,16236	0,09573	2,26	-0,21	0,74	-0,24	0,08	0,24
0,52	1,38282	1,94382	0,56	0,09	-0,47	0,00	-0,35	-0,20
-0,09	-2,18055	-2,67663	-0,50	2,85	-3,33	-3,37	-1,49	-0,75
-0,26	1,00290	0,53579	-0,47	-0,57	0,34	-0,44	-0,02	0,70
-0,37	-0,80076	0,69846	1,50	-1,44	-1,15	-0,40	-0,82	-0,35
-1,41	-2,73509	-2,02814	0,71	-2,42	-0,41	-1,09	-0,37	-0,65
0,39	-0,08747	-0,51851	-0,43	0,36	0,33	-0,22	0,34	0,19
-0,51	-0,33721	-0,13827	0,20	-0,53	-1,16	-0,26	-0,30	-0,16
0,06	-0,15976	-0,51965	-0,36	0,39	0,34	-0,04	-0,62	-0,35
#LEEG!	0,12508	#LEEG!	#LEEG!	-0,53	0,38	-1,44	-0,11	0,14
-0,73	-0,05839	-1,20533	-1,15	-0,20	-0,67	-0,12	-0,24	-0,62
-0,06	-3,12240	-1,02807	2,09	-0,43	-0,59	-0,10	-0,72	-0,93
0,24	-0,26884	-0,69994	-0,43	0,33	0,32	-0,37	-0,29	-0,24
-0,93	-0,44692	0,30198	0,75	0,71	0,29	-2,55	-0,49	-0,96
-0,35	-1,40964	0,19140	1,60	-0,89	0,26	1,33	-1,08	-0,27
0,92	0,13024	-1,69596	-1,83	-1,63	1,18	-0,61	0,02	-0,16
-1,98	-2,92888	-1,42622	1,50	1,44	-0,95	-1,47	-1,01	-0,94
0,00	-0,83770	-0,00379	0,83	-0,13	0,31	1,22	-0,35	-0,28
1,21	-0,61151	-0,51475	0,10	0,60	0,32	-0,36	-0,54	-0,14
0,94	1,73592	0,64560	-1,09	-0,44	0,39	0,91	-0,07	0,29
-1,72	-0,41308	-1,19901	-0,79	-2,31	0,58	-2,97	-0,59	-0,21
-0,69	-1,79462	-1,23521	0,56	1,46	0,27	-0,07	-1,10	-0,93
-0,96	-1,20556	-2,60202	-1,40	-0,55	-3,72	-0,08	-0,99	-0,87
-1,11	-0,57385	-0,36779	0,21	-0,25	0,03	-3,92	-0,41	0,18
1,24	-0,63954	-0,04529	0,59	-0,53	0,07	-3,67	-0,73	-0,03
#LEEG!	-1,50261	#LEEG!	#LEEG!	0,71	-0,28	-0,06	-0,87	-0,96
0,31	-0,54972	0,28095	0,83	1,26	1,01	0,76	-0,86	-0,62
1,15	-0,28316	-3,68691	-3,40	-0,50	0,02	-0,70	-0,44	-0,15
-0,67	-0,16062	0,24829	0,41	0,03	-0,01	0,12	-0,25	0,04
0,37	-0,02484	-0,59417	-0,57	0,58	-0,38	-0,40	-0,79	-0,55
-1,33	-0,51935	-0,48966	0,03	-0,46	-2,69	-0,25	-1,06	-0,83
-2,49	-1,51717	-0,00745	1,51	-1,48	0,64	-0,76	-0,59	-0,75
0,93	1,09440	-1,06753	-2,16	-0,02	0,04	-0,21	0,34	0,38
0,06	-0,08706	0,80546	0,89	-0,04	0,14	0,43	-0,36	-0,24
0,00	-1,53055	0,08822	1,62	-0,29	-0,03	-0,27	-0,45	-0,40
-0,29	-1,75669	-2,13924	-0,38	3,91	0,53	1,50	-1,39	-1,17
0,04	-0,60430	0,19812	0,80	1,20	2,36	-0,12	-0,86	-0,89
0,04	-1,63823	-0,93907	0,70	1,89	0,71	0,77	-1,16	-1,11
0,56	-0,71575	-0,40022	0,32	0,05	-0,03	-0,04	-0,10	-0,05
1,13	-0,19684	-1,46934	-1,27	-0,94	-1,16	-0,05	-0,41	-0,51
-0,51	0,91089	0,65181	-0,26	-0,17	1,19	-0,11	-0,23	-0,09
#LEEG!	1,16865	#LEEG!	#LEEG!	-0,56	-0,82	0,40	-0,28	-0,04
-0,31	-1,13194	0,09946	1,23	1,03	-0,48	0,68	-0,77	-0,95
-0,49	-0,22635	-0,46461	-0,24	-0,07	-0,05	0,05	-0,21	0,10
2,13	-0,47540	-1,04694	-0,57	0,03	-0,10	0,30	0,49	0,01
3,41	-0,70221	-1,53398	-0,83	0,06	-0,36	0,80	-0,65	-0,54

BL_GS_sub	M6_GS_tot	M6_GS_ob	M6_GS_sul	Change_GS	Change_GS	Change_GS_subjective_function
-1,36	-1,07	-0,93	-0,83	0,87	0,85	0,53
-0,23	-1,02	-0,68	-0,73	-0,60	-0,12	-0,50
0,55	0,45	0,19	0,29	0,22	-0,26	-0,26
#LEEG!	#LEEG!	#LEEG!	#LEEG!	#LEEG!	#LEEG!	#LEEG!
0,61	0,46	0,18	0,34	-0,48	-0,45	-0,27
#LEEG!	#LEEG!	#LEEG!	#LEEG!	#LEEG!	#LEEG!	#LEEG!
0,06	-0,46	-0,25	-0,28	-0,09	-0,08	-0,34
0,21	-0,17	0,28	-0,21	0,01	-0,24	-0,42
#LEEG!	#LEEG!	#LEEG!	#LEEG!	#LEEG!	#LEEG!	#LEEG!
#LEEG!	#LEEG!	#LEEG!	#LEEG!	#LEEG!	#LEEG!	#LEEG!
-0,43	-0,70	-0,38	-0,30	-0,16	0,18	0,12
0,30	-0,57	-0,09	-0,05	-0,95	-0,03	-0,35
-0,18	-1,31	-0,61	-0,61	-0,97	-0,03	-0,44
-0,80	-1,08	0,11	-0,64	-0,16	0,55	0,16
0,58	#LEEG!	#LEEG!	#LEEG!	#LEEG!	#LEEG!	#LEEG!
-0,47	-0,52	0,06	-0,29	0,12	0,39	0,19
#LEEG!	#LEEG!	#LEEG!	#LEEG!	#LEEG!	#LEEG!	#LEEG!
-0,51	-1,44	-0,95	-0,63	-0,79	-0,71	-0,13
#LEEG!	#LEEG!	#LEEG!	#LEEG!	#LEEG!	#LEEG!	#LEEG!
-0,16	-0,77	-0,19	-0,27	-0,50	0,20	-0,11
-0,01	0,19	-0,05	0,07	0,14	0,11	0,08
0,10	#LEEG!	#LEEG!	#LEEG!	#LEEG!	#LEEG!	#LEEG!
-0,14	-0,50	-0,37	-0,34	-0,17	-0,11	-0,20
0,14	0,26	0,47	0,02	-0,04	0,15	-0,12
0,08	0,61	0,52	0,39	0,57	0,47	0,31
-0,26	-0,94	-0,97	-0,61	-0,67	-0,82	-0,35
-0,11	-0,47	-0,26	-0,35	-0,37	-0,11	-0,23
0,02	0,66	0,84	0,27	0,61	0,67	0,25
0,19	-0,40	-0,38	-0,17	-0,73	-0,82	-0,36
-0,05	0,73	0,66	0,40	0,78	0,59	0,45
-0,11	0,35	0,49	0,18	0,71	0,70	0,30
0,16	0,47	0,40	0,28	0,33	0,46	0,12
-0,08	0,38	-0,26	0,37	0,52	-0,04	0,45
0,69	0,47	0,57	0,27	-0,47	-0,06	-0,42
-0,56	0,26	0,39	0,15	1,64	1,64	0,71
-0,34	0,26	0,08	0,18	0,67	0,09	0,52
0,29	0,57	0,83	0,19	-0,12	0,16	-0,10
-0,21	0,25	-0,02	0,14	0,39	-0,03	0,35
-0,42	#LEEG!	#LEEG!	#LEEG!	#LEEG!	#LEEG!	#LEEG!
0,39	#LEEG!	#LEEG!	#LEEG!	#LEEG!	#LEEG!	#LEEG!
0,37	0,73	0,58	0,50	0,46	0,51	0,13
-0,12	0,35	0,23	0,16	0,40	0,25	0,28
-0,29	0,47	0,44	0,21	0,63	0,23	0,50
0,03	-0,16	0,28	-0,34	-0,55	-0,18	-0,37
0,13	0,38	0,97	0,03	0,15	0,90	-0,10
0,27	0,67	0,40	0,46	0,10	-0,03	0,19
-1,09	0,02	0,59	-0,19	1,59	1,16	0,90
0,31	0,81	0,71	0,44	0,34	0,56	0,14
-0,22	0,19	0,11	0,10	0,75	0,70	0,32

-0,13	0,15	-0,08	0,05	0,33	0,15	0,18
0,29	0,26	0,28	-0,02	-0,36	-0,09	-0,31
-0,13	-0,08	-0,17	-0,09	0,19	0,40	0,03
-0,10	0,38	0,47	0,22	0,53	0,43	0,32
0,37	0,40	-0,06	0,49	-0,13	-0,38	0,12
0,68	0,52	0,42	0,21	-0,63	-0,26	-0,46
-0,31	0,30	0,20	0,09	0,94	0,89	0,40
0,57	0,89	0,86	0,54	0,04	0,16	-0,03
0,32	0,23	0,24	0,19	-0,21	-0,24	-0,13
0,12	0,39	0,03	0,24	0,24	0,31	0,12
0,42	0,90	1,03	0,48	0,18	0,41	0,06
0,19	-0,17	0,09	-0,16	-0,63	-0,33	-0,36
-0,03	-0,61	-0,11	-0,56	-0,73	-0,56	-0,53
0,49	0,08	0,02	0,04	-0,76	-0,67	-0,45
-0,31	0,07	0,24	-0,13	0,27	0,15	0,17
0,15	-0,17	-0,13	-0,17	-0,44	-0,10	-0,32
0,40	0,51	0,50	0,32	-0,27	-0,23	-0,09
0,47	-0,46	-0,42	-0,25	-0,97	-0,67	-0,73
0,33	#LEEG!	0,78	0,22	#LEEG!	0,24	-0,11
-0,25	-0,37	-0,15	-0,38	-0,30	-0,15	-0,13
0,55	0,75	0,20	0,59	0,06	-0,10	0,04
-0,29	0,19	0,03	0,07	0,70	0,67	0,36
-0,20	0,32	0,23	0,18	0,44	-0,06	0,38
-0,15	1,23	0,94	0,79	1,33	0,67	0,95
0,40	-1,22	-0,93	-0,40	-1,73	-0,99	-0,79
-0,10	0,04	0,10	-0,08	0,14	0,21	0,02
-0,09	0,21	0,41	0,02	0,18	0,23	0,11
-0,50	#LEEG!	#LEEG!	#LEEG!	#LEEG!	#LEEG!	#LEEG!
0,01	0,65	0,33	0,44	0,60	0,44	0,42
-0,18	0,25	-0,08	0,33	0,57	0,29	0,51
-0,35	-1,07	-0,89	-0,61	-0,38	-0,10	-0,26
0,69	-0,01	0,27	-0,16	-1,01	-0,20	-0,85
-0,25	#LEEG!	#LEEG!	#LEEG!	#LEEG!	#LEEG!	#LEEG!
-0,31	#LEEG!	#LEEG!	#LEEG!	#LEEG!	#LEEG!	#LEEG!
0,38	0,48	0,74	0,06	-0,09	0,43	-0,33
-0,48	-0,14	0,10	-0,26	0,24	0,15	0,22
0,26	0,54	0,73	0,22	0,09	0,24	-0,04
0,03	0,25	0,53	0,06	0,12	0,27	0,02
0,27	0,44	0,87	-0,03	0,00	0,60	-0,30
0,61	0,49	0,59	0,19	-0,42	-0,13	-0,42
-0,24	-0,22	-0,64	-0,06	0,01	-0,19	0,18
0,09	0,63	0,59	0,30	0,30	0,16	0,21
-0,62	-0,18	-0,10	-0,19	0,55	0,46	0,43
0,29	0,29	0,38	0,26	-0,29	-0,38	-0,03
0,13	0,19	0,43	0,01	-0,08	0,47	-0,12
0,34	1,16	0,94	0,70	0,45	0,48	0,36
#LEEG!	#LEEG!	#LEEG!	#LEEG!	#LEEG!	#LEEG!	#LEEG!
0,36	0,75	0,73	0,40	0,24	0,32	0,04
-0,62	-1,03	-0,34	-0,81	-0,37	-0,14	-0,19
#LEEG!	#LEEG!	#LEEG!	#LEEG!	#LEEG!	#LEEG!	#LEEG!

-0,13	0,17	0,24	0,08	0,32	0,06	0,21
-0,30	-0,51	-0,26	-0,45	-0,14	0,17	-0,16
0,49	1,07	1,06	0,52	0,12	0,24	0,03
0,02	0,07	0,22	-0,12	-0,16	-0,01	-0,14
0,21	0,02	-0,15	0,01	-0,45	-0,37	-0,20
0,23	1,00	0,52	0,73	0,49	-0,03	0,50
0,17	0,58	0,46	0,31	0,16	0,20	0,14
0,10	#LEEG!	#LEEG!	#LEEG!	#LEEG!	#LEEG!	#LEEG!
0,39	0,86	0,81	0,46	0,05	-0,09	0,07
0,68	1,23	0,86	0,87	0,33	0,44	0,19
0,38	0,85	0,41	0,61	0,53	0,53	0,23
0,36	0,13	0,19	0,11	-0,22	-0,12	-0,25
0,75	1,41	0,90	0,98	0,25	0,14	0,23
0,02	-0,26	-0,16	-0,23	-0,35	0,05	-0,25
0,27	0,25	0,55	0,04	-0,21	0,24	-0,23
0,61	0,09	0,37	-0,06	-0,85	-0,32	-0,67
0,09	0,29	0,43	0,01	-0,02	-0,01	-0,07
-0,06	0,40	0,54	0,18	0,31	0,03	0,24
0,23	0,24	0,17	0,08	-0,12	-0,08	-0,15
0,40	0,00	-0,16	-0,02	-0,64	-0,55	-0,42
0,02	0,65	0,88	0,27	0,46	0,57	0,25
0,53	0,63	0,12	0,58	-0,11	-0,25	0,05
0,08	#LEEG!	#LEEG!	#LEEG!	#LEEG!	#LEEG!	#LEEG!
0,39	0,82	0,81	0,45	0,24	0,42	0,07
0,49	0,73	0,31	0,50	0,02	0,01	0,01
0,67	0,65	0,81	0,29	-0,47	-0,10	-0,38
-0,80	0,60	0,65	0,19	1,71	1,61	0,99
-0,23	0,33	0,01	0,29	0,50	0,09	0,52
-0,49	-0,12	0,25	-0,31	0,54	0,75	0,18
-0,33	-0,68	0,11	-0,58	-0,34	0,39	-0,25
-0,09	-0,47	-0,34	-0,49	-0,43	-0,27	-0,40
0,72	0,98	1,02	0,54	-0,18	0,22	-0,18
-0,45	0,15	0,48	-0,13	0,66	0,95	0,32
0,36	0,46	0,48	0,15	-0,19	0,04	-0,20
0,00	-0,15	0,14	-0,36	-0,52	-0,77	-0,36
0,06	0,48	0,55	0,11	0,16	0,20	0,06
-0,13	0,47	0,36	0,21	0,36	0,23	0,33
0,33	0,06	0,17	-0,10	-0,72	-0,41	-0,42
0,30	0,86	0,52	0,56	0,38	0,23	0,26
0,01	#LEEG!	#LEEG!	#LEEG!	#LEEG!	#LEEG!	#LEEG!
0,31	0,56	0,78	0,26	0,02	0,17	-0,05
-0,42	-0,33	-0,09	-0,44	-0,12	-0,30	-0,02
0,25	0,18	0,12	-0,01	-0,30	-0,05	-0,25
0,17	-0,31	-0,28	-0,22	-0,65	-0,49	-0,39
0,03	0,51	0,45	0,24	0,31	0,41	0,21
-0,68	-0,73	0,42	-0,77	-0,08	0,59	-0,09
0,12	0,34	0,08	0,13	0,27	0,30	0,01
0,31	0,91	0,82	0,41	0,39	0,06	0,11
-0,52	-0,23	0,17	-0,27	0,50	0,31	0,25
-0,38	-0,67	-0,27	-0,56	-0,22	-0,17	-0,19

-0,24	0,38	0,31	0,21	0,72	0,52	0,45
0,48	1,00	1,04	0,52	0,16	0,16	0,04
0,37	0,22	0,44	-0,08	-0,69	-0,41	-0,46
-0,22	-0,06	0,29	-0,21	0,12	-0,01	0,01
-0,90	-0,76	-0,39	-0,70	0,36	0,00	0,20
0,12	0,24	0,04	0,19	0,21	0,25	0,07
0,23	0,09	0,95	0,29	-0,41	0,60	0,06
0,98	1,06	0,71	0,62	-0,36	-0,21	-0,36
-0,01	-0,34	-0,16	-0,32	-0,37	-0,27	-0,31
#LEEG!	#LEEG!	#LEEG!	#LEEG!	#LEEG!	#LEEG!	#LEEG!
-0,04	-0,31	-0,17	-0,26	-0,14	0,32	-0,22
-0,41	-0,42	0,73	-0,43	0,22	0,92	-0,02
#LEEG!	#LEEG!	#LEEG!	#LEEG!	#LEEG!	#LEEG!	#LEEG!
0,17	0,11	0,48	0,39	-0,09	0,30	0,22
-0,63	0,03	0,57	-0,23	0,88	0,89	0,40
0,06	-0,13	0,39	0,16	-0,18	0,15	0,10
-0,18	#LEEG!	-0,26	-0,06	#LEEG!	0,18	0,11
-0,02	-0,55	0,03	-0,30	-0,79	-0,46	-0,28
-0,36	-0,72	0,12	-0,31	-0,33	-0,21	0,05
-0,64	-0,59	0,06	-0,36	0,01	0,22	0,28
-0,41	-0,35	0,16	-0,14	0,19	0,60	0,27
0,66	-0,01	0,77	0,16	-1,16	-0,13	-0,51
0,17	-0,30	-0,16	0,03	-0,83	-0,65	-0,14
-0,64	-0,95	-0,17	-0,52	-0,13	0,45	0,12
0,33	0,97	1,18	0,43	0,29	0,55	0,11
-0,25	0,40	0,79	0,04	0,49	0,53	0,29
-0,27	#LEEG!	#LEEG!	#LEEG!	#LEEG!	#LEEG!	#LEEG!
0,37	0,52	0,65	0,29	-0,03	0,33	-0,08
-0,32	#LEEG!	#LEEG!	#LEEG!	#LEEG!	#LEEG!	#LEEG!
#LEEG!	0,32	0,14	0,19	#LEEG!	0,34	#LEEG!
0,13	-0,40	0,04	-0,05	-0,64	-0,16	-0,18
0,44	0,31	0,66	0,57	-0,40	0,06	0,13
0,37	0,48	0,62	0,35	-0,09	0,03	-0,02
0,30	0,86	0,53	0,51	0,21	0,21	0,21
0,33	0,42	0,20	0,24	0,00	-0,18	-0,09
0,48	0,69	0,61	0,31	-0,07	0,12	-0,17
-0,06	-0,31	-0,15	-0,30	-0,30	-0,28	-0,23
0,66	#LEEG!	#LEEG!	#LEEG!	#LEEG!	#LEEG!	#LEEG!
0,55	0,55	-0,11	0,41	-0,24	-0,59	-0,13
-0,08	0,21	0,39	0,50	0,30	0,30	0,59
0,08	-0,70	-0,58	-0,19	-0,96	-0,75	-0,27
0,34	0,20	0,14	0,08	-0,25	-0,02	-0,25
-0,16	0,18	-0,15	0,17	0,40	0,07	0,33
#LEEG!	#LEEG!	#LEEG!	#LEEG!	#LEEG!	#LEEG!	#LEEG!
-0,15	-0,72	-0,21	-0,56	-0,50	-0,14	-0,41
0,61	1,17	0,92	0,75	0,22	0,14	0,14
#LEEG!	#LEEG!	#LEEG!	#LEEG!	#LEEG!	#LEEG!	#LEEG!
-0,05	-0,38	-0,04	-0,11	-0,46	-0,05	-0,06
#LEEG!	#LEEG!	#LEEG!	#LEEG!	#LEEG!	#LEEG!	#LEEG!
0,30	0,73	0,65	0,38	0,14	0,04	0,08

0,10	0,60	0,60	0,87	0,40	0,38	0,77
0,44	0,50	0,76	0,67	-0,20	-0,19	0,24
0,09	-0,37	0,05	-0,17	-0,62	-0,31	-0,26
0,55	0,71	0,56	0,35	-0,09	-0,09	-0,20
0,18	0,23	0,20	0,07	-0,17	0,00	-0,11
-0,03	-0,10	-0,31	-0,01	-0,01	-0,14	0,01
-0,19	0,15	0,18	0,05	0,30	0,13	0,24
0,24	-0,20	0,35	0,25	-0,52	0,43	0,00
0,56	0,65	0,83	0,69	-0,20	0,20	0,13
0,05	0,46	0,44	0,19	0,20	0,16	0,13
0,59	0,62	0,58	0,28	-0,41	-0,08	-0,31
0,07	-0,34	-0,07	-0,02	-0,49	-0,32	-0,09
0,21	0,39	0,51	0,16	-0,10	0,05	-0,06
-0,03	0,15	-0,05	0,06	0,14	0,10	0,09
0,02	#LEEG!	#LEEG!	#LEEG!	#LEEG!	#LEEG!	#LEEG!
0,55	1,07	0,99	0,58	0,05	0,07	0,03
-0,38	-0,65	-0,13	-0,52	-0,22	0,03	-0,14
0,30	0,73	0,20	0,52	0,09	-0,34	0,21
0,20	-0,22	0,27	0,09	-0,63	0,05	-0,11
-0,48	-0,43	-0,43	-0,42	0,00	0,16	0,05
0,03	0,59	0,78	0,70	0,82	0,44	0,67
-0,31	-1,20	-0,91	-0,52	-0,37	0,09	-0,21
0,18	0,43	0,50	0,61	0,36	0,68	0,44
0,46	0,44	0,78	0,56	0,19	0,12	0,10
-0,17	-0,03	0,19	-0,14	0,05	0,22	0,03
-0,01	-0,21	-0,33	-0,13	-0,19	-0,24	-0,12
0,31	0,15	0,28	-0,01	-0,46	-0,17	-0,32
0,10	-0,25	-0,05	-0,15	-0,28	-0,30	-0,25
-0,28	-0,44	-0,27	-0,32	-0,39	-0,47	-0,04
0,12	0,19	0,03	0,15	0,03	0,01	0,03
-0,30	0,15	0,16	-0,03	0,47	0,65	0,26
-0,35	0,29	0,52	-0,02	0,39	0,28	0,32
0,07	0,01	0,01	-0,02	-0,16	-0,09	-0,10
0,11	-0,11	-0,20	-0,13	-0,23	-0,12	-0,25
-0,23	#LEEG!	#LEEG!	#LEEG!	#LEEG!	#LEEG!	#LEEG!
0,19	0,47	0,56	0,21	0,03	0,44	0,02
0,23	0,07	-0,07	0,06	-0,33	-0,30	-0,17
-0,06	-0,07	0,55	0,14	0,01	0,33	0,20
-0,65	-1,76	-0,86	-1,13	-0,98	-0,39	-0,49
0,04	0,44	0,32	0,22	0,28	0,16	0,18
0,36	0,17	0,62	-0,14	-0,37	-0,16	-0,50
0,08	0,48	0,16	0,35	0,36	0,23	0,28
0,07	-0,18	-0,07	0,19	-0,34	-0,17	0,12
0,21	#LEEG!	#LEEG!	#LEEG!	#LEEG!	#LEEG!	#LEEG!
0,43	-0,23	0,33	0,22	-0,77	-0,11	-0,20
-0,27	#LEEG!	#LEEG!	#LEEG!	#LEEG!	#LEEG!	#LEEG!
-0,03	-0,24	-0,16	0,35	-0,11	0,02	0,38
0,27	-0,43	-0,11	0,02	-0,82	-0,12	-0,25
0,12	-0,22	0,88	-0,01	-0,70	0,01	-0,14
0,26	0,03	0,29	0,27	-0,58	-0,63	0,01

0,42	#LEEG!	#LEEG!	#LEEG!	#LEEG!	#LEEG!	#LEEG!
-0,20	-0,58	-0,28	-0,07	-0,12	0,25	0,13
-0,08	-0,09	0,54	0,03	-0,17	0,30	0,10
-0,23	-0,23	0,24	0,16	0,11	0,44	0,39
-0,95	-1,96	-0,97	-1,26	-0,47	-0,22	-0,31
-0,30	0,06	-0,09	-0,10	0,09	-0,79	0,19
-0,57	-0,74	-0,29	-0,66	0,08	0,06	-0,10
-0,08	-0,93	-0,97	-0,60	-0,56	-0,32	-0,52
0,21	0,03	-0,21	-0,10	-0,32	-0,41	-0,31
-0,23	-0,66	-0,24	-0,38	-0,36	-0,08	-0,15
-0,46	0,16	-0,07	0,19	0,78	0,29	0,65
-0,19	#LEEG!	#LEEG!	#LEEG!	#LEEG!	#LEEG!	#LEEG!
-0,03	-0,67	-0,66	-0,38	-0,43	-0,04	-0,35
-0,34	-0,34	-0,33	-0,16	0,38	0,61	0,18
-0,30	-0,16	-0,23	-0,22	0,13	0,02	0,08
-0,33	-0,81	-0,57	-0,57	-0,33	0,39	-0,24
-0,65	-0,53	-0,16	-0,47	0,55	0,10	0,18
0,10	-1,41	-1,11	-0,79	-1,43	-0,95	-0,89
-0,58	-1,22	-1,06	-0,57	-0,22	-0,12	0,01
-0,36	-0,39	-0,15	-0,46	-0,04	0,13	-0,10
-0,51	-0,59	0,16	-0,47	-0,04	0,30	0,04
-0,13	0,17	-0,18	0,13	0,24	-0,47	0,26
-0,54	-0,66	-0,70	-0,40	-0,07	-0,49	0,14
-0,78	-0,62	-0,69	-0,34	0,48	0,24	0,44
-0,40	-1,40	-1,37	-0,67	-0,41	-0,50	-0,27
-0,38	-0,48	-0,58	-0,28	-0,07	-0,76	0,10
-0,66	-0,45	-0,36	-0,39	0,28	-0,33	0,27
-0,63	#LEEG!	#LEEG!	#LEEG!	#LEEG!	#LEEG!	#LEEG!
-0,68	-0,55	-0,63	-0,37	0,31	-0,01	0,32
-0,40	#LEEG!	#LEEG!	#LEEG!	#LEEG!	#LEEG!	#LEEG!
-0,33	0,03	0,05	-0,04	0,28	0,01	0,28
-0,46	-0,86	-0,74	-0,56	-0,07	-0,18	-0,11
-0,59	-0,90	-0,48	-0,59	0,16	0,34	0,01
-0,25	-0,02	-0,07	0,03	0,57	0,67	0,28
0,15	-0,72	-0,36	-0,54	-1,06	-0,74	-0,69
-0,28	-0,16	-0,12	-0,07	0,20	0,12	0,21
-0,37	-0,40	-0,57	-0,29	0,05	-0,17	0,08
-0,93	-1,78	-1,32	-1,24	-0,39	-0,15	-0,31
-0,56	-0,32	-0,41	-0,13	0,54	0,48	0,43
-0,69	-1,18	-1,19	-0,65	-0,03	-0,08	0,04
-0,09	0,07	-0,06	0,05	0,17	-0,01	0,14
-0,21	-0,60	-0,41	-0,39	-0,19	0,09	-0,18
-0,21	-0,39	0,04	-0,38	-0,16	0,12	-0,18
-0,38	#LEEG!	#LEEG!	#LEEG!	#LEEG!	#LEEG!	#LEEG!
-0,49	-0,40	-0,40	-0,29	0,37	0,55	0,20
-0,28	-1,22	0,15	-0,97	-1,01	0,06	-0,69
0,42	-0,32	-0,13	-0,33	-0,81	-0,15	-0,74
-0,39	-0,60	-0,37	-0,49	0,05	0,16	-0,09
